# Metabolities from Marine Sponges of the Genus *Callyspongia*: Occurrence, Biological Activity, and NMR Data

**DOI:** 10.3390/md19120663

**Published:** 2021-11-26

**Authors:** Lucas Hilário Nogueira de Sousa, Rusceli Diego de Araújo, Déborah Sousa-Fontoura, Fabrício Gava Menezes, Renata Mendonça Araújo

**Affiliations:** 1 Instituto de Química, Universidade Federal do Rio Grande do Norte, Natal 59078-970, Brazil; lucas_hilario@live.com (L.H.N.d.S.); rusceli@gmail.com (R.D.d.A.); fabricio.menezes@ufrn.br (F.G.M.); 2 Biotério Central, Universidade Federal do Rio Grande do Norte, Natal 59078-970, Brazil; deborah.fontoura@ufrn.br

**Keywords:** demosponges, *Callyspongia*, polyacetylenes, anticancer action

## Abstract

The genus *Callyspongia* (Callyspongiidae) encompasses a group of demosponges including 261 described species, of which approximately 180 have been accepted after taxonomic reviews. The marine organisms of *Callyspongia* are distributed in tropical ecosystems, especially in the central and western Pacific, but also in the regions of the Indian, the West Atlantic, and the East Pacific Oceans. The reason for the interest in the genus *Callyspongia* is related to its potential production of bioactive compounds. In this review, we group the chemical information about the metabolites isolated from the genus *Callyspongia*, as well as studies of the biological activity of these compounds. Through NMR data, 212 metabolites were identified from genus *Callyspongia* (15 species and *Callyspongia* sp.), belonging to classes such as polyacetylenes, terpenoids, steroids, alkaloids, polyketides, simple phenols, phenylpropanoids, nucleosides, cyclic peptides, and cyclic depsipeptides. A total of 109 molecules have been reported with bioactive activity, mainly cytotoxic and antimicrobial (antibacterial and antifungal) action. Thus, we conclude that polyacetylenes, terpenoids and steroids correspond to the largest classes of compounds of the genus, and that future research involving the anticancer action of the species’ bioactive metabolites may become relevant.

## 1. Introduction

The genus *Callyspongia* Duchassaing and Michelotti, 1864, belonging to the family Callyspongiidae and the order Haplosclerida, is structured in six subgenera: *Callyspongia* (*Callyspongia*) Duchassaing and Michelotti, *Callyspongia* (*Cavochalina*) Carter, *Callyspongia* (*Cladochalina*) Schmidt, *Callyspongia* (*Euplacella*) Lendenfeld, *Callyspongia* (*Toxochalina*) Ridley, and *Callyspongia* (*Spinosella*) Vosmaer [1,2]. This group of demosponges includes 261 described species and approximately 180 accepted by taxonomic review [3,4].

The marine organisms of *Callyspongia* are distributed in tropical ecosystems, especially in the Central and Western Pacific [1,5,6]. They can also be seen in regions of the Indian Ocean, the West Atlantic Ocean, and the East Pacific Ocean, such as Indonesia [4], the Red Sea [7,8], Cuba [3], Barbados [9], Brazil [10,11], and Ecuador [12]. Because of this, the great variety of species allows the existence of new studies, but it also generates a large amount of data, which can cause confusion in research due to the accumulation of information.

Sponge species have their particularities, but they also have common characteristics. Regarding sponges of the genus *Callyspongia*, their regular ectosomal tangential reticulation (formed mainly by primary and secondary spongin fibers, but also by tertiary ones) identifies them [13]. In general, marine organisms produce compounds with enormous diversity and structural complexity resulting from the chemical strategies of their secondary metabolism to adapt to the extreme and competitive conditions of the marine environment [14,15]. NMR spectroscopy is the most important tool for structural elucidation of natural products, and it have been efficiently used to characterize the complex marine-derived molecules [16]. A compilation of the ^13^C NMR data for a plant or animal genus optimizes the exhaustive structural elucidation process.

As confirmed by biological studies, *Callyspongia*’s species are very rich sources of bioactive compounds. Several classes of primary and secondary metabolites have been associated with the genus, such as fatty acids [17], alkaloids [18], steroids [19], nucleosides [20], peptides [4], polyacetylenes [21], and terpenoids [11]. Furthermore, molecules isolated from these species are found to present relevant biological activities, including antibacterial [7], antituberculosis [22], anti-inflammatory [19], antimalarial [23], and cytotoxic [7,12,24].

A respectable number of publications focusing on isolation, structural characterization, and bioactivity of species from the *Callyspongia* genus are reported in the literature. However, to the best of our knowlegment, the genus *Callyspongia* lacks in deeper discussion on structural aspects and biological activities. Therefore, this review aims to fill a relevant gap associated with the occurrence and frequency of several metabolites isolated from species from the *Callyspongia* genus in the last 40 years [25,26], as well as to present a prospection and compilation of Nuclear Magnetic Resonance (NMR) spectroscopy data of these molecules, which can be employed as a library for further studies. Additionally, this work presents a survey of their biological activities, which magnifies the relevance of the *Callyspongia* genus in relation to development in the field of natural products, and its significance in the development of nature-based bioactive compounds.

## 2. Chemical Aspects of *Callyspongia* species

NMR spectroscopy-based studies on *Callyspongia* unidentified species (*Callyspongia* sp.) along with other 15 identified species (*C*. *abnormis*, *C*. *aerizusa*, *C*. *bilamellata*, *C*. *californica*, *C*. *diffusa*, *C*. *fibrosa*, *C*. *fistularis*, *C*. *flammea*, *C*. *implexa*, *C*. *lindgreni*, *C*. *pseudoreticulata*, *C*. *siphonella*, *C*. *spinosissima*, *C*. *truncata* and *C*. *vaginalis*) resulted in the structural characterization of 212 isolated metabolites from different classes: polyacetylenes; terpenoids and steroids; alkaloids; simple phenols and phenylpropanoids; nucleosides; cyclic peptides and cyclic depsipeptides; polyketides; and miscellaneous.

These substances were described according to the extract used in the isolation, relevant structural characteristics, and the elucidation data based on NMR data. This information is presented in Tables S1–S8 together with additional information such as chemical formula, type of metabolite, one-dimensional NMR data, geographic location, and references related to the compound obtention in *Callyspongia* species. Regarding the 1D NMR data, the chemical shifts, the solvent and frequency used in process, and the coupling constant of all compounds, were investigated. In addition, although NMR was the only spectroscopic information reported in this study, mainly due to the large volume of data, other techniques were used in the primary studies to support structural identification and elucidation, such as: specific rotation, X-ray crystallography, Thin-Layer Chromatography (TLC), melting point, two-dimensional NMR spectroscopy, Mass Spectrometry (EM), and spectroscopy in the infrared (IR) and ultraviolet (UV) regions.

### 2.1. Polyacetylenes

**Figure 1:**
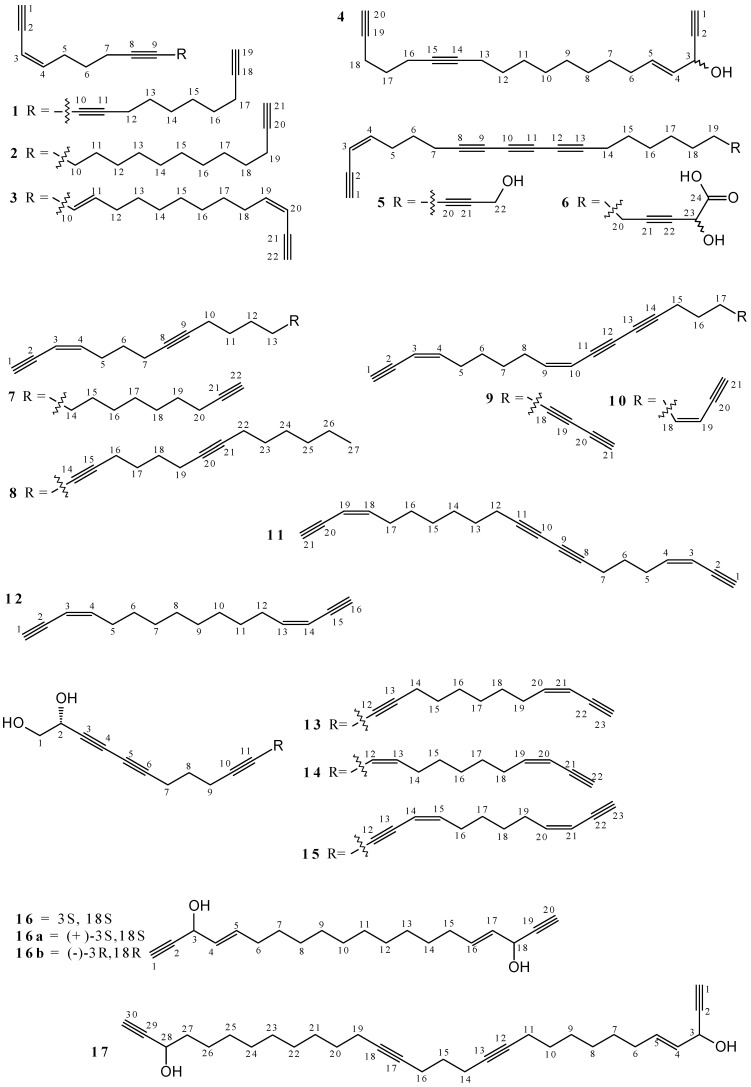

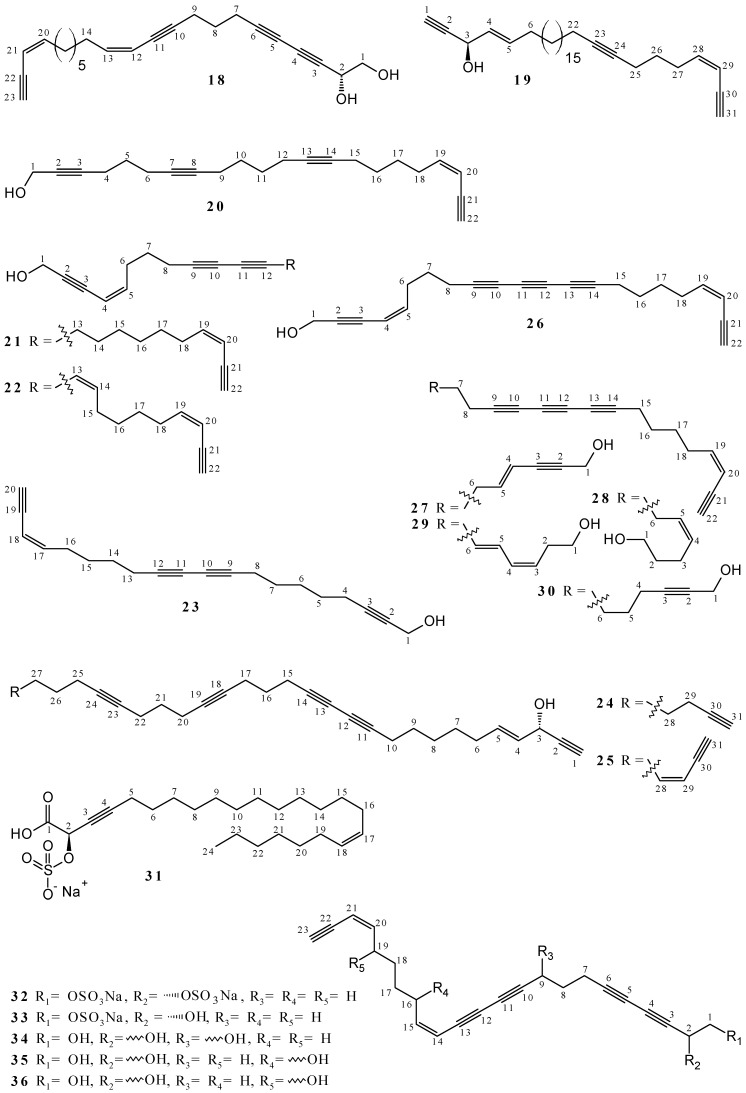

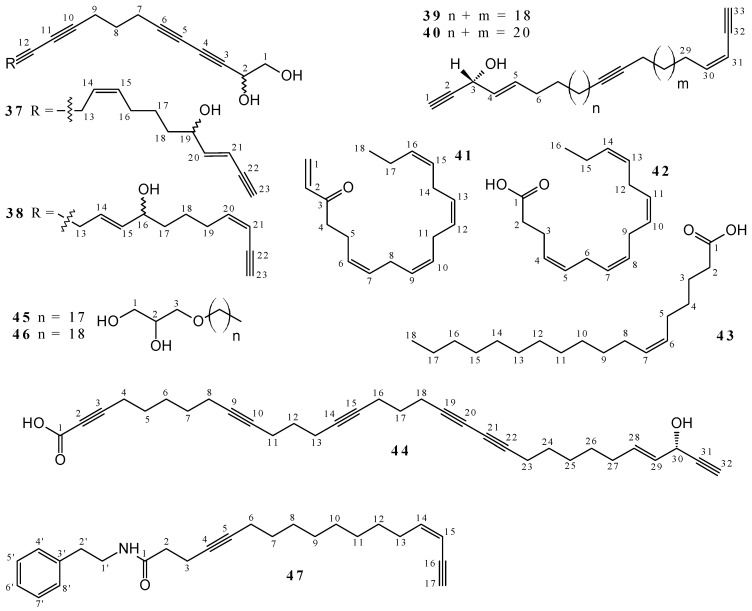
Structures of polyacetylenes isolated from *Callyspongia* species.

The polyacetylenes aikupikanynes A (**1**), B (**2**) and C (**3**), D (**4**), E (**5**) and F (**6**) and octahydrosiphonochalyne (**7**) were isolated from methanol (MeOH) extract of *Callyspongia* sp., a red sea sponge [27]. Other metabolites were also isolated: callimplexen A (**8**) from *Callyspongia implexa* (MeOH/Dichloromethane (CH_2_Cl_2_) 1:1 extract) [28]; callyberynes A (**9**), B (**10**) and C (**11**) from *Callyspongia* sp. (MeOH/CH_2_Cl_2_ 3:1 extract) [21]; **9** and **11** from *Callyspongia truncata* (MeOH extract) [29]; and the diacetylene Callydiyne (**12**) from *Callyspongia flammea* (MeOH extract) [30]. Polyacetylenes **1**–**12** (Figure 1 and Table S1) were elucidated by ^1^H and ^13^C NMR and have unsaturated hydrocarbon moieties associated with olefinic and alkynyl double and triple bonds, respectively. The only symmetrical compound is **12** and structures **4**, **5** and **6** have characteristics of fatty acyls.

Six polyacetylene diols were obtained from studies based on *Callyspongia* genus. 14,15-dihydrosiphonodiol (**13**), Callyspongidiol (**14**) and siphonodiol (**15**) were isolated from Ethyl acetate (EtOAc) extract of *Callyspongia* sp. [31]; **13** and **15** from ethanol (EtOH) extract of *Callyspongia lindgreni* [32]; from these later, only **15** from *Callyspongia lindgreni* (CH_2_Cl_2_ extract) [33] and *Callyspongia truncata* (MeOH extract) [29]. Two isomeric structures were isolated from *Callyspongia* sp. (EtOH extract): (3*S*,18*S*,4*E*,16*E*)-eicosa-1,19-diyne-3,18-diol-4,16-diene (**16a**) and (−)-(4*E*,16*E*)-icosa-4,16-diene-1,19-diyne-3,18-diol (**16b**). Compound **16a** has also been identified in *Callyspongia pseudoreticulata* (MeOH extract) [34,35]. In addition, callyspongendiol (**17**) was isolated from *Callyspongia siphonella* (CH_2_Cl_2_/MeOH 1:1 extract) [8,36], and Tetrahydrosiphonodiol (**18**) from *Callyspongia lindgreni* (EtOH extract) [32]. Polyacetylene Diols **13**–**18** are open chain unsaturated hydrocarbons (Figure 1 and Table S1) that have their structures elucidated by ^1^H and ^13^C NMR. The regiochemistry patterns for the two hydroxyls in the structures vary considerably depending on the metabolite, having close proximity in **13**, **14**, **15** and **18**. Isomers **16a** and **16b** are the only structures with symmetric atom connectivity; they differ from each other according to the configuration of stereogenic centers.

A total of 12 polyacetylene alcohols were obtained from *Callyspongia* species: (3*R*,4*E*,28*Z*)-hentriacont-4,28-diene-1,23,30-triyn-3-ol (**19**), Callyspongenols A (**20**), B (**21**), C (**22**) and D (**23**), Callysponynes A (**24**) and B (**25**), dehydroisophonochalynol (**26**), siphonellanols A (**27**), B (**28**) and C (**29**) and siphonchalynol (**30**) (Figure 1 and Table S1). Studies involving *Callyspongia* sp. afforded different metabolites depending on the solvent used in the extraction: acetone (**19**) [37], MeOH/CH_2_Cl_2_ 1:1 (**20**–**22** and **26**) [38] and EtOAc (**24** and **25**) [39] extracts; while those related to *Callyspongia siphonella* were obtained from MeOH/CH_2_Cl_2_ 1:1 (**23** and **26**) [8,36] and MeOH (**26**–**30**) [40] extracts. The polyacetylene alcohols were elucidated by ^1^H and ^13^C NMR, but only **19**–**29** present elucidative data.

Studies involving *Callyspongia truncata* resulted in obtaining the acetylenic sulfate fatty acid callysponginol sulfate A (**31**) from a mixture of H_2_O, MeOH, CHCl_3_, and EtOAc extracts [41]. The methanolic extract provided callyspongins A (**32**) and B (**33**) [29,42], as well as callytriols A (**34**), B (**35**), C (**36**), D (**37**), and E (**38**) [29]. The polyacetylene lipids callyspongynes A (**39**) and B (**40**) were also isolated from an ethanolic extract of *Callyspongia* sp. [43]. The metabolites **32**–**40** were elucidated by ^1^H and ^13^C NMR and have an oxygenated and unsaturated aliphatic structure with double and triple bonds (Figure 1 and Table S1). Compounds **32** and **33** are derived from siphonodiol and along with **31** are classified as sulfated compounds. Metabolites **34**–**38** have three hydroxyls, while **39** and **40** are simple monoalcohol.

Four metabolites were isolated from ethanolic extracts from different species: (6*Z*,9*Z*,12*Z*,15*Z*)-1,6,9,12,15-octadecapenten-3-one (**41**) (*Callyspongia sp.*) [17], (4*Z*,7*Z*,10*Z*,13*Z*)-4,7,10,13-hexadecatetraenoic acid (**42**) (*Callyspongia sp.*) [17], petroselenic acid (**43**) (*Callyspongia siphonella*) [7], and callyspongynic Acid (**44**) (*Callyspongia truncata*) [44]. In addition, glycerolipid 3-octadecyloxy-propane-1,2-diol (**45**) was obtained from 95% EtOH + MeOH/CH_2_Cl_2_ 1:1 extracts [45], and batyl alcohol (**46**) from methanolic extract, both from *Callyspongia fibrosa* [23]; the polyacetylenic amide callyspongamide A (**47**) was isolated from *Callyspongia fistularis* (MeOH/CH_2_Cl_2_ 1:1 extract) [46,47,48]. Among the elucidated compounds, only **41**, **44**, **45**, and **47** have ^1^H and ^13^C NMR data reported. Compound **46** was characterized by ^1^H NMR only, while **41** and **44**–**47** present the spectroscopic data. The metabolites are structurally distinct, but some similarities are visible (Figure 1 and Table S1). Substance **41** has a conjugated ketone system, while **42**–**44** have carboxyl groups, among which **44** also has a hydroxyl unit. Glycerolipids **45** and **46** are the only saturated compounds having hydroxyls and ether oxygen, with the only structural difference between them being the presence of an additional methylene unit in **45**. Double and triple bonds, an aromatic unit, and an amide form compound **47**.

### 2.2. Terpenoids and Steroids

**Figure 2:**
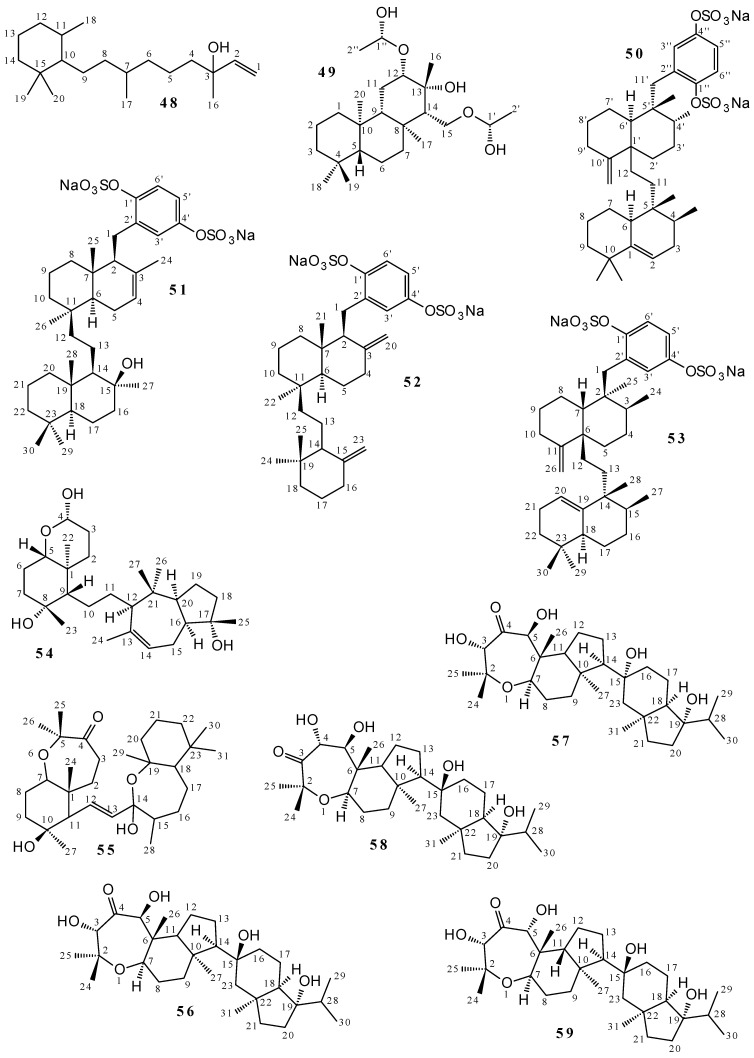

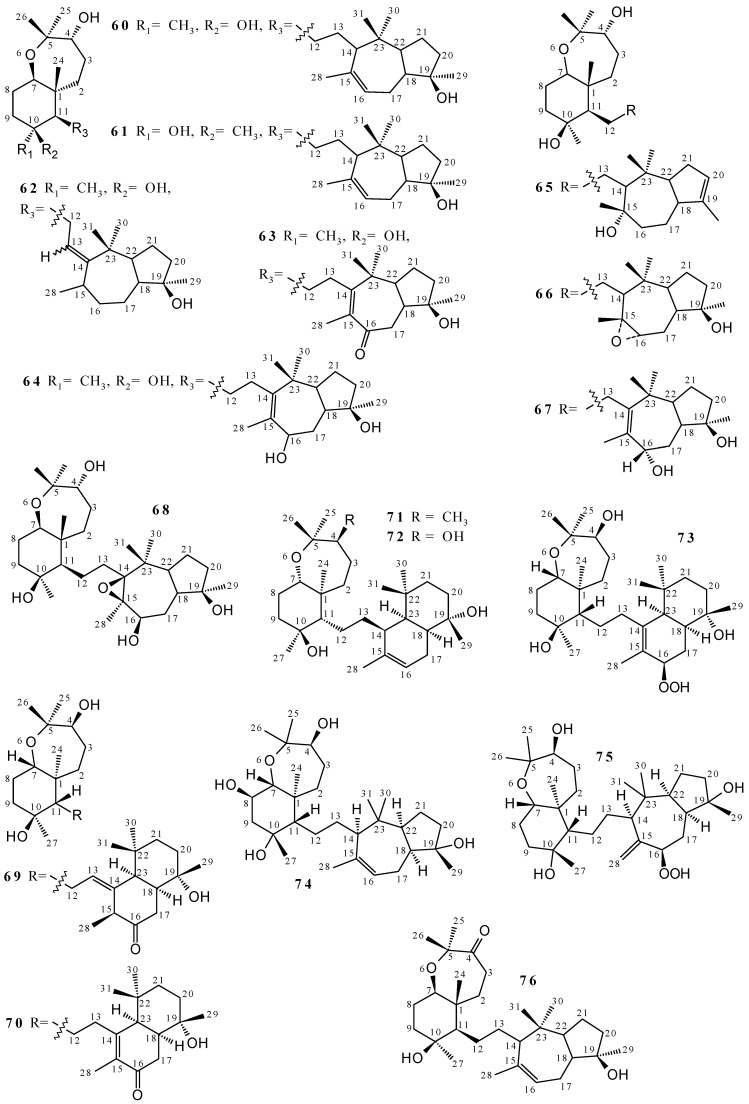

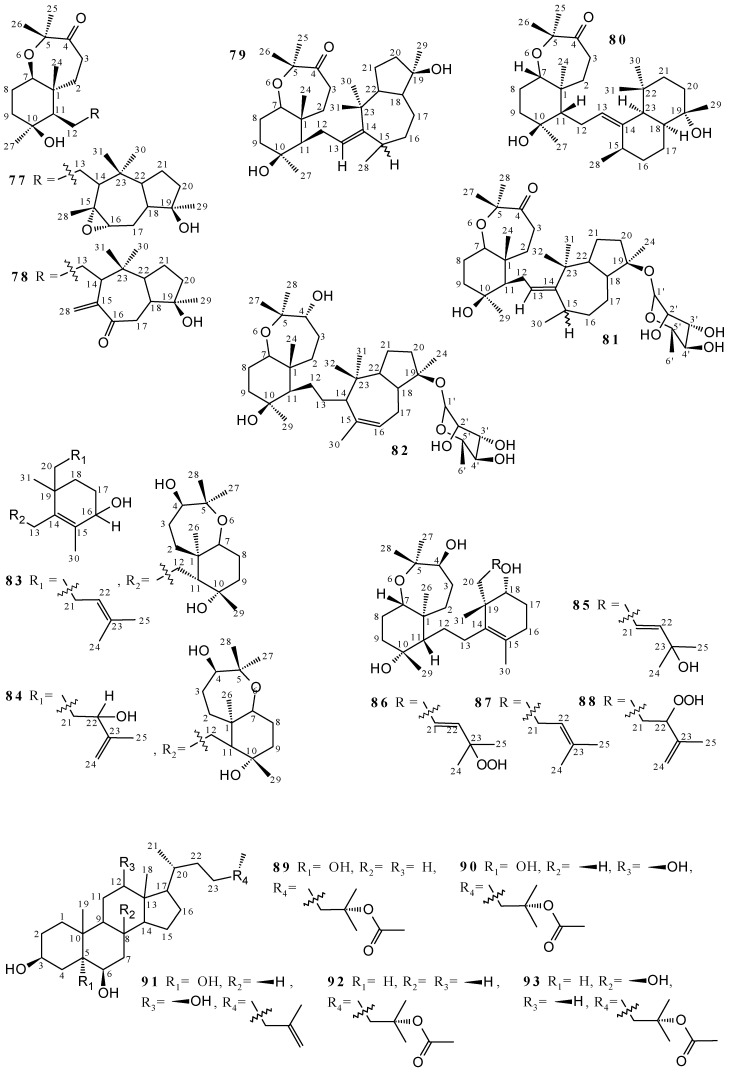

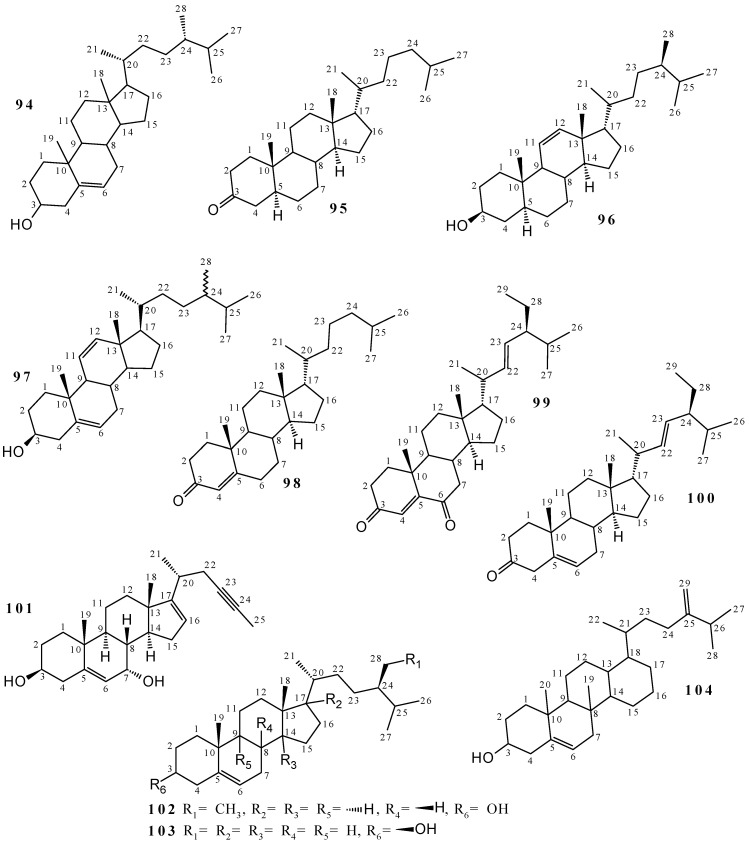
Structures of terpenoids and steroids from *Callyspongia* species.

The diterpenes callyspinol (**48**) and isocopalanol (**49**) were isolated, respectively, from *Callyspongia spinosissima* (MeOH extract) [49] and *Callyspongia sp.* (acetone extract) [50]. Compounds **48** and **49** were elucidated by ^1^H and ^13^C NMR and are structurally different (Figure 2 and Table S2): **48** has only one ring and a double bond, and is monooxygenated, while **49** has a three-membered ring and is saturated and polyoxygenated. Four *Callyspongia* sp. triterpenes were also isolated: akaterpin (**50**) from an acetone extract [51] and ilhabelanol (**51**), ilhabrene (**52**), and isoakaterpin (**53**) from an extraction with EtOH followed by MeOH [11]. The molecules **50**–**53** (Figure 2 and Table S2) were characterized by ^1^H and ^13^C NMR and they are oxygenated, sulfated, and formed by cyclic and aromatic units.

A total of 38 sipholane triterpenoids were isolated from *Callyspongia sipholena* (*Siphonochalina Siphonela*): (2*S*,4a*S*,5*S*,6*R*,8a*S*)-5-(2-((1*S*,3a*S*,5*R*,8a*S*,*Z*)-1-hydroxy-1,4,4,6-tetramethyl-1,2,3,3a,4,5,8,8a-octahydroazulen-5-yl)-ethyl)-4a,6-dimethyloctahydro-2H-chromene-2,6-diol (**54**) [52]; dahabinone A (**55**) [53]; neviotives A (**56**) [54,55,56,57], B (**57**) [53], C (**58**) [55], and D (**59**) [57]; sipholenols A (**60**) [7,8,25,55,56,57,58,59,60,61], B (**61**) [61], C (**62**) [61], D (**63**) [61], E (**64**) [61], F (**65**) [53], G (**66**) [53], H (**67**) [53], I (**68**) [59], J (**69**) [52], K (**70**) [52], L (**71**) [55], L (**72**) [8,52,56], M (**73**) [52], N (**74**) [57], and O (**75**) [57]; sipholenones A (**76**) [7,8,25,55,56,58,59,60,61], B (**77**) [61], C (**78**) [61], D (**79**) [53], and E (**80**) [52]; sipholenosides A (**81**) [53] and B (**82**) [53]; siphonellinol (**83**) [62] and siphonellinols B (**84**) [53], C (**85**) [59], C-23-hydroperoxide (**86**) [52], D (**87**) [52,57], and E (**88**) [52]. The extracts studied were: EtOAc (**54**, **60**, **69**, **70**, **72**, **73**, **76**, **80**, and **86**–**88**), EtOAc/MeOH 1:1 (**55**, **57**, **65**–**67**, **79**, **81**–**82**, and **84**), petroleum ether (**60**–**64**, **76**–**78**, and **83**), chloroform (**56**), CH_2_Cl_2_/MeOH 1:1 (**56**, **58**, **60**, **71**, **72**, and **76**), MeOH (**60**, **68**, **76**, and **85**), EtOH (**56**, **59**, **60**, **74**–**76**, and **87**) and EtOH 70% (**56**, **60**, **72**, and **76**) extracts. Molecules **63** and **67** present elucidating ^1^H NMR data, and the other metabolites are fully characterized by both ^1^H and ^13^C NMR. Sipholane triterpenoids have distinct structures (Figure 2 and Table S2), which are composed of monocyclic and polycyclic rings, unsaturation, epoxy oxygen, ether, alcohol, and carbonyls.

Fifteen sterols were isolated from *Callyspongia* species: 24*S*-24-methyl-cholestane-3*β*,5*α*,6*β*,25-tetraol-25-mono acetate (**89**), 24*S*-24-methyl chelestane-3*β*,5*α*,6*β*,12*β*,25-pentaol-25-*O*-acetate (**90**), 24*S*-24-methyl cholest-25-ene-3*β*,5*α*,6*β*,12*β*-tetrol (**91**), 24*S*-24-methyl cholestane-3*β*,6*β*,25-triol-25-*O*-acetate (**92**), 24S-24-methyl cholestane-3*β*,6*β*,8*β*,25-tetraol-25-*O*-acetate (**93**) and 24*S*-24-methylcholesterol (**94**), *5*α-cholestanone (**95**), callysterol (**96** and **97**) or ergosta-5,11-dien-3*β*-ol (**97**), cholestenone (**98**), Stigmasta-4,22-dien-3,6-dione (**99**), stigmasterone (**100**), gelliusterol E (**101**), *β*-sitosterol (**102**), siphonocholin (**103**), and ergosta-5,24(28)-dien-3*β*-ol (**104**). The obtainment of these metabolites is associated with the following extracts: **89**–**94** to MeOH extract from *Callyspongia fibrosa* [23]; **95**, **96** [7], **98**–**100** [7], and **103** [63,64] to EtOH extract from *Callyspongia siphonella*; **97** [19] and **104** [8] to MeOH/CH_2_Cl_2_ 1:1 extract from *Callyspongia siphonella* and, **101**, and **102** to MeOH/CH_2_Cl_2_ 1:1 extract from *Callyspongia implexa* [28]. Compounds **89**–**94**, **97**, and **101** were elucidated by ^1^H and ^13^C NMR, while remaining compounds of this set do not present NMR data, but are compared with information from other studies. These compounds are four-ring sterols (Figure 2 and Table S2), with **89**–**103** being formed by three six-membered rings and one of five, while in **104** a four six-membered ring system is present.

### 2.3. Alkaloids

**Figure 3:**
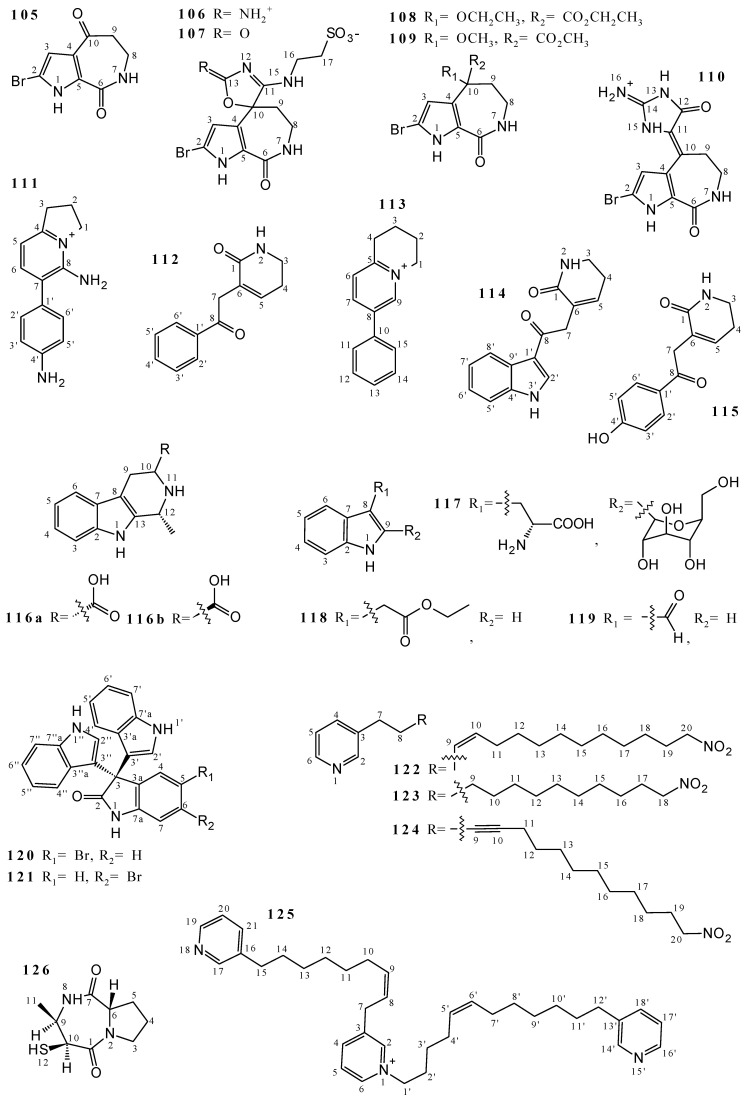

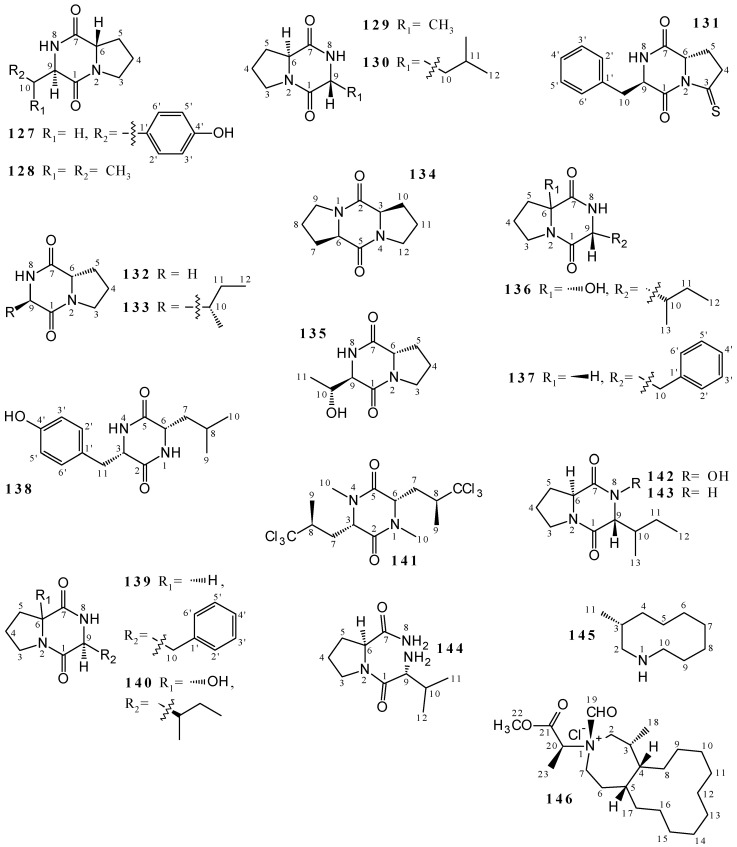
Structures of alkaloids isolated from *Callyspongia* species.

Several alkaloids were isolated and properly characterized from *Callyspongia* species. The bromopyrrole alkaloids 2-bromoaldisine (**105**), callyspongisines A (**106**), B (**107**), C (**108**), and D (**109**) and hymenialdisine (**110**) were obtained from the hydroalcoholic extract from *Callyspongia* sp. [65]. The bicyclic structures of compounds **105**–**110** were elucidated by ^1^H and ^13^C NMR and are formed by a seven-membered cyclic amide and a pyrrole attached to a bromine atom (Figure 3 and Table S3).

Some alkaloids were obtained from EtOH 95% extract of *Callyspongia* sp.: callyimine A (**111**) [18], callylactam A (**112**) [18], clathryimine B (**113**) [18], 3-(2-(1*H*-indol-3-yl)-2-oxoethyl)-5,6-dihydropyridin-2(1*H*)-one (**114**) [18], 3-(2-(4-hydroxyphenyl)-2-oxoethyl)-5,6-dihydropyridin-2(1*H*)-one (**115**) [18], (1*R*,3*R*)-1-methyl-2,3,4,9-tetrahydro-1*H*-pyrido[3,4-b]indole-3-carboxylic acid (**116a**) [66], (1*R*,3*S*)-1-methyl-2,3,4,9-tetrahydro-1*H*-pyrido[3,4-b]indole-3-carboxylic acid (**116b**) [66], C^2^-*α*-*D*-mannosylpyranosyl-tryptophan (**117**) [66], Ethyl 2-(1*H*-indol-3-yl) acetate (**118**) [67], and the indol derivative 1*H*-indole-3-carbaldehyde (**119**) [67] (Figure 3 and Table S3). Molecules **111** and **113** are structurally similar due to the presence of aromatic rings and nitrogen as a heteroatom, while **112** and **115** are only differentiated by the presence of a hydroxyl group in **115**; and the structures **114** and **116a**-**119** are formed by an indol heterocycle. Metabolites **111**–**119** not present NMR data, but compare with information from others studies.

The isomers 5-bromo trisindoline (**120**) and 6-bromo trisindoline (**121**) were isolated from the ethanolic extract of *Callyspongia siphonella* [7], and they are differentiated by the position of bromine in the aromatic ring of the indole unit of the molecules. In addition, from *Callyspongia* sp. were isolated the untenines A (**122**), B (**123**), and C (**124**), from the methanolic extract [68], and niphatoxin C (**125**), from the mixture of CH_2_Cl_2_/MeOH 4:1 and MeOH extracts [69]. The **122**–**125** structures have the pyridine group in the molecule. Metabolites **120**–**125** (Figure 3 and Table S3) were determined by ^1^H and ^13^C NMR.

Studies of some sponges *Callyspongia* sp. resulted in the isolation of Callysponine (**126**), cyclo-(*S*-Pro-*R*-Tyr) (**127**), cyclo-(*S*-Pro-*R*-Val) (**128**), cyclo-(*S*-Pro-*R*-Ala) (**129**), cyclo-(*S*-Pro-*R*-Leu) (**130**), callysponine A (**131**), cyclo-(Gly-Pro) (**132**), cyclo-(Ile-Pro) (**133**), cyclo-(Pro-Pro) (**134**), cyclo-(Thr-Pro) (**135**), cyclo-(*R*-Pro-6-hydroxyl-*R*-Ile) (**136**), cyclo-(*R*-Pro-*R*-Phe) (**137**), cyclo-(*R*-Tyr-*R*-Phe) (**138**), cyclo-(*S*-Pro-*S*-Phe) (**139**), Staphyloamide A (**140**), dysamide A (**141**), callyspongidipeptide A (**142**), cyclo-((*S*)-Pro-(*R*)-Ile) (**143**), seco-((*S*)-Pro-(*R*)-Val) (**144**), (3*R*)-methylazacyclodecane (**145**), and callyazepin (**146**) (Figure 3 and Table S3). The analyzed metabolites were obtained from the following extracts: EtOH for **126–130** [70] and **141** [6], EtOH 95% for **129** and **130** [66,71], **136**–**140** [66] and **142**–**144** [71], EtOH/H_2_O 9:1 for **131**–**135** [72,73,74,75,76,77,78,79], and MeOH + CH_2_Cl_2_ for **145** and **146** [5]. Only **126**, **130**, **131**, **136**, **141**, **142**, and **144**–**146** present ^1^H and ^13^C NMR data. The structures of **138**, **141**, **144**, and **145** are monocyclic, while **126**–**137**, **139**, **140**, **142**, **143**, and **146** are bicyclic.

### 2.4. Simple Phenols and Phenylpropanoids

**Figure 4:**
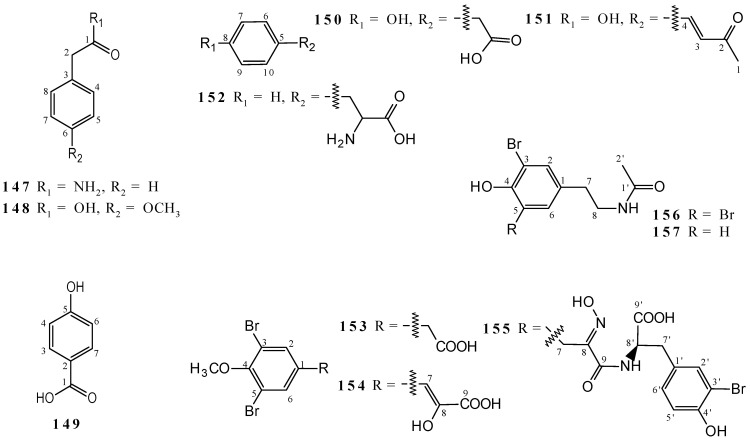
Structures of simple phenols and phenylpropanoids isolated from *Callyspongia* species.

2-Phenylacetamide (**147**) and *ρ*-methoxyphenylacetic acid (**148**) were isolated from the 95% ethanolic extract of *Callyspongia* sp. [67] and 4-hydroxybenzoic acid (**149**) from the mixture of 95% MeOH/CH_2_Cl_2_ 1:1 and EtOH extracts of *Callyspongia fibrosa* [45]. The metabolites **147**–**149** were elucidated by ^1^H NMR, but only **1** by ^13^C NMR (Table S4). All benzenoids have a substituted aromatic monocyclic structure (Figure 4).

Other metabolites were isolated from *Callyspongia*’s species: 4-hydroxyphenylacetic acid (**150**), (*E*)-4-(4-hydroxyphenyl)-3-buten-2-one (**151**), phenylalanine (**152**), 3,5-dibromo-4-methoxyphenylacetic acid (**153**), 3,5-dibromo-4-methoxyphenylpyruvic acid (**154**), callyspongic acid (**155**), *N*-acetyl-3,5-dibromo-4-hydroxyl phenylethamine (**156**), and *N*-acetyl-3-bromo-4-hydroxyphenylethamine (**157**). The metabolites **150**–**152** were obtained from 95% hydroalcoholic extracts [67] and **153**–**157** from combination of extracts MeOH/CH_2_Cl_2_ [80], all from *Callyspongia* sp. The metabolites were elucidated by ^1^H and ^13^C NMR; however, only **151**, **153**–**155**, and **157**, present the spectroscopic data. The compounds **150** and **151** are phenol derivatives, **152** is an amino acid, and **153**–**157** are bromotyrosine derivatives (Figure 4 and Table S4).

### 2.5. Nucleosides

**Figure 5:**
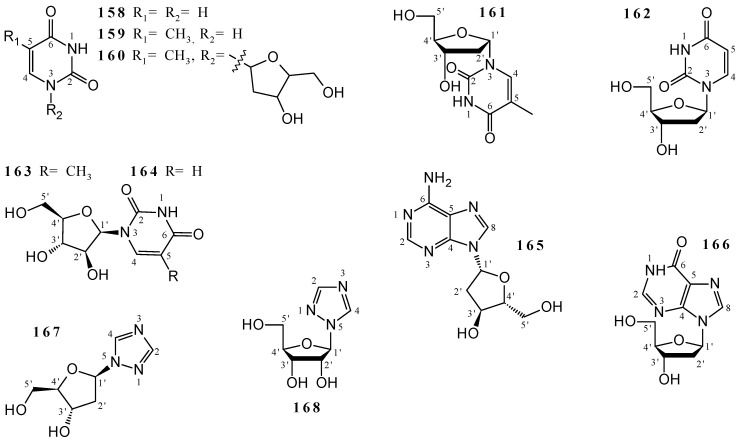
Structures of nucleosides isolated from *Callyspongia* species.

A total of 11 nucleosides was obtained from *Callyspongia* species (Figure 5 and Table S5): the diazines ^1^*H*-pyrimidine-2,4-dione (**158**) and 5-methylpyrimidine-2,4 (^1^*H*, ^3^*H*)-dione (**159**), the pyrimidine nucleosides 1-(4-hydroxy-5-hydroxymethyl-tetrahydro-furan-2-yl)-5-methyl-^1^*H*-pyrimidine-2,4-dione (**160**), 1-(2’-deoxy-*α*-*D*-ribofuranosyl)thymine (**161**), 2’-deoxyuridine (**162**), spongothymidine (**163**) and spongouridine (**164**), the purine nucleosides 2’-deoxyadenosine (**165**) and 2’-deoxyinosine (**166**), and the triazole ribonucleosides 1-(2’-deoxy-*β*-*D*-erythro-pentofuranosyl)-1*H*-1,2,4-triazole (**167**) and 1-(*β*-*D*-ribofuranosyl)-1*H*-1,2,4-triazole (**168**). The metabolites **158**–**160** were isolated from the mixture of EtOH 95% and CH_2_Cl_2_/MeOH extracts of *Callyspongia fibrosa* [45], while **161**–**168** were isolated from EtOH 90% extract of *Callyspongia* sp. [20]. The structures were elucidated based on ^1^H and ^13^C NMR data. Nucleosides **158**–**164** were characterized by the presence of pyrimidine (or 1,3-diazine) units, while **165** and **166** contain purine units in their structures, and **167**, **168** were characterized as 1,2,4-triazole derivatives.

### 2.6. Cyclic Peptides and Cyclic Depsipeptides

**Figure 6:**
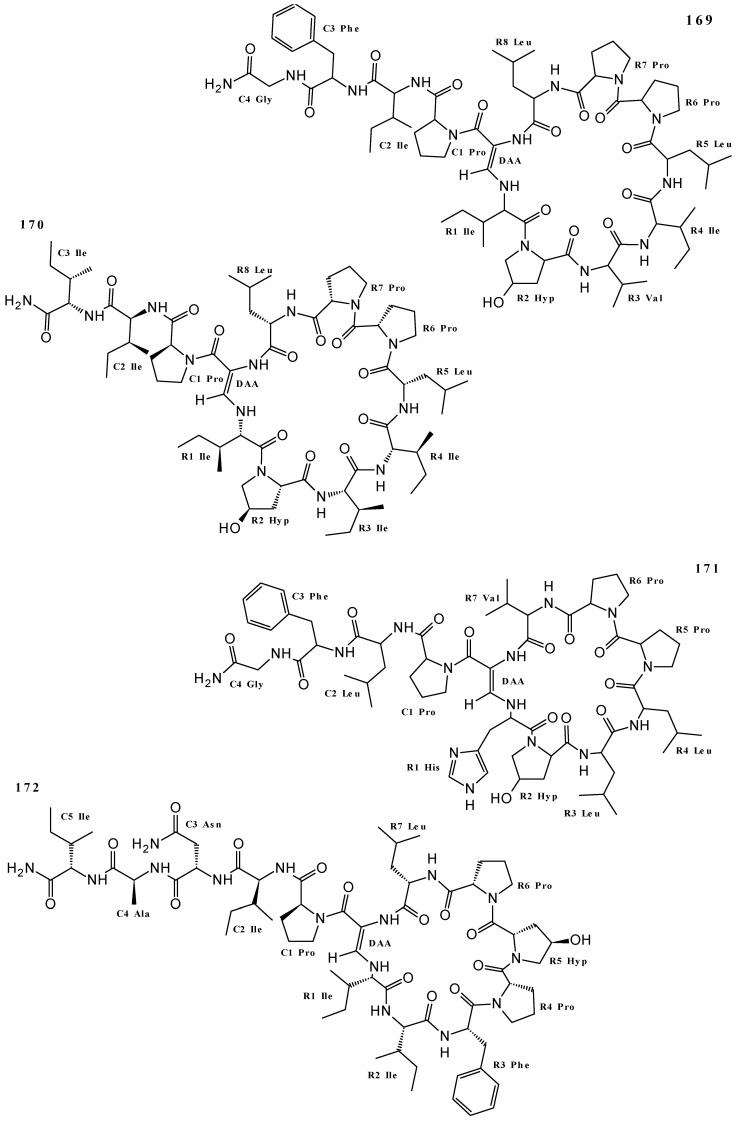

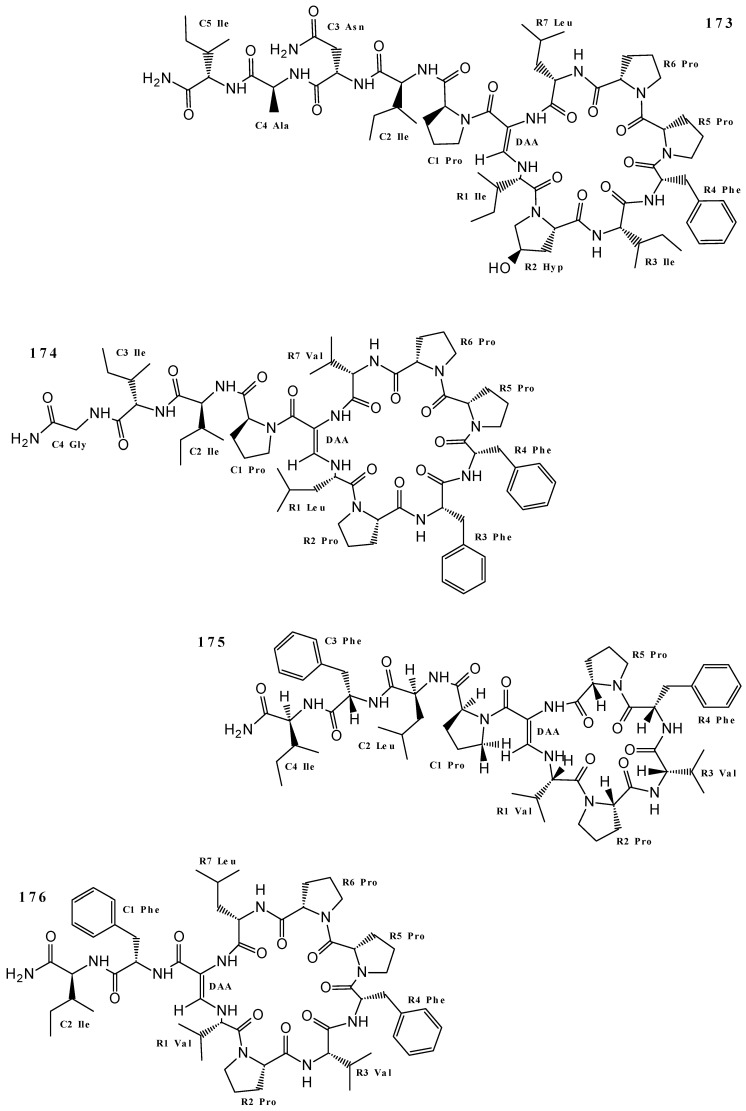

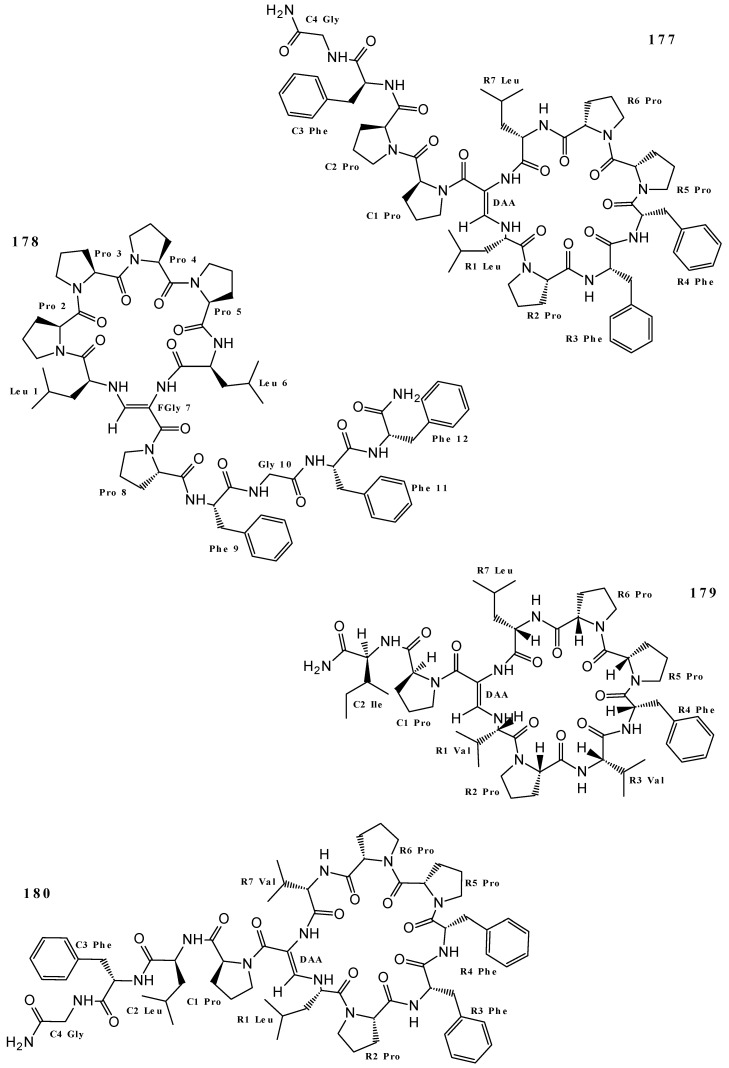

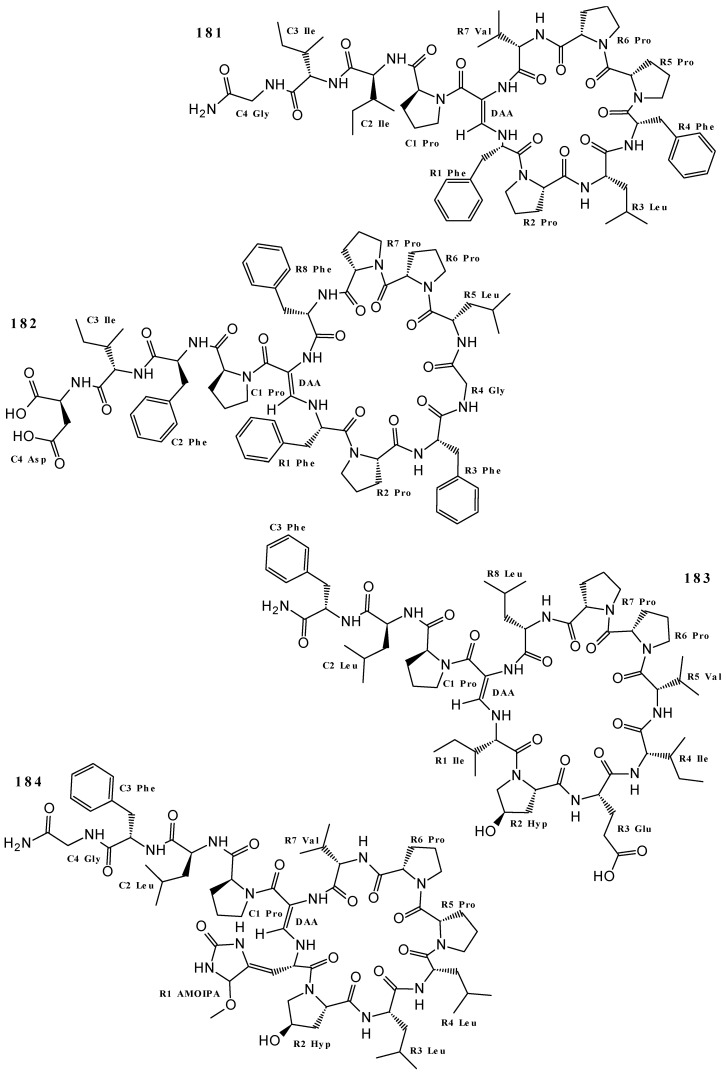

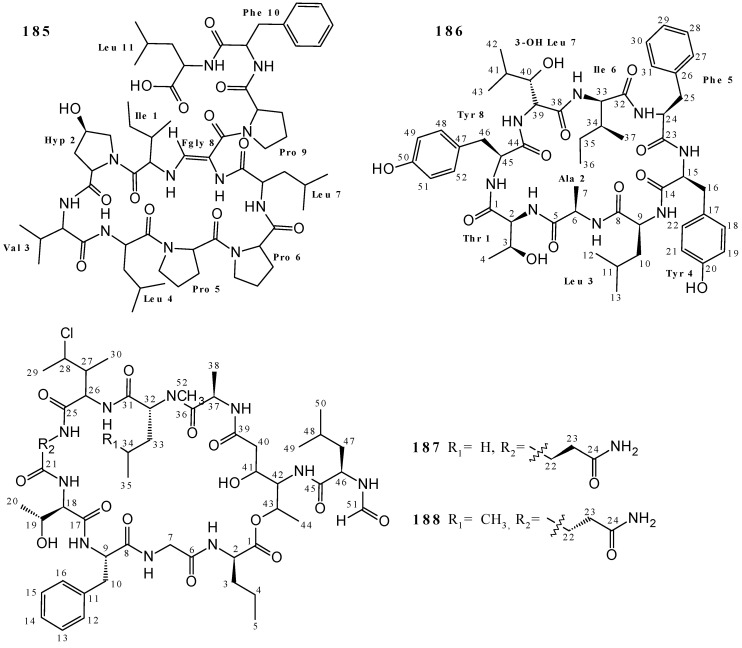
Structures of cyclic peptides and cyclic depsipeptides isolated from *Callyspongia* species.

The structures of a series of 16 Callyaerins were elucidated by ^1^H and ^13^C NMR in research exploring *Callyspongia aerizusa*: callyaerins A (**169**), B (**170**), C (**171**), D (**172** and **173**), E (**174**), F (**175** and **176**), G (**177** and **178**), H (**179**), I (**180**), J (**181**), K (**182**), L (**183**), and M (**184**). Compounds **169**–**172**, **174**, **175**, and **179** were isolated from EtOAc extract [4], and **169**–**171**, **173**, **174**, and **176**–**178** as well as **180**–**184** were obtained from MeOH extract [22,81,82]. Cyclic peptides **169**–**184** (Figure 6 and Table S6) have long chains, and for the callyaerins D (**172** and **173**), F (**175** and **176**) and G (**177** and **178**), more than one structure has been associated with the same metabolite name. In addition, callynormine A (**185**) was isolated from *Callyspongia abnormis* [83] (but no information was found on the extract used), callyptide A (**186**) from CH_2_Cl_2_/MeOH 1:1 extract of *Callyspongia* sp. [84], and the phoriospongins A (**187**) and B (**188**) were isolated from the EtOH extract of *Callyspongia bilamellata* [85]. Structures **185–188** are characteristic of cyclic peptides, and **187**–**188** are cyclic depsipeptides (Figure 6 and Table S6).

### 2.7. Polyketides

**Figure 7:**
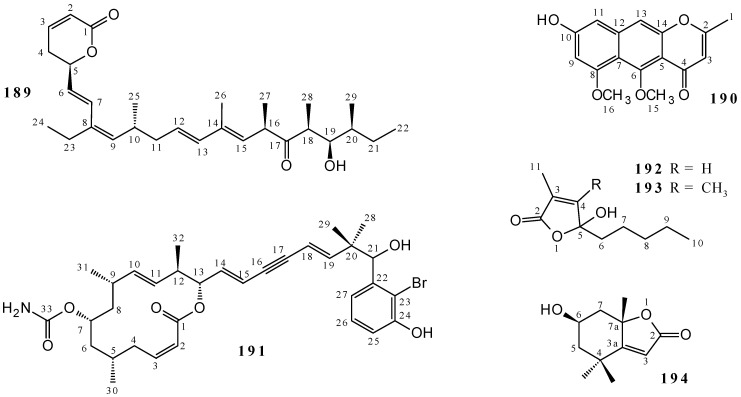
Structures of polyketides isolated from *Callyspongia* species.

Callystatin A (**189**) were characterized from the acetone extract of *Callyspongia truncata* [86,87], comantherin (**190**) from the mixture of MeOH/CH_2_Cl_2_ (1:1) and MeOH extracts of *Callyspongia* sp. [80], and callyspongiolide (**191**) from MeOH extract of *Callyspongia* sp. [88,89,90]. Compounds **189** and **190**, despite being structurally different, have common characteristics, such as the presence of dihydropyranone cycle derivatives and unsaturated bonds, as well as carbonyl, hydroxyl, and heteroatom units (Figure 7 and Table S7). In addition, butenolide 5-hydroxy-3-methyl-5-pentyl-2,5-dihydrofuran-2-one (**192**) was isolated from the acetone extract of *Callyspongia vaginalis* [9], and furans hydroxydihydrobovolide (**193**) as well as (−)-Loliolide (**194**) from the EtOH 95% extract of *Callyspongia* sp. [67]. Structures **192**–**194** were proposed as furanone derivatives (Figure 7 and Table S7). The elucidation of these compounds was performed by NMR; however, only **189**, **191**, and **192** present the data of ^1^H and ^13^C NMR.

### 2.8. Miscellanous

**Figure 8:**
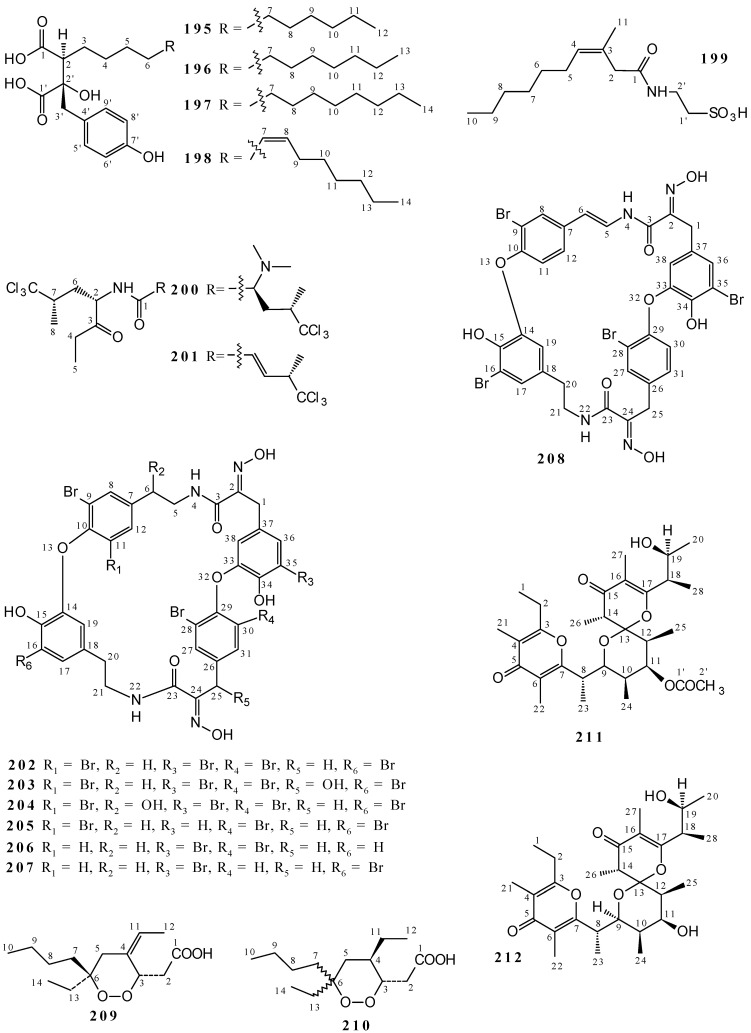
Structures of miscellaneous compounds isolated from *Callyspongia* species.

Callyspongidic acids C12:0 (**195**), C13:0 (**196**), C14:0 (**197**), and C14:1 (**198**) were isolated from MeOH/CH_2_Cl_2_ 1:1 extract from *Callyspongia californica* and characterized as phenol derivatives bearing carbonyl and hydroxyl groups (Figure 8 and Table S8) [12].

Other compounds were isolated from species of the genus *Callyspongia*: 2-(3-methyl-dec-3-enamido)ethanesulfonic acid (**199**); the Callyspongiamides A (**200**) and B (**201**); the bastadins 6 (**202**), 7 (**203**), 8 (**204**), 9 (**205**), 16 (**206**), 18 (**207**) and 24 (**208**); [(3*S*,4*Z*,6*S*)-6-butyl-6-ethyl-4-ethylidene-1,2-dioxan-3-yl]acetic acid (**209**); [(3*S*,4*R*)-6-butyl-4,6-diethyl-1,2dioxan-3-yl]acetic acid (**210**); and the callypyrones A (**211**) and B (**212**). Except for substances **211** and **212** that were isolated from an EtOAc/MeOH 1:1 of *Callyspongia diffusa* [26], these metabolites were obtained from ethanolic extract of *Callyspongia* sp. (**200** and **201**) [6], as well as 90% (**199**) hydroalcoholic [91] extracts. Also, the combination of extracts MeOH + CHCl_3_/MeOH provided **209**–**210** [92,93,94,95] while MeOH + CH_2_Cl_2_ afforded **202**–**208** [80]. The metabolites were elucidated by ^1^H and ^13^C NMR; however, only **195**–**201**, and **209**–**212** present the spectroscopic data. The structures of **199**–**212** are varied (Figure 8 and Table S8), but some of the metabolites can be grouped by structural similarity: polychlorine-containing modified dipeptides **200** and **201**, bastadins **202**–**208**, cyclic peroxides **209**–**210**, and the callypyrones **211**–**212**.

## 3. Biological Aspects of Metabolites Isolated in *Callyspongia* species

The biological activities of metabolites **1**–**212** were investigated by considering any research involving these substances, including the articles about *Callyspongia* species. In this sense, 108 compounds (including isomers **16a**,**b** and **116a**,**b**) have been associated with some type of biological action, including anti-hiv, antimalarial, antioxidant, antihypertensive, anti-angiogenic, anti-tuberculosis, antimicrobial, antiproliferative, antifouling, modulatory, inhibitory (enzyme), and cytotoxic, for example. This information is also complemented in Table 1, and discussed in the topics below. 

### 3.1. Polyacetylenes

The aikupikanynes E (**5**) and F (**6**) from *Callyspongia* sp. showed moderate activity (with IC_50_ values of 5 and 10 μg/mL) against the cancer cell lines studied (Table 1) [27]. Other polyacetylenes obtained from *Callyspongia truncata* showed a potent metamorphosis-inducing activity in the ascidian *Halocynthia roretzi* larvae (with ED_100_ values of 0.13–1.3 μg/mL) for **9**, **11**, **15**, and **32**–**38**, and antifouling activity against the barnacle *Balanus amphitrite* larvae (with ED_50_ values of 0.24–4.5 μg/mL) for **15** and **32**–**38** [29]. In addition, the inhibitory effect of the fertilization of starfish gametes of **32** and **33** in concentrations of 6.3 and 50 μM, respectively, [42].

Three polyacetylene diols were isolated from *Callyspongia* sp. and have driving Th1 polarization and antiproliferative effect against HL-60 (IC_50_ values: 6.5 μg/mL for **13**,**14** and 2.8 μg/mL for **15**) and HCT-15 (IC_50_ values: 21 μg/mL for **13**, 22 μg/mL for **14** and 34 μg/mL for **15**) [31]. **13**, **15** and **18** exhibited strong inhibitory activity against gastric H,K-ATPase (IC_50_ 1.0 × 10^−5^ M) [32,96]. The **16a** and **16b** isomers are weakly cytotoxic, with IC_50_ values of 0.47 for **16a** natural, 1.5 (± 0.29) for **16a** synthetic, 0.11 for **16b** natural and 0.35 (± 0.13) for **16b** synthetic against TR-LE and 1.8 (± 5.0) for **16a** and 5.3 (± 1.1) for **16b** synthetics against HeLa [35]. Other activities have been attributed to siphonodiol (**15**): medium antibacterial effect against *S. aureus* (MIC 12.5 μg/mL) and *S. pyrogenes* C-203 (MIC 6.2 μg/mL), and weak antifungal activity against *T. asteroids* (MIC 25.0 μg/mL) [33,96].

The metabolites **17** and **23** from *Callyspongia siphonella* proved to be weakly cytotoxic active against HCT-116. In addition, **17** and **26** were found to be weak cytotoxic against cells of MCF-7 with IC_50_ values of 65.7 and 73.6 μM, respectively, while **23** (IC_50_: 11.7 μM) presented greater activities [36].

The compound (3*R*,4*E*,28*Z*)-hentriacont-4,28-diene-1,23,30-triyn-3-ol (**19**) has been reported to be cytotoxic against the NBT-II cell line at concentrations of 5 and 10 μg/mL [37]. The metabolites **20**–**22** and **26** are moderately cytotoxic against the P388 cell lines (IC_50_ values in μg/mL: 2.2 for **20**, **22**, and **26** and 10.0 for **21**) and HeLa (IC_50_ values in μg/mL: 4.5 for **20**, 10.0 for **21**, 3.9 for **22**, and 5.1 for **26**) [38]. Cytotoxic compounds **26**–**30** also have moderate activity against HeLa (IC_50_ values 23.9–26.5 μM), MCF-7 (IC_50_ values 54.9–69.2 μM), and A549 (IC_50_ values 58.5–63.4 μM) cell lines [40]. In vitro cytotoxicity activities of compounds **24** and **25** were evaluated and verified to fight MOLT-4 cell lines (IC_50_ values: 1.9 μM for both), K-562 (IC_50_ values 5.6–6.1 μM), and HCT 116 (IC_50_ values 5.4–7.0 μM), only **24** against T-47D (IC_50_ value: 8.9 μM) and **25** against MDA-MB-231 (IC_50_ value: 9.9 μM) [39].

Two interesting compounds were isolated from *Callyspongia truncata*, the Callysponginol sulfate A (**31**), which was found to inhibit MT1-MMP with an IC_50_ of 15.0 μg/mL [41], and Callyspongynic Acid (**44**), a α-glucosidase inhibitor with an IC_50_ of 0.25 μg/mL [44]. The glycerolipid Batyl alcohol **46** showed biofilm inhibition capacity for *Alteromona macleodii*, *Ochrobactrum pseudogrignonense*, *Vibrio harveyi*, and *Staphylococcus aureus* at 0.5 and 0.025 mg/mL [97]. The polyacetylenic amide callyspongamide A (**47**) was shown to be moderately cytotoxic against HeLa (IC_50_ of 4.1 μg/mL) [46].

### 3.2. Terpenoids and Steroids

The metabolites **60**, **72**, **76**, and **104**, from *Callyspongia siphonella*, proved to be weakly cytotoxic active against HCT-116, but **60**, **72**, and **76** were found to have moderate activity (at the respective IC_50_ values of 14.8 ± 2.33, 19.8 ± 3.78, and 95.8 ± 1.34 μM) [8]. In addition, **60** presented high cytotocix activity against cells of MCF-7 with IC_50_ values of 8.8 μM [36]. The effects on Reversing P-gp-Mediated MDR to colchicine involving the KB-3-1 cell lines were also tested (IC_50_ values in μM: 5.6 ± 0.5 for **54**, 4.8 ± 0.1 for **60**, 5.1 ± 0.3 for **72**, 4.7 ± 0.3 for **73**, 4.7 ± 0.4 for **80**, 4.2 ± 0.1 for **87** and 4.6 ± 0.6 for **88**) and KB-C2 (IC_50_ values in μM: 390 ± 40 for **54**, 140 ± 30 for **60**, 150 ± 10 for **72**, 780 ± 60 for **73**, 62 ± 11 for **80**, 180 ± 10 for **87** and 560 ± 50 for **88**) [52]. 

The isocopalanol (**49**) showed inhibition ability for the PANC-1 cell line with an IC_50_ of 0.1 μg/mL [50]. akaterpin (**50**) has been proven to inhibit PI-PLC (IC_50_ of 0.5 μg/mL) and neural sphingomyelinase (IC_50_ of 30 μg/mL) [51]. The sulfated meroterpenoids **51–53** are inhibitors of L-APRT at IC_50_ of 0.7, 0.7 and 1.05 μM, respectively, [11].

The metabolites **56**, **58**, **60**, and **71** showed activity against PC-3 (IC_50_ 7.9 ± 0.12–71.2 ± 0.34 μM) and A549 (IC_50_ 8.9 ± 0.01–87.2 ± 1.34 μM) cell lines, with compound **60** being the most active [55]. The cell lines MCF-7 (IC_50_ 3.0 ± 0.4–19.2 ± 0.6 μM) and HepG-2 (IC_50_ 2.8 ± 0.4–18.7 ± 0.9 μM) were tested for **56**, **60**, **71**, and **76**, and **76** had the most significant effect [56] (also obtained MCF-7 IC_50_ values of 1.162 for **60** and 0.9 μM for **76** [58]). In the same study, antiviral activity against HAV-10 was also weak for **56** and **71** (which also showed weak effectiveness against HSV-1) and moderate for **60** [56] (**60** is an inhibitor of P-gp too) [98]. In addition, the antimicrobial activities of **56** and **71** were measured (Table 1), in which **56** obtained the greater result (12.7 ± 0.58–17.2 ± 0.58 mm) and **71** obtained a moderate one against gram positive bacteria only (12.3 ± 0.72–14.5 ± 0.72 mm) [56]. Compounds **56** and **59** also strongly inhibit RANKL-induced osteoclastogenesis with IC_50_ values of 32.8 and 12.8 μM, respectively, [57].

Sipholenol A (**60**) and sipholenone A (**76**) exhibited antiproliferative activity against +SA mouse mammary epithelial cells. While compound **76** was found to be a potential inhibitor (IC_50_ 20–30 μM), **60** had lower activity (IC_50_ 70 μM) [58]. Substances **60** and **76**, in addition to **85**, showed Reversal effects for KB-C2 [59], and **76** had both anti-angiogenic activity in CAM assay (0.026 μM per pellet) [58] and antibacterial activity (Table 1) [56]. In another study, substances **89**–**92** were associated with moderate antimalarial activity against *Plasmodium falciparum* [23], in which **89** showed the best result. Callysterol (**97**) showed an anti-inflammatory effect [19] and cholestenone (**98**) had an anti-metastatic effect on lung adenocarcinoma [98,99]. Gelliusterol E (**101**) inhibited the formation and growth of *chlamydial trachomatis* (IC_50_ value 2.3 μM) [28], and siphonocholin (**103**) inhibited the production of violacein, being an Anti-QS and Anti-biofilm compound (Table 1) [63]. β-Sitosterol (**102**) was found to exhibit anthelminthic [100], antimutagenic (at 0.5 mg/kg inhibited the mutagenicity of tetracycline) [100], angiogenic [101], antibacterial (Table 1) [102,103,104], antifungal against *Fusarium* spp. [104], antidiabetic [102,105], analgesic [100,106], antipyretic [107], anti-inflammatory [100,106,107,108,109,110,111,112,113,114], cytotoxic (Table 1) [108,109,110,111,112,113,114], hypocholesterolemic [115], and immunomodulatory activities [116].

### 3.3. Alkaloids

Furthermore, 2-Bromoaldisine (**105**) was evaluated as a potential compound for anti-HIV action, by inhibiting type 1 of this virus with an infection vector to 1/3 at 200 nM in a 96-well plate [117]. Compound **105** also inhibited MEK-1 reasonably [118], and GSK-3 (IC_50_ > 41.2 μM), DYRK1A (IC_50_ > 41.2 μM), and CK-1 significantly (IC_50_ 1.6 μM) [119]. Hymenialdisine (**110**) was reported as inhibitor kinase, acting against CK1*δ* (IC_50_ 0.03 μM), CDK5/p25 (IC_50_ 0.16 μM), and GSK-3β (IC_50_ 0.07 μM) [65,120], as well as being also moderately cytotoxic against SW620 (IC_50_ 3.1 μM) and KB-3-1 (IC_50_ 2.0 μM) cell lines [65]. 

3-(2-(4-Hydroxyphenyl)-2-oxoethyl)-5,6-dihydropyridin-2(1*H*)-one (**115**) had an in vitro anti-allergic effect predicted by in silico computational chemistry approaches [121]. The **116a**–**116b** isomers showed antioxidant activity [122] and 1*H*-indole-3-carbaldehyde (**119**) antifungal effect against the YL185 fungus [123]. The nitroalkyl pyridine alkaloids **122**–**123** exhibited a potent anti-microfouling action with IC_100_ values of 3.0, 6.1, and 5.8 mg/cm^2^, respectively, [68]. In addition, niphatoxin C (**125**) was shown to be cytotoxic against THP-1 cells and exhibited the ability to form a permeable ion [69].

The brominated oxindole alkaloid isomers **120** and **121** exhibited the following activities with the values, respectively, grouped: potent antibacterial effect against *Staphylococcus aureus* (MIC: 8 and 4 μg/mL) and *Bacillus subtilis* (MIC: 16 and 4 μg/mL), moderate biofilm inhibitory with 49.32% and 41.76% inhibition (Table 1), moderate in vitro antitrypanosomal (13.47 and 10.27 μM), and strong cytotoxicity against HT-29 (IC_50_ 8 ± 0.8 and 12.5 ± 0.3 μM), OVCAR-3 (IC_50_ 7 ± 0.3 and 9 ± 0.6 μM), and MM.1S (IC_50_ 9 ± 0.7 and 11 ± 0.9 μM) [7].

Diketopiperazines **129** and **130** have been associated with antifouling activity against cyprid larvae of the barnacle (LC_50_ 6.0 μg/cm^2^ and 3.5 μg/cm^2^) [66], while **141** has been reported as SOAT isozymes [6]. **145** and **146** are moderately cytotoxic against K562 (IC_50_ values 3.2 and 7.4 μg/mL, respectively) and A549 cell lines (IC_50_ values 3.8 and 3.0 μg/mL, respectively) [5].

### 3.4. Simple Phenols and Phenyl Propanoids

The compound 2-phenylacetamide (**147**) presented estrogenic activities in a study involving the seeds of *Lepidium apetalum*, indicating a potential for the treatment of perimenopause syndrome [124]. It was also produced by *Actinomyces* with an inhibitory effect on the plant growth of rice, lettuce, barnyard millet, and rape [125]. 4-hydroxybenzoic acid (**149**) was identified as an antimicrobial substance from Rice Hull sensitive for the tested fungi and bacteria (Table 1), in which gram-positive bacteria were inhibited (IC_50_ values ranging from 100 to 1000 µg/mL) more efficiently than the gram-negative [126]. Other studies have shown the inhibition of the growth of *Ganoderma boninense* [127] and the hypoglycemic activity [128] from **149**. In addition, 3,5-dibromo-4-methoxyphenylpyruvic acid (**154**) is weakly active in increasing the apolipoprotein E secretion from human CCF-STTG1 cells at (40 μM) [80].

### 3.5. Nucleosides

The only nucleoside from *Callyspongia* found to be biologically a is 2′-deoxyadenosine (**165**), which inhibited the keratinocyte outgrowth [129] and is toxic to E3 embryos [130] (Table 1).

### 3.6. Cyclic Peptides and Cyclic Depsipeptides

Cyclic peptides **169**–**172**, **174**–**175**, and **178**–**179** exhibited cytotoxic activity against the L5178Y cell line, especially **174** and **179**, which were potent with the respective ED_50_ of 0.39 and 0.48 μM values, respectively, while **169**–**172**, **175**, and **178** were less active (ED_50_ 2.92 to 4.14 μM) [4,22]. Still, in the same study, antimicrobial activities against *Escherichia coli*, *Staphylococcus aureus*, *Candida albicans*, and *Bacilus subtilis* were associated with the molecules **169**, **170** and **174** (Table 1) [4].

Other bioactivities have been reported among callyaerins, including potent anti-tuberculosis for **169** [22,131] and **170** [22], and moderate cytotoxicity against THP-1 (IC_50_ 5 μM), MRC-5 (IC_50_ 2 μM), and HeLa (ED_50_ 5.4 μg/mL) cell lines for **178** [22,82]. In this sense, callyptide A (**186**) was also shown to be cytotoxic, but against MDA-MB-231; ATCC: HTB 38, A549 (ATCC: CCL-185), and HT-29 (ATCC: HTB 38) cell lines [84].

### 3.7. Polyketides

Callystatin A (**189**) are moderately cytotoxic against A2058 (IC_50_ 3.2 μM) [12] and KB (IC_50_ 0.01 ng/mL) [86,87] cell lines. Callyspongiolide (**191**) has been shown to be a potent vacuolar ATPase inhibitor (IC_50_ 10 nM) [131,132] and also has a high cytotoxicity against the L5178Y cell line (IC_50_ 320 nM), Jurkat J16 T (IC_50_ 70 nM), and Ramos B lymphocytes (IC_50_ 60 nM) [88].

Hydroxydihydrobovolide (**193**) has been reported as a type 1 anti-HIV substance (IC_50_ 122.7 μM) [67,133], significantly cytotoxic against the SH-SY5Y cell line (50 μM) [134] and inhibitor of hypocotyl growth of cress seedlings (100 μM) [135]. Compound (−)-Loliolide (**194**) has a broad spectrum of bioactivity, including antibacterial (Table 1) [136,137,138], antidepressant [138,139], antifungal (Table 1) [137,138], antimutagen [138,140], moderately antioxidant (Table 1) [138,141], germination inhibitor [138,142], repellent for ants *Atta cephalotes* [67,138] and cytotoxicity against cell line L5187Y (ED_50_: 4.7 mg/mL) [136,138].

### 3.8. Miscellanous

Callyspongidic acid C13:0 (**196**) is effective against A2058 (IC_50_ 3.2 μM) [12]. Callyspongiamides **200** and **201** inhibited the SOAT1 and SOAT2 isozymes [6]. Bastadin 6 (**202**) inhibited tumor angiogenesis by inducing selective apoptosis to endothelial cells (Table 1) [143]; compounds **205** and **206** exhibited in vitro cytostatic and/or cytotoxic effects against MCF-7 (IC_50_ 4 to 8 μM), A549 (IC_50_ 3 to 8 μM), Hs683 (IC_50_ 3 to 4 μM), U373 (IC_50_ 3 to 11 μM), B16F10 (IC_50_ 4 to 6 μM), and SKMEL 28 (IC_50_ 4 to 7 μM) cells, and only **202** and **206** against L5178Y (IC_50_ 1.5 to 1.9 μM, respectively) [144,145]. Bastadin 7 (**203**) is also cytotoxic against L5178Y, however, with IC_50_ 5.3 μM [145]; and also significantly inhibited the serum + hEGF-induced tubular formation of HUVEC (1 μg/mL) [94]. Bastadin 8 (**204**) showed moderate inhibitory activity of IMPDH [95], while bastadin 24 (**208**) had cytotoxicity against CNXF SF268, LXFA 629L, MAXF 401NL, MEXF 276L, and PRXF 22RV1 [94]. Other compounds have been proven to be cytotoxic: **209** and **210** against the P-388 cell line (ED_50_ values 5.5 and 2.6 μg/mL, respectively) [92]. Lastly, **211** and **212** exhibited antihypertensive and antioxidant activity [26].

**Table 1:** Biological aspects in active metabolites of *Callyspongia* species.

Metabolite Name	Biological Activity	Ref.
Aikupikanyne E (**5**)	Cytotoxicity {(P-388, ATCC: CCL 46), (A-549, ATCC: CL 8) and (HT-29, ATCC: HTB 38)}	[27]
Aikupikanyne F (**6**)	Cytotoxicity {(P-388, ATCC: CCL 46), (A-549, ATCC: CL 8) and (HT-29, ATCC: HTB 38)}	[27]
Callyberyne A (Callypentayne) (**9**)	Metamorphosis-inducing (Ascidian *Halocynthia roretzi* larvae)	[29]
Callyberyne C (Callytetrayne) (**11**)	Metamorphosis-inducing (Ascidian *Halocynthia roretzi* larvae)	[29]
14,15-Dihydrosiphonodiol (Dihydrosiphonodiol) (**13**)	Antiproliferative activity (HL-60 and HCT-15 cell lines)	[31]
	Inhibitory activity (gastric H,K-ATPase)	[32,96]
Callyspongidiol (**14**)	Antiproliferative activity (HL-60 and HCT-15 cell lines)	[31]
Siphonodiol (**15**)	Metamorphosis-inducing (Ascidian *Halocynthia roretzi* larvae)	[29]
	Antifouling activity (Barnacle *Balanus Amphitrite* larvae)	[29]
	Antiproliferative activity (HL-60 and HCT-15 cell lines)	[31]
	Antibacterial (*Staphylococcus aureus* and *Streptococcus pyogenes*)	[33,96]
	Antifungal (*Trichophyton asteroides*)	[33,96]
	Inhibitory activity (gastric H,K-ATPase)	[32,96]
(+)-(4*E*,16*E*)-icosa-4,16-diene-1,19-diyne-3,18-diol (**16a**)	Cytotoxic (TR-LE and HeLa cell lines)	[35]
(−)-(4*E*,16*E*)-icosa-4,16-diene-1,19-diyne-3,18-diol (**16b**)	Cytotoxic (TR-LE and HeLa cell lines)	[35]
Callyspongendiol (**17**)	Cytotoxicity (HCT-166 and MCF-7 cell lines)	[8,36]
Tetrahydrosiphonodiol (**18**)	Inhibitory activity (gastric H,K-ATPase)	[29,96]
(3*R*,4*E*,28*Z*)-Hentriacont-4,28-diene-1,23,30-triyn-3-ol (**19**)	Cytotoxicity (NBT-II cell line)	[37]
Callyspongenol A (**20**)	Cytotoxicity (P388 and HeLa cell lines)	[38]
Callyspongenol B (**21**)	Cytotoxicity (P388 and HeLa cell lines)	[38]
Callyspongenol C (**22**)	Cytotoxicity (P388 and HeLa cell lines)	[38]
Callyspongenol D (**23**)	Cytotoxicity (MCF-7 and HCT-116 cell lines)	[8,36]
Callysponyne A (**24**)	Cytotoxicity (MOLT-4, K-562, T-47D and HCT 116 cell lines)	[39]
Callysponyne B (**25**)	Cytotoxicity (MOLT-4, K-562, MDA-MB-231 and HCT 116 cell lines)	[39]
Dehydroisophonochalynol (Dehydrosiphonochalynol) (**26**)	Cytotoxicity (P388, HeLa, MCF-7 and A549 cell lines)	[36,38,40]
Siphonellanol A (**27**)	Cytotoxicity (HeLa, MCF-7 and A549 cell lines)	[40]
Siphonellanol B (**28**)	Cytotoxicity (HeLa, MCF-7 and A549 cell lines)	[40]
Siphonellanol C (**29**)	Cytotoxicity (HeLa, MCF-7 and A549 cell lines)	[40]
Siphonchalynol (**30**)	Cytotoxicity (HeLa, MCF-7 and A549 cell lines)	[40]
Callysponginol sulfate A (**31**)	Inhibitor of MT1-MMP	[41]
Callyspongin A (Siphonodiol disulfate) (**32**)	Inhibitor of fertilization of starfish gametes	[42]
	Metamorphosis-inducing (Ascidian *Halocynthia roretzi* larvae)	[29]
	Antifouling activity (Barnacle *Balanus Amphitrite* larvae)	[29]
Callyspongin B (Siphonodiol sulfate) (**33**)	Inhibitor of fertilization of starfish gametes	[42]
	Metamorphosis-inducing (Ascidian *Halocynthia roretzi* larvae)	[29]
	Antifouling activity (Barnacle *Balanus Amphitrite* larvae)	[29]
Callytriol A (**34**)	Metamorphosis-inducing (Ascidian *Halocynthia roretzi larvae*)	[29]
	Antifouling activity (Barnacle *Balanus Amphitrite* larvae)	
Callytriol B (**35**)	Metamorphosis-inducing (Ascidian *Halocynthia roretzi larvae*)	[29]
	Antifouling activity (Barnacle *Balanus Amphitrite* larvae)	
Callytriol C (**36**)	Metamorphosis-inducing (Ascidian *Halocynthia roretzi larvae*)	[29]
	Antifouling activity (Barnacle *Balanus Amphitrite* larvae)	
Callytriol D (**37**)	Metamorphosis-inducing (Ascidian *Halocynthia roretzi larvae*)	[29]
	Antifouling activity (Barnacle *Balanus Amphitrite* larvae)	
Callytriol E (**38**)	Metamorphosis-inducing (Ascidian *Halocynthia roretzi larvae*)	[29]
	Antifouling activity (Barnacle *Balanus Amphitrite* larvae)	
Callyspongynic Acid (**44**)	α-glucosidase inhibitor	[44]
Batyl alcohol (**46**)	Biofilm inhibition (*Alteromona macleodii*, *Ochrobactrum pseudogrignonense*, *Vibrio harveyi* and *Staphylococcus aureus*)	[97]
Callyspongamide A (**47**)	Cytotoxicity (HeLa cell lines)	[46]
Isocopalanol (**49**)	Cytotoxicity (PANC-1 cell line)	[50]
Akaterpin (**50**)	Enzyme Inhibitor (PI-PLC and neural sphingomyelinase)	[51]
Ilhabelanol (**51**)	Inhibitor of L-APRT	[11]
Ilhabrene (**52**)	Inhibitor of L-APRT	[11]
Isoakaterpin (**53**)	Inhibitor of L-APRT	[11]
(2*S*,4a*S*,5*S*,6*R*,8a*S*)-5-(2-((1*S*,3a*S*,5*R*,8a*S*,*Z*)-1-hydroxy-1,4,4,6-tetramethyl-1,2,3,3a,4,5,8,8a-octahydroazulen-5-yl)-ethyl)-4a,6-dimethyloctahydro-2H-chromene-2,6-diol (**54**)	Cytotoxicity (KB-3-1 and KB-C2)	[52]
Neviotine A (**56**)	Inhibitory activity (RANKL induced osteoclastogenesis)	[57]
	Cytotoxicity (PC-3, A549, MCF-7 and HepG-2 cell lines)	[55,56]
	Antibacterial activity (*Staphylococcus aureus*, *Bacillis subtilis* and *Escherichia coli*)	[56]
	Antiviral activity (HAV-10)	[56]
Neviotine C (**58**)	Cytotoxicity (PC-3 and A549 cell lines)	[55]
Neviotine D (**59**)	Inhibitory activity (RANKL induced osteoclastogenesis)	[57]
Sipholenol A (15-sipholen-4,10,19-triol) (**60**)	Cytotoxicity (KB-3-1, KB-C2, HepG-2, PC-3, A549, MCF-7 and HCT-116 cell lines)	[8,36,52,55,56,58,59]
	Inhibitor of P-gp	[98]
	Antiproliferative activity (+SA mouse mammary epithelial cells)	[58]
	Antiviral (HAV-10)	[56]
Sipholenol L (**71**)	Cytotoxicity (MCF-7 and HepG-2 cell lines)	[56]
	Antibacterial activity (*Staphylococcus aureus* and *Bacillis subtilis*)	[56]
	Antiviral (HAV-10 and HSV-1)	[56]
Sipholenol L (**72**)	Cytotoxicity (HCT-116, KB-3-1 and KB-C2 cell lines)	[8,52]
Sipholenol M (**73**)	Cytotoxicity (KB-3-1 and KB-C2 cell lines)	[52]
Sipholenone A (15-sipholen-10,19-diol-4-one) (**76**)	Cytotoxicity (HCT-116, PC-3, A549, MCF-7 and HepG-2 cell lines)	[8,55,56,58]
	Antibacterial activity (*Staphylococcus aureus*, *Bacillis subtilis* and *Escherichia coli*)	[56]
	Reversal effects for KB-C2	[59]
	Antiproliferative activity (+SA mouse mammary epithelial cells)	[58]
	Anti-angiogenic activity (CAM assay)	[58]
Sipholenone E (**80**)	Cytotoxicity (KB-3-1 and KB-C2 cell lines)	[52]
Siphonellinol C (**85**)	Reversal effects for KB-C2	[59]
Siphonellinol D (**87**)	Cytotoxicity (KB-3-1 and KB-C2 cell lines)	[52]
Siphonellinol E (**88**)	P-gp modulatory activity	[52]
24*S*-24-methyl-cholestane-3*β*,5*α*,6*β*,25-tetraol-25-mono acetate (**89**)	Antimalarial (*Plasmodium falciparum*)	[23]
24*S*-24-methyl chelestane-3*β*,5*α*,6*β*,12*β*,25-pentaol-25-*O*-acetate (**90**)	Antimalarial (*Plasmodium falciparum*)	[23]
24*S*-24-methyl cholest-25-ene-3*β*,5*α*,6*β*,12*β*-tetrol (**91**)	Antimalarial (*Plasmodium falciparum*)	[23]
24*S*-24-methyl cholestane-3*β*,6*β*,25-triol-25-*O*-acetate (**92**)	Antimalarial (*Plasmodium falciparum*)	[23]
Callysterol (ergosta-5,11-dien-3*β*-ol) (**97**)	Anti-inflammatory	[19]
Cholestenone (4-cholesten-3-one) (**98**)	Anti-metastasis of lung adenocarcinoma	[99]
Gelliusterol E (**101**)	Antichlamydial (*Chlamydia trachomatis*)	[28]
*β*-sitosterol (**102**)	Analgesic	[100,106]
	Angiogenic	[101]
	Anthelminthic	[100]
	Antibacterial (*Bacillus subtilis*, *Escherichia coli*, *Staphylococcus aureus*, *Pseudomonas aeruginosa*, *Salmonella typhii*, *Corynebacterium diphtheria* and *Klebsiella pneumoniae*)	[102,103,104]
	Antidiabetic	[102,105]
	Antifungal (*Fusarium spp.*)	[104]
	Anti-inflammatory	[100,106,107,108]
	Antimutagenic	[100]
	Antipyretic	[107]
	Cytotoxicity (MCF-7, HT-29, U937, MDA-MB-231, SGC-7901 and LNCaP)	[108,109,110,111,112,113,114]
	Hypocholesterolemic	[115]
	Immunomodulatory (pigs imune)	[116]
Siphonocholin (**103**)	Anti-QS (inhibit the production of violacein)	[63]
	Anti-biofilm (*Paracoccus* sp., *Pseudomonas aeruginosa*, *Pseudoalteromonas* sp. and *Bacillus* sp.)	[63]
Ergosta-5,24(28)-dien-3*β*-ol (**104**)	Cytotoxicity (HCT-116 cell line)	[8]
2-bromoaldisine (**105**)	Anti-HIV-1	[117]
	Inhibitory (Raf/MEK-1/MAPK cascade)	[118]
	Inhibitory (GSK-3, DYRK1A, CK-1)	[119]
Hymenialdisine (**110**)	Cytotoxicity (SW620 and KB-3-1 cell lines)	[65]
	Kinase inhibitor (CK1, CDK5 and GSK-3β)	[65,120]
3-(2-(4-hydroxyphenyl)-2-oxoethyl)-5,6-dihydropyridin-2(1*H*)-one (**115**)	Anti-allergic	[121]
(1*R*,3*R*)-1-methyl-2,3,4,9-tetrahydro-1*H*-pyrido[3,4-b]indole-3-carboxylic acid (**116a**)	Anti-oxidant	[122]
(1*R*,3*S*)-1-methyl-2,3,4,9-tetrahydro-1*H*-pyrido[3,4-b]indole-3-carboxylic acid (**116b**)	Anti-oxidant	[122]
1*H*-indole-3-carbaldehyde (**119**)	Inhibitor (Tyrosinase)	[123]
5-bromo trisindoline (**120**)	Antibacterial (*Staphylococcus aureus* and *Bacillus subtilis*)	[7]
	Biofilm inhibitory (*Pseudomonas aeruginosa*)	[7]
	Antitrypanosomal	[7]
	Cytotoxicity (HT-29, OVCAR-3 and MM.1S)	[7]
6-bromo trisindoline (**121**)	Antibacterial (*Staphylococcus aureus* and *Bacillus subtilis*)	[7]
	Biofilm inhibitory (*Pseudomonas aeruginosa*)	[7]
	Antitrypanosomal	[7]
	Cytotoxicity (HT-29, OVCAR-3 and MM.1S)	[7]
Untenine A (**122**)	Anti-microfouling	[68]
Untenine B (**123**)	Anti-microfouling	[68]
Untenine C (**124**)	Anti-microfouling	[68]
Niphatoxin C (**125**)	Cytotoxicity (THP-1 cell line)	[69]
Cyclo-(*S*-Pro-*R*-Ala) (**129**)	Antifouling (Cyprid larvae of the barnacle)	[66]
Cyclo-(*S*-Pro-*R*-Leu) (Cyclo-((*S*)-Pro-(*R*)-Leu)) (**130**)	Antifouling (Cyprid larvae of the barnacle)	[66]
Dysamide A (**141**)	Inhibitor of the SOAT1 and SOAT2 isozymes	[6]
(3*R*)-methylazacyclodecane (**145**)	Cytotoxic (K562 and A549 cell lines)	[5]
Callyazepin (**146**)	Cytotoxic (K562 and A549 cell lines)	[5]
2-phenylacetamide (**147**)	Estrogenic activities	[124]
	Inhibitory effect to the growth (rice, lettuce, barnyard millet and rape)	[125]
4-hydroxybenzoic acid (**149**)	Antimicrobial Activity (*Staphylococcus aureus*, *Staphylococcus epidermidis*, *Bacillus subtilis*, *Lactobacillus plantarum*, *Leuconostoc mesenteroides*, *Escherichia coli*, *Salmonella typhimurium*, *Pseudomonas aeruginosa*, *Pseudomonas*. *Syringae*, *Pseudomonas*. *syringae* pv. *Tobaci*, *Ewinia carotovora* subsp. *carotovora*, *Xanthomonas campestri* and *Agrobacterium*)	[126]
	Fungitoxicity (inhibited the growth of *Ganoderma boninense*)	[127]
	Hypoglycemic activity	[128]
3,5-dibromo-4-methoxyphenylpyruvic acid (**154**)	ApoE modulatory (CCF-STTG1 cell line)	[80]
2’-Deoxyadenosine (**165**)	Inhibitor of keratinocyte proliferation	[129]
	Toxic to E3 embryos	[130]
Callyaerin A (**169**)	Anti-Tuberculosis	[22,131]
	Antibacterial (*Escherichia coli* and *Staphylococcus aureus*)	[4]
	Antifungal (*Candida albicans*)	[4]
	Cytotoxicity (L5178Y cell line)	[4]
Callyaerin B (**170**)	Anti-Tuberculosis	[22]
	Antibacterial (*Escherichia coli* and *Staphylococcus aureus*)	[4]
	Antifungal (*Candida albicans*)	[4]
	Cytotoxicity (L5178Y, THP-1 and MRC-5 cell lines)	[4,22]
Callyaerin C (**171**)	Cytotoxicity (L5178Y cell line)	[4]
Callyaerin D (**172**)	Cytotoxicity (L5178Y cell line)	[4]
Callyaerin E (**174**)	Cytotoxicity (L5178Y cell line)	[4]
	Antimicrobial (*Escherichia coli*, *Staphylococcus aureus Candida albicans* and *Bacilus subtilis*)	[4]
Callyaerin F (**175**)	Cytotoxicity (L5178Y cell line)	[4]
Callyaerin G (**178**)	Cytotoxicity (L5178Y and HeLa cell lines)	[4,82]
Callyaerin H (**179**)	Cytotoxicity (L5178Y cell line)	[4]
Callyptide A (**186**)	Cytotoxicity {MDA-MB-231; ATCC: HTB 38, A549 (ATCC: CCL-185) and HT-29 (ATCC: HTB 38) cell lines}	[84]
Callystatin A (**189**)	Cytotoxicity (KB cell line)	[86,87]
Callyspongiolide (**191**)	Cytotoxicity (L5178Y cell line and Jurkat J16 T and Ramos B lymphocytes)	[88]
	Inhibitor (Vacuolar ATPase)	[132]
Hydroxydihydrobovolide (**193**)	Anti-HIV	[67,133]
	Cytotoxicity (SH-SY5Y cell line)	[134]
	Plant growth inhibitor	[135]
(–)-loliolide (**194**)	Antibacterial (*Bacillus subtilis*, *Neisseria gonorrhoeae*, *Pseudomonas aeruginosa*, *Escherichia coli*, *Staphylococcus aureus*, *Staphylococcus epidermidis*, *Enterobacter cloacae* and *Klebsiella pneumoniae*)	[136,137,138]
	Antidepressant	[138,139]
	Antifungal (*Candida albicans* and *Aspergillus niger*)	[137,138]
	Antimutagen	[138,140]
	Antioxidant (DPPH, H_2_O_2_ radicals and intercellular ROS)	[138,141]
	Cytotoxicity (L5187Y cell line)	[136,138]
	Germination inhibitor (lettuce and alfalfa seeds)	[138,142]
	Repellent for ants (Atta cephalotes)	[67,138]
Callyspongidic acid C13:0 (**196**)	Cytotoxicity (A2058 cell line)	[12]
Callyspongiamide A (**200**)	Inhibitors of the SOAT1 and SOAT2 isozymes	[6]
Callyspongiamide B (**201**)	Inhibitors of the SOAT1 and SOAT2 isozymes	[6]
Bastadin 6 (**202**)	Anti-angiogenic activity (inhibit VEGF and bFGF of HUVECs)	[143]
	Cytostatic and/or cytotoxic effects (L5178Y, MCF-7, A549, Hs683, U373, B16F10 and SKMEL 28)	[144,145]
Bastadin 7 (**203**)	Cytotoxicity (L5178Y)	[145]
	Inhibitor (the serum + hEGF-induced tubular formation of HUVEC)	[94]
Bastadin 8 (**204**)	Inhibitor (IMPDH)	[95]
Bastadin 9 (**205**)	Cytostatic and/or cytotoxic effects (MCF-7, A549, Hs683, U373, B16F10 and SKMEL 28)	[144]
Bastadin 16 (**206**)	Cytostatic and/or cytotoxic effects (L5178Y, MCF-7, A549, Hs683, U373, B16F10 and SKMEL 28)	[144,145]
Bastadin 24 (**208**)	Cytotoxicity (CNXF SF268, LXFA 629L, MAXF 401NL, MEXF 276L and PRXF 22RV1)	[94]
[(3*S*,4*Z*,6*S*)-6-butyl-6-ethyl-4-ethylidene-1,2-dioxan-3-yl]acetic acid (**209**)	Cytotoxicity (P-388 cell line)	[92]
[(3*S*,4*R*)-6-butyl-4,6-diethyl-1,2dioxan-3-yl]acetic acid (**210)**	Cytotoxicity (P-388 cell line)	[92]
Callypyrone A (**211**)	Antihypertensive	[26]
	Antioxidant	[26]
Callypyrone B (**212**)	Antihypertensive	[26]
	Antioxidant	[26]

## 4. Discussion

The genus *Callyspongia* is composed of various species of sponges, in which 261 have been described and approximately 180 accepted by reviews of taxonomists [3,4]. Although only 15 species were identified in this review, these metabolites were isolated and properly characterized by NMR. *Callyspongia* sp. species were also considered in the bibliographic survey, but their non-identification makes the distinction between them impossible, allowing only a speculative approach based on localities of origin of these sponges. However, these results suggest that there are still many *Callyspongia* sponges that can be studied.

The first study about the isolation of metabolites from *Callyspongia* was published in 1981 [25] and the most recent ones have been published in 2020 [26,63]. Analyzing this time range, the expansion in the rate of publications is notable, especially if publications of the last decade are taken into account, indicating the increased interest in researching *Callyspongia* species. Still, during this period, two species of Shiphonochalina have been taxonomically reclassified and are currently known as *Callyspongia lindgreni* (*Siphonochalina truncata*) [32,33] and *Callyspongia siphonella* (*Siphonochalina siphonela*) [25,36,40,53,54,55,56,57,60,61,62,63]. 

In total, 212 metabolites were identified from *Callyspongia*, in which 103 are categorized in two classes, polyacetylenes (**1**–**47**), and terpenoids and steroids (**48**–**104**), in agreement with previous studies that present substances of this class as characteristic in the genus. In this sense, because of the greater number of isolations in different species, polyacetylenes could be classified as chemical markers for *Callyspongia* [9,27].

The sipholane triterpenoids (**54**–**88**) were also extensively documented, being the first isolated metabolites according to the investigations of this review [25], but they are only associated with *Callyspongia siphonella*. In addition, most of isolated compounds were collected from sponges of Red Sea regions, China, Japan, Indonesia, and Australia. This fact highlights the potential for further research in regions where the genus is less explored, such as Brazil, Ecuador, and Barbados, for example. It is also important to note that in some studies, no trace was found on the place of origin of the marine material studied [20,33,51,87].

Molecules **1**–**212** are structurally varied, and because of this, confusion such as the changing names of metabolites [29,42] and the attribution of different structures to the same compound can occur, for example, the Callyaerins D [4,22], F [4,22] and G [22,82]. The unavailability of ^1^H and ^13^C NMR data was also identified in some articles, but it is still possible to obtain spectroscopic information from other studies. The number of isolated compounds confirms the interest in the genus, but other investigations not covered in the review also contribute to this aspect: isolation accompanied by characterization [10], identification by dereplication [7], Mass Spectrometry [146,147] (process also present in some of the metabolites **1**–**212**), and the isolation of compounds from beings that establish symbiotic relationships with *Callyspongia* species [148,149]. Thus, it can be said that this genus has been widely explored through different types of research.

Some of the 212 metabolites reported herein were described in original reviews and articles as biologically relevant. Among these compounds, 109 molecules (including isomers **16a**–**16b** and **116a**–**116b**) have been reported as bioactive (Table 1), corresponding to approximately half of the metabolites elucidated in *Callyspongia*. The absence of biological approaches for some substances in the studies indicates a great opportunity for future research and advances in the field. In addition, polyacetylenes correspond to the largest class of bioactive metabolites in the genus, and the most frequent biological activities were cytotoxicity and antimicrobial (antibacterial and antifungal). In this sense, the results are in agreement with the data that prove the relevance of the metabolites in the genus with anticancer action [24,40,58,94,98,109,111,113,144].

Future perspectives are encouraging, with regard to the emergence of new chemical contributions to the genus *Callyspongia*. However, there are still limitations in the study of sponges, some of the most significant are: the geographical location in the collection of species, the high concentration of marine salts in samples and extracts, the high cost of carrying out the experimental procedures and the probability of isolating metabolite with low yield. Some of the patterns observed in the methodologies of the articles can be pointed out the procedures used to minimize research problems in marine beings; Because of this, the frequent collection of sponges in regions close to places with anthropogenic action and the predominance in the isolation of non-polar compounds was observed. Consequently, we believe that the exploitation of *Callyspongia* species will expand.

## 5. Materials and Methods

The literature review on the genus Callyspongia was based on the theme: “metabolites isolated from Callyspongia species and characterized by the NMR spectroscopic technique”. This systematic secondary study was adopted through the qualitative and quantitative approach to information on the topic and conducted in electronic scientific databases and in websites of the selected journals, such as as: ACS Publications, Google Scholar, PubMed, ResearchGate, SciELO, Science Direct, SciFinder, Semantic Scholar, Springer Link, Taylor & Francis Online and Wiley Online Library. The only word investigated in isolation was “Callyspongia”, but “activity”, “biological”, “biological activity”, “NMR” was also used.

The knowledge about the species existing in the genus Callyspongia was obtained through the World Marine Species Register (WoRMS). The species were classified by nomenclature and researched individually. Additional information was obtained by searching for the term “Callyspongia” accompanied by keywords specific to the articles, such as the species name, the collection site, the name of the isolated metabolites and the types of biological activity. In addition, the data of biological activities of metabolites were searches by the name of the structures accompanied by the terms “biological”, “activity” and “biological activity”.

The selection of articles proceeded using inclusion criteria, i.e., the characterization of molecules by NMR as the primary criterion and the presence of biological activity as the secondary. The articles were identified by means of a summarized reading of the published content. The investigations reached a total of 973 articles, of which, 145 were considered compatible with the inclusion criteria, and selected for the review.

**Figure 9:**
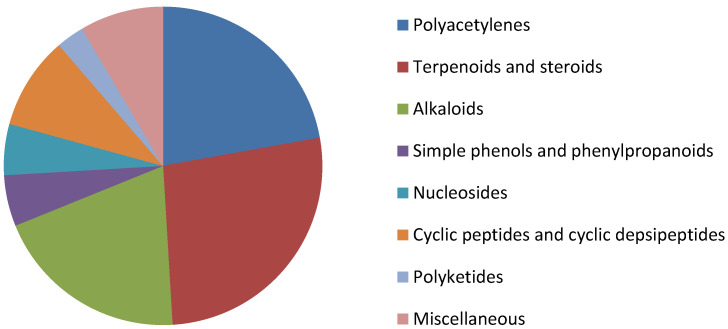
Classes of compounds isolated from *Callyspongia* species.

Through NMR data, 212 metabolites were identified from genus Callyspongia (15 species and Callyspongia sp.), which were classifying into the following groups: polyacetylenes, polyketides, terpenoids and steroids, simple phenols and phenylpropanoids, alkaloids, nucleosides, cyclic peptides and cyclic depsipeptides, and miscellaneous (Figure 9).

## 6. Conclusions

Sponges of the *Callyspongia* genus are producers of several classes of primary and secondary metabolites, mainly polyacetylenes and lipids. In addition, many of these compounds are biologically active and have activities that may prove to be promising in fighting diseases. Thus, this literature review gathered essential information for the emergence of new research on the species of the genus.

## Supplementary Materials

The following are available online at https://www.mdpi.com/article/10.3390/md19120663/s1, Table S1: Polyacetylenes isolated from *Callyspongia* species, Table S2: Terpenoids and steroids isolated from *Callyspongia* species, Table S3: Alkaloids isolated from *Callyspongia* species, Table S4: Simple phenols and phenylpropanoids isolated from *Callyspongia* species, Table S5: Nucleosides isolated from *Callyspongia* species, Table S6: Cyclic peptides and cyclic depsipeptides isolated from *Callyspongia* species. Table S7: Polyketides isolated from *Callyspongia* species, Table S8: Miscellaneous compounds isolated from *Callyspongia* species.

## References

[1] WoRMS Editorial Board (2020). Word Register of Marine Species. http://www.marinespecies.org.

[2] Desqueyroux-Faúndez R., Valentine C. (2002). Family Callyspongiidae de Laubenfels, 1936. Systema Porifera.

[3] Busutil L., García-Hernández M.R., Díaz M.C., Pomponi S.A. (2018). Mesophotic sponges of the genus *Callyspongia* (Demospongiae, Haplosclerida) from Cuba, with the description of two new species. Zootaxa.

[4] Ibrahim S.R.M., Min C.C., Teuscher F., Ebel R., Kakoschke C., Lin W., Wray V., Edrada-Ebel R., Proksch P. (2010). Callyaerins A-F and H, new cytotoxic cyclic peptides from the Indonesian marine sponge *Callyspongia aerizusa*. Bioorg. Med. Chem..

[5] Kim C.K., Woo J.K., Lee Y.J., Lee H.S., Sim C.J., Oh D.C., Oh K.B., Shin J. (2016). Callyazepin and (3R)-methylazacyclodecane, nitrogenous macrocycles from a *Callyspongia* sp. sponge. J. Nat. Prod..

[6] Kapojos M.M., Abdjul D.B., Yamazaki H., Ohshiro T., Rotinsulu H., Wewengkang D.S., Sumilat D.A., Tomoda H., Namikoshi M., Uchida R. (2018). Callyspongiamides A and B, sterol *O*-acyltransferase inhibitors, from the Indonesian marine sponge *Callyspongia* sp. Bioorg. Med. Chem. Lett..

[7] El-Hawary S.S., Sayed A.M., Mohammed R., Hassan H.M., Rateb M.E., Amin E., Mohammed T.A., El-Mesery M., Muhsinah A.B., Alsayari A. (2019). Bioactive brominated oxindole alkaloids from the red sea sponge *Callyspongia siphonella*. Mar. Drugs.

[8] Sobahi T.R.A., Ayyad S.E.N., Abdel-Lateff A., Algandaby M.M., Alorfi H.S., Abdel-Naim A.B. (2017). Cytotoxic metabolites from *Callyspongia siphonella* display antiproliferative activity by inducing apoptosis in HCT-116 cells. Pharmacogn. Mag..

[9] Layne T.H., Tinto W.F. (2006). A butenolide from the marine sponge *Callyspongia vaginalis*. Heterocycles.

[10] Araújo R.D., Caridade T.N.S., Araújo R.M. (2018). Sulfated polysaccharide from the marine sponge *Callyspongia vaginalis*. Rev. Virtual Quim..

[11] Gray C.A., Lira S.P., Silva M., Pimenta E.F., Thiemann O.H., Oliva G., Hajdu E., Andersen R.J., Berlinck R.G.S. (2006). Sulfated meroterpenoids from the brazilian sponge *Callyspongia* sp. are inhibitors of the antileishmaniasis target adenosine phosphoribosyl transferase. J. Org. Chem..

[12] Calabro K., Chalén B.E., Genta-Jouve G., Jaramillo K.B., Domínguez C., Cruz M., Cautain B., Reyes F., Thomas O.P., Rodríguez J. (2018). Callyspongidic acids: Amphiphilic diacids from the tropical eastern pacific sponge *Callyspongia* cf. *californica*. J. Nat. Prod..

[13] Van Soest R.W.M. (1980). Marine sponges from Curaçao and other Caribbean localities Part II. Haplosclerida. Stud. Fauna Curaçao Other Caribb. Isl..

[14] Lane A.L., Moore B.S. (2011). A sea of biosynthesis: Marine natural products meet the molecular age. Nat. Prod. Rep..

[15] Conte M., Fontana E., Nebbioso A., Altucci L. (2021). Marine-derived secondary metabolites as promising epigenetic bio-compounds for anticancer therapy. Mar. Drugs.

[16] Reynolds W.F., Mazzola E.P. (2014). Nuclear magnetic resonance in the structural elucidation of natural products. Progress in the Chemistry of Organic Natural Products.

[17] Urban S., Capon R.J. (1997). A New lipid from an australian marine sponge, *Callyspongia* sp. Lipids.

[18] Yang B., Tao H., Zhou X., Lin X.P., Liu Y. (2012). Two new alkaloids from marine sponge *Callyspongia* sp. Nat. Prod. Res..

[19] Youssef D.T.A., Ibrahim A.K., Khalifa S.I., Mesbah M.K., Mayer A.M.S., Soest R.W.M. (2010). New Anti-inflammatory sterols from the red sea sponges *Scalarispongia aqabaensis* and *Callyspongia siphonella*. Nat. Prod. Commun..

[20] Huang R., Zhou X., Peng Y., Yang X., Xu T., Liu Y. (2011). Nucleosides from the marine sponge *Callyspongia* sp. Chem. Nat. Compd..

[21] Umeyama A., Nagano C., Arihara S. (1997). Three novel C_21_ polyacetylenes from the marine sponge *Callyspongia* sp. J. Nat. Prod..

[22] Daletos G., Kalscheuer R., Koliwer-Brandl H., Hartmann R., Voogd N.J., Wray V., Lin W., Proksch P. (2015). Callyaerins from the sarine sponge *Callyspongia aerizusa*: Cyclic peptides with antitubercular activity. J. Nat. Prod..

[23] Rao T.S.P., Sarma N.S., Murthy Y.L.N., Kantamreddi V.S.S.N., Wright C.W., Parameswaran P.S. (2010). New polyhydroxy sterols from the marine sponge *Callyspongia fibrosa* (Ridley & Dendly). Tetrahedron Lett..

[24] Azcuna M., Tun J.O., Yap H.T., Concepcion G.P. (2018). *Callyspongia samarensis* (Porifera) extracts exhibit anticancer activity and induce bleaching in *Porites cylindrica* (Scleractinia). Chem. Ecol..

[25] Shmueli U., Carmely S., Groweiss A., Kashman Y. (1981). Sipholenol and Sipholenone, two new triterpenes from the marine sponge *Siphonochalina Siphonella*. Tetrahedron Lett..

[26] Chakraborty K., Francis P. (2020). Callypyrones from marine Callyspongiidae sponge *Callyspongia diffusa*: Antihypertensive bis-γ-pyrone polypropionates attenuate angiotensin-converting enzyme. Nat. Prod. Res..

[27] Youssef D.T.A., Yoshida W.Y., Kelly M., Scheuer P.J. (2000). Polyacetylenes from a red sea sponge *Callyspongia* species. J. Nat. Prod..

[28] Abdelmohsen U.R., Cheng C., Reimer A., Kozjak-Pavlovic V., Ibrahim A.K., Rudel T., Hentschel U., Edrada-Ebel R., Ahmed S.A. (2015). Antichlamydial sterol from the red sea sponge *Callyspongia* aff *implexa*. Planta Med..

[29] Tsukamoto S., Kato H., Hirota H., Fusetani N. (1997). Seven new polyacetylene derivatives, showing both potent metamorphosis-inducing activity in ascidian larvae and antifouling activity against barnacle larvae, from the marine Sponge *Callyspongia truncata*. J. Nat. Prod..

[30] Miao S., Andersen R.J. (1991). Callydiyne, a new diacetylenic hydrocarbon from the sponge *Callyspongla flammea*. J. Nat. Prod..

[31] Umeyama A., Matsuoka N., Mine R., Nakata A., Arimoto E., Matsui M., Shoji N., Arihara S., Takei M., Hashimoto T. (2010). Polyacetylene diols with antiproliferative and driving Th1 polarization effects from the marine sponge *Callyspongia* sp. J. Nat. Med..

[32] Fusetani N., Sugano M., Matsunaga S., Hashimoto K. (1987). H,K-atpase inhibitors from the marine sponge *Siphonochalina truncata*: Absolute configuration of siphonodiol and two related metabolites. Tetrahedron Lett..

[33] Tada H., Yasuda F. (1984). Siphonodiol, a new polyacetylenic metabolite from the sponge *Siphonochalina truncate*. Chem. Lett..

[34] Braekman J.C., Daloze D., Devijver C., Dubut D., Soest R.W.M. (2003). A new C-20 polyacetylene from the sponge *Callyspongia pseudoreticulata*. J. Nat. Prod..

[35] Shirouzu T., Watari K., Ono M., Koizumi K., Saiki I., Tanaka C., Soest R.W.M., Miyamoto T. (2013). Structure, synthesis, and biological activity of a C-20 bisacetylenic alcohol from a marine sponge *Callyspongia* sp. J. Nat. Prod..

[36] Ayyad S.E.N., Angawy R., Alarif W.M., Saqer E.A., Badria F.A. (2014). Cytotoxic polyacetylenes from the red sea sponge *Siphonochalina siphonella*. Z. Nat. C.

[37] Balansa W., Trianto A., Voogd N.J., Tanaka J. (2017). A new cytotoxic polyacetylenic alcohol from a sponge *Callyspongia* sp. Nat. Prod. Commun..

[38] Youssef D.T.A., Soest R.W.M., Fusetani N. (2003). Callyspongenols A-C, new cytotoxic C_22_-polyacetylenic alcohols from a red sea sponge, *Callyspongia* species. J. Nat. Prod..

[39] Chiu C.W., Su H.J., Lu M.C., Wang W.H., Sheu J.H., Su J.H. (2014). Cytotoxic polyacetylenes from a formosan marine sponge *Callyspongia* sp. Bull. Chem. Soc. Jpn..

[40] Ki D.W., El-Desoky A.H., Wong C.P., Abdel-Ghani M., El-Beih A.A., Mizuguchi M., Morita H. (2020). New cytotoxic polyacetylene alcohols from the egyptian marine sponge *Siphonochalina siphonella*. J. Nat. Med..

[41] Fujita M., Nakao Y., Matsunaga S., Soest R.W.M., Itoh Y., Seiki M., Fusetani N. (2003). Callysponginol sulfate A, an MT1-MMP inhibitor isolated from the marine sponge *Callyspongia truncata*. J. Nat. Prod..

[42] Uno M., Ohta S., Ohta E., Ikegami S. (1996). Callyspongins A and B: Novel polyacetylene sulfates from the marine sponge *Callyspongia truncata* that inhibit fertilization of starfish gametes. J. Nat. Prod..

[43] Rooney F., Capon R.J. (1998). Callyspongynes A and B: New polyacetylenic lipids from a southern Australian marine sponge, *Callyspongia* sp. Lipids.

[44] Nakao Y., Uehara T., Matunaga S., Fusetani N., Soest R.W.M. (2002). Callyspongynic acid, a polyacetylenic acid which inhibits α-glucosidase, from the marine sponge *Callyspongia truncata*. J. Nat. Prod..

[45] Xiao-Jian L., Shi-Hai X., Qi-Chang H., Dong-Hong H. (2005). Studies on chemical constituents from *Callyspongia fibrosa*. Chin. J. Spectrosc. Lab..

[46] Youssef D.T.A., Soest R.W.M., Fusetani N. (2003). Callyspongamide A, a new cytotoxic polyacetylenic amide from the red sea sponge *Callyspongia fistularis*. J. Nat. Prod..

[47] Rotem M., Kashman Y. (1979). New polyacetylenes from the sponge *Siphonochalina* sp. Tetrahedron Lett..

[48] Delbeke E.I.P., Everaert J., Uitterhaegen E., Verweire S., Verlee A., Talou T., Soetaert W., Bogaert I.N.A., Stevens C.V. (2016). Petroselinic acid purification and its use for the fermentation of new sophorolipids. AMB Express.

[49] Garg H.S., Agraval S. (1995). Callyspinol, a new diterpene from sponge *Callyspongia spinossima*. Tetrahedron Lett..

[50] Kurnianda V., Faradilla S., Karina S., Agustina S., Ulfah M., Octavina C., Syahliza F., Ramadhan M.R., Purnawan S., Musman M. (2019). Polyoxygenated diterpene produced by the indonesian marine sponge *Callyspongia* sp. as an inhibitor of the human pancreatic cancer cells. Microbiol. Indones..

[51] Fukami A., Ikeda Y., Kondo S., Naganawa H., Takeuchi T., Furuya S., Hirabayashi Y., Shimoike K., Hosaka S., Watanabe Y. (1997). Akaterpin, a novel bioactive triterpene from the marine sponge *Callyspongia* sp. Tetrahedron Lett..

[52] Jain S., Abraham I., Carvalho P., Kuang Y.H., Shaala L.A., Youssef D.T.A., Avery M.A., Chen Z.S., Sayed K.A. (2009). Sipholane triterpenoids: Chemistry, reversal of ABCB1/P-glycoprotein-mediated multidrug resistance, and pharmacophore modeling. J. Nat. Prod..

[53] Kashman Y., Yosief T., Carmeli S. (2001). New triterpenoids from the red sea sponge *Siphonochalina siphonella*. J. Nat. Prod..

[54] Carmely S., Kashman Y. (1986). Neviotine-A, a new triterpene from the red sea sponge *Siphonochalina siphonella*. J. Org. Chem..

[55] Ayyad S.E.N., Angawi R.F., Saqer E., Abdel-Lateff A., Badria F.A. (2014). Cytotoxic neviotane triterpene-type from the red sea sponge *Siphonochalina siphonella*. Pharmacogn. Mag..

[56] Al-Massarani S.M., El-Gamal A.A., Al-Said M.S., Al-Lihaibi S.S., Basoudan O.A. (2015). In vitro cytotoxic, antibacterial and antiviral activities of triterpenes from the red sea sponge, *Siphonochalina siphonella*. Trop. J. Pharm. Res..

[57] El-Beih A.A., El-Desoky A.H., Al-hammady M.A., Elshamy A.I., Hegazy M.E.F., Kato H., Tsukamoto S. (2018). New inhibitors of RANKL-induced osteoclastogenesis from the marine sponge *Siphonochalina siphonella*. Fitoterapia.

[58] Jain S., Shirode A., Yacoub S., Barbo A., Sylvester P.W., Huntimer E., Halaweish F., Sayed K.A. (2007). Biocatalysis of the anticancer sipholane triterpenoids. Planta Med..

[59] Jain S., Laphookhieo S., Shi Z., Fu L.W., Akiyama S.I., Chen Z.S., Youssef D.T.A., Soest R.W.M., Sayed K.A. (2007). Reversal of P-glycoprotein-mediated multidrug resistance by sipholane triterpenoids. J. Nat. Prod..

[60] Al-Lhaibi S.S., Abdel-Lateff A., Alarif W.M., Nogata Y., Ayyad S.E.N., Okino T. (2015). Potent antifouling metabolites from red sea organisms. Asian J. Chem..

[61] Carmely S., Kashman Y. (1983). The sipholanes: A novel group of triterpenes from the marine sponge *Siphonochalina siphonella*. J. Org. Chem..

[62] Carmely S., Loya Y., Kashman Y. (1983). Siphonellinol, a new triterpene from the marine sponge *Siphonochalina siphonella*. Tetrahedron Lett..

[63] Alam P., Alqahtani A.S., Husain F.M., Rehman M.T., Alajmi M.F., Noman O.M., Gamal A.A., Al-Massarani S.M., Khan M.S. (2020). Siphonocholin isolated from red sea sponge *Siphonochalina siphonella* attenuates quorum sensing controlled virulence and biofilm formation. Saudi Pharm. J..

[64] Carmely S., Kashman Y. (1986). The study of sipholanes by two-dimensional NMR spectroscopy. Magn. Reson. Chem..

[65] Plisson F., Prasad P., Xiao X., Piggott A.M., Huang X.C., Khalil Z., Capon R.J. (2014). Callyspongisines A-D: Bromopyrrole alkaloids from an australian marine sponge, *Callyspongia* sp. Org. Biomol. Chem..

[66] Yang B., Huang J., Lin X., Zhang Y., Tao H., Liu Y. (2016). A new diketopiperazine from the marine sponge *Callyspongia* species. Rec. Nat. Prod..

[67] Yang B., Hu J., Lei H., Chen X.Q., Zhou X.F., Liu Y.H. (2012). Chemical constituents of marine sponge *Callyspongia* sp. from the south China sea. Chem. Nat. Compd..

[68] Wang G.Y.S., Kuramoto M., Uemura D., Yamada A., Yamaguchi K., Yazawa K. (1996). Three Novel Anti-mierofouling nitroalkyl pyridine alkaloids from the Okinawan marine sponge *Callyspongia* sp. Tetrahedron Lett..

[69] Buchanan M.S., Carroll A.R., Addepalli R., Avery V.M., Hooper J.N.A., Quinn R.J. (2007). Niphatoxin C, a cytotoxic tripyridine alkaloid from *Callyspongia* sp. J. Nat. Prod..

[70] Huang R.M., Ma W., Dong J.D., Zhou X.F., Xu T., Lee K.J., Yang X., Xu S.H., Liu Y. (2010). A new 1,4-diazepine from south China sea marine sponge *Callyspongia* species. Molecules.

[71] Yang B., Dong J., Zhou X., Yang X., Lee K.J., Wang L., Zhang S., Liu Y. (2009). Proline-containing dipeptides from a marine sponge of a *Callyspongia* Species. Helv. Chim. Acta..

[72] Chen Y., Peng Y., Gao C., Huang R. (2014). A new diketopiperazine from South China Sea marine sponge *Callyspongia* sp. Nat. Prod. Res..

[73] Sperry S., Crews P. (1996). A novel alkaloid from the indo-pacific sponge *Clathria basilana*. Tetrahedron Lett..

[74] Gopichand Y., Schmitz F.J. (1979). Two novel lactams from the marine sponge *Halichondria melanodocia*. J. Org. Chem..

[75] Jayatilake G.S., Thornton M.P., Leonard A.C., Grimwade J.E., Baker B.J. (1996). Metabolites from an antarctic sponge-associated bacterium, *Pseudomonas aeruginosa*. J. Nat. Prod..

[76] Adamczeski M., Reed A.R., Crews P. (1995). New and known diketopiperazines from the Caribbean sponge, *Calyx* cf. Podatypa. J. Nat. Prod..

[77] Gautschi M., Schmid J.P., Peppard T.L., Ryan T.P., Tuorto R.M., Yang X. (1997). Chemical characterization of diketopiperazines in beer. J. Agric. Food Chem..

[78] Stark T., Hofmann T. (2005). Structures, sensory activity, and dose/response functions of 2,5-diketopiperazines in roasted cocoa nibs (*Theobroma cacao*). J. Agric. Food Chem..

[79] Fdhila F., Vázquez V., Sánchez J.L., Riguera R. (2003). DD-diketopiperazines: Antibiotics active against *Vibrio anguillarum* isolated from marine bacteria associated with cultures of *Pecten maximus*. J. Nat. Prod..

[80] Tian L.W., Feng Y., Shimizu Y., Pfeifer T.A., Wellington C., Hooper J.N.A., Quinn R.J. (2014). ApoE secretion modulating bromotyrosine derivative from the australian marine sponge *Callyspongia* sp. Bioorg. Med. Chem. Lett..

[81] Weller D.D., Stirchak E.P., Yokoyama A. (1984). Preparation of oxygenated phenylacetic acids. J. Org. Chem..

[82] Ibrahim S.R.M., Edrada-Ebel R.A., Mohamed G.A., Youssef D.T.A., Wray V., Proksch P. (2008). Callyaerin G, a new cytotoxic cyclic peptide from the marine sponge *Callyspongia aerizusa*. Arkivoc.

[83] Berer N., Rudi A., Goldberg I., Benayahu Y., Kashman Y. (2004). Callynormine A, a new marine cyclic peptide of a novel class. Org. Lett..

[84] Shaala L.A., Youssef D.T.A., Ibrahim S.R.M., Mohamed G.A. (2016). Callyptide A, a new cytotoxic peptide from the red sea marine sponge *Callyspongia* species. Nat. Prod. Res..

[85] Capon R.J., Ford J., Lacey E., Gill J.H., Heiland K., Friedel T. (2002). Phoriospongin A and B: Two new nematocidal depsipeptides from the Australian marine sponges *Phoriospongia* sp. and *Callyspongia bilamellata*. J. Nat. Prod..

[86] Kobayashi M., Higuchi K., Murakami N., Tajima H., Aoki S. (1997). Callystatin A, a potent cytotoxic polyketide from the marine sponge, *Callyspongia truncata*. Tetrahedron Lett..

[87] Murakami N., Wang W., Aoki M., Tsutsui Y., Higuchi K., Aoki S., Kobayashi M. (1997). Absolute stereostructure of Callystatin A, a potent cytotoxic polyketide from the marine sponge, *Callyspongia truncata*. Tetrahedron Lett..

[88] Pham C.D., Hartmann R., Böhler P., Stork B., Wesselborg S., Lin W., Lai D., Proksch P. (2014). Callyspongiolide, a cytotoxic macrolide from the marine sponge *Callyspongia* sp. Org. Lett..

[89] Koshino H., Yoshihara T., Sakamura S., Shimanuki T., Sato T., Tajimi A. (1989). Novel C-11 epoxy fatty acid from stromata of *Epichloe typhina* on *Phleum pratense*. Agric. Biol. Chem..

[90] Mori K., Khlebnikov V. (1993). Synthesis of (+)-dihydroactinidiolide, (+)- and (−)-actinidiolide, (+)- and (−)-loliolide as well as (+)- and (−)-epiloliolide. Liebigs Ann. Chem..

[91] Huang R., Chen Y., Zhou X., Yang X., Liu Y. (2015). A new n-acyl taurine from the South China Sea marine sponge *Callyspongia* sp. Chem. Nat. Compd..

[92] Toth S.I., Schmitz F.J. (1994). Two new cytotoxic peroxide-containing acids from a new guinea sponge, *Callyspongia* sp. J. Nat. Prod..

[93] Kazlauskas R., Lidgard R.O., Murphy P.T., Wells R.J., Blount J.F. (1981). Brominated tyrosine-derived metabolites from the sponge *Ianthella basta*. Aust. J. Chem..

[94] Greve H., Kehraus S., Krick A., Kelter G., Maier A., Fiebig H.H., Wright A.D., Konig G.M. (2008). Cytotoxic bastadin 24 from the australian sponge *Ianthella quadrangulata*. J. Nat. Prod..

[95] Jaspars M., Rali T., Laney M., Schatzman R.C., Diaz M.C., Schmitz F.J., Pordesimo E.O., Crews P. (1994). The Search for inosine 5’-phosphate dehydrogenase (IMPDH) inhibitors from marine sponges. Evaluation of the bastadin alkaloids. Tetrahedron.

[96] López S., Fernández-Trillo F., Midón P., Castedo L., Saá C. (2005). First stereoselective syntheses of (−)-siphonodiol and (−)-tetrahydrosiphonodiol, bioactive polyacetylenes from marine sponges. J. Org. Chem..

[97] Díaz Y.M., Laverde G.V., Gamba L.R., Wandurraga H.M., Arévalo-Ferro C., Rodríguez F.R., Beltrán C.D., Hernández L.C. (2015). Biofilm inhibition activity of compounds isolated from two *Eunicea* species collected at the Caribbean Sea. Rev. Bras. Farmacogn..

[98] Shi Z., Jain S., Kim I.W., Peng X.X., Abraham I., Youssef D.T.A., Fu L.W., Sayed K., Ambudkar S.V., Chen Z.S. (2007). Sipholenol A, a marine-derived sipholane triterpene, potently reverses P-glycoprotein (ABCB1)-mediated multidrug resistance in cancer cells. Cancer Sci..

[99] Ma J., Fu G., Wu J., Han S., Zhang L., Yang M., Yu Y., Zhang M., Lin Y., Wang Y. (2016). 4-cholesten-3-one suppresses lung adenocarcinoma metastasis by regulating translocation of HMGB1, HIF1α and Caveolin-1. Cell Death Dis..

[100] Villaseñor I.M., Angelada J., Canlas A.P., Echegoyen D. (2002). Bioactivity studies on β-sitosterol and its glucoside. Phytother. Res..

[101] Choi S., Kim K.W., Choi J.S., Han S.T., Park Y.I., Lee S.K., Kim J.S., Chung M.H. (2002). Angiogenic activity of β-sitosterol in the ischaemia/reperfusion-damaged brain of mongolian gerbil. Planta Med..

[102] Beltrame F.L., Pessini G.L., Doro D.L., Filho B.P.D., Bazotte R.B., Cortez D.A.G. (2002). Evaluation of the antidiabetic and antibacterial activity of *Cissus sicyoides*. Braz. Arch. Biol. Technol..

[103] Sen A., Dhavan P., Shukla K.K., Singh S., Tejovathi G. (2012). Analysis of IR, NMR and antimicrobial activity of β-sitosterol isolated from *Momordica charantia*. Sci. Secur. J. Biotech..

[104] Kiprono P.C., Kaberia F., Keriko J.M., Karanja J.N. (2000). The in vitro Anti-fungal and anti-bacterial activities of β-sitosterol from *Senecio lyratus* (Asteraceae). Verl. Z. Nat..

[105] Zeb M.A., Khan S.U., Rahman T.U., Sajid M., Seloni S. (2017). Isolation and biological activity of β-sitosterol and stigmasterol from the roots of *Indigofera heterantha*. Pharm. Pharmacol. Int. J..

[106] Nirmal S.A., Pal S.C., Mandal S.C., Patil A.N. (2012). Analgesic and anti-inflammatory activity of β-sitosterol isolated from *Nyctanthes arbortristis* leaves. Inflammopharmacology.

[107] Gupta M.B., Nath R., Srivastava N., Shanker K., Kishor K., Bhargava K.P. (1980). Anti-inflammatory and antipyretic activities of β-sitosterol. Planta Med..

[108] Loizou S., Lekakis I., Chrousos G.P., Moutsatsou P. (2010). β-Sitosterol exhibits anti-inflammatory activity in human aortic endothelial cells. Mol. Nutr. Food Res..

[109] Chai J.W., Kuppusamy U.R., Kanthimathi M.S. (2008). Beta-sitosterol induces apoptosis in MCF-7 Cells. Malays. J. Biochem. Mol. Biol..

[110] Awad A.B., Holtz R.L., Cone J.P., Fink C.S., Chen Y.C. (1998). Beta-sitosterol inhibits growth of HT-29 human colon cancer cells by activating the sphingomyelin cycle. Anticancer Res..

[111] Park C., Moon D.O., Rhu C.H., Choi B.T., Lee W.H., Kim G.Y., Choi Y.H. (2007). β-sitosterol induces anti-proliferation and apoptosis in human leukemic U937 cells through activation of caspase-3 and induction of Bax/Bcl-2 ratio. Biol. Pharm. Bull..

[112] Vundru S.S., Kale R.K., Singh R.P. (2013). β-sitosterol induces G1 arrest and causes depolarization of mitochondrial membrane potential in breast carcinoma MDA-MB-231 cells. BMC Complement. Altern. Med..

[113] Zhao Y., Chang S.K.C., Qu G., Li T., Cui H. (2009). β-sitosterol inhibits cell growth and induces apoptosis in SGC-7901 human stomach cancer cells. J. Agric. Food Chem..

[114] Holtz R.L., Fink C.S., Awad A.B. (1998). β-sitosterol activates the sphingomyelin cycle and induces apoptosis in LNCaP human prostate cancer cells. Nutr. Cancer.

[115] Sugano M., Morioka H., Ikeda I. (1977). A Comparison of hypocholesterolemic activity of β-sitosterol and β-sitostanol in rats. J. Nutr..

[116] Fraile L., Crisci E., Córdoba L., Navarro M.A., Osada J., Montoya M. (2012). Immunomodulatory properties of beta-sitosterol in pig immune responses. Int. Immunopharmacol..

[117] Izumida M., Suga K., Ishibashi F., Kubo Y. (2019). The spirocyclic imine from a marine benthic dinoflagellate, portimine, is a potent anti-human immunodeficiency virus type 1 therapeutic lead compound. Mar. Drugs.

[118] Tasdemir D., Mallon R., Greenstein M., Feldberg L.R., Kim S.C., Collins K., Wojciechowicz D., Mangalindan G.C., Concepcio G.P., Harper M.K. (2002). Aldisine alkaloids from the philippine sponge *Stylissa massa* are potent inhibitors of mitogen-activated protein kinase kinase-1 (MEK-1). J. Med. Chem..

[119] Ebada S.S., Linh M.H., Longeon A., Voogd N.J., Durieu E., Meijer L., Bourguet-Kondracki M.L., Singab A.N.B., Müller W.E.G., Proksch P. (2015). Dispacamide E and other bioactive bromopyrrole alkaloids from two Indonesian marine sponges of the genus *Stylissa*. Nat. Prod. Res..

[120] Meijer L., Thunnissen A.M.W.H., White A.W., Garnier M., Nikolic M., Tsai L.H., Walter J., Cleverley K.E., Salinas P.C., Wu Y.Z. (2000). Inhibition of cyclin-dependent kinases, GSK-3β and CK1 by hymenialdisine, a marine sponge constituent. Chem. Biol..

[121] Andrioli W.J., Santos M.S., Silva V.B., Oliveira R.B., Chagas-Paula D.A., Jorge J.A., Furtado N.A.J.C., Pupo M.T., Silva C.H.T.P., Naal R.M.Z.G. (2012). δ-Lactam derivative from thermophilic soil fungus exhibits in vitro anti-allergic activity. Nat. Prod. Res..

[122] Ishikawa M., Yoshida J., Ide N., Sasaoka T., Yamaguchi H., Ono K. (2006). Tetrahydro-β-carboline derivatives in aged garlic extract show antioxidant properties. J. Nutr..

[123] Shimizu K., Geng X., Hashiguchi M., Suhara H., Fukunaga S., Yasutake S., Kondo R., Tsutsui M., Sato I. (2003). Indole-3-carbaldehyde: A tyrosinase inhibitor from fungus YL185. J. Wood Sci..

[124] Zeng M., Li M., Li M., Zhang B., Li B., Zhang L., Feng W., Zheng X. (2018). 2-Phenylacetamide isolated from the seeds of *Lepidium apetalum* and its estrogen-like effects in vitro and in vivo. Molecules.

[125] Takai M., Miyamoto S., Hattori Y., Tamura S. (1963). Isolation of 2-phenylacetamide as a plant growth regulator produced by *Actinomyces*. Agric. Biol. Chem..

[126] Cho J.Y., Moon J.H., Seong K.Y., Park K.H. (1998). Antimicrobial Activity of 4-hydroxybenzoic acid and *trans* 4-hydroxycinnamic acid isolated and identified from rice hull. Biosci. Biotechnol. Biochem..

[127] Chong K.P., Rossall S., Atong M. (2009). In vitro antimicrobial activity and fungitoxicity of syringic acid, caffeic acid and 4-hydroxybenzoic acid against *Ganoderma Boninense*. J. Agric. Sci..

[128] Peungvicha P., Temsiririrkkul R., Prasain J.K., Tezuka Y., Kadota S., Thirawarapan S.S., Watanabe H. (1998). 4-hydroxybenzoic acid: A hypoglycemic constituent of aqueous extract of *Pandanus odorus* root. J. Ethnopharmacol..

[129] Iizuka H., Adachi R., Koizumi H., Aoyagi T., Ohkawara A., Miura Y. (1984). Effects of adenosine and 2’-deoxyadenosine on epidermal keratinocyte proliferation: Its relation to cyclic AMP formation. J. Investig. Dermatol..

[130] Zhao Z., Crossland W.J., Kulkarni J.S., Wakade T.D., Wakade A.R. (1999). 2’-Deoxyadenosine causes cell death in embryonic chicken sympathetic ganglia and brain. Cell Tissue Res..

[131] Zhang S., Rodriguez L.M.L., Leung I.K.H., Cook G.M., Harris P.W.R., Brimble M.A. (2018). Total synthesis and conformational study of the anti-tubercular cyclic peptide callyaerin a bearing a rare rigidifying (z)-2,3- diaminoacrylamide moiety. Angew. Chem. Int. Ed..

[132] Fogarty S., Ouyang Y., Li L., Chen Y.C., Rane H., Manoni F., Parra K.J., Rutter J., Harran P.G. (2020). Callyspongiolide is a potent inhibitor of the vacuolar ATPase. J. Nat. Prod..

[133] Zhang H.J., Hung N., Cuong N.M., Soejarto D.D., Pezzuto J.M., Fong H.H.S., Tan G.T. (2005). Sesquiterpenes and butenolides, natural anti-HIV constituents from *Litsea verticillata*. Planta Med..

[134] Xu L., Tao X., Gao Y., Zhang W., Meng Y., Li C., Jiang M., Ying X. (2017). Cytotoxicity of hydroxydihydrobovolide and its pharmacokinetic studies in *Portulaca oleracea* L. extract. Braz. J. Pharm. Sci..

[135] Hasegawa T., Yamada K., Shigemori H., Hasegava K., Miyamoto K., Ueda J. (2002). Isolation and identification of a growth inhibitor from blue light-illuminated cress seedlings. Plant Growth Regul..

[136] Elkhayat E.S. (2009). Cytotoxic and antibacterial constituents from the roots of *Sonchus oleraceus* L. growing in Egypt. Pharmacogn. Mag..

[137] Zajdel S.M., Graikou K., Głowniak K., Chinou I. (2012). Chemical analysis of *Penstemon campanulatus* (Cav.) Willd.–Antimicrobial activities. Fitoterapia.

[138] Grabarczyk M., Wińska K., Mączka W., Potaniec B., Anioł M. (2015). Loliolide–the most ubiquitous lactone. Folia Biol. Oecologica.

[139] Neergaard J.S., Rasmussen H.B., Stafford G.I., Staden J., Jäger A.K. (2010). Serotonin transporter affinity of (−)-loliolide, a monoterpene lactone from *Mondia whitei*. S. Afr. J. Bot..

[140] Ragasa C.Y., Agbayani V., Hernández R.B., Rideout J.A. (1997). An Antimutagenic monoterpene from *Malachra Fasciata* (Malvaceae). Philipp. J. Sci..

[141] Yang X., Kang M.C., Lee K.W., Kang S.M., Lee W.W., Jeon Y.J. (2011). Antioxidant activity and cell protective effect of loliolide isolated from *Sargassum ringgoldianum* subsp. *coreanum*. Algae.

[142] Kuniyoshi M. (1985). Germination inhibitors from the brown alga *Sargassum crassifolium* (Phaeophyta, Sargassaceae). Bot. Mar..

[143] Aoki S., Cho S.H., Ono M., Kuwano T., Nakao S., Kuwano M., Nakagawa S., Gao J.Q., Mayumi T., Shibuya M. (2006). Bastadin 6, a spongean brominated tyrosine derivative, inhibits tumor angiogenesis by inducing selective apoptosis to endothelial cells. Anti-Cancer Drugs.

[144] Mathieu V., Wauthoz N., Lefranc F., Niemann H., Amighi K., Kiss R., Proksch P. (2013). Cyclic *versus* hemi-bastadins. Pleiotropic anti-cancer effects: From apoptosis to anti-angiogenic and anti-migratory effects. Molecules.

[145] Niemann H., Lin W., Müller W.E.G., Kubbutat M., Lai D., Proksch P. (2013). trimeric hemibastadin congener from the marine sponge *Ianthella basta*. J. Nat. Prod..

[146] Resuello D.L., Lirio S.B., Porto A.E., Macabeo A.P.G., Huang H.Y., Corpuz M.J.A.T., Villaflores O.B. (2018). β-secretase 1 inhibitory activity and AMP-activated protein kinase activation of *Callyspongia samarensis* extracts. Nat. Prod. Res..

[147] El-Damhougy K.A., Bashar M.A.E., El-Naggar H.A., Ibrahim H.A.N., Senna F.M.A. (2017). GC-MS analysis of bioactive components of *Callyspongia crassa* (porifera) from gulf of aqaba red sea (egypt). Al Azhar Bull. Sci..

[148] Carballeira N.M., Pagán M. (2001). New methoxylated fatty acids from the Caribbean sponge *Callyspongia fallax*. J. Nat. Prod..

[149] Edrada R.A., Wray V., Berg A., Gräfe U., Brauers G., Proksch P. (2000). Novel spiciferone derivatives from the fungus *Drechslera hawaiiensis* isolated from the marine sponge *Callyspongia aerizusa*. Verl. Z. Nat..

