# Supplementary material for: Metabolities from Marine Sponges of the Genus Callyspongia: Occurrence, Biological Activity, and NMR Data
Source: Mar Drugs. 2021 Nov 26;19(12):663. doi: 10.3390/md19120663 (PMC8706505; doi:10.3390/md19120663)
Supplement: Supplementary file 1 [file marinedrugs-19-00663-s001.zip › marinedrugs-1447474-supplementary.pdf]

Supplementary Material

METABOLITES FROM MARINE SPONGES OF THE GENUS *Callyspongia*: OCCURRENCE, BIOLOGICAL ACTIVITY, AND NMR DATA.

Lucas Sousa [1], Rusceli Araújo [1], Déborah Sousa-Fontoura [2], Fabrício Menezes [1] and Renata Araújo [1]*

[1] Instituto de Química, Universidade Federal do Rio Grande do Norte, 59078-970, Natal-RN, Brasil; renata.mendonça@ufrn.br

[2] Biotério Central, Universidade Federal do Rio Grande do Norte, 59078-970, Natal-RN, Brasil; deborah.fontoura@ufrn.br

* Correspondence: renat.onca@gmail.com; Tel.: +55-84-99647-4237

Table S1. Polyacetylenes isolated from *Callyspongia* species.

Metabolite name<br>Chemical formula<br>Type of metabolite	Specie<br>Geographic Location	^{1}H and ^{13}C NMR data [Reference]
Aikupikanyne A (**1**)<br>$C_{19}H_{22}$<br>Polyacetylene	*Callyspongia* sp.<br>Egypt	^{1}H NMR (500 MHz, $CDCl_3$, δ/ppm, *J*/Hz): 3.08 (d, 1.9, H-1), 5.46 (ddd, 10.8, 2.2 and 0.7, H-3), 5.96 (dt, 10.8 and 7.4, H-4), 2.34 (q, 7.5, H-5), 1.58-1.51 (m, H-6), 2.29 (t, 6.5, H-7), 2.31 (t, 7.0, H-12), 1.58-1.51 (m, H-13, H-14, H-15, H-16), 2.19 (dt, 6.6 and 2.6, H-17) and 1.95 (t, 2.6, H-19). ^{13}C NMR (125 MHz, $CDCl_3$): 81.2 (C-1), 80.3 (C-2), 108.6 (C-3), 145.1 (C-4), 29.5 (C-5), 27.5 (C-6), 19.2 (C-7), 79.0 (C-8), 60.4 (C-9), 65.8 (C-10), 79.0 (C-11), 19.1 (C-12), 27.6 (C-13), 27.8 (C-14, C-16), 27.4 (C-15), 18.2 (C-17), 84.2 (C-18) and 68.4 (C-19). [27]
Aikupikanyne B (**2**)<br>$C_{21}H_{30}$<br>Polyacetylene	*Callyspongia* sp.<br>Egypt	^{1}H NMR (500 MHz, $CDCl_3$, δ/ppm, *J*/Hz): 3.07 (d, 2.0, H-1), 5.44 (ddt, 11.0, 2.0 and 1.1, H-3), 5.99 (dtd, 11.0, 7.5 and 0.7, H-4), 2.32 (dq, 7.5 and 1.1, H-5), 1.45 (quin., 7.5, H-6), 2.14 (dt, 7.5 and 1.1, H-7), 2.12 (dt, 7.5 and 1.1, H-10), 1.48 (H-11), 1.25 (m, H-12), 1.27 (m, H-13, H-14, H-15), 1.53 (m, H-16, H-17), 1.55 (m, H-18), 2.18 (dt, 7.0 and 2.6, H-19) and 1.93 (t, 2.6, H-21). ^{13}C NMR (125 MHz, $CDCl_3$): 81.1 (C-1), 80.5 (C-2), 107.9 (C-3), 146.2 (C-4), 30.2 (C-5), 28.6 (C-6), 18.6 (C-7), 80.4 (C-8), 79.8 (C-9), 18.7 (C-10), 28.7 (C-11), 29.3 (C-12), 29.1 (C-13, C-14), 29.0 (C-15), 28.8 (C-16), 28.0 (C-17), 27.9 (C-18), 18.3 (C-19), 84.5 (C-20) and 68.1 (C-21). [27]
Aikupikanyne C (**3**)<br>$C_{22}H_{28}$<br>Polyacetylene	*Callyspongia* sp.<br>Egypt	^{1}H NMR (500 MHz, $CDCl_3$, δ/ppm, *J*/Hz): 3.08 (d, 2.2, H-1), 5.48 (ddt, 10.8, 2.2 and 1.2, H-3), 6.02 (dt, 10.8 and 7.5, H-4), 2.45 (dq, 7.5 and 1.2, H-5), 1.67 (quin., 7.3, H-6), 2.37 (dt, 7.2 and 1.9, H-7), 5.42 (H-10), 5.80 (td, 14.8 and 7.5, H-11), 2.27 (dq, 7.5 and 1.2, H-12), 1.33 (m, H-13, H-14), 1.54 (m, H-15), 1.41 (m, H-16, H-17), 2.31 (dq, 7.0 and 1.4, H-18), 5.98 (td, 10.8 and 7.3, H-19), 5.44 (H-20) and 3.07 (d, 2.4, H-22). ^{13}C NMR (125 MHz, $CDCl_3$): 81.6 (C-1), 80.5 (C-2), 108.9 (C-3), 144.8 (C-4), 29.5 (C-5), 28.0 (C-6), 19.1 (C-7), 93.7 (C-8), 77.8 (C-9), 109.3 (C-10), 142.7 (C-11), 30.0 (C-12), 28.9 (C-13, C-14), 27.8 (C-15), 28.8 (C-16), 28.6 (C-17), 30.2 (C-18), 146.2 (C-19), 108.0 (C-20), 80.3 (C-21) and 81.1 (C-20). [27]
Aikupikanyne D (**4**)<br>$C_{20}H_{28}O$	*Callyspongia* sp.<br>Egypt	^{1}H NMR (500 MHz, $CDCl_3$, δ/ppm, *J*/Hz): 2.56 (d, 2.1, H-1), 4.83 (m, H-3), 5.61 (ddt, 15.2, 6.0 and 1.2, H-4),

Polyacetylene		5.91 (dtd, 15.2, 6.0 and 1.1, H-5), 2.06 (q, 7.0, H-6), 1.67-1.25 (m, H-7, H-8, H-9, H-10, H-11, H-12), 2.14 (H-13, H-16), 1.59 (m, H-17), 2.19 (dt, 6.8 and 2.8, H-18) and 1.93 (t, 2.8, H-20). ^{13}C NMR (125 MHz, $CDCl_3$): 74.0 (C-1), 83.3 (C-2), 62.8 (C-3), 128.3 (C-4), 134.5 (C-5), 31.9 (C-6), 29.7-28.4 (C-7, C-8, C-9, C-10, C-11, C-12), 18.7 (C-13), 79.8 (C-14), 80.7 (C-15), 18.7 (C-16), 28.0 (C-17), 18.3 (C-18), 84.8 (C-19) and 68.1 (C-20). [27]
Aikupikanyne E (**5**)<br>$C_{22}H_{24}O$<br>Polyacetylene	*Callyspongia* sp.<br>Egypt	^{1}H NMR (500 MHz, $CDCl_3$, δ/ppm, *J*/Hz): 4.24 (t, 2.3, H-22), 2.20 (tt, 6.8 and 2.1, H-19), 1.58-1.47 (m, H-6, H-18, H-19, H-20, H-21), 2.30 (H-14), 2.29 (t, 7.0, H-7), 2.34 (dq, 7.4 and 1.3, H-5), 5.96 (dtd, 10.8, 7.5 and 0.8, H-4), 5.46 (ddt, 10.8, 2.3 and 1.3, H-3) and 3.08 (tdd, 2.3, 0.8 and 0.4, H-1). ^{13}C NMR (125 MHz, $CDCl_3$): 51.3 (C-22), 78.6 (C-21), 86.0 (C-20), 18.5 (C-19), 27.9 (C-18, C-15), 27.6 (C-17), 27.5 (C-16), 19.1 (C-14), 79.0 (C-13), 65.9 (C-12), 60.4 (C-11), 60.3 (C-10), 65.8 (C-9), 79.0 (C-8), 19.2 (C-7), 27.3 (C-6), 29.7 (C-5), 145.1 (C-4), 108.6 (C-3), 80.3 (C-2) and 81.5 (C-1). [27]
Aikupikanyne F (**6**)<br>$C_{24}H_{26}O_3$<br>Polyacetylene	*Callyspongia* sp.<br>Egypt	^{1}H NMR (500 MHz, DMSO-d_6, δ/ppm, *J*/Hz): 4.32 (br s, H-23), 2.15 (t-like, 6.1, H-20), 1.40 (m, H-19), 1.48 (m, H-18, H-17, H-16, H-15), 2.38 (t, 6.6, H-14), 2.36 (t, 7.0, H-7), 1.48 (m, H-6), 2.24 (q, 6.6, H-5), 6.02 (dt, 10.7 and 7.5, H-4), 5.51 (br d, 10.7, H-3) and 4.08 (br d, 2.0, H-1). ^{13}C NMR (125 MHz, DMSO-d_6): 170.3 (C-24), 62.4 (C-23), 80.4 (C-22), 82.4 (C-21), 18.0 (C-20), 27.6 (C-19, C-16, C-15), 27.2 (C-18), 27.0 (C-17), 18.2 (C-14), 80.7 (C-13), 65.0 (C-12), 60.14 (C-11), 60.18 (C-10), 65.1 (C-9), 80.9 (C-8), 18.4 (C-7), 26.9 (C-6), 29.1 (C-5), 144.9 (C-4), 108.8 (C-3), 80.5 (C-2) and 84.9 (C-1). [27]
Octahydrosiphonochalyne (**7**)<br>$C_{22}H_{32}$<br>Polyacetylene	*Callyspongia* sp.<br>Egypt	^{1}H NMR (500 MHz, $CDCl_3$, δ/ppm, *J*/Hz): 3.06 (d, 2.3, H-1), 5.43 (ddt, 10.6, 2.3 and 1.5, H-3), 6.03 (dtd, 10.6, 7.6 and 0.9, H-4), 2.31 (dq, 7.3 and 1.0, H-5), 1.46 (quin., 7.3, H-6), 2.12 (H-7), 2.24 (tt, 7.1 and 2.4, H-10), 1.56 (quin., 7.1, H-11), 1.67-1.25 (m, H-12, H-13, H-14, H-15, H-16, H-17, H-18), 1.54 (m, H-19), 2.17 (dt, 7.0 and 2.6, H-20) and 1.94 (t, 2.5, H-22). ^{13}C NMR (125 MHz, $CDCl_3$): 81.1 (C-1), 80.4 (C-2), 107.9 (C-3), 146.3 (C-4), 30.2 (C-5), 29.9-28.4 (C-6), 18.6 (C-7), 80.7 (C-8), 79.2 (C-9), 17.9 (C-10), 29.9-28.4 (C-11, C-12, C-

		13, C-14, C-15, C-16, C-17, C-18), 27.9 (C-19), 18.3 (C-20), 84.5 (C-21) and 68.0 (C-22). [27]
Callimplexen A (**8**)<br>$C_{27}H_{38}$<br>Polyacetylene	*Callyspongia implexa*<br>Egypt	^{1}H NMR (400 MHz, $CDCl_3$, δ/ppm, *J*/Hz): 3.01 (s, H-1), 5.47 (d, H-3), 5.97 (m, H-4), 2.15 (m, H-5) 1.54 (m, H-6, H-17, H-18), 2.33 (m, H-7, H-10, H-13, H-22), 1.58 (m, H-11, H-12), 2.36 (m, H-16, H-19), 1.52 (m, H-23), 1.25 (m, H-24, H-25, H-26) and 0.87 (m, H-27). ^{13}C NMR (100 MHz, $CDCl_3$): 81.7 (C-1), 80.4 (C-2, C-8, C-9, C-14, C-15, C-20, C-21), 108.8 (C-3), 145.2 (C-4), 32.0 (C-5, C-7, C-10, C-13, C-16, C-19, C-22, C-23, C-24, C-25 C-26), 19.3 (C-6, C-11, C-12, C-17, C-18) and 14.8 (C-27). [28]
Callyberyne A (Callypentayne) (**9**)<br>$C_{21}H_{20}$<br>Polyacetylene	*Callyspongia truncata*<br>Japan	^{1}H NMR (500 MHz, CD_3OD, δ/ppm, *J*/Hz): 1.98 (s, H-21), 2.42 (2H, t, 7.0, H-17), 1.79 (2H, quint., 7.0, H-16), 2.47 (2H, t, 7.0, H-15), 5.49 (d, 10.8, H-10), 6.04 (dt, 10.8 and 7.6, H-9), 2.36 (2H, m, H-8), 1.46 (2H, m, H-7, H-6), 2.36 (2H, m, H-5), 5.99 (dt, 10.8 and 7.6, H-4), 5.46 (dd, 10.8 and 1.7, H-3) and 3.08 (d, 1.7, H-1). ^{13}C NMR (125 MHz, CD_3OD): 64.9 (d, C-21), 68.2 (s, C-20), 65.6 (s, C-19), 76.9 (s, C-18), 18.2 (t, C-17), 26.8 (t, C-16), 18.8 (t, C-15), 82.9 (s, C-14), 72.5 (s, C-13), 66.2 (s, C-12), 78.0 (s, C-11), 108.31 (d, C-10), 147.7 (d, C-9), 30.5 (t, C-8), 28.2 (t, C-7), 28.1 (t, C-6), 30.0 (t, C-5), 145.7 (d, C-4), 108.24 (d, C-3), 81.3 (s, C-2) and 80.5 (d, C-1). [29]
	Callyspongia sp.<br>Japan	^{1}H NMR (600 MHz, $CDCl_3$, δ/ppm, *J*/Hz): 6.05 (1H, dt, 10.7 and 7.4, H-9), 6.00 (1H, ddt, 10.7, 0.8 and 7.4, H-4), 5.48 (1H, dt, 10.7 and 1.1, H-10), 5.46 (1H, ddt, 10.7, 2.2 and 1.4, H-3), 3.09 (1H, dd, 2.2 and 0.8, H-1), 2.48 (2H, dt, 0.8 and 6.9, H-15), 2.42 (2H, dt, 1.1 and 6.9, H-17), 2.36 (4H, m, H-5, H-8), 1.99 (1H, t, 1.1, H-21), 1.79 (2H, quint, 6.9, H-16) and 1.46 (4H, m, H-6, H-7). ^{13}C NMR (150 MHz, $CDCl_3$): 147.7 (d, C-9), 145.7 (d, C-4), 108.3 (d, C-3), 108.2 (d, C-10), 82.9 (s, C-14, C-19), 81.3 (d, C-1), 80.4 (s, C-2), 77.9 (s, C-12), 76.8 (s, C-18), 72.5 (s, C-11), 68.2 (s, C-20), 66.2 (d, C-21), 65.5 (s, C-13), 30.5 (t, C-8), 30.0 (t, C-5), 28.2 (t, C-7), 28.1 (t, C-6), 26.7 (t, C-16), 18.7 (t, C-15) and 18.2 (t, C-17). [21]
Callyberyne B (**10**)<br>$C_{21}H_{22}$<br>Polyacetylene	*Callyspongia* sp.<br>Japan	^{1}H NMR (600 MHz, $CDCl_3$, δ/ppm, *J*/Hz): 6.03 (1H, dt, 10.7 and 7.4, H-9), 5.988 (1H, ddt, 10.7, 0.8 and 7.4, H-18), 5.994 (1H, ddt, 10.7, 0.8 and 7.4, H-4), 5.50 (1H, dt, 10.7 and 1.2, H-10), 5.48 (1H, ddt, 10.7, 2.2 and 0.8,

		H-3), 5.46 (1H, ddt, 10.7, 2.2 and 0.8, H-19), 3.10 (1H, dd, 2.2 and 0.8, H-21), 3.08 (1H, dd, 2.2 and 0.8, H-1), 2.45 (1H, ddt, 7.4, 1.1 and 7.4, H-17), 2.37 (2H, dt, 0.8 and 7.4, H-15), 2.35 (2H, m, H-5, H-8), 1.69 (2H, quint, 7.4, H-16) and 1.46 (4H, m, H-6, H-7). ^{13}C NMR (150 MHz, $CDCl_3$): 147.5 (d, C-9), 145.8 (d, C-4), 144.3 (d, C-18), 109.3 (d, C-19), 108.33 (d, C-3), 108.28 (d, C-10), 84.2 (s, C-14), 81.8 (d, C-21), 81.3 (d, C-1), 80.5 (s, C-2), 80.2 (s, C-20), 78.2 (s, C-12), 72.2 (s, C-11), 65.6 (s, C-13), 30.5 (t, C-8), 30.0 (t, C-5), 28.4 (t, C-17), 28.22 (t, C-7), 28.15 (t, C-6), 27.5 (t, C-16) and 19.2 (t, C-15). [21]
Callyberyne C (Callytetrayne) (**11**)<br>$C_{21}H_{24}$<br>Polyacetylene	*Callyspongia* sp.<br>Japan	^{1}H NMR (600 MHz, $CDCl_3$, δ/ppm, *J*/Hz): 5.99 (1H, ddt, 10.7, 1.0 and 7.4, H-18), 5.98 (1H, ddt, 10.7, 0.8 and 7.4, H-4), 5.49 (1H, ddt, 10.7, 2.2 and 1.4, H-3), 5.45 (1H, ddt, 10.7, 2.2 and 1.4, H-19), 3.09 (1H, dd, 2.2 and 0.8, H-1), 3.08 (1H, dd, 2.7 and 1.0, H-21), 2.43 (2H, dt, 1.1 and 7.4, H-5), 2.33 (2H, dt, 1.1 and 7.4, H-17), 2.29 (2H, dt, 1.1 and 7.4, H-7), 2.25 (2H, dt, 1.1 and 7.4, H-12), 1.66 (2H, quint, 7.4, H-6), 1.52 (2H, quint, 7.4, H-13), 1.42 (2H, m, H-16), 1.41 (2H, m, H-14) and 1.33 (2H, m, H-15). ^{13}C NMR (150 MHz, $CDCl_3$): 146.0 (d, C-18), 144.4 (d, C-4), 109.2 3 (d, C-3), 108.1 (d, C-19), 81.7 (d, C-1), 81.2 (d, C-21), 80.5 (s, C-20), 80.2 (s, C-2), 77.7 (s, C-11), 77.0 (s, C-8), 65.7 (s, C-9), 65.3 (s, C-10), 30.1 (t, C-17), 29.4 (t, C-5), 28.6 (t, C-15), 28.53 (t, C-14), 28.49 (t, C-16), 28.2 (t, C-13), 27.5 (t, C-6), 19.2 (t, C-12) and 18.8 (t, C-7). [21]
	Callyspongia truncata<br>Japan	^{1}H NMR (500 MHz, CD_3OD, δ/ppm, *J*/Hz): 3.09 (d, 1.9, H-1), 5.48 (dd, 10.8 and 1.9, H-3), 5.96 (m, H-4), 2.42 (2H, m, H-5), 1.67 (2H, m, H-6), 2.29 (2H, m, H-7), 2.24 (2H, m, H-12), 1.53 (2H, m, H-13), 1.39 (2H, m, H-14), 1.32 (2H, m, H-15), 1.42 (2H, m, H-16), 2.33 (2H, m, H-17), 5.99 (m, H-18), 5.44 (dd, 10.8 and 1.9, H-19) and 3.07 (d, 1.9, H-21). ^{13}C NMR (125 MHz, CD_3OD): 8.12 (d, C-1), 80.2 (s, C-2), 109.2 (d, C-3), 144.3 (d, C-4), 29.4 (t, C-5), 27.5 (t, C-6), 18.8 (t, C-7), 76.5 (s, C-8), 65.8 (s, C-9), 65.3 (s, C-10), 77.4 (s, C-11), 19.2 (t, C-12), 28.2 (t, C-13), 28.52 (t, C-14), 28.56 (t, C-15), 28.48 (t, C-16), 30.1 (t, C-17), 145.9 (d, C-18), 108.1 (d, C-19), 80.5 (s, C-20) and 8.17 (d, C-21). [29]
Callydiyne (**12**)<br>$C_{16}H_{22}$<br>Diacetylene	*Callyspongia flammea*<br>Papua, New Guinea	^{1}H NMR (400 MHz, $CDCl_3$, δ/ppm, *J*/Hz): 1.30 (4H, bs), 1.40 (2H, m), 2.32 (2H, dq, 1.4 and 7.4), 3.06 (1H,

		dd, 1.4 and 0.8), 5.44 (1H, ddt, 10.6, 2.3 and 1.4) and 5.99 (1H, ddt, 10.6, 0.8 and 7.5). ^{13}C NMR (75 MHz, CDCl$_3$): 28.7 (CH$_2$), 29.1 (CH$_2$), 29.3 (CH$_2$), 30.2 (CH$_2$), 80.3 (C, C-2, C-15), 81.2 (CH, C-1, C-16), 107.9 (CH, C-3, C-14) and 146.2 (CH, C-4, C-13). [30]
14,15-Dihydrosiphonodiol (Dihydrosiphonodiol) (**13**)<br>$C_{23}H_{26}O_2$<br>Polyacetylene diol	*Callyspongia lindgreni*[1]<br>Japan	^{1}H NMR* (CDCl$_3$, δ/ppm, *J*/Hz): 6.00 (1H, ddt, 10.8, 1.0 and 7.6), 5.45 (1H, dtt, 2.0, 1.4 and 10.8), 4.49 (1H, br dd, 3.3 and 6.9), 3.75 (1H, dt, 11.4 and 3.3), 3.68 (1H, 11.4 and 6.9), 3.07 (1H, br d, 2.0), 2.42 (2H, t, 6.9), 2.37 (2H, t, 6.9), 2.32 (2H, qd, 7.6 and 1.4), 2.25 (2H, t, 7.5), 1.75 (2H, quint., 6.9), 1.55 (2H, m) and 1.3-1.45 (6H, m). ^{13}C NMR (CDCl$_3$): 145.9 (d), 108.2 (d), 81.3 (d), 80.7 (s), 80.5 (s), 78.0 (s), 75.6 (s), 73.4 (s), 71.1 (s), 66.3 (t), 65.1 (s), 65.0 (s), 63.6 (d), 30.1 (t), 28.6 (t), 28.5 (t), 28.2 (t), 26.9 (t), 19.1 (t) and 18.4 (t). [32]
	Callyspongia sp.<br>Japan	Spectroscopic data not provided (Reference 32 is cited). [31]
Callyspongidiol (**14**)<br>$C_{22}H_{26}O_2$<br>Polyacetylene diol	*Callyspongia* sp.<br>Japan	^{1}H NMR (600 MHz, CDCl$_3$, δ/ppm, *J*/Hz): 6.01 (1H, dtd, 11.0, 7.4 and 0.8, H-19), 5.84 (1H, dt, 10.7 and 7.4, H-13), 5.45 (1H, dtt, 11.0, 2.7 and 1.6, H-20), 5.42 (1H, dtt, 10.7, 1.6 and 1.6, H-12), 4.50 (1H, dd, 6.6 and 3.8, H-2), 3.75 (1H, dd, 11.5 and 3.8, H-1), 3.68 (1H, dd, 11.5 and 6.6, H-1), 3.09 (1H, dd, 2.7 and 0.8, H-22), 2.47 (2H, td, 6.6 and 1.6, H-9), 2.44 (2H, t, 6.6, H-7), 2.34 (2H, tdd, 7.4, 7.4 and 1.6, H-18), 2.27 (2H, tdd, 7.4, 7.4 and 1.6, H-14), 1.77 (2H, tt, 6.6 and 6.6, H-8), 1.43 (2H, tt, 6.6 and 6.6, H-17), 1.40 (2H, tt, 6.6 and 6.6, H-15) and 1.34 (2H, m, H-16). ^{13}C NMR (150 MHz, CDCl$_3$): 146.2 (d, C-19), 143.1 (d, C-13), 109.0 (d, C-12), 107.9 (d, C-20), 92.5 (s, C-10), 81.2 (d, C-22), 81.0 (s, C-6), 80.6 (s, C-21), 78.4 (s, C-11), 73.3 (s, C-3), 71.1 (s, C-4), 66.3 (t, C-1), 64.7 (s, C-5), 63.6 (d, C-2), 30.2 (t, C-18), 30.0 (t, C-14), 28.9 (t, C-16), 28.7 (t, C-15), 28.6 (t, C-17), 27.3 (t, C-8), 18.7 (t, C-9) and 18.3 (t, C-7). [31]
	Callyspongia sp.<br>Japan	Spectroscopic data not provided (References 32 and 33 are cited). [31]
Siphonodiol (**15**)<br>$C_{23}H_{24}O_2$<br>Polyacetylene diol	*Callyspongia lindgreni*[1]<br>Japan	^{1}H NMR* (CDCl$_3$, δ/ppm, *J*/Hz): 6.04 (1H, dt, 10.9 and 7.5), 5.98 (1H, ddt, 10.8, 0.7 and 7.6), 5.46 (1H, br d, 10.9), 5.44 (1H, ddt, 10.8, 2.0 and 1.4), 4.48 (1H, br dd, 3.3 and 6.7), 3.74 (1H, dd, 11.4 and 3.3), 3.67 (1H, dd, 11.4 and 6.7), 3.07 (1H, br d, 2.0), 2.45 (2H, t, 6.8), 2.42 (2H, t, 6.8), 2.34 (4H, m), 1.77 (2H, quint., 6.9)

		and 1.44 (4H, m). ^{13}C NMR ($CDCl_3$): 147.7 (d), 145.7 (d), 108.3 (d), 108.1 (d), 83.0 (s), 81.4 (d), 80.5 (s), 78.0 (s), 73.6 (s), 72.5 (s), 70.9 (s), 66.2 (t), 66.1 (s), 65.0 (s), 63.5 (d), 30.4 (t), 29.9 (t), 28.2 (t), 28.1 (t), 26.8 (t), 18.7 (t) and 18.4 (t). [32]
	Callyspongia lindgreni[1]<br>Not reported	^{1}H NMR* (δ/ppm, *J*/Hz): 6.05 (d-t, 11 and 7), 5.99 (d-t, 11 and 7), 5.48 (2H, d-m), 4.50, 3.73, 3.08 (d, 2, -CCH), 2.46 (4H, -CC-CH_2), 2.35 (4H, =C-CH_2), 1.78 (2H, quint., 7) and 1.45 (4H, quint., 7). [33]
	Callyspongia truncata<br>Japan	Spectroscopic data not provided (References 32 and 33 are cited). [29]
(3*S*,18*S*,4*E*,16*E*)-eicosa-1,19-diyne-3,18-diol-4,16-diene (**16**)<br>$C_{20}H_{30}O_2$<br>Polyacetylene diol	*Callyspongia pseudoreticulata*<br>Indonesia	^{1}H NMR (600 MHz, $CDCl_3$, δ/ppm, *J*/Hz): 2.57 (d, 2, H-1, H-20), 4.84 (br d, 6, H-3, H-18), 5.61 (ddt, 15, 6 and 1, H-4, H-17), 5.92 (dtd, 15, 7 and 1, H-5, H-16), 2.07 (dt, 7 and 7, H-6, H-15) and 1.61-1.26 (m, H-7, H-14). ^{13}C NMR (150 MHz, $CDCl_3$): 74.0 (C-1, C-20), 83.4 (C-2, C-19), 62.8 (C-3, C-18), 128.4 (C-4, C-17), 134.6 (C-5, C-16), 31.9 (C-6, C-15), 28.8 (C-7, C-14), 29.1 (C-8, C-13), 29.4 (C-9, C-12) and 29.5 (C-10, C-11). [34]
(+)-(3*S*,4*E*,16*E*,18*S*)-Icosa-4,16-diene-1,19-diyne-3,18-diol (**16a**)<br>$C_{20}H_{30}O_2$<br>Polyacetylene diol	*Callyspongia* sp.<br>Japan	Spectroscopic data not provided. [35]
(-)-(3*R*,4*E*,16*E*,18*R*)-Icosa-4,16-diene-1,19-diyne-3,18-diol (**16b**)<br>$C_{20}H_{30}O_2$<br>Polyacetylene diol	*Callyspongia* sp.<br>Japan	^{1}H NMR (600 MHz, $CDCl_3$, δ/ppm, *J*/Hz): 1.25 (12H, br s, H-8, H-9, H-10, H-11, H-12, H-13), 1.38 (4H, quint., H-7, H-14), 2.05 (4H, q, 7.0, H-6, H-15), 2.54 (2H, d, 2.0, H-1, H-20), 4.82 (2H, d, 6.0, H-3, H-18), 5.61 (2H, dd, 6.0 and 15.5, H-4, H-17) and 5.90 (2H, dt, 8.0 and 15.5, H-5, H-16). [35]
Callyspongendiol (**17**)<br>$C_{30}H_{44}O_2$<br>Polyacetylene diol	*Callyspongia siphonella*<br>Saudi Arabia	^{1}H NMR (600 MHz, $CDCl_3$, δ/ppm, *J*/Hz): 2.57 (d, 1.8, H-1), 4.84 (br d, 5.4, H-3), 5.61 (dd, 15.0 and 6.0, H-4), 5.92 (dt, 15.0, 6.0, H-5), 2.07 (m, H-6), 1.37 (m, H-7), 1.25 (m, H-8, H-9), 1.66 (p, 6.6, H-10), 2.25 (tt, 7.2 and 2.4, H-11), 2.13 (tt, 7.2 and 2.4, H-14), 1.47 (p, 7.2, H-15), 2.13 (tt, 7.2 and 2.4, H-16), 2.25 (tt, 7.2 and 2.4, H-19), 1.65 (p, 6.6, H-20), 1.25 (m, H-21, H-22, H-23, H-24, H-25), 1.47 (p, 7.2, H-26), 1.71 (q, 7.2, H-27), 4.37 (dt, 6.6 and 1.8, H-28) and 2.46 (d, 1.8, H-30). ^{13}C NMR (150 MHz, $CDCl_3$): 74.2 (d, C-1), 83.6 (s, C-2), 63.1 (d, C-3), 128.6 (d, C-4), 134.8 (d, C-5), 32.1 (t, C-6), 29.3 (t, C-7), 29.7 (t, C-8, C-9), 29.2 (t, C-10), 18.2 (t, C-11), 81.0 (s, C-12), 79.5 (s, C-13), 19.0 (t, C-14), 25.2 (t, C-15), 19.0 (t, C-16), 79.5 (s, C-17), 81.0 (s, C-18), 18.2 (t, C-19), 29.2 (t, C-20), 29.7 (t, C-21, C-22, C-23,

		C-24, C-25), 29.6 (t, C-26), 37.9 (t, C-27), 62.6 (d, C-28), 85.2 (s, C-29) and 73.1 (d, C-30). [36]
	Callyspongia siphonella Saudi Arabia	Spectroscopic data not provided (Reference 36 is cited). [8]
Tetrahydrosiphonodiol (**18**) $C_{23}H_{28}O_2$ Polyacetylene diol	*Callyspongia lindgreni*[1] Japan	^{1}H NMR* ($CDCl_3$, δ/ppm, *J*/Hz): 6.00 (1H, ddt, 10.7, 1.0 and 6.8), 5.82 (1H, dt, 10.8 and 7.4), 5.44 (1H, ddt, 10.7, 2.0 and 1.4), 5.42 (1H, dtt, 10.8, 2.0 and 1.4), 4.49 (1H, br dd, 3.8 and 6.2), 3.75 (1H, 11.7 and 3.8), 3.69 (1H, dd, 11.7 and 6.2), 3.07 (1H, br d, 2.0), 2.46 (2H, dt, 2.0 and 6.9), 2.44 (2H, t, 6.9), 2.33 (2H, qd, 6.4 and 1.4), 2.27 (2H, qd, 6.8 and 1.4), 1.77 (2H, quint., 6.9) and 1.3-1.45 (8H, br m). ^{13}C NMR ($CDCl_3$): 146.1 (d), 143.0 (d), 108.9 (d), 107.9 (d), 81.0 (d), 79.5 (s), 73.1 (s), 71.0 (s), 66.2 (t), 63.5 (d), 30.1 (t), 30.0 (t), 28.8 (t), 28.7 (t), 27.3 (t), 26.9 (t), 18.6 (t) and 18.3 (t). [32]
(3*R*,4*E*,28*Z*)-Hentriacont-4,28-diene-1,23,30-triyn-3-ol[2] (**19**) $C_{31}H_{48}O$ Polyacetylene alcohol	*Callyspongia* sp. Indonesia	^{1}H NMR (500 MHz, $CDCl_3$, δ/ ppm, *J*/Hz): 2.57 (d, 2.4, H-1), 4.84 (br s, H-3), 1.83 (br d, 6.0, OH), 5.61 (ddt, 15.2, 6.1 and 1.4, H-4), 5.92 (br dt, 15.2 and 6.7, H-5), 2.06 (br q, 7.0, H-6), 1.38 (m, H-7), 1.27 (br s, H-8, H-9, H-10, H-11, H-12, H-13, H-14, H-15, H-16, H-17, H-18, H-19), 1.35 (m, H-20), 1.48 (quint., 7.3, H-21), 2.13 (tt, 7.3 and 2.1, H-22), 2.18 (tt, 7.3 and 2.1, H-25), 1.61 (quint., 7.3, H-26), 2.42 (br q, 7.3, H-27), 6.01 (dt, 11.0 and 7.3, H-28), 5.48 (br d, 11.0, H-29) and 3.07 (d, 2.1, H-31). ^{13}C NMR (125 MHz, $CDCl_3$): 73.9 (C-1), 83.3 (C-2), 62.8 (C-3), 128.3 (C-4), 134.8 (C-5), 31.9 (C-6), 28.8 (C-7), 29.1 (C-8), 29.4-7 (C-9, C-10, C-11, C-12, C-13, C-14, C-15, C-16, C-17, C-18, C-19), 28.9 (C-20), 29.2 (C-21), 18.7 (C-22), 80.8 (C-23), 79.5 (C-24), 18.4 (C-25), 28.3 (C-26), 29.7 (C-27), 145.2 (C-28), 108.6 (C-29), 80.3 (C-30) and 81.4 (C-31). [37]
Callyspongenol A (**20**) $C_{22}H_{28}O$ Polyacetylene alcohol	*Callyspongia* sp. Egypt	^{1}H NMR (600 MHz, $CDCl_3$, δ/ppm, *J*/Hz): 4.21 (t, 2.0, H-1), 2.18 (m, H-4), 1.49 (m, H-5), 2.22 (t, 7.0, H-6), 1.47 (m, H-9), 1.37 (m, H-10, H-11), 1.49 (m, H-12), 2.02 (q, 7.0, H-15), 1.29 (m, H-16), 1.37 (m, H-17), 2.29 (q, 7.3, H-18), 5.99 (dt, 7.3 and 10.7, H-19), 5.41 (br d, 10.7, H-20) and 3.04 (br d, 2.0, H-22). ^{13}C NMR (150 MHz, $CDCl_3$): 51.2 (t, C-1), 78.6 (s, C-2), 86.0 (s, C-3), 18.5 (t, C-4), 28.2 (t, C-5), 19.0 (t, C-6), 77.5 (s, C-7), 65.4 (s, C-8), 27.9 (t, C-9), 27.2 (t, C-10), 28.1 (t, C-11), 27.7 (t, C-12), 65.1 (s, C-13), 77.1 (s, C-14), 19.1 (t, C-15), 28.4 (t, C-16, C-17), 30.0 (t, C-18), 145.9 (d, C-19), 108.0 (d, C-20), 80.4 (s, C-21) and 81.2 (d, C-22). [38]

Callyspongenol B (**21**)<br>$C_{22}H_{26}O$<br>Polyacetylene alcohol	*Callyspongia* sp.<br>Egypt	^{1}H NMR (600 MHz, $CDCl_3$, δ/ppm, *J*/Hz): 4.41 (br s, H-1), 5.51 (br d, 10.8, H-4), 5.87 (td, 10.8 and 7.3, H-5), 2.41 (q, 7.3, H-6), 1.62 (quint., 7.0, H-7), 2.27 (t, 7.0, H-8), 2.23 (t, 7.0, H-13), 1.50 (quint., 7.0, H-14), 1.41 (m, H-15), 1.31 (quint., 7.0, H-16), 1.36 (m, H-17), 2.31 (q, 7.3, H-18), 5.97 (dt, 7.3 and 10.8, H-19), 5.30 (br d, 10.8, H-20) and 3.06 (br s, H-22). ^{13}C NMR (150 MHz, $CDCl_3$): 51.6 (t, C-1), 91.9 (s, C-2), 82.1 (s, C-3), 109.6 (d, C-4), 142.8 (d, C-5), 29.1 (t, C-6), 27.4 (t, C-7), 18.6 (t, C-8), 77.1 (s, C-9), 65.6 (s, C-10), 65.2 (s, C-11), 77.8 (s, C-12), 19.1 (t, C-13), 28.1 (t, C-14), 28.4 (t, C-15), 28.55 (t, C-16), 28.49 (t, C-17), 30.1 (t, C-18), 145.9 (d, C-19), 108.0 (d, C-20), 80.5 (s, C-21) and 81.1 (d, C-22). [38]
Callyspongenol C (**22**)<br>$C_{22}H_{24}O$<br>Polyacetylene alcohol	*Callyspongia* sp.<br>Egypt	^{1}H NMR (600 MHz, $CDCl_3$, δ/ppm, *J*/Hz): 4.40 (br s, H-1), 5.51 (dd, 10.8 and 1.5, H-4), 5.86 (dt, 7.7 and 10.8, H-5), 2.40 (q, 7.3, H-6), 1.65 (quint., 7.3, H-7), 2.35 (m, H-8), 5.45 (br d, 11.5, H-13), 5.99 (td, 11.5 and 7.3, H-14), 2.32 (m, H-15), 1.42 (m, H-16, H-17), 2.34 (m, H-18), 5.96 (dt, 7.3 and 11.5, H-19), 5.41 (br d, 11.5, H-20) and 3.06 (br s, H-22). ^{13}C NMR (150 MHz, $CDCl_3$): 51.5 (t, C-1), 91.9 (s, C-2), 82.7 (s, C-3), 109.7 (d, C-4), 142.7 (d, C-5), 29.1 (t, C-6), 27.3 (t, C-7), 19.0 (t, C-8), 84.4 (s, C-9), 72.2 (s, C-10), 65.5 (s, C-11), 78.7 (s, C-12), 108.1 (d, C-13), 147.6 (d, C-14), 29.9 (t, C-15), 28.1 (t, C-16, C-17), 29.9 (t, C-18), 145.7 (d, C-19), 108.2 (d, C-20), 80.4 (s, C-21) and 81.3 (d, C-22). [38]
	Callyspongia siphonella<br>Saudi Arabia	Spectroscopic data not provided (Reference 36 is cited). [8]
Callyspongenol D (**23**)<br>$C_{20}H_{24}O$<br>Polyacetylene alcohol	*Callyspongia siphonella*<br>Saudi Arabia	^{1}H NMR (600 MHz, $CDCl_3$, δ/ ppm, *J*/Hz): 4.29 (dt, 6.0 and 1.8, H-1), 2.26 (tt, 6.6 and 1.8, H-4), 1.25 (m, H-5), 1.49-1.62 (m, H-6), 1.25 (m, H-7), 2.33 (t, 7.2, H-8), 2.35 (t, 7.2, H-13), 1.49-1.62 (m, H-14), 1.25 (m, H-15), 2.38 (q, 7.8, H-16), 6.01 (dt, 10.8 and 7.8, H-17), 5.50 (dd, 10.8 and 1.8, H-18) and 3.11 (d, 1.8, H-20). ^{13}C NMR (150 MHz, $CDCl_3$): 51.6 (t, C-1), 78.8 (s, C-2), 86.3 (s, C-3), 18.7 (t, C-4), 27.8 (t, C-5), 27.7 (t, C-6), 27.5 (t, C-7), 19.3 (t, C-8), 79.2 (s, C-9), 66.1 (s, C-10), 66.0 (s, C-11), 79.2 (s, C-12), 19.4 (t, C-13), 28.1 (t, C-14, C-15), 29.6 (t, C-16), 145.2 (d, C-17), 108.7 (d, C-18), 80.5 (s, C-19) and 81.7 (d, C-20). [36]
Callysponyne A (**24**)<br>$C_{31}H_{38}O$<br>Polyacetylene alcohol	*Callyspongia* sp.<br>Taiwan	^{1}H NMR (500 MHz, $CDCl_3$, δ/ ppm, *J*/Hz): 1.95 (t, 2.5, H-31), 2.19 (dt, 7.0 and 2.5, H-29), 1.55-1.49 (m, H-26,

		H-27, H-28), 2.15 (m, H-25), 2.24 (m, H-22), 1.64 (tt, 7.0 and 7.0, H-21), 2.24 (m, H-12, H-17), 1.69 (tt, 7.0 and 6.5, H-16), 2.36 (t, 7.0, H-15), 2.24 (m, H-10), 1.55-1.49 (m, H-9), 1.41 (m, H-7, H-8), 2.08 (m, H-6), 5.90 (dt, 15.0 and 7.0, H-5), 5.62 (dd, 15.0 and 6.0, H-4), 4.83 (d, 5.5, H-3) and 2.57 (d, 2.0, H-1). ^{13}C NMR (125 MHz, $CDCl_3$): 68.2 (C-31), 84.5 (C-30), 18.3 (C-29), 28.5-27.9 (C-26, C-27, C-28), 17.9 (C-25), 80.5 (C-24), 79.4 (C-23), 18.6 (C-22), 28.5 (C-21), 19.0 (C-20), 80.3 (C-19), 79.2 (C-18), 17.9 (C-17), 27.8 (C-16), 18.3 (C-15), 76.6 (C-14), 65.7 (C-13), 65.3 (C-12), 77.5 (C-11), 17.9 (C-10), 28.5-27.9 (C-8, C-9), 28.5-27.8 (C-7), 31.7 (C-6), 134.1 (C-5), 128.6 (C-4), 62.7 (C-3), 83.2 (C-2) and 74.0 (C-1). [39]
Callysponyne B (**25**)<br>$C_{31}H_{36}O$<br>Polyacetylene alcohol	*Callyspongia* sp.<br>Taiwan	^{1}H NMR (500 MHz, $CDCl_3$, δ/ppm, *J*/Hz): 3.10 (s, H-31), 5.48 (d, 10.5, H-29), 6.00 (dd, 10.5 and 7.5, H-28), 2.43 (dd, 7.5 and 7.5, H-27), 1.62 (m, H-26), 2.19 (m, H-25), 2.25 (m, H-22), 1.65 (m, H-21), 2.25 (m, H-17, H-20), 1.69 (m, H-16), 2.37 (t, 7.0, H-15), 2.24 (m, H-10), 1.53 (m, H-9), 1.41 (m, H-7, H-8), 2.08 (m, H-6), 5.92 (dt, 15.0 and 6.5, H-5), 5.62 (dd, 15.0 and 5.5, H-4), 4.84 (d, 5.5, H-3) and 2.57 (s, H-1). ^{13}C NMR (125 MHz, $CDCl_3$): 81.5 (C-31), 80.3 (C-30), 108.7 (C-29), 145.2 (C-28), 29.5 (C-27), 28.1 (C-26), 18.4-17.9 (C-25), 80.3 (C-24), 79.7 (C-23), 18.4-17.9 (C-22), 28.5 (C-21), 19.1 (C-20), 80.2 (C-19), 79.3 (C-18), 18.4-17.9 (C-17), 27.8 (C-16), 18.4-17.9 (C-15), 76.6 (C-14), 65.7 (C-13), 65.3 (C-12), 77.5 (C-11), 18.4-17.9 (C-10), 28.2 (C-7, C-8, C-9), 31.7 (C-6), 134.1 (C-5), 128.6 (C-4), 62.7 (C-3), 83.3 (C-2) and 74.0 (C-1). [39]
Dehydroisophonochalynol<br>(Dehydrosiphonochalynol) (**26**)<br>$C_{22}H_{22}O$<br>Polyacetylene alcohol	*Callyspongia sp.*<br>Egypt	^{1}H NMR (600 MHz, $CDCl_3$, δ/ppm, *J*/Hz): 4.40 (br s, H-1), 5.51 (br d, 10.4, H-4), 5.85 (dt, 7.3 and 10.4, H-5), 2.38 (m, H-6), 1.62 (quint., 7.3, H-7), 2.27 (m, H-8), 2.29 (m, H-15), 1.50 (m, H-16), 1.51 (m, H-17), 2.31 (m, H-18), 5.99 (dt, 7.3 and 10.4, H-19), 5.44 (br d, 10.4, H-20) and 3.06 (br s, H-22). ^{13}C NMR (125 MHz, $CDCl_3$): 51.5 (t, C-1), 92.0 (s, C-2), 81.9 (s, C-3), 109.9 (d, C-4), 142.4 (d, C-5), 29.1 (t, C-6), 27.1 (t, C-7), 19.1 (t, C-8), 79.8 (s, C-9), 65.7 (s, C-10), 60.3 (s, C-11), 60.5 (s, C-12), 66.0 (s, C-13), 79.1 (s, C-14), 19.8 (t, C-15), 27.3 (t, C-16), 27.6 (t, C-17), 29.5 (t, C-18), 145.1 (d, C-19), 108.6 (d, C-20), 80.3 (s, C-21) and 81.5 (d, C-22). [38]

	Callyspongia siphonella Egypt	^{1}H NMR (400 MHz, $CDCl_3$, δ/ppm, *J*/Hz): 4.40 (d, 2.0, H-1), 5.48 (d, 11.0, H-4), 5.83 (dt, 11.0 and 7.3, H-5), 2.35 (m, H-6), 1.60 (quint, 7.3, H-7), 2.27 (m, H-8, H-15), 1.50 (m, H-16), 1.51 (m, H-17), 2.28 (m, H-18), 5.94 (dt, 10.9 and 7.8, H-19), 5.42 (d, 10.9, H-20) and 3.04 (d, 1.8, H-22). ^{13}C NMR (100 MHz, $CDCl_3$): 51.4 (C-1), 92.0 (C-2), 81.8 (C-3), 109.8 (C-4), 142.4 (C-5), 29.0 (C-6), 27.1 (C-7), 18.7 (C-8), 79.1 (C-9), 65.7 (C-10), 60.2 (C-11), 60.4 (C-12), 65.9 (C-13), 78.8 (C-14), 19.0 (C-15), 27.3 (C-16), 27.6 (C-17), 29.4 (C-18), 145.0 (C-19), 108.5 (C-20), 80.2 (C-21) and 81.5 (C-22). [40]
	Callyspongia siphonella Saudi Arabia	^{1}H NMR (90 MHz, $CDCl_3$, δ/ppm): 1.55 (6H, m), 2.3 (9H, m), 3.08 (d, 2, =CH-CCH), 4.43 (d, 2, CH_2OH), 5.52 (2H, m) and 5.90 (2H, m). [36,47[3]]
Siphonellanol A (**27**) $C_{22}H_{22}O$ Polyacetylene alcohol	*Callyspongia siphonella* Egypt	^{1}H NMR (400 MHz, $CDCl_3$, δ/ppm, *J*/Hz): 4.37 (br s, H-1), 5.54 (d, 15.8, H-4), 6.11 (dt, 15.8 and 7.8, H-5), 2.24 (m, H-6), 1.64 (m, H-7), 2.31 (m, H-8, H-15), 1.54 (m, H-16), 1.52 (m, H-17), 2.33 (m, H-18), 5.98 (dt, 10.8 and 7.4, H-19), 5.48 (d, 10.8, H-20) and 3.09 (d, 1.8, H-22). ^{13}C NMR (100 MHz, $CDCl_3$): 51.7 (C-1), 86.4 (C-2), 84.2 (C-3), 110.2 (C-4), 143.6 (C-5), 31.9 (C-6), 27.2 (C-7), 18.7 (C-8), 79.2 (C-9), 66.0 (C-10), 60.0 (C-11), 60.3 (C-12), 66.5 (C-13), 78.4 (C-14), 19.4 (C-15), 27.5 (C-16), 27.8 (C-17), 29.6 (C-18), 145.3 (C-19), 108.9 (C-20), 80.5 (C-21) and 81.8 (C-22). [40]
Siphonellanol B (**28**) $C_{22}H_{26}O$ Polyacetylene alcohol	*Callyspongia siphonella* Egypt	^{1}H NMR (500 MHz, $CDCl_3$, δ/ppm, *J*/Hz): 3.67 (t, 6.5, H-1), 1.61 (m, H-2), 2.32 (m, H-3), 5.34 (dt, 11.0 and 7.3, H-4), 5.44 (dt, 11.0 and 7.2, H-5), 2.13 (m, H-6), 1.56 (m, H-7), 2.30 (m, H-8, H-15), 1.54 (m, H-16), 1.52 (m, H-17), 2.37 (m, H-18), 5.98 (dt, 10.8, H-19), 5.48 (d, 10.8, H-20) and 3.09 (d, 1.8, H-22). ^{13}C NMR (100 MHz, $CDCl_3$): 62.6 (C-1), 32.6 (C-2), 29.7 (C-3), 130.5 (C-4), 128.8 (C-5), 26.0 (C-6), 27.4 (C-7), 18.8 (C-8), 72.9 (C-9), 65.9 (C-10), 60.4 (C-11, C-12), 65.9 (C-13), 79.0 (C-14), 19.2 (C-15), 27.9 (C-16), 27.6 (C-17), 29.5 (C-18), 145.2 (C-19), 108.7 (C-20), 80.3 (C-21) and 81.5 (C-22). [40]
Siphonellanol C (**29**) $C_{22}H_{24}O$ Polyacetylene alcohol	*Callyspongia siphonella* Egypt	^{1}H NMR (400 MHz, $CDCl_3$, δ/ ppm, *J*/Hz): 3.69 (t, 6.4, H-1), 2.45 (q, 6.4, H-2), 5.38 (dt, 10.7 and 7.7, H-3), 6.12 (t, 10.7, H-4), 6.41 (dd, 15.0 and 10.7, H-5), 5.71 (dt, 15.0 and 7.7, H-6), 2.39 (m, H-7), 2.36 (m, H-8, H-15), 1.60 (m, H-16, H-17), 2.37 (m, H-18), 5.98 (dt, 11.0 and 7.5, H-19), 5.42 (d, 11.0, H-20) and 3.09 (d, 2.3, H-

		22). ^{13}C NMR (100 MHz, $CDCl_3$): 62.2 (C-1), 31.2 (C-2), 127.0 (C-3), 132.4 (C-4), 126.3 (C-5), 131.2 (C-6), 31.4 (C-7), 19.7 (C-8), 73.0 (C-9), 65.8 (C-10), 60.6 (C-11), 60.2 (C-12), 65.8 (C-13), 79.2 (C-14), 19.2 (C-15), 27.7 (C-16), 27.4 (C-17), 29.5 (C-18), 145.2 (C-19), 108.7 (C-20), 80.3 (C-21) and 81.5 (C-22). [40]
Siphonochalynol (**30**)<br>$C_{22}H_{24}O$<br>Polyacetylene alcohol	*Callyspongia siphonella*<br>Egypt	^1H NMR (270 MHz, $CDCl_3$, δ/ppm, *J*/Hz): 1.50 (10H, m), 2.30 (8H, m), 3.09 (d, 2.3, =CH-CCH), 4.25 (t, 2.2, -CH_2-CC-CH_2OH), 5.47 (1H, dd, 10.9 and 2.3) and 5.96 (1H, dt, 10.9 and 6.7). [40,47[3]]
Callysponginol sulfate A (**31**)<br>$C_{24}H_{41}O_6SNa$<br>Acetylenic sulfate fatty acid	*Callyspongia truncata*<br>Japan	^1H NMR (600 MHz, CD_3OD, δ/ppm, *J*/Hz): 5.16 (t, 2.3, H-2), 2.18 (dt, 2.3 and 7.3, H-5), 1.48 (quint., 7.3, H-6), 1.31 (H-7), /1.25 (H-8, H-9, H-10, H-11, H-12, H-13, H-14), 1.30 (H-15), 2.00 (q, 6.4, H-16), 5.32 (dd, 4.6 and 5.8, H-17, H-18), 2.00 (q, 6.4, H-19), 1.30 (H-20), 1.25 (H-21, H-22), 1.29 (H-23) and 0.87 (t, 7.1, H-24). ^{13}C NMR (150 MHz, CD_3OD): 173.30 (C-1), 70.01 (C-2), 76.42 (C-3), 87.37 (C-4), 19.47 (C-5), 29.99 (C-6), 30.78 (C-7, C-8, C-9, C-10, C-11, C-12, C-13, C-14, C-15), 28.11 (C-16), 130.78 (C-17, C-18), 28.11 (C-19), 30.78 (C-20, C-21, C-22), 23.69 (C-23) and 14.50 (C-24). [41]
Callyspongin A<br>(Siphonodiol disulfate) (**32**)<br>$C_{23}H_{22}Na_2O_8S_2$<br>Polyacetylene sulfate	*Callyspongia truncata*<br>Japan	^1H NMR (500 MHz, CD_3OD/D_2O, δ/ppm, *J*/Hz): 4.14 (2H, d, 5.5, H-1), 5.20 (t, 5.5, H-2), 2.44 (2H, t, 6.9, H-7), 1.75 (2H, quint., 6.9, H-8), 2.45 (2H, t, 6.9, H-9), 5.52 (d, 11.0, H-14), 6.14 (dt, 11.0 and 7.3, H-15), 2.31 (2H, m, H-16), 1.44 (2H, m, H-17, H-18), 2.32 (2H, m, H-19), 6.04 (dt, 11.0 and 7.8, H-20), 5.48 (br d, 11.0, H-21) and 3.46 (br s, H-23). ^{13}C NMR (125 MHz, CD_3OD/D_2O): 70.2 (t, C-1), 68.6 (d, C-2), 72.0 (s, C-3), 73.3 (s, C-4), 66.3 (s, C-5), 83.2 (s, C-6), 19.5 (t, C-7), 28.3 (t, C-8), 19.8 (t, C-9), 85.3 (s, C-10), 67.3 (s, C-11), 79.4 (s, C-12), 74.2 (s, C-13), 109.6 (d, C-14), 150.0 (d, C-15), 31.7 (t, C-16), 29.5 (t, C-17), 29.4 (t, C-18), 31.3 (t, C-19), 147.6 (d, C-20), 109.8 (d, C-21), 82.3 (s, C-22) and 83.8 (d, C-23). [29,42[3]]
Callyspongin B<br>(Siphonodiol sulfate) (**33**)<br>$C_{23}H_{23}NaO_5S$<br>Polyacetylene sulfate	*Callyspongia truncata*<br>Japan	^1H NMR (500 MHz, DMSO-d_6, δ/ppm, *J*/Hz): 3.68 (2H, m, H-1), 4.43 (m, H-2), 2.38 (2H, t, 6.9, H-7), 1.67 (2H, quint., 6.9, H-8), 2.42 (2H, t, 6.9, H-9), 5.56 (d, 11.0, H-14), 6.12 (dt, 11.0 and 7.8, H-15), 2.22 (2H, m, H-16), 1.35 (2H, m, H-17, H-18), 2.23 (2H, m, H-19), 5.99 (dt, 11.0 and 7.8, H-20), 5.46 (dd, 11.0 and 1.5, H-21), 3.95 (d, 1.5, H-23) and 5.68 (br s, 2-OH). ^{13}C NMR

		(125 MHz, DMSO-d$_6$): 69.1 (t, C-1), 60.5 (d, C-2), 77.1 (s, C-3), 68.6 (s, C-4), 65.2 (s, C-5), 80.5 (s, C-6), 17.7 (t, C-7), 26.4 (t, C-8), 18.0 (t, C-9), 84.5 (s, C-10), 65.5 (s, C-11), 78.0 (s, C-12), 72.5 (s, C-13), 108.0 (d, C-14), 148.3 (d, C-15), 30.0 (t, C-16), 27.6 (t, C-17, C-18), 29.5 (t, C-19), 145.2 (d, C-20), 108.6 (d, C-21), 80.2 (s, C-22) and 84.6 (d, C-23). [29,42^{3}]
Callytriol A (**34**)<br>$C_{23}H_{24}O_3$<br>Polyacetylene triol	*Callyspongia truncata*<br>Japan	^{1}H NMR (500 MHz, CD$_3$OD, δ/ppm, *J*/Hz): 3.54 (dd, 11.2 and 6.8, H-1), 3.56 (dd, 11.2 and 5.1, H-1), 4.33 (dd, 6.8 and 5.1, H-2), 2.445 (m, H-7), 2.455 (m, H-7), 1.86 (m, H-8), 1.88 (m, H-8), 4.49 (t, 6.5, H-9), 5.54 (d, 10.7, H-14), 6.15 (dt, 10.7 and 7.8, H-15), 2.34 (m, H-16), 1.46 (m, H-17, H-18), 2.34 (m, H-19), 5.99 (dt, 10.8 and 7.4, H-20), 5.46 (dd, 10.8 and 2.0, H-21) and 3.40 (d, 2.0, H-23). ^{13}C NMR (125 MHz, CD$_3$OD): 16.0 (t, C-7), 29.2 (2C, t, C-17 and C-18), 30.8 (t, C-19), 31.4 (t, C-16), 37.2 (t, C-8), 61.8 (d, C-9), 64.5 (d, C-2), 66.0 (s, C-5), 67.1 (t, C-1), 69.0 (s, C-11), 70.5 (s, C-4), 76.3 (s, C-3), 76.4 (s, C-12 or C-13), 77.9 (s, C-13 or C-12), 80.3 (s, C-6), 81.2 (s, C-22), 82.9 (d, C-23), 84.3 (s, C-10), 108.9 (d, C-14), 109.6 (d, C-21), 146.0 (d, C-20), and 149.5 (d, C-15). [29]
Callytriol B (**35**)<br>$C_{23}H_{24}O_3$<br>Polyacetylene triol	*Callyspongia truncata*<br>Japan	^{1}H NMR (500 MHz, CD$_3$OD, δ/ppm, *J*/Hz): 3.54 (dd, 11.2 and 6.8, H-1), 3.57 (dd, 11.2 and 5.1, H-1), 4.34 (dd, 6.8 and 5.1, H-2), 2.42 (t, 7.0, H-7), 1.74 (quint., 7.0, H-8), 2.46 (t, 7.0, H-9), 5.57 (d, 10.3, H-14), 6.02 (m, H-15), 4.55 (m, H-16), 1.49 (m, H-17, H-18), 2.36 (m, H-19), 5.99 (m, H-20), 5.47 (dd, 10.3 and 2.0, H-21) and 3.40 (d, 2.0, H-23). ^{13}C NMR (125 MHz, CD$_3$OD): 18.9 (t), 19.2 (t), 25.5 (t), 28.1 (t), 30.9 (t), 37.3 (t), 64.5 (d), 66.3 (s), 66.5 (s), 67.1 (t), 70.5 (s), 70.6 (d), 72.2 (s), 76.3 (s), 80.3 (s), 81.2 (s), 82.9 (d), 84.9 (s), 109.1 (d), 109.7 (d), 146.0 (d) and 150.6 (d). [29]
Callytriol C (**36**)<br>$C_{23}H_{24}O_3$<br>Polyacetylene triol	*Callyspongia truncata*<br>Japan	^{1}H NMR (500 MHz, CD$_3$OD, δ/ ppm, *J*/Hz): 3.54 (dd, 11.2 and 6.6, H-1), 3.57 (dd, 11.2 and 5.1, H-1), 4.33 (dd, 6.6 and 5.1, H-2), 2.42 (t, 7.0, H-7), 1.74 (quint., 7.0, H-8), 2.46 (t, 7.0, H-9), 5.51 (d, 10.8, H-14), 6.09 (dt, 10.8 and 7.4, H-15), 2.35 (m, H-16), 1.50 (m, H-17), 1.61 (m, H-17), 1.50 (m, H-18), 1.62 (m, H-18), 4.62 (m, H-19), 5.92 (dd, 11.0 and 8.8, H-20), 5.53 (dd, 11.0 and 2.0, H-21) and 3.50 (d, 2.0, H-23). ^{13}C NMR (125 MHz, CD$_3$OD): 18.9 (t), 19.2 (t), 25.5 (t), 28.2 (t), 31.5 (t), 37.2 (t), 64.5 (d), 66.2 (s), 66.7 (s), 67.1 (t), 70.4

		(d), 70.5 (s), 73.0 (s), 76.3 (s), 79.0 (s), 80.4 (s), 84.0 (d), 109.3 (d), 109.5 (d), 148.40 (d) and 148.44 (d). [29]
Callytriol D (**37**)<br>$C_{23}H_{24}O_3$<br>Polyacetylene triol	*Callyspongia truncata*<br>Japan	^{1}H NMR (500 MHz, CD_3OD, δ/ppm, *J*/Hz): 3.55 (dd, 11.2 and 6.7, H-1), 3.57 (dd, 11.2 and 5.1, H-1), 4.33 (dd, 6.7 and 5.1, H-2), 2.41 (t, 7.0, H-7), 1.73 (quint., 7.0, H-8), 2.44 (t, 7.0, H-9), 5.72 (d, 16.0, H-14), 6.24 (dd, 16.0 and 5.7, H-15), 4.11 (m, H-16), 1.51 (m, H-17, H-18), 2.34 (m, H-19), 5.99 (dt, 10.4 and 7.3, H-20), 5.46 (dd, 10.4 and 1.7) and 3.41 (d, 1.7, H-23). ^{13}C NMR (125 MHz, CD_3OD): 18.87 (t), 18.93 (t), 25.6 (t), 28.2 (t), 31.3 (t), 37.2 (t), 64.5 (d), 66.1 (s), 66.2 (s), 66.6 (s), 67.1 (d), 70.4 (s), 70.5 (s), 72.2 (t), 73.0 (s), 76.3 (s), 78.8 (s), 80.4 (s), 84.1 (d), 109.4 (d), 109.5 (d), 148.4 (d) and 148.8 (d). [29]
Callytriol E (**38**)<br>$C_{23}H_{24}O_3$<br>Polyacetylene triol	*Callyspongia truncata*<br>Japan	^{1}H NMR (500 MHz, CD_3OD, δ/ppm, *J*/Hz): 3.55 (dd, 11.2 and 6.9, H-1), 3.57 (dd, 11.2 and 5.1, H-1), 4.34 (dd, 6.9 and 5.1, H-2), 2.42 (t, 7.0, H-7), 1.74 (quint., 7.0, H-8), 2.46 (t, 7.0, H-9), 5.51 (d, 10.1, H-14), 6.09 (dt, 10.1 and 7.3, H-15), 2.35 (m, H-16), 1.44 (m, H-17), 1.51 (m, H-17, H-18), 4.11 (m, H-19), 6.17 (dd, 15.7 and 5.5, H-20), 5.67 (dd, 15.7 and 2.2, H-21) and 3.20 (d, 2.2, H-23). ^{13}C NMR (125 MHz, CD_3OD): 18.9 (t), 19.1 (t), 25.6 (t), 28.2 (t), 30.9 (t), 37.2 (t), 64.5 (d), 66.2 (s), 66.7 (s), 67.1 (d), 70.5 (s), 72.2 (t), 74.4 (s), 75.1 (s), 76.3 (s), 80.4 (s), 81.2 (s), 82.9 (d), 83.3 (s), 109.0 (d), 109.7 (d), 145.9 (d) and 150.7 (d). [29]
Callyspongyne A (**39**)<br>$C_{33}H_{52}O$<br>Polyacetylenic Lipid	*Callyspongia* sp.<br>Australia	^{1}H NMR (400 MHz, $CDCl_3$, δ/ppm, *J*/Hz): 2.56 (d, 2.2, H-1), 4.84 (dd, 6.4 and 2.2, H-3), 5.60 (dd, 15.1 and 6.4, H-4), 5.90 (dt, 15.1 and 6.8, H-5), 2.06 (m, H-6), 1.29-1.40 (m, H-7, H-8, H-9, H-10, H-11, H-12, H-13, H-14), 2.15 (t, 6.8, H-15), 2.14 (t, 6.8, H-18), 1.29-1.40 (m, H-19, H-20, H-21, H-22, H-23, H-24, H-25, H-26, H-27, H-28), 2.32 (dt, 7.5 and 7.5, H-29), 5.99 (ddt, 10.6, 0.8 and 7.5, H-30), 5.44 (dd, 10.6 and 1.4, H-31) and 3.06 (dd, 1.4 and 0.8, H-33). ^{13}C NMR ($CDCl_3$): 74.0 (C-1), 83.3 (C-2), 62.8 (C-3), 128.3 (C-4), 134.7 (C-5), 31.9 (C-6), 29.6-22.7 (C-7, C-8, C-9, C-10, C-11, C-12, C-13, C-14, C-19, C-20, C-21, C-22, C-23, C-24, C-25, C-26, C-27, C-28), 18.7 (C-15, C-18), 80.2 (C-16, C-17), 30.2 (C-29), 146.2 (C-30), 108.0 (C-31), 81.1 (C-32) and 80.3 (C-33). [43]
Callyspongyne B (**40**)<br>$C_{35}H_{56}O$<br>Polyacetylenic Lipid	*Callyspongia* sp.<br>Australia	
(6Z,9Z,12Z,15Z)-1,6,9,12,15-octadecapenten-3-one (**41**)<br>$C_{18}H_{26}O$	*Callyspongia* sp.<br>Australia	^{1}H NMR (300 MHz, $CDCl_3$, δ/ppm, *J*/Hz): 6.36 (dd, 11.0 and 17.6, H-2), 6.22 (dd, 1.3 and 17.6, H-1_{trans}),

Lipid		5.83 (dd, 1.3 and 11.0, H-1cis), 5.38 (bm, H-6, H-7, H-9, H-10, H-12, H-13, H-15, H-16), 2.83 (bm, $(H\text{-}8)_2$, $(H\text{-}11)_2$, $(H\text{-}14)_2$), 2.65 (t, 7.3, $(H\text{-}4)_2$), 2.39 (dt, 6.9 and 7.3, $(H\text{-}5)_2$), 2.07 (dq, 6.9 and 7.3, $(H\text{-}17)_2$) and 0.97 (t, 7.3, $(H\text{-}18)_3$). ^{13}C NMR (75 MHz, $CDCl_3$): 200.1 (s, C-3), 136.5 (d, C-2), 132.0 (d, C-16), 129.0 (d, C-6), 128.6 (d, C-15), 128.3 (2d, C-9, C-10), 128.1 (t, C-1), 128.0 (d, C-7), 127.8 (d, C-12), 126.9 (d, C-13), 39.4 (t, C-4), 25.6 (2d, C-8, C-11), 25.5 (t, C-14), 21.7 (t, C-5), 20.6 (t, C-17) and 14.3 (q, C-18). [17]
(4Z,7Z,10Z,13Z)-4,7,10,13-hexadecatetraenoic acid (**42**)<br>$C_{16}H_{24}O_2$<br>Fatty Acid	*Callyspongia* sp.<br>Australia	Spectroscopic data not provided. [17]
Petroselenic acid (**43**)<br>$C_{18}H_{34}O_2$<br>Fatty Acid	*Callyspongia siphonella*<br>Egypt	^{13}C NMR (100 MHz, $CDCl_3$): 183.10 (C-1), 130.94 (C-7), 130.88 (C-6), 39.31 (C-2), 33.20 (C-16), 31.14-30.50 (C-9, C-10, C-11, C-12, C-13, C-14, C-15), 28.27 (C-5), 27.64 (C-3), 23.87 (C-17) and 14.57 (C-18). [7]<br>^{1}H NMR (400 MHz, $CDCl_3$, δ/ppm, *J*/Hz): 0.88 (3H, t, 6.8, H-18), 1.26-1.36 (18H, m, H-9, H-10, H-11, H-12, H-13, H-14, H-15, H-16, H-17), 1.37-1.44 (2H, m, H-4), 1.62-1.69 (2H, m, H-3), 2.01 (2H, dxt, 7.0 and 6.9, H-5 or H-8), 2.05 (2H, dxt, 7.1 and 7.1, H-5 or H-8), 2.36 (2H, t, 7.5, H-2), 5.29-5.41 (2H, m, H-6, H-7) and 11.60 (1H, br s, 1-OH). ^{13}C NMR (100 MHz, $CDCl_3$): 14.1 (C-18), 22.7 (C-17), 24.3 (C-3), 26.8-27.2 (C-5, C-8), 29.1 (C-4), 29.3-31.9 (C-9, C-10, C-11, C-12, C-13, C-14, C-15, C-16), 34.0 (C-2), 128.9-130.6 (C-6, C-7) and 180.1 (C-1). [7,48[4]]
Callyspongynic Acid (**44**)<br>$C_{32}H_{38}O_3$<br>Polyacetylenic Acid	*Callyspongia truncata*<br>Japan	^{1}H NMR (600 MHz, CD_3OD, δ/ppm, *J*/Hz): 2.28 (t, 6.9, H-4), 1.55 (m, H-5), 1.49 (m, H-6), 1.48 (m, H-7), 2.15 (tt, 6.9 and 2.1, H-8), 2.23 (t, 7.0, H-11), 1.60 (quint, 7.0, H-12), 2.24 (t, 7.0, H-13), 2.24 (t, 6.9, H-16), 1.65 (tt, 7.0 and 6.9, H-17), 2.36 (t, 7.1, H-18), 2.25 (t, 6.9, H-23), 1.52 (m, H-24), 1.42 (m, H-25, H-26), 2.08 (br dt, 6.4 and 6.2, H-27), 5.85 (ddt, 15.2, 1.2 and 6.4, H-28), 5.56 (ddt, 15.2, 6.2 and 1.7, H-29), 4.75 (br d, 6.2, H-30) and 2.85 (d, 2.3, H-32). ^{13}C NMR (CD_3OD): 163.1 (s, C-1), 79.0 (s, C-2), 83.3 (s, C-3), 19.2 (t, C-4), 28.9 (t, C-5), 29.1 (t, C-6), 29.7 (t, C-7), 19.3 (t, C-8), 81.3 (s, C-9), 80.1 (s, C-10), 18.5 (t, C-11), 29.7 (t, C-12), 18.5 (t, C-13), 81.1 (s, C-14), 80.0 (s, C-15), 18.5 (t, C-16), 29.1 (t, C-17), 18.8 (t, C-18), 77.1 (s, C-19), 66.7 (s, C-20), 66.3 (s, C-21), 78.0 (s, C-22), 19.6 (t, C-23), 29.1

		(t, C-24), 29.3 (t, C-25), 29.5 (t, C-26), 32.8 (t, C-27), 133.9 (d, C-28), 130.8 (d, C-29), 63.1 (d, C-30), 84.7 (s, C-31) and 74.5 (d, C-32). [44]
3-octadecyloxy-propane-1,2-diol (**45**) $C_{21}H_{44}O_3$ Glycerolipid[5]	*Callyspongia fibrosa* China	^{1}H NMR (500 MHz, $CDCl_3$, δ/ppm): 3.84 (1H, br s), 3.70 (1H), 3.63 (1H), 3.40-3.60 (4H, m), 1.55 (2H), 1.23 (30H) and 0.85 (3H, t). ^{13}C NMR ($CDCl_3$): 64.4 (t), 70.5 (d), 72.5 (t), 71.9 (t), 29.4 (t), 26.1 (t), 29.7 (t), 22.6 (t) and 14.1 (q). [45]
Batyl alcohol (**46**) $C_{22}H_{46}O_3$ Glycerolipid[5]	*Callyspongia fibrosa* India	^{1}H NMR* ($CHCl_3$, δ/ppm, *J*/Hz): 3.86 (1H, q, 6.5), 3.67–3.70 (2H, m), 3.43–3.58 (4H, m), 2.6 (1H, d, 6.5), 2.15 (1H, 6.5), 1.60 (2H, m), 1.28 (28H, br s) and 0.88 (3H, t, 6.5). [23]
Callyspongamide A (**47**) $C_{25}H_{33}NO$ Polyacetylenic Amide	*Callyspongia fistularis* Egypt	^{1}H NMR (600 MHz, $CDCl_3$, δ/ppm, *J*/Hz): 2.24 (t, 7.3, H-2), 2.38 (tt, 7.3 and 2.3, H-3), 2.00 (tt, 7.3 and 2.3, H-6), 1.36 (quint, 7.3, H-7), 1.22 (m, H-8, H-9, H-10, H-11), 1.33 (quint, 7.3, H-12), 2.25 (q, 7.3, H-13), 5.91 (td, 10.7 and 7.3, H-14), 5.36 (br d, 10.7, H-15), 3.00 (br s, H-17), 5.70 (br s, NH), 3.46 (q, 6.9, H-1′), 2.75 (t, 6.9, H-2′), 7.13 (d, 7.6, H-4′), 7.24 (t, 7.7, H-5′), 7.16 (t, 7.3, H-6′), 7.24 (t, 7.7, H-7′) and 7.13 (d, 7.6, H-8′). ^{13}C NMR (150 MHz, $CDCl_3$): 171.5 (s, C-1), 36.1 (t, C-2), 15.3 (t, C-3), 78.5 (s, C-4), 81.6 (s, C-5), 18.6 (t, C-6), 28.6 (t, C-7), 29.2 (t, C-8), 28.9 (t, C-9), 28.8 (t, C-10), 29.0 (t, C-11), 28.9 (t, C-12), 30.2 (t, C-13), 146.2 (d, C-14), 107.9 (d, C-15), 80.5 (s, C-16), 81.2 (d, C-17), 40.6 (t, C-1′), 35.7 (t, C-2′), 138.8 (s, C-3′), 128.7 (d, C-4′, C-8′), 128.6 (d, C-5′, C-7′) and 126.5 (d, C-6′). [46]

* Magnetic field strength did not report in the reference.
1 Accepted name for Siphonochalina truncate, according to the World Register of Marine Species (WoRMS).
2 IUPAC nomenclature (Name not specified by the authors).
3 Metabolite NMR data.
4 Cited reference.
5 Classification based on the electronic database The Human Metabolome Database (HMDB) Version 4.0.

Table S2. Terpenoids and steroids isolated from *Callyspongia* species.

**Metabolite name**<br>**Chemical formula**<br>**Type of metabolite**	**Specie**<br>**Geographic Location**	**^{1}H and ^{13}C NMR data [Reference]**
Callyspinol (**48**)<br>$C_{20}H_{38}O$<br>Diterpene	*Callyspongia spinosissima*<br>India	^{1}H NMR* (CDCl$_3$, δ/ppm, *J*/Hz): 5.20 (d, 12, H-1a), 5.05 (d, 10, H-1b), 5.94 (dd, 12 and 10, H-2), 1.28 (s, H-16), 0.83 (d, 3, H-17), 0.85 (d, 3, H-18), 0.86 (s, H-19) and 0.88 (s, H-20). ^{13}C NMR (CDCl$_3$): 111.4 (C-1), 143.0 (C-2), 73.3 (C-3), 42.7 (C-4), 21.4 (C-5), 39.4 (C-6), 32.8 (C-7), 37.4 (C-8), 24.8 (C-9), 32.7 (C-10), 27.7 (C-11), 29.8 (C-12), 37.4 (C-13), 24.5 (C-14), 33.9 (C-15), 27.99 (C-16), 22.6 (C-17), 22.7 (C-18), 19.70 (C-19) and 19.74 (C-20). [49]
Isocopalanol (**49**)<br>$C_{24}H_{44}O_5$<br>Polyoxygenated Diterpene	*Callyspongia* sp.<br>Indonesia	^{1}H NMR (500 MHz, CDCl$_3$, δ/ppm, *J*/Hz): 1.56 (1H, m, H-1), 1.3 (1H, m, H-1), 1.46 (1H, m, H-2), 1.53 (1H, m, H-2), 1.06 (1H, m, H-3), 1.29 (1H, m, H-3), 0.69 (1H, m, H-5), 1.62 (1H, m, H-6), 1.64 (1H, m, H-6), 1.33 (1H, m, H-7), 1.56 (1H, m, H-7), 1.36 (1H, m, H-9), 1.67 (1H, m, H-11), 1.82 (1H, m, H-11), 3.05 (1H, br t, 6.8 and 2.0, H-12), 1.46 (1H, m, H-14), 2.71 (1H, br d, 12.0, H-15), 1.12 (3H, s, H-16), 1.49 (3H, s, H-17), 0.82 (3H, s, H-18), 0.85 (3H, s, H-19), 0.84 (3H, s, H-20), 4.74 (1H, br t, 7.0 and 2.7, H-1′), 1.17 (3H, d, 7.0, H-2′), 4.81 (1H, br t, 7.0 and 2.5, H-1′′) and 1.15 (3H, s, 7.0, H-2′′). ^{13}C NMR (125 MHz, CDCl$_3$): 36.7 (t, C-1), 19.6 (t, C-2), 41.2 (t, C-3), 34.1 (s, C-4), 55.9 (d, C-5), 15.08 (t, C-6), 40.1 (t, C-7), 35.2 (s, C-8), 45.5 (d, C-9), 37.7 (s, C-10), 25.2 (t, C-11), 78.8 (d, C-12), 75.7 (s, C-13), 57.4 (d, C-14), 57.7 (t, C-15), 27.8 (q, C-16), 25.2 (q, C-17), 21.9 (q, C-18), 29.3 (q, C-19), 12.6 (q, C-20), 45.4 (d, C-1′), 15.4 (q, C-2′), 43.9 (d, C-1′′) and 15.4 (q, C-2′′). [50]
Akaterpin (**50**)<br>$C_{36}H_{52}Na_2O_6S_2$<br>Triterpene	*Callyspongia* sp.<br>Not reported	^{1}H NMR (400 MHz, CD$_3$OD, δ/ppm, *J*/Hz): 5.47 (ddd, H-2), 1.84 (ddd, H-3), 1.77 (ddd, H-3), 1.47 (m, H-4) 0.86 (d, 4-CH$_3$), 0.59 (s, 5-CH$_3$), 2.23 (m, H-6), 1.68 (m, H-7), 0.98 (m, H-7), 1.57 (m, H-8), 1.42 (m, H-9), 1.20 (m, H-9), 1.06 (s, 10-(CH$_3$)$_2$), 1.04 (s, 10-(CH$_3$)$_2$), 1.15 (m, H-11), 2.13 (ddd, H-12), 1.04 (m, H-12), 1.98 (m, H-2′), 1.46 (m, H-2′), 1.96 (m, H-3′), 1.20 (m, H-3′), 1.65 (m, H-4′), 1.15 (d, 4′-CH$_3$), 1.03 (s, 5′-CH$_3$), 1.68 (m, H-6′), 2.20 (m, H-7′), 1.99 (m, H-7′), 1.65 (m, H-8′), 2.32 (ddd, H-9′), 2.18 (m, H-9′), 4.76 (brs, 10′-CH$_2$), 4.73 (brs, 10′-CH$_2$), 3.32 (d, H-11′), 2.40 (d, H-11′), 7.30 (d, 2.4, H-3′′), 7.09 (dd, 2.4 and 8.5, H-5′′)

		and 7.38 (d, 8.5, H-6′′). ^{13}C NMR (100 MHz, CD_3OD): 147.4 (C-1), 117.4 (C-2), 32.7 (C-3), 34.6 (C-4), 15.6 (4-CH_3), 37.6 (C-5), 16.8 (5-CH_3), 41.0 (C-6), 28.9 (C-7), 23.3 (C-8), 42.2 (C-9), 37.1 (C-10), 30.3 (10-$(CH_3)_2$), 29.5 (10-$(CH_3)_2$), 30.2 (C-11), 36.4 (C-12), 44.1 (C-1′), 28.3 (C-2′), 26.2 (C-3′), 38.6 (C-4′), 16.5 (4′-CH_3), 43.1 (C-5′), 25.2 (5′-CH_3), 47.0 (C-6′), 22.4 (C-7′), 25.7 (C-8′), 34.0 (C-9′), 153.4 (C-10′), 109,4 (10′-CH_2), 37.3 (C-11′), 150.1 (C-1′′), 135.2 (C-2′′), 125.1 (C-3′′), 149.9 (C-4′′), 120.4 (C-5′′) and 123.2 (C-6′′). [51]
Ilhabelanol (**51**)<br>$C_{36}H_{54}Na_2O_9S_2$<br>Sulfated Meroterpenoid	*Callyspongia* sp.<br>Brazil	^{1}H NMR (600 MHz, CD_3OD, δ/ppm, *J*/Hz): 2.73 (1H, d, 16.0, H-1), 2.90 (1H, dd, 16.0 and 9.4, H-1), 2.41 (1H, br d, 8.9, H-2), 5.33 (1H, br s, H-4), 1.97 (2H, m, H-5), 1.36 (1H, m, H-6), 1.23 (1H, m, H-8), 1.95 (1H, m, H-8), 1.47 (2H, m, H-9), 1.00 (1H, m, H-10), 1.85 (1H, m, H-10), 1.27 (1H, m, H-12), 1.72 (1H, m, H-12), 1.25 (1H, m, H-13), 1.33 (1H, m, H-13), 0.71 (1H, m, H-14), 1.45 (1H, m, H-16), 1.75 (1H, m, H-16), 1.47 (1H, m, H-17), 1.60 (1H, m, H-17), 0.90 (1H, m, H-18), 0.94 (1H, m, H-20), 1.73 (1H, m, H-20), 1.46 (1H, m, H-21), 1.64 (1H, m, H-21), 1.21 (1H, m, H-22), 1.40 (1H, m, H-22), 1.43 (3H, br s, H-24), 0.96 (3H, s, H-25), 0.95 (3H, s, H-26), 1.13 (3H, s, H-27), 0.99 (3H, s, H-28), 0.88 (3H, s, H-29), 0.85 (3H, s, H-30), 7.30 (1H, d, 2.4, H-3′), 7.06 (1H, dd, 8.8 and 2.4, H-5′) and 7.37 (1H, d, 8.8, H-6′). ^{13}C NMR (150 MHz, CD_3OD): 28.1 (t, C-1), 56.4 (d, C-2), 137.1 (s, C-3), 123.0 (d, C-4), 24.4 (t, C-5), 54.0 (d, C-6), 38.3 (s, C-7), 40.9 (t, C-8), 20.1 (t, C-9), 38.4 (t, C-10), 37.9 (s, C-11), 37.7 (t, C-12), 20.8 (t, C-13), 61.6 (d, C-14), 74.2 (s, C-15), 43.3 (t, C-16), 19.8 (t, C-17), 57.6 (d, C-18), 40.6 (s, C-19), 40.8 (t, C-20), 19.5 (t, C-21), 43.5 (t, C-22), 34.4 (s, C-23), 23.0 (q, C-24), 15.6 (q, C-25), 29.6 (q, C-26), 31.2 (q, C-27), 16.0 (q, C-28), 34.2 (q, C-29), 22.4 (q, C-30), 148.8 (s, C-1′), 139.3 (s, C-2′), 123.3 (d, C-3′), 150.9 (s, C-4′), 120.1 (d, C-5′) and 123.6 (d, C-6′). [11]
Ilhabrene (**52**)<br>$C_{31}H_{44}Na_2O_8S_2$<br>Sulfated Meroterpenoid	*Callyspongia* sp.<br>Brazil	^{1}H NMR (600 MHz, CD_3OD, δ/ppm, *J*/Hz): 2.92 (2H, d, 7.1, H-1), 2.29 (1H, br t, 6.7, H-2), 2.04 (1H, m, H-4), 2.33 (1H, dt, 12.7 and 2.6, H-4), 1.39 (1H, m, H-5), 1.75 (1H, m, H-5), 1.32 (1H, m, H-6), 1.30 (1H, m, H-8), 1.88 (1H, br d, 11.8, H-8), 1.46 (2H, m, H-9), 1.04 (1H, td, 13.5 and 4.8, H-10), 1.74 (1H, m, H-10), 0.85 (1H, m, H-12), 1.63 (1H, m, H-12), 1.30 (1H, m, H-13), 1.37

		(1H, m, H-13), 1.62 (1H, m, H-14), 2.06 (2H, m, H-16), 1.55 (2H, m, H-17), 1.23 (1H, m, H-18), 1.53 (1H, m, H-18), 4.63 (1H, br s, H-20), 4.69 (1H, br s, H-20), 0.80 (3H, s, H-21), 0.90 (3H, s, H-22), 4.54 (1H, br s, H-23), 4.78 (1H, br s, H-23), 0.92 (3H, s, H-24), 0.85 (3H, s, H-25), 7.12 (1H, d, 2.8, H-3′), 7.04 (1H, dd, 8.9 and 2.8, H-5′) and 7.34 (1H, d, 8.9, H-6′). ^{13}C NMR (150 MHz, CD_3OD): 25.2 (t, C-1), 57.5 (d, C-2), 149.2 (s, C-3), 39.6 (t, C-4), 25.6 (t, C-5), 59.3 (d, C-6), 41.1 (s, C-7), 40.6 (t, C-8), 20.5 (t, C-9), 38.8 (t, C-10), 37.3 (s, C-11), 32.6 (t, C-12), 22.2 (t, C-13), 56.5 (d, C-14), 151.5 (s, C-15), 33.4 (t, C-16), 25.0 (t, C-17), 37.6 (t, C-18), 36.0 (s, C-19), 108.9 (t, C-20), 16.2 (q, C-21), 30.0 (q, C-22), 109.6 (t, C-23), 29.1 (q, C-24), 27.3 (q, C-25), 149.1 (s, C-1′), 137.7 (s, C-2′), 123.2 (d, C-3′), 150.7 (s, C-4′), 119.9 (d, C-5′) and 123.5 (d, C-6′). [11]
Isoakaterpin (**53**)<br>$C_{36}H_{52}Na_2O_8S_2$<br>Sulfated Meroterpenoid	*Callyspongia* sp.<br>Brazil	^{1}H NMR (600 MHz, CD_3OD, δ/ppm, *J*/Hz): 2.38 (1H, d, 14.1, H-1), 3.30 (1H, d, 14.1, H-1), 1.65 (1H, m, H-3), 1.20 (1H, m, H-4), 1.97 (1H, m, H-4), 1.37 (1H, m, H-5), 2.00 (1H, m, H-5), 1.60 (1H, m, H-7), 1.91 (1H, m, H-8), 2.17 (1H, m, H-8), 1.60 (1H, m, H-9), 1.86 (1H, m, H-9), 2.16 (1H, m, H-10), 2.33 (1H, td, 13.0 and 6.4, H-10), 0.76 (1H, td, 13.0 and 2.8, H-12), 1.87 (1H, m, H-12), 0.91 (1H, m, H-13), 1.30 (1H, m, H-13), 1.24 (1H, m, H-15), 1.40 (1H, m, H-16), 1.50 (1H, m, H-16), 1.07 (1H, dd, 12.4 and 3.9, H-17), 1.80 (1H, m, H-17), 1.60 (1H, m, H-18), 5.38 (1H, br s, H-20), 2.01 (1H, m, H-21), 2.10 (1H, m, H-21), 1.12 (1H, m, H-22), 1.39 (1H, m, H-22), 1.15 (3H, d, 6.9, H-24), 1.00 (3H, s, H-25), 4.72 (1H, br s, H-26), 4.83 (1H, br s, H-26), 0.84 (3H, d, 6.6, H-27), 1.03 (3H, s, H-28), 0.97 (3H, s, H-29), 0.86 (3H, s, H-30), 7.31 (1H, d, 2.8, H-3′), 7.08 (1H, dd, 8.9 and 2.8, H-5′) and 7.37 (1H, d, 8.9, H-6′). ^{13}C NMR (150 MHz, CD_3OD): 37.3 (t, C-1), 43.3 (s, C-2), 38.7(d, C-3), 26.4 (t, C-4), 28.6 (t, C-5), 43.7 (s, C-6), 46.8 (d, C-7), 22.3 (t, C-8), 25.7 (t, C-9), 34.3 (t, C-10), 154.0 (s, C-11), 38.0 (t, C-12), 25.1 (t, C-13), 43.2 (s, C-14), 46.4 (d, C-15), 32.6 (t, C-16), 31.4 (t, C-17), 45.3 (d, C-18), 147.6 (s, C-19), 118.2 (d, C-20), 24.4 (t, C-21), 33.0 (t, C-22), 32.7 (s, C-23), 16.7 (q, C-24), 25.1 (q, C-25), 108.7 (t, C-26), 17.0 (q, C-27), 24.3 (q, C-28), 28.6 (q, C-29), 28.3 (q, C-30), 150.1 (s, C-1′), 135.4 (s, C-2′),

		125.1 (d, C-3′), 150.3 (s, C-4′), 120.5 (d, C-5′) and 123.4 (d, C-6′). [11]
(2*S*,4*aS*,5*S*,6*R*,8*aS*)-5-(2-((1*S*,3*aS*,5*R*,8*aS*,*Z*)-1-hydroxy-1,4,4,6-tetramethyl-1,2,3,3a,4,5,8,8a-octahydroazulen-5-yl)-ethyl)-4a,6-dimethyloctahydro-2H-chromene-2,6-diol (**54**)<br>$C_{27}H_{46}O_4$<br>Sipholane triterpenoid	*Callyspongia siphonella*<br>Egypt	^{1}H NMR (400 MHz, C_5D_5N, δ/ppm, *J*/Hz): 2.01(m, H-2), 2.12 (m, H-3), 5.23 (d, 9.2, H-4), 3.31 (ddd, 11.9 and 3.9, H-5), 1.74 (m, H-6), 2.36 (m, H-6), 1.63 (m, H-7), 1.95 (m, H-7), 0.85 (m, H-9), 1.35 (m, H-10), 2.13 (m, H-10), 1.97 (m, H-11), 2.32 (m, H-11), 1.76 (m, H-12), 5.49 (m, H-14), 1.96 (m, H-15), 2.35 (m, H-15), 2.18 (m, H-16), 1.40 (m, H-18), 1.89 (m, H-18), 2.03 (H-19), 2.25 (m, H-19), 3.06 (m, H-20), 1.56 (s, H-22), 1.43 (s, H-23), 1.72 (s, H-24), 1.46 (s, H-25), 1.16 (s, H-26) and 1.21 (s, H-27). ^{13}C NMR (100 MHz, C_5D_5N): 37.7 (C-1), 30.5 (C-2), 37.8 (C-3), 97.6 (C-4), 82.2 (C-5), 25.2 (C-6), 40.3 (C-7), 71.9 (C-8), 55.3 (C-9), 26.4 (C-10), 34.4 (C-11), 57.9 (C-12), 143.2 (C-13), 121.9 (C-14), 25.2 (C-15), 49.1 (C-16), 80.5 (C-17), 36.9 (C-18), 25.6 (C-19), 52.9 (C-20), 35.6 (C-21), 13.3 (C-22), 30.6 (C-23), 30.1 (C-24), 26.0 (C-25), 29.6 (C-26) and 31.9 (C-27). [52]
Dahabinone A (**55**)<br>$C_{30}H_{50}O_5$<br>Triterpene	*Callyspongia siphonella*<br>Gulf of Eilat	^{1}H NMR (500 MHz, $CDCl_3$, δ/ppm, *J*/Hz): 1.15 (m, H-2), 1.68 (m, H-2), 3.10 (ddd, 13.4, 11.2 and 2.2, H-3), 2.03 (m, H-3′), 2.98 (dd, 8.7 and 6.9, H-7), 1.81 (d, 10.1, H-11), 5.84 (dd, 15.6 and 10.1, H-12), 5.64 (d, 15.6, H-13), 2.02 (m, H-15), 2.47 (d, 5.1, H-18), 1.16 (s, Me-24), 1.32 (s, Me-25), 1.25 (s, Me-26), 1.20 (s, Me-27), 0.79 (d, 7.0, Me-28), 1.23 (s, Me-29), 1.09 (s, Me-30) and 1.06 (s, Me-31). ^{13}C NMR (125 MHz, $CDCl_3$): 41.4 (s, C-1), 40.4 (t, C-2), 35.5 (t, C-3), 217.5 (s, C-4), 82.6 (s, C-5), 81.0 (d, C-7), 27.1 (t, C-8), 38.7 (t, C-9), 71.2 (s, C-10), 62.1 (d, C-11), 125.9 (d, C-12), 133.5 (d, C-13), 92.3 (s, C-14), 40.5 (d, C-15), 35.2 (t, C-16), 28.3 (t, C-17), 46.3 (d, C-18), 73.8 (s, C-19), 41.9 (t, C-20), 24.1 (t, C-21), 32.2 (t, C-22), 33.0 (s, C-23), 12.4 (q, Me-24), 20.6 (q, Me-25), 26.7 (q, Me-26), 28.8 (q, Me-27), 19.2 (q, Me-28), 24.6 (q, Me-29), 25.0 (q, Me-30) and 34.2 (q, Me-31). [53]
Neviotine A (**56**)<br>$C_{30}H_{50}O_6$<br>Triterpene	*Callyspongia siphonella*<br>Gulf of Eilat	^{1}H NMR (360 MHz, $CDCl_3/CD_3OD$, δ/ppm): 4.10 (H-3), 5.10 (H-5), 5.02 (H-7), 1.43 (H-8), 1.84 (H-8), 1.82 (H-9), 1.88 (H-9), 1.54 (H-11), 1.68 (H-12), 1.83 (H-12), 1.69 (H-13), 1.76 (H-13), 1.39 (H-14), 1.11 (H-16), 1.65 (H-16), 1.35 (H-17), 1.45 (H-17), 1.41 (H-18), 1.71 (H-20), 1.82 (H-20), 1.45 (H-21), 2.30 (H-21), 1.46 (H-23), 1.76 (H-23), 1.31 (H-24), 1.17 (H-25), 0.67 (H-26), 1.30 (H-27), 1.76 (H-28), 0.89 (H-29), 0.87 (H-30) and 1.25

		(H-31). ^{13}C NMR (90 MHz, $CDCl_3$/CD_3OD): 75.92 (C-2), 83.17 (C-3), 213.01 (C-4), 74.59 (C-5), 43.47 (C-6), 68.36 (C-7), 25.83 (C-8), 36.09 (C-9), 42.50 (C-10), 54.73 (C-11), 22.55 (C-12), 21.48 (C-13), 62.22 (C-14), 73.76 (C-15), 36.00 (C-16), 20.38 (C-17), 53.86 (C-18), 87.53 (C-19), 35.55 (C-20), 35.15 (C-21), 41.38 (C-22), 46.49 (C-23), 21.63 (C-24), 25.83 (C-25), 13.84 (C-26), 19.34 (C-27), 32.51 (C-28), 16.71 (C-29), 16.11 (C-30) and 34.11 (C-31). [54]
	Callyspongia siphonella Saudi Arabia	^{1}H NMR (600 MHz, $CDCl_3$, δ/ppm, *J*/Hz): 4.24 (1H, d, 4.8, H-3), 5.07 (1H, d, 7.2, H-5), 4.88 (1H, dd, 13.2 and 3.6, H-7), 1.47 (1H, m, H-8), 1.75 (1H, m, H-8), 1.07 (1H, m, H-9), 1.61 (1H, m, H-9), 1.56 (1H, m, H-11), 1.70 (1H, m, H-12), 1.88 (1H, m, H-12), 1.71 (1H, m, H-13), 1.74 (1H, m, H-13), 1.40 (1H, m, H-14), 1.09 (1H, m, H-16), 1.62 (1H, m, H-16), 1.37 (1H, m, H-17), 1.44 (1H, m, H-17), 1.40 (1H, m, H-18), 1.85 (1H, m, H-20), 1.90 (1H, m, H-20), 1.48 (1H, m, H-21), 2.31 (1H, br dt, 12.0 and 5.4, H-21), 1.43 (1H, br d, 14.4, H-23), 1.79 (1H, br d, 14.4, H-23), 1.33 (3H, s, H-24), 1.22 (3H, s, H-25), 0.69 (3H, s, H-26), 1.31 (3H, s, H-27), 1.79 (1H, br s, H-28), 0.90 (1H, d, 7.2, H-29), 0.89 (3H, d, 7.2, H-30), 1.26 (3H, s, H-31), 2.84 (1H, d, 4.8, OH-3) and 3.36 (1H, d, 7.8, OH-5). ^{13}C NMR (150 MHz, $CDCl_3$): 76.2 (C-2), 84.4 (C-3), 212.3 (C-4), 75.5 (C-5), 44.4 (C-6), 68.9 (C-7), 26.5 (C-8), 36.9 (C-9), 43.1 (C-10), 55.1 (C-11), 23.2 (C-12), 22.0 (C-13), 62.4 (C-14), 74.4 (C-15), 36.7 (C-16), 20.9 (C-17), 54.8 (C-18), 88.2 (C-19), 36.6 (C-20), 35.6 (C-21), 42.0 (C-22), 47.3 (C-23), 22.3 (C-24), 26.8 (C-25), 14.6 (C-26), 20.4 (C-27), 32.9 (C-28), 17.5 (C-29), 16.9 (C-30) and 35.0 (C-31). [55]
	Callyspongia siphonella Saudi Arabia	^{1}H NMR (500 MHz, $CDCl_3$, δ/ppm, *J*/Hz): 0.67 (1H, m, H-26), 0.87 (1H, d, 6.6, H-30), 0.89 (1H, d, 6.0, H-29), 1.17 (1H, m, H-25), 1.11 (1H, m, H-16), 1.25 (1H, m, H-31), 1.31 (1H, m, H-24), 1.35 (1H, m, H-17), 1.30 (1H, m, H-27), 1.38 (1H, m, H-14), 1.41 (1H, m, H-18), 1.46 (1H, m, H-23), 1.45 (1H, m, H-17′, H-21), 1.46 (1H, m, H-23), 1.65 (1H, m, H-16′), 1.69 (1H, m, H-12), 1.70 (1H, m, H-13), 1.71 (1H, m, H-20), 1.76 (1H, m, H-13′, H-23, H-28), 1.76 (1H, s, H-28), 1.82 m (1H, m, H-8, H-20), 1.83 (1H, m, H-12′), 1.84 (1H, m, H-8′), 1.88 (1H, m, H-9), 1.54 (1H, m, H-11), 2.30 (1H, m, H-

		21), 3.50 (1H, d, 8.0, OH-5), 3.98 (1H, d, 4.5, OH-3), 4.16 (1H, d, 4.0, H-3), 4.92 (1H, dd, 13.0 and 3.5, H-7) and 5.03 (1H, d, 5.0, H-5). ^{13}C NMR (125 MHz, $CDCl_3$): 14.1 (C-26), 16.9 (C-30), 17.5 (C-29), 20.5 (C-27), 21.0 (C-17), 22.2 (C-13), 22.7 (C-12, C-24), 26.5 (C-8), 26.9 (C-25), 31.6 (C-28), 33.1 (C-31), 35.2 (C-21), 35.8 (C-20), 36.7 (C-9), 37.1 (C-16), 42.1 (C-22), 43.3 (C-10), 44.4 (C-6), 47.4 (C-23), 54.8 (C-18), 55.8 (C-10), 62.4 (C-14), 68.9 (C-7), 74.5 (C-15), 75.7 (C-5), 76.8 (C-2), 84.3 (C-3), 88.3 (C-19) and 212.9 (C-4). [56]
	Callyspongia siphonella Egypt	^{13}C NMR (400 MHz, $CDCl_3$): 76.3 (C-2), 84.3 (C-3), 212.6 (C-4), 75.4 (C-5), 44.3 (C-6), 68.9 (C-7), 26.5 (C-8), 36.6 (C-9), 43.1 (C-10), 55.1 (C-11), 23.2 (C-12), 22.3 (C-13), 62.4 (C-14), 74.4 (C-15), 37.0 (C-16), 20.9 (C-17), 54.7 (C-18), 88.2 (C-19), 36.7 (C-20), 35.6 (C-21), 42.0 (C-22), 47.3 (C-23), 22.1 (C-24), 26.9 (C-25), 14.5 (C-26), 20.4 (C-27), 33.0 (C-28), 17.5 (C-29), 16.9 (C-30) and 35.1 (C-31). [57]
Neviotine B (**57**) $C_{30}H_{50}O_6$ Triterpene	*Callyspongia siphonella* Gulf of Eilat	^{1}H NMR (500 MHz, $CDCl_3$, δ/ppm, *J*/Hz): 4.14 (s, H-3), 5.08 (s, H-25), 4.97 (dd, 3.0 and 12.5, H-7), 2.26 (dt, 5.0 and 11.4, H-21), 1.32 (s, Me-24), 1.20 (s, Me-25), 0.68 (s, Me-26), 1.28 (s, Me-27), 0.91 (d, 6.5, Me-29), 0.89 (d, 6.5, Me-30) and 1.25 (s, Me-31). ^{13}C NMR (125 MHz, $CDCl_3$): 76.3 (s, C-1), 84.1 (d, C-2), 213.4 (s, C-4), 75.4 (d, C-5), 44.3 (s, C-6), 68.9 (d, C-7), 26.6 (t, C-8), 36.6 (t, C-9), 43.5 (s, C-10), 55.0 (d, C-11), 23.4 (t, C-12), 23.6 (t, C-13), 61.1 (d, C-14), 75.4 (s, C-15), 31.4 (t, C-16), 26.6 (t, C-17), 54.1 (d, C-18), 88.1 (s, C-19), 36.3 (t, C-20), 35.9 (t, C-21), 41.8 (s, C-22), 45.0 (t, C-23), 22.2 (q, Me-24), 26.8 (q, Me-25), 14.6 (q, Me-26), 20.0 (q, Me-27), 33.9 (d, C-28), 17.3 (q, Me-29), 16.8 (q, Me-30) and 33.0 (q, Me-31). [53]
Neviotine C (**58**) $C_{30}H_{50}O_6$ Triterpene	*Callyspongia siphonella* Saudi Arabia	^{1}H NMR (600 MHz, $CDCl_3$, δ/ppm, *J*/Hz): 4.66 (1H, dd, 9.0 and 7.8, H-4), 3.46 (1H, d, 9.0, H-5), 3.34 (1H, dd, 12.0 and 1.2, H-7), 1.50 (1H, m, H-8), 1.88 (1H, m, H-8), 1.06 (1H, m, H-9), 1.59 (1H, m, H-9), 1.55 (1H, m, H-11), 1.72 (1H, m, H-12), 1.86 (1H, m, H-12), 1.62 (1H, m, H-13), 1.70 (1H, m, H-13), 1.36 (1H, m, H-14), 1.69 (1H, m, H-16), 1.83 (1H, m, H-16), 1.34 (1H, m, H-17), 1.44 (1H, m, H-17), 1.34 (1H, m, H-18), 1.77 (1H, m, H-20), 1.86 (1H, m, H-20), 1.46 (1H, m, H-21), 2.28 (1H, br dt, 12.0 and 5.4, H-21), 1.39 (1H, br d, H-23), 1.72 (1H, br d, H-23), 1.30 (3H, s, H-24), 1.41 (3H,

		s, H-25), 1.16 (3H, s, H-26), 1.00 (3H, s, H-27), 1.78 (1H, m, H-28), 0.89 (3H, d, 7.2, H-29), 0.88 (3H, d, 7.2, H-30), 1.25 (3H, s, H-31), 3.42 (1H, d, 7.8, OH-4) and 3.01 (1H, s, OH-5). ^{13}C NMR (150 MHz, $CDCl_3$): 83.2 (C-2), 215.3 (C-3), 76.3 (C-4), 79.1 (C-5), 43.1 (C-6), 76.3 (C-7), 26.6 (C-8), 36.9 (C-9), 42.8 (C-10), 55.6 (C-11), 22.9 (C-12), 21.9 (C-13), 62.3 (C-14), 74.3 (C-15), 36.7 (C-16), 20.9 (C-17), 54.8 (C-18), 88.2 (C-19), 36.3 (C-20), 35.6 (C-21), 42.0 (C-22), 47.3 (C-23), 21.6 (C-24), 26.5 (C-25), 15.5 (C-26), 21.5 (C-27), 32.9 (C-28), 17.5 (C-29), 16.9 (C-30) and 35.0 (C-31). [55]
Neviotine D (**59**)<br>$C_{30}H_{50}O_6$<br>Triterpene	*Callyspongia siphonella*<br>Egypt	^{1}H NMR (600 MHz, $CDCl_3$, δ/ppm, *J*/Hz): 4.46 (s, H-3β), 3.99 (s, H-5), 3.87 (dd, 12.0 and 3.6, H-7), 1.43 (m, H-8α), 1.70 (m, H-8β), 1.67 (m, H-9α), 1.74 (m, H-11), 1.69 (m, H-12a), 1.18 (m, H-13a), 1.69 (m, H-13b), 1.37 (m, H-14), 1.74 (m, H-16), 1.31 (m, H-17α, H-18), 1.81 (m, H-20α), 1.65 (m, H-20β), 1.43 (m, H-21α), 2.30 (dt, 12.6 and 6.0, H-21β), 1.71 (m, H-23α), 1.39 (m, H-23β), 1.01 (s, H-24), 1.36 (s, H-25), 1.21 (s, H-26), 1.05 (s, H-27), 1.76 (m, H-28), 0.88 (d, 7.2, H-29), 0.87 (d, 7.2, H-30), 1.23 (s, H-31) and 3.59 (s, OH-3). ^{13}C NMR (150 MHz, $CDCl_3$): 76.8 (C-2), 81.8 (C-3), 213.8 (C-4), 82.8 (C-5), 42.3 (C-6), 70.2 (C-7), 27.3 (C-8), 37.1 (C-9), 42.3 (C-10), 58.6 (C-11), 22.3 (C-12), 21.1 (C-13), 62.4 (C-14), 74.4 (C-15), 37.0 (C-16), 19.9 (C-17), 54.9 (C-18), 88.2 (C-19), 36.8 (C-20), 35.7 (C-21), 42.1 (C-22), 47.6 (C-23), 17.8 (C-24), 28.9 (C-25), 23.4 (C-26), 23.9 (C-27), 33.0 (C-28), 17.5 (C-29), 16.9 (C-30) and 35.0 (C-31). [57]
Sipholenol A (15-sipholen-4,10,19-triol) (**60**)<br>$C_{30}H_{52}O_4$<br>Sipholane triterpenoid	*Callyspongia siphonella*<br>Gulf of Eilat	^{1}H NMR (270 MHz, $CDCl_3$, δ/ppm, *J*/Hz): 0.99, 1.03, 1.08, 1.09, 1.13, 1.24, 1.25, 1.76, 2.45 (1H, m), 3.46 (1H, dd, 15.3 and 4.4), 3.77 (1H, d, 6.5) and 5.39 (1H, m). ^{13}C NMR (75 MHz, $CDCl_3$): 143.2 (s, C=CH), 121.3 (d, C=CH), 77.0 (d, C-4), 82.1 (s, C-10 or C-19), 72.5 (s, C-10 or C-19), 77.9 (s, C-5), 76.5 (d, C-7), 57.6 (C-11), 55.7 (C-14), 52.6 (C-18) and 48.6 (C-22). [25]
	Callyspongia siphonella<br>Egypt	^{1}H NMR (400 MHz, $CDCl_3/CD_3OD$ 4:1, δ/ppm): 1.45 (H-2), 1.64 (H-2), 1.70 (H-3), 1.86 (H-3), 3.74 (H-4), 3.56 (H-7), 1.33 (H-8), 1.70 (H-8), 1.47 (H-9), 1.61 (H-9), 0.87 (H-11), 1.21 (H-12), 1.55 (H-12), 1.80 (H-13), 2.01 (H-13), 1.64 (H-14), 5.45 (H-16), 1.80 (H-17), 2.01 (H-17), 1.81 (H-18), 1.59 (H-20), 1.66 (H-20), 1.77 (H-21), 1.98 (H-21), 2.50 (H-22), 0.97 (H-24), 1.25 (H-25),

		1.12 (H-26), 1.08 (H-27), 1.77 (H-28), 1.23 (H-29), 1.03 (H-30) and 1.09 (H-31). ^{13}C NMR (100 MHz, $CDCl_3$/CD_3OD 4:1): 42.16 (s, C-1), 33.59 (t, C-2), 24.57 (t, C-3), 75.92 (d, C-4), 78.00 (s, C-5), 76.18 (d, C-7), 26.16 (t, C-8), 38.60 (t, C-9), 71.93 (s, C-10), 55.37 (d, C-11), 26.43 (t, C-12), 33.42 (t, C-13), 57.26 (d, C-14), 142.71 (s, C-15), 120.90 (d, C-16), 24.30 (t, C-17), 47.99 (d, C-18), 81.40 (s, C-19), 36.54 (t, C-20), 24.74 (t, C-21), 52.25 (d, C-22), 34.99 (s, C-23), 12.56 (q, C-24), 21.04 (q, C-25), 28.52 (q, C-26), 29.19 (q, C-27), 29.55 (q, C-28), 24.90 (q, C-29), 28.98 (q, C-30) and 31.19 (q, C-31). [58,64[1]]
	Callyspongia siphonella Egypt	Spectroscopic data not provided (References 61 and 64 are cited). [59]
	Callyspongia siphonella Saudi Arabia	^{1}H NMR (600 MHz, $CDCl_3$, δ/ppm, *J*/Hz): 2.02 (1H, m, H-2), 1.72 (1H, m, H-2), 1.97 (1H, m, H-3), 1.77 (1H, m, H-3), 3.82 (1H, d, 6.6, H-4), 3.51 (1H, dd, 12.0 and 4.2, H-7), 1.71 (1H, m, H-8), 1.38 (1H, m, H-8), 1.61 (1H, m, H-9), 1.46 (1H, m, H-9), 0.86 (1H, br dd, 2.4 and 1.8, H-11), 1.52 (1H, m, H-12), 1.20 (1H, m, H-12), 1.99 (1H, m, H-13), 1.94 (1H, m, H-13), 1.62 (1H, m, H-14), 5.46 (1H, br dd, 9.6 and 4.8, H-16), 2.06 (1H, m, H-17), 1.81 (1H, m, H-17), 2.48 (1H, ddd, 12.6 and 8.4, H-18), 1.58 (1H, m, H-20), 1.45 (1H, dt, 13.8 and 4.8, H-20), 1.66 (1H, m, H-21), 1.59 (1H, m, H-21), 1.80 (1H, m, H-22), 1.00 (3H, s, H-24), 1.27 (3H, s, H-25), 1.13 (3H, s, H-26), 1.08 (3H, s, H-27), 1.76 (br s, H-28), 1.26 (3H, s, H-29), 1.03 (3H, s, H-30) and 1.09 (3H, s, H-31). ^{13}C NMR (150 MHz, $CDCl_3$): 42.6 (C-1), 25.1 (C-2), 33.7 (C-3), 77.0 (C-4), 77.8 (C-5), 76.4 (C-7), 26.6 (C-8), 39.1 (C-9), 72.4 (C-10), 55.7 (C-11), 26.8 (C-12), 24.9 (C-13), 57.5 (C-14), 143.1 (C-15), 121.2 (C-16), 24.7 (C-17), 52.7 (C-18), 82.1 (C-19), 33.8 (C-20), 37.1 (C-21), 48.8 (C-22), 35.4 (C-23), 12.9 (C-24), 21.4 (C-25), 29.1 (C-26), 29.8 (C-27), 30.2 (C-28), 25.6 (C-29), 29.5 (C-30) and 31.7 (C-31). [55]
	Callyspongia siphonella Saudi Arabia	^{1}H NMR (500 MHz, $CDCl_3$, δ/ppm, *J*/Hz): 0.87 (1H, m, H-11), 1.00 (3H, s, H-24), 1.03 (3H, s, H-31), 1.08 (3H, s, H-30), 1.10 (3H, s, H-29), 1.13 (3H, s, H-26), 1.25 (3H, s, H-27), 1.27 (3H, s, H-25), 1.40 (1H, m, H-2), 1.50 (1H, m, H-2′, H-12), 1.54 (1H, m, H-9), 1.62 (1H, m, H-9′), 1.65 (1H, m, H-20), 1.71 (1H, m, H-8), 1.72 (1H, m, H-3), 1.73 (1H, m, H-17), 1.75 (1H, m, H-

		21), 1.76 (3H, br s, H-28), 1.79 (1H, m, H-13), 1.80 (1H, m, H-18), 1.97 (1H, m, H-3′), 1.98 (2H, m, H-13′, H-21′), 2.02 (1H, m, H-14), 2.49 (1H, m, H-22), 3.46 (1H, dd, 11.0 and 4.5, H-7), 3.50 (1H, d, 8.0, OH-5), 3.81 (1H, dd, 6.5 and 3.5), 3.98 (1H, d, 4.5, OH-3) and 5.45 (1H, br dd, 8.5 and 4.5, H-16). ^{13}C NMR (125 MHz, $CDCl_3$): 13.1 (C-24), 21.5 (C-25), 24.9 (C-17), 25.1 (C-3), 25.3 (C-21), 25.7 (C-29), 26.8 (C-8, C-12), 29.2 (C-26), 29.6 (C-27), 29.9 (C-30), 30.2 (C-28), 31.7 (C-31), 33.8 (C-13), 34.0 (C-2), 35.5 (C-23), 37.3 (C-20), 39.2 (C-9), 42.8 (C-1), 49.0 (C-18), 53.0 (C-22), 55.9 (C-11), 57.7 (C-14), 72.5 (C-10), 76.3 (C-7), 76.8 (C-4), 78.0 (C-5), 82.2 (C-19), 121.4 (C-16) and 143.2 (C-15). [56]
	Callyspongia siphonella Saudi Red Sea territorial water	Spectroscopic data not provided (Reference 25 is cited). [60]
	Callyspongia siphonella Saudi Arabia	Spectroscopic data not provided (Reference 61 is cited). [8]
	Callyspongia siphonella Egypt	Spectroscopic data not provided (Reference 61 is cited). [7]
	Callyspongia siphonella Egypt	^{13}C NMR (150 MHz, $CDCl_3$): 42.6 (C-1), 25.1 (C-2), 33.7 (C-3), 77.2 (C-4), 77.8 (C-5), 76.4 (C-7), 26.6 (C-8), 39.1 (C-9), 72.4 (C-10), 55.7 (C-11), 26.8 (C-12), 24.9 (C-13), 57.5 (C-14), 143.1 (C-15), 121.2 (C-16), 24.7 (C-17), 52.7 (C-18), 82.1 (C-19), 33.8 (C-20), 37.1 (C-21), 48.8 (C-22), 35.4 (C-23), 12.9 (C-24), 21.4 (C-25), 29.1 (C-26), 29.8 (C-27), 30.2 (C-28), 25.6 (C-29), 29.5 (C-30) and 31.6 (C-31). [57]
	Callyspongia siphonella Gulf of Eilat	^{1}H NMR (270 MHz, $CDCl_3$, δ/ppm, *J*/Hz): 3.77 (d, 6.5, H-4), 3.46 (dd, 11.7 and 4.4, H-7), 5.36 (br dd, 8.2 and 4.7, H-16), 0.99 (s, Me(24)), 1.24 (s, Me(25)), 1.13 (s, Me(26)), 1.25 (s, Me(27)), 1.75 (br s, Me(28)), 1.09 (s, Me(29)), 1.08 (s, Me(30)) and 1.03 (s, Me(31)). ^{13}C NMR (75 MHz, $CDCl_3$): 42.78 (d, C-1), 77.14 (d, C-4), 77.83 (s, C-5), 76.54 (d, C-7), 37.27 (t), 39.24 (t, C-9), 72.44 (s, C-10), 55.87 (d, C-11), 57.73 (d, C-14), 143.23 (s, C-15), 121.37 (d, C-16), 52.87 (d, C-18), 82.14 (s, C-19), 48.95 (d, C-22), 35.50 (s, C-23), 34.02 (t), 33.82 (t), 31.70 (q), 30.14 (q), 29.90 (t), 29.51 (q), 29.18 (q) 26.93 (t), 26.72 (t), 25.83 (q), 25.28 (q), 25.04 (t), 24.83 (t), 21.42 (q) and 13.03 (q, C-24). [61]
Sipholenol B (**61**) $C_{30}H_{52}O_4$ Sipholane triterpenoid	*Callyspongia siphonella* Gulf of Eilat	^{1}H NMR (270 MHz, $CDCl_3$, δ/ppm, *J*/Hz): 3.82 (d, 6.4, H-4), 3.60 (dd, 10.9 and 5.0, H-7), 5.50 (br dd, 10.0 and 4.5, H-16), 0.82 (s, Me(24)), 1.24 (s, Me(25)), 1.12

		(s, Me(26)), 1.27 (s, Me(27)), 1.77 (br s, Me(28)), 1.15 (s, Me(29)), 1.09 (s, Me(30)) and 1.03 (s, Me(31)). ^{13}C NMR (75 MHz, $CDCl_3$): 143.69 (s, C-15), 121.73 (d, C-16), 82.28 (s, C-19), 77.92 (s, C-5), 77.08 (d, C-4), 76.49 (d, C-7), 74.02 (s, C-10), 57.71 (d, C-14), 58.36 (d, C-11), 52.84 (d, C-18), 48.94 (d, C-22), 42.76 (s, C-1), 40.42 (t, C-9), 37.36 (t), 35.55 (s, C-23), 34.73 (t), 33.54 (t), 31.47 (q), 30.08 (q), 29.52 (q), 29.07 (q), 28.94 (t), 27.05 (t), 25.61 (q), 25.48 (t), 25.10 (t), 24.82 (t), 23.63 (q), 21.56 (q) and 12.94 (q, C-24). [61]
Sipholenol C (**62**)<br>$C_{30}H_{52}O_4$<br>Sipholane triterpenoid	*Callyspongia siphonella*<br>Gulf of Eilat	^{1}H NMR (270 MHz, $CDCl_3$, δ/ppm, *J*/Hz): 3.79 (d, 6.5, H-4), 3.64 (dd, 10.9 and 4.4, H-7), 5.34 (t, 7.0, H-13), 0.96 (s, Me(24)), 1.26 (s, Me(25)), 1.11 (s, Me(26)), 1.21 (s, Me(27)), 1.13 (d, 7.2, Me(28)), 1.20 (s, Me(29)), 0.88 (s, Me(30)) and 0.89 (s, Me(31)). ^{13}C NMR (75 MHz, $CDCl_3$): 140.05 (s, C-14), 133.76 (d, C-13), 77.95 (s, C-5), 77.05 (d, C-4), 76.48 (d, C-7), 73.58 (s, H-19), 72.56 (s, C-10), 58.37 (d, C-11), 50.84 (d, C-18), 50.12 (d, H-22), 42.90 (s, C-1), 40.80 (t, C-9), 37.24 (t), 35.23 (t), 33.85 (d, C-15), 33.55 (t), 33.35 (s, H-23), 32.17 (t), 29.93 (q), 29.06 (q), 28.85 (q), 28.76 (t), 26.30 (q), 25.64 (t), 24.00 (t), 23.49 (q), 22.35 (q), 21.51 (q), 21.41 (t) and 13.15 (q, H-24). [61]
Sipholenol D (**63**)<br>$C_{30}H_{50}O_5$<br>Sipholane triterpenoid	*Callyspongia siphonella*<br>Gulf of Eilat	^{1}H NMR (270 MHz, $CDCl_3$, δ/ppm, *J*/Hz): 3.83 (d, 6.5, H-4), 3.54 (dd, 11.7 and 4.8, H-7), 0.98 (s, Me(24)), 1.25 (s, Me(25)), 1.13 (s, Me(26)), 1.22 (s, Me(27)), 1.89 (s, Me(28)), 1.18 (s, Me(29)), 0.75 (s, Me(30)) and 1.00 (s, Me(31)). [61]
Sipholenol E (**64**)<br>$C_{30}H_{52}O_5$<br>Sipholane triterpenoid	*Callyspongia siphonella*<br>Gulf of Eilat	^{13}C NMR (75 MHz, $CDCl_3$): 42.84 (s, C-1), 79.27 (C-4), 77.29 (s, C-5), 76.87 (d, C-7), 39.45 (t, C-9), 72.56 (s, C-10), 55.99 (d, C-11), 163.51 (C-14), 120.36 (C-15), 71.93 (C-16), 58.75 (d, C-18), 70.49 (s, C-19), 55.99 (d, C-22), 31.55 (s, C-23), 13.30 (q, C-24) and 19.23 (C-28). [61]
Sipholenol F (**65**)<br>$C_{30}H_{52}O_4$<br>Sipholane triterpenoid	*Callyspongia siphonella*<br>Eritrea and Gulf of Eilat	^{1}H NMR (500 MHz, $CDCl_3$, δ/ppm, *J*/Hz): 1.47 (m, H-2), 1.56 (m, H-2), 1.71 (m, H-3), 1.98 (m, H-3), 3.81 (d, 6.7, H-4), 3.52 (dd, 4.4 and 11.7, H-7), 1.39 (m, H-8), 1.79 (m, H-8), 1.51 (m, H-9), 1.63 (m, H-9), 0.94 (m, H-11), 1.52 (m, H-12), 1.57 (m, H-12), 1.35 (m, H-13), 1.88 (m, H-13), 2.08 (m, H-14), 1.06 (m, H-16), 1.75 (m, H-16), 1.31 (m, H-17), 1.72 (m, H-17), 2.46 (m, H-18), 5.25 (brs, H-20), 1.70 (m, H-21), 1.82 (m, H-21), 1.90 (m, H-22), 1.00 (s, Me-24), 1.27 (s, Me-25), 1.13 (s, Me-26), 1.16 (s, Me-27), 1.17 (s, Me-28), 1.73 (s, Me-

		29), 0.90 (s, Me-30) and 1.09 (s, Me-31). ^{13}C NMR (125 MHz, $CDCl_3$): 42.8 (C-1), 34.4 (C-2), 25.3 (C-3), 77.0 (C-4), 77.8 (C-5), 76.4 (C-7), 26.7 (C-8), 39.2 (C-9), 72.4 (C-10), 56.6 (C-11), 27.5 (C-12), 29.6 (C-13), 42.0 (C-14), 72.9 (C-15), 39.7 (C-16), 30.5 (C-17), 47.1 (C-18), 135.6 (C-19), 121.7 (C-20), 36.2 (C-21), 47.6 (C-22), 34.7 (C-23), 13.3 (C-24), 21.3 (C-25), 28.9 (C-26), 30.3 (C-27), 28.4 (C-28), 21.8 (C-29), 24.6 (C-30) and 35.5 (C-31). [53]
Sipholenol G (**66**)<br>$C_{30}H_{50}O_6$<br>Sipholane triterpenoid	*Callyspongia siphonella*<br>Gulf of Eilat	^{1}H NMR (500 MHz, $CDCl_3$, δ/ppm, *J*/Hz): 3.77 (d, 6.6, H-4), 3.52 (dd, 11.9 and 3.8, H-7), 2.94 (t, 7.5, H-16), 1.01 (s, Me-24), 1.24 (s, Me-25), 1.13 (s, Me-26), 1.22 (s, Me-27), 1.38 (s, Me-28), 1.27 (s, Me-29), 1.04 (s, Me-30) and 1.26 (s, Me-31). ^{13}C NMR (125 MHz, $CDCl_3$): 42.6 (C-1), 34.0 (C-2), 25.2 (C-3), 76.6 (C-4), 77.9 (C-5), 76.6 (C-7), 26.6 (C-8), 39.0 (C-9), 71.8 (C-10), 55.8 (C-11), 27.6 (C-12), 33.1 (C-13), 56.0 (C-14), 60.8 (C-15), 61.9 (C-16), 30.4 (C-17), 46.9 (C-18), 81.3 (C-19), 37.0 (C-20), 24.0 (C-21), 52.9 (C-22), 37.1 (C-23), 12.8 (C-24), 21.2 (C-25), 28.9 (C-26), 30.0 (C-27), 30.4 (C-28), 25.3 (C-29), 28.1 (C-30) and 33.4 (C-31). [53]
Sipholenol H (**67**)<br>$C_{30}H_{52}O_5$<br>Sipholane triterpenoid	*Callyspongia siphonella*<br>Eritrea and Gulf of Ei-lat	^{1}H NMR (500 MHz, $CDCl_3$, δ/ppm, *J*/Hz): 3.75 (d, 6.7, H-4), 3.57 (dd, 4.8 and 12.0, H-7), 0.91 (br d, H-11), 2.06 (dt, 6 and 12.0, H-13), 2.52 (dt, 8.4 and 12.0, H-13′), 4.23 (br d, 5.7, H-16), 1.79 (ddd, 5.7, 13.5 and 14.3, H-17), 1.71 (ddd, 4.2, 4.6 and 14.3, H-18), 2.39 (br d, 4.6, H-22), 0.94 (s, Me-24), 1.25 (s, Me-25), 1.12 (s, Me-26), 1.20 (s, Me-27), 1.86 (s, Me-28), 1.23 (s, Me-29), 0.74 (s, Me-30) and 1.00 (s, Me-31). ^{13}C NMR (125 MHz, $CDCl_3$): 42.4 (C-1), 34.2 (C-2), 25.0 (C-3), 76.4 (C-4), 78.2 (C-5), 76.4 (C-7), 26.3 (C-8), 38.8 (C-9), 72.0 (C-10), 55.2 (C-11), 24.8 (C-12), 37.9 (C-13), 125.8 (C-14), 143.0 (C-15), 83.0 (C-16), 27.4 (C-17), 42.1 (C-18), 72.0 (C-19), 30.3 (C-20), 36.1 (C-21), 44.7 (C-22), 34.4 (C-23), 12.5 (C-24), 21.1 (C-25), 28.7 (C-26), 29.9 (C-27), 16.7 (C-28), 28.3 (C-29), 23.2 (C-30) and 35.3 (C-31). [53]
Sipholenol I (**68**)<br>$C_{30}H_{52}O_6$<br>Sipholane triterpenoid	*Callyspongia siphonella*<br>Egypt	^{1}H NMR (400 MHz, $CDCl_3$, δ/ppm, *J*/Hz): 1.46 (H-2), 1.64 (m, H-2), 1.68 (H-3), 2.02 (m, H-3), 3.79 (d, 6.6, H-4), 3.48 (dd, 11.7 and 4.4, H-7), 1.37 (H-8), 1.74 (m, H-8), 1.46 (H-9), 1.64 (m, H-9), 0.83 (m, H-11), 1.55 (H-12), 1.63 (m, H-12), 1.50 (m, H-13), 2.22 (ddd, 14.3, 12.4 and 7.3, H-13), 3.99 (dd, 9.9 and 6.6, H-16), 1.28

		(H-17), 2.01 (m, H-17), 1.84 (br m, H-18), 1.36 (H-20), 1.60 (m, H-20), 1.21 (H-21), 1.79 (m, H-21), 2.48 (d, 4.4, H-22), 1.03 (s, H-24), 1.25 (s, H-25), 1.12 (s, H-26), 1.15 (s, H-27), 1.42 (s, H-28), 1.14 (s, H-29), 1.00 (s, H-30) and 1.24 (s, H-31). ^{13}C NMR (100 MHz, $CDCl_3$): 42.9 (C-1), 34.5 (C-2), 25.3 (C-3), 77.2 (C-4), 77.8 (C-5), 76.8 (C-7), 26.8 (C-8), 39.3 (C-9), 72.7 (C-10), 55.7 (C-11), 21.0 (C-12), 36.7 (C-13), 70.0 (C-14), 65.7 (C-15), 69.5 (C-16), 31.3 (C-17), 39.2 (C-18), 72.2 (C-19), 30.2 (C-20), 38.0 (C-21), 40.2 (C-22), 31.8 (C-23), 13.4 (C-24), 21.4 (C-25), 29.2 (C-26), 29.5 (C-27), 16.8 (C-28), 30.4 (C-29), 25.4 (C-30) and 35.6 (C-31). [59]
Sipholenol J (**69**)<br>$C_{30}H_{50}O_5$<br>Sipholane triterpenoid	*Callyspongia siphonella*<br>Egypt	^{1}H NMR (400 MHz, $CDCl_3$, δ/ppm, *J*/Hz): 1.48 (H-2), 1.62 (m, H-2), 1.73 (m, H-3), 2.00 (m, H-3), 3.80 (dd, 6.6 and 3.0, H-4), 3.53 (ddd, 11.9 and 4.4, H-7), 1.37 (m, H-8), 1.71 (m, H-8), 1.54 (m, H-9), 1.07 (m, H-11), 2.02 (m, H-12), 2.51 (m, H-12), 5.28 (ddt, 9.6 and 2.6, H-13), 3.39 (m, H-15), 2.40 (ddd, 14.3 and 5.5, H-17), 2.47 (m, H-17), 1.79 (m, H-18), 1.49 (m, H-20), 1.79 (m, H-20), 1.30 (m, H-21), 1.92 (m, H-21), 3.17 (dd, 5.1, H-23), 0.99 (s, H-24), 1.12 (s, H-25), 1.25 (s, H-26), 0.93 (s, H-27), 1.15 (dd, 6.6, H-28), 1.16 (s, H-29), 1.00 (s, H-30) and 1.16 (s, H-31). ^{13}C NMR (100 MHz, $CDCl_3$): 42.7 (C-1), 34.6 (C-2), 25.4 (C-3), 77.0 (C-4), 77.9 (C-5), 76.3 (C-7), 26.7 (C-8), 39.7 (C-9), 72.4 (C-10), 56.3 (C-11), 25.7 (C-12), 131.0 (C-13), 136.5 (C-14), 49.9 (C-15), 211.0 (C-16), 42.4 (C-17), 47.6 (C-18), 71.9 (C-19), 31.2 (C-20), 37.1 (C-21), 35.3 (C-22), 41.5 (C-23), 13.3 (C-24), 29.2 (C-25), 21.5 (C-26), 30.7 (C-27), 12.3 (C-28), 29.1 (C-29), 32.9 (C-30) and 26.4 (C-31). [52]
Sipholenol K (**70**)<br>$C_{30}H_{50}O_5$<br>Sipholane triterpenoid	*Callyspongia siphonella*<br>Egypt	^{1}H NMR (400 MHz, $CDCl_3$, δ/ppm, *J*/Hz): 1.44 (m, H-2), 1.70 (m, H-3), 2.00 (m, H-3), 3.81 (dd, 6.6, H-4), 3.53 (ddd, 11.9 and 4.4, H-7), 1.39 (m, H-8), 1.71 (m, H-8), 1.52 (m, H-9), 1.60 (m, H-9), 0.99 (m, H-11), 1.48 (m, H-12), 1.63 (m, H-12), 2.15 (m, H-13), 2.73 (m, H-13), 2.33 (m, H-17), 2.07 (m, H-18), 1.41 (m, H-20), 1.26 (m, H-21), 1.81 (ddd, 13.6 and 3.3, H-21), 2.69 (dd, 4.8, H-23), 0.97 (s, H-24), 1.11 (s, H-25), 1.25 (s, H-26), 1.20 (s, H-27), 1.87 (s, H-28), 1.16 (s, H-29), 0.98 (s, H-30) and 0.73 (s, H-31). ^{13}C NMR (100 MHz, $CDCl_3$): 42.9 (C-1), 34.7 (C-2), 25.3 (C-3), 77.0 (C-4), 77.9 (C-5), 76.3 (C-7), 26.6 (C-8), 39.4 (C-9), 72.3 (C-

		10), 56.2 (C-11), 24.7 (C-12), 40.0 (C-13), 163.1 (C-14), 133.0 (C-15), 199.7 (C-16), 38.2 (C-17), 45.3 (C-18), 71.6 (C-19), 30.5 (C-20), 35.9 (C-21), 35.6 (C-22), 46.6 (C-23), 13.1 (C-24), 29.1 (C-25), 21.4 (C-26), 30.5 (C-27), 11.8 (C-28), 28.7 (C-29), 34.5 (C-30) and 22.7 (C-31). [52]
Sipholenol L (**71**)<br>$C_{31}H_{54}O_3$<br>Sipholane triterpenoid	*Callyspongia siphonella*<br>Saudi Arabia	^{1}H NMR (600 MHz, $CDCl_3$, δ/ppm, *J*/Hz): 1.54 (1H, m, H-2), 1.46 (1H, m, H-2), 2.01 (1H, dd, 12.6 and 2.4, H-3), 1.71 (1H, m, H-3), 3.81 (1H, d, 6.6, H-4), 3.51 (1H, dd, 12 and 4.2, H-7), 1.72 (1H, m, H-8), 1.39 (1H, m, H-8), 1.62 (1H, m, H-9), 1.50 (1H, m, H-9), 0.92 (1H, m, H-11), 1.51 (1H, m, H-12), 1.49 (1H, m, H-12), 1.78 (1H, m, H-13), 1.72 (1H, m, H-13), 2.45 (1H, m, H-14), 5.26 (1H, br d, 9.6, H-16), 2.10 (1H, m, H-17), 1.86 (1H, m, H-17), 1.90 (H-18), 1.72 (1H, m, H-20), 1.25 (1H, m, H-20), 1.73 (1H, m, H-21), 1.06 (1H, m, H-21), 2.06 (1H, dd, 4.2 and 3.6, H-23), 1.00 (3H, s, H-24), 1.13 (3H, s, H-25), 1.27 (3H, s, H-26), 1.15 (3H, s, H-27), 1.77 (br s, H-28), 1.17 (3H, s, H-29), 1.08 (3H, s, H-30) and 0.89 (3H, s, H-31). ^{13}C NMR (150 MHz, $CDCl_3$): 42.7 (C-1), 34.2 (C-2), 25.1 (C-3), 77.0 (C-4), 77.8 (C-5), 76.4 (C-7), 26.6 (C-8), 39.2 (C-9), 72.4 (C-10), 56.6 (C-11), 27.5 (C-12), 36.3 (C-13), 47.0 (C-14), 135.6 (C-15), 121.7 (C-16), 28.9 (C-17), 47.6 (C-18), 72.9 (C-19), 29.7 (C-20), 39.6 (C-21), 34.8 (C-22), 41.9 (C-23), 13.2 (C-24), 29.1 (C-25), 21.4 (C-26), 30.3 (C-27), 21.9 (C-28), 28.5 (C-29), 35.5 (C-30) and 24.7 (C-31). [55]
Sipholenol L (**72**)<br>$C_{30}H_{52}O_4$<br>Sipholane triterpenoid	*Callyspongia siphonella*<br>Saudi Arabia	^{1}H NMR (500 MHz, $CDCl_3$, δ/ppm, *J*/Hz): 0.87 (1H, s, H-31), 0.90 (1H, m, H-11), 0.98 (1H, s, H-24), 1.04 (1H, m, H-21), 1.07 (1H, s, H-30), 1.10 (1H, s, H-25), 1.13 (1H, s, H-27), 1.15 (1H, s, H-29), 1.25 (1H, s, H-26), 1.30 (1H, m, H-20), 1.38 (1H, m, H-8), 1.44 (1H, m, H-2), 1.48 (1H, m, H-12), 1.52 (1H, m, H-9), 1.59 (1H, m, H-2′), 1.70 (1H, m, H-3), 1.72 (1H, m, H-8′), 1.73 (1H, m, H-21′), 1.74 (1H, m, H-20′), 1.75 (1H, m, H-13), 1.75 (1H, s, H-28), 1.80 (1H, m, H-17), 1.86 (1H, m, H-18), 1.99 (1H, m, H-3′), 2.05 (1H, m, H-23), 2.10 (1H, m, H-17), 2.44 (1H, br s, H-14), 3.49 (1H, dd, 12.0 and 4.5, H-7), 3.80 (1H, d, 7.0, H-4) and 5.26 (1H, br s, H-16). ^{13}C NMR (125 MHz, $CDCl_3$): 13.3 (C-24), 21.4 (C-26), 22.0 (C-28), 24.8 (C-31), 25.3 (C-3), 26.8 (C-8), 27.6 (C-12), 28.6 (C-29), 29.2 (C-17, C-25), 30.5 (C-20, C-

		27), 34.4 (C- 2), 34.9 (C-22), 35.6 (C-30), 36.4 (C-13), 39.3 (C-9), 39.8 (C-21), 42.1 (C-23), 42.9 (C-1), 47.2 (C-14), 47.7 (C-18), 56.7 (C-11), 72.4 (C-10), 73.0 (C-19), 76.8 (C-7), 77.0 (C-4), 77.9 (C-5), 121.8 (C-16) and 135.7 (C-15). [56]
	Callyspongia siphonella<br>Saudi Arabia	Spectroscopic data not provided (Reference 52 is cited). [8]
	Callyspongia siphonella<br>Egypt	^{1}H NMR (400 MHz, $CDCl_3$, δ/ppm, *J*/Hz): 1.44 (H-2), 1.59 (m, H-2), 1.70 (m, H-3), 1.99 (m, H-3), 3.80 (d, 6.6, H-4), 3.49 (dd, 11.9 and 4.4, H-7), 1.38 (m, H-8), 1.71 (m, H-8), 1.52 (m, H-9), 0.91 (m, H-11), 1.48 (m, H-12), 1.76 (m, H-13), 2.44 (br s, H-14), 5.24 (br s, H-16), 1.80 (m, H-17), 2.10 (m, H-17), 1.86 (m, H-18), 1.30 (m, H-20), 1.73 (m, H-20), 1.03 (m, H-21), 1.73 (m, H-21), 2.04 (m, H-23), 0.98 (s, H-24), 1.11 (s, H-25), 1.25 (s, H-26), 1.13 (s, H-27), 1.75 (s, H-28), 1.15 (s, H-29), 1.07 (s, H-30) and 0.87 (s, H-31). ^{13}C NMR (100 MHz, $CDCl_3$): 42.8 (C-1), 34.3 (C-2), 25.2 (C-3), 77.0 (C-4), 77.9 (C-5), 76.5 (C-7), 26.7 (C-8), 39.3 (C-9), 72.5 (C-10), 56.7 (C-11), 27.6 (C-12), 36.4 (C-13), 47.2 (C-14), 135.7 (C-15), 121.8 (C-16), 29.1 (C-17), 47.7 (C-18), 73.0 (C-19), 30.5 (C-20), 39.7 (C-21), 34.8 (C-22), 42.0 (C-23), 13.3 (C-24), 29.2 (C-25), 21.5 (C-26), 30.4 (C-27), 22.1 (C-28), 28.6 (C-29), 35.6 (C-30) and 24.8 (C-31). [52]
Sipholenol M (**73**)<br>$C_{30}H_{52}O_6$<br>Sipholane triterpenoid	*Callyspongia siphonella*<br>Egypt	^{1}H NMR (400 MHz, CD_3OD, δ/ppm, *J*/Hz): 1.56 (m, H-2), 1.67 (m, H-3), 1.96 (m, H-3), 3.70 (d, 7.0, H-4), 3.57 (dd, 11.7 and 4.0, H-7), 1.29 (m, H-8), 1.76 (m, H-8), 1.48 (m, H-9), 1.62 (m, H-9), 0.83 (m, H-11), 1.45 (m, H-12), 2.05 (m, H-13), 2.49 (m, H-13), 4.43 (dd, 7.7 and 7.7, H-16), 1.88 (m, H-17), 2.04 (m, H-17), 1.64 (m, H-18), 1.40 (m, H-20), 1.58 (m, H-20), 1.16 (m, H-21), 1.82 (m, H-21), 2.49 (d, 4.8, H-23), 0.98 (s, H-24), 1.09 (s, H-25), 1.22 (s, H-26, H-27), 1.82 (s, H-28), 1.17 (s, H-29), 0.99 (s, H-30) and 0.90 (s, H-31). ^{13}C NMR (100 MHz, CD_3OD): 42.9 (C-1), 34.4 (C-2), 25.2 (C-3), 76.0 (C-4), 78.5 (C-5), 76.7 (C-7), 26.5 (C-8), 38.8 (C-9), 71.6 (C-10), 55.4 (C-11), 23.8 (C-12), 36.5 (C-13), 142.5 (C-14), 127.0 (C-15), 84.9 (C-16), 29.4 (C-17), 46.4 (C-18), 71.8 (C-19), 30.3 (C-20), 38.4 (C-21), 33.3 (C-22), 44.2 (C-23), 12.3 (C-24), 28.1 (C-25), 20.8 (C-26), 29.3 (C-27), 15.3 (C-28), 27.9 (C-29), 35.2 (C-30) and 23.6 (C-31). [52]

Sipholenol N (**74**)<br>$C_{30}H_{52}O_5$<br>Sipholane triterpenoid	*Callyspongia siphonella*<br>Egypt	^{1}H NMR (600 MHz, $CDCl_3$, δ/ppm, *J*/Hz): 1.38 (m, H-2α), 1.62 (m, H-2β), 1.75 (m, H-3α), 1.95 (m, H-3β), 3.79 (d, 7.2, H-4), 3.32 (d, 9.6, H-7), 3.66 (ddd, 9.6, 4.8 and 2.4, H-8α), 1.95 (m, H-9α), 1.41 (m, H-9β), 0.90 (m, H-11), 1.15 (m, H-12a), 1.47 (m, H-12b), 1.76 (m, H-13a), 1.95 (m, H-13b), 1.59 (m, H-14), 5.44 (dd, 9.0 and 4.8, H-16), 1.72 (m, H-17α), 1.77 (m, H-17β, H-18), 1.55 (m, H-20α), 1.63 (m, H-20β), 1.65 (m, H-21α), 1.95 (m, H-21β), 2.45 (m, H-22), 0.98 (s, H-24), 1.16 (s, H-25), 1.30 (s, H-26), 1.12 (s, H-27), 1.73 (s, H-28), 1.22 (s, H-29), 1.00 (s, H-30) and 1.06 (s, H-31). ^{13}C NMR (150 MHz, $CDCl_3$): 43.3 (C-1), 34.2 (C-2), 24.9 (C-3), 76.5 (C-4), 78.6 (C-5), 82.4 (C-7), 66.5 (C-8), 45.5 (C-9), 73.8 (C-10), 55.7 (C-11), 26.6 (C-12), 33.7 (C-13), 57.6 (C-14), 143.0 (C-15), 121.4 (C-16), 24.7 (C-17), 48.8 (C-18), 82.1 (C-19), 37.2 (C-20), 25.0 (C-21), 52.7 (C-22), 35.4 (C-23), 13.9 (C-24), 29.0 (C-25), 23.0 (C-26), 30.1 (C-27), 30.2 (C-28), 25.6 (C-29), 29.5 (C-30) and 31.6 (C-31). [57]
Sipholenol O (**75**)<br>$C_{30}H_{52}O_6$<br>Sipholane triterpenoid	*Callyspongia siphonella*<br>Egypt	^{1}H NMR (600 MHz, $CDCl_3$, δ/ppm, *J*/Hz): 1.54 (m, H-2α), 1.39 (m, H-2β), 1.55 (m, H-3α), 1.69 (m, H-3β), 3.78 (d, 6.6, H-4), 3.47 (dd, 12.0 and 4.2, H-7), 1.70 (m, H-8α), 1.32 (m, H-8β), 1.57 (m, H-9α), 1.43 (m, H-9β), 0.90 (dd, 4.8 and 1.8, H-11), 1.02 (m, H-12a), 1.55 (m, H-12b), 1.70 (m, H-13a), 1.78 (m, H-13b), 1.92 (dd, 11.4 and 1.8, H-14), 4.47 (dd, 11.4 and 5.4, H-16), 1.50 (m, H-17α), 1.97 (m, H-17β), 1.75 (m, H-18), 1.57 (m, H-20α), 1.65 (m, H-20β), 1.67 (m, H-21α), 1.98 (m, H-21β), 2.37 (m, H-22), 0.96 (s, H-24), 1.11 (s, H-25), 1.24 (s, H-26), 1.16 (s, H-27), 5.07 (d, 1.8, H-28), 5.26 (d, 1.8, H-28), 1.27 (s, H-29), 0.92 (s, H-30) and 1.01 (s, H-31). ^{13}C NMR (150 MHz, $CDCl_3$): 42.8 (C-1), 34.1 (C-2), 25.3 (C-3), 77.1 (C-4), 77.7 (C-5), 76.5 (C-7), 26.7 (C-8), 39.4 (C-9), 72.4 (C-10), 55.3 (C-11), 27.0 (C-12), 32.5 (C-13), 60.2 (C-14), 145.8 (C-15), 88.5 (C-16), 27.7 (C-17), 48.1 (C-18), 81.9 (C-19), 38.1 (C-20), 25.1 (C-21), 50.5 (C-22), 36.9 (C-23), 12.9 (C-24), 29.1 (C-25), 21.3 (C-26), 30.5 (C-27), 121.0 (C-28), 25.3 (C-29), 26.7 (C-30) and 33.7 (C-31). [57]
Sipholenone A (15-sipholen-10,19-diol-4-one) (**76**)<br>$C_{30}H_{50}O_4$<br>Triterpene	*Callyspongia siphonella*<br>Gulf of Eilat	^{1}H NMR (270 MHz, $CDCl_3$, δ/ppm, *J*/Hz): 1.02, 1.09, 1.15, 1.25, 1.26, 1.31, 1.76 (br s), 2.30 (1H, dd, 11.4 and 3.8), 3.19 (1H, ddd, 13.0, 10.8 and 2.4) and 5.46 (1H, m). [25]

	Callyspongia siphonella Gulf of Eilat	^{1}H NMR (270 MHz, $CDCl_3$, δ/ppm, *J*/Hz): 3.19 (ddd, 13.0, 11.0 and 2.4, H-3), 2.10 (ddd, 11.0, 6.3 and 1.8, H-3), 2.90 (dd, 11.4 and 3.8, H-7), 5.46 (br dd, 8.5 and 4.8, H-16), 1.02 (s, Me(24)), 1.31 (s, Me(25)), 1.26 (s, Me(26)), 1.25 (s, Me(27)), 1.76 (br s, Me(28)), 1.15 (s, Me(29)) and 1.09 (s, Me(30), Me(31)). ^{13}C NMR (75 MHz, $CDCl_3$): 218.60 (s, C-4), 143.02 (s, C-15), 121.57 (d, C-16), 82.44 (s, C-5), 81.99 (s, C-19), 81.24 (d, C-7), 72.23 (s, C-10), 57.70 (d, C-14), 55.69 (d, C-11), 52.78 (d, C-18), 48.80 (d, C-22), 42.09 (s, C-1), 39.69 (t), 39.18 (t, C-9), 37.33 (t), 35.50 (s, C-23), 35.02 (q), 33.82 (t), 31.67 (q), 30.08 (q), 30.05 (q), 29.45 (q), 26.90 (t), 26.69 (t), 26.54 (t), 25.55 (q), 25.04 (t), 24.80 (t), 20.45 (q) and 12.22 (q, C-24). [61]
	Callyspongia siphonella Egypt	^{1}H NMR (400 MHz, $CDCl_3/CD_3OD$ 4:1, δ/ppm): 1.21 (H-2), 1.90 (H-2), 2.12 (H-3), 3.24 (H-3), 2.96 (H-7), 1.47 (H-8), 1.94 (H-8), 1.45 (H-9), 1.66 (H-9), 0.71 (H-11), 1.15 (H-12), 1.62 (H-12), 1.73 (H-13), 1.99 (H-13), 1.61 (H-14), 5.47 (H-16), 2.03 (H-17), 1.79 (H-17), 1.82 (H-18), 1.57 (H-20), 1.60 (H-20), 1.79 (H-21), 1.98 (H-21), 2.49 (H-22), 1.16 (H-24), 1.31 (H-25), 1.26 (H-26), 1.099 (H-27), 1.77 (H-28), 1.23 (H-29), 1.03 (H-30) and 1.097 (H-31). ^{13}C NMR (100 MHz, $CDCl_3/CD_3OD$ 4:1): 41.50 (s, C-1), 39.02 (t, C-2), 34.54 (t, C-3), 218.40 (s, C-4), 82.02 (s, C-5), 81.04 (d, C-7), 25.97 (t, C-8), 38.37 (t, C-9), 71.39 (s, C-10), 55.21 (d, C-11), 26.26 (t, C-12), 33.38 (t, C-13), 57.06 (d, C-14), 142.50 (s, C-15), 120.93 (d, C-16), 24.16 (t, C-17), 47.76 (d, C-18), 81.13 (s, C-19), 36.32 (t, C-20), 24.54 (t, C-21), 52.09 (d, C-22), 34.88 (s, C-23), 11.46 (q, C-24), 19.86 (q, C-25), 26.02 (q, C-26), 28.99 (q, C-27), 29.41 (q, C-28), 24.49 (q, C-29), 28.89 (q, C-30) and 31.00 (q, C-31). [58,64[1]]
	Callyspongia siphonella Egypt	Spectroscopic data not provided (References 61 and 64 are cited). [59]
	Callyspongia siphonella Egypt	Spectroscopic data not provided (Reference 61 is cited). [7]
	Callyspongia siphonella Saudi Arabia	^{1}H NMR (600 MHz, $CDCl_3$, δ/ppm, *J*/Hz): 1.87 (1H, m, H-2), 1.21 (1H, t, 13.8, H-2), 3.22 (1H, ddd, 13.8, 10.8 and 3, H-3), 2.13 (1H, ddd, 6.6, 4.2 and 1.8, H-3), 2.93 (1H, dd, 12 and 4.2, H-7), 1.90 (1H, m, H-8), 1.46 (1H, m, H-8), 1.66 (1H, m, H-9), 1.44 (1H, m, H-9), 0.72 (1H, dd, 4.2 and 1.8, H-11), 1.55 (1H, m, H-12), 1.16 (1H, m, H-12), 1.90 (1H, m, H-13), 1.81 (1H, dt,

		12.6 and 4.2, H-13), 1.61 (1H, m, H-14), 5.47 (2H, br dd, 6.0 and 1.8, H-16), 2.00 (1H, m, H-17), 1.77 (1H, m, H-17), 2.49 (1H, dd, 6.0 and 1.8, H-18), 1.96 (1H, m, H-20), 1.71 (1H, m, H-20), 1.65 (1H, m, H-21), 1.58 (1H, m, H-21), 1.79 (1H, m, H-22), 1.15 (3H, s, H-24), 1.31 (3H, s, H-25), 1.27 (3H, s, H-26), 1.10 (3H, s, H-27), 1.76 (br s, H-28), 1.25 (3H, s, H-29), 1.02 (3H, s, H-30) and 1.09 (3H, s, H-31). ^{13}C NMR (150 MHz, $CDCl_3$): 41.9 (C-1), 39.5 (C-2), 34.9 (C-3), 218.2 (C-4), 82.4 (C-5), 81.1 (C-7), 26.4 (C-8), 39.0 (C-9), 72.2 (C-10), 55.5 (C-11), 26.8 (C-12), 24.9 (C-13), 57.4 (C-14), 142.9 (C-15), 121.4 (C-16), 24.7 (C-17), 52.6 (C-18), 82.1 (C-19), 33.7 (C-20), 37.1 (C-21), 48.7 (C-22), 35.4 (C-23), 12.1 (C-24), 20.4 (C-25), 26.6 (C-26), 29.9 (C-27), 30.2 (C-28), 25.6 (C-29), 29.4 (C-30) and 31.6 (C-31). [55]
	Callyspongia siphonella<br>Saudi Arabia	^{1}H NMR (500 MHz, $CDCl_3$, δ/ppm, *J*/Hz): 0.84 (1H, m, H-11), 1.02 (3H, s, H-24), 1.09 (3H, s, H-30), 1.10 (3H, s, H-31), 1.15 (3H, s, H-29), 1.25 (3H, s, H-27), 1.24 (1H, m, H-2), 1.26 (3H, s, H-26), 1.30 (3H, s, H-25), 1.50 (1H, m, H-12), 1.52 (1H, m, H-9), 1.62 (1H, m, H-20), 1.68 (1H, m, H-8), 1.70 (1H, m, H-17), 1.73 (1H, m, H-21), 1.76 (3H, br s, H-28), 1.77 (1H, m, H-18), 1.82 (1H, m, H-13), 1.83 (1H, m, H-2′), 1.93 (1H, m, H-13′, H-21′), 2.10 (1H, ddd, 11.0, 6.3 and 1.8, H-3), 2.11 (1H, m, H-14), 2.49 (1H, m, H-22), 2.91 (1H, dd, 11.5 and 4.0, H-7), 3.21 (1H, ddd, 13.0, 11.0 and 2.4, H-3′) and 5.45 (1H, br dd, 8.5 and 5.0, H-16). ^{13}C NMR (125 MHz, $CDCl_3$): 12.2 (C-24), 20.5 (C-25), 24.8 (C-29), 25.1 (C-17), 26.5 (C-12), 26.7 (C-8), 29.5 (C-26), 30.0 (C-27), 30.1 (C-28), 31.2 (C-31), 31.6 (C-30), 33.8 (C-13), 35.1 (C-3), 35.5 (C-23), 37.3 (C-20), 39.2 (C-9), 39.7 (C-2), 42.1(C-1), 48.9 (C-18), 52.8 (C-22), 55.7 (C-11), 57.7 (C-14), 72.3 (C-10), 81.2 (C-7), 82.0 (C-19), 82.5 (C-5), 121.5 (C-16), 143.0 (C-15) and 217.7 (C-4). [56]
	Callyspongia siphonella<br>Saudi Red Sea territorial water	Spectroscopic data not provided (Reference 25 is cited). [60]
	Callyspongia siphonella<br>Saudi Arabia	Spectroscopic data not provided (Reference 61 is cited). [8]
Sipholenone B (**77**)<br>$C_{30}H_{50}O_5$<br>Triterpene	*Callyspongia siphonella*<br>Gulf of Eilat	^{1}H NMR (270 MHz, $CDCl_3$, δ/ppm, *J*/Hz): 3.22 (ddd, 13.0, 11.0 and 2.5, H-3), 2.09 (ddd, 11.0, 6.3 and 1.8, H-3), 2.92 (dd, 12.0 and 4.3, H-7), 2.90 (t, 7.5, H-16),

		1.03 (s, Me(24)), 1.31 (s, Me(25)), 1.26 (s, Me(26)), 1.28 (s, Me(27)), 1.37 (s, Me(28)), 1.17 (s, Me(29)), 1.21 (s, Me(30)) and 1.27 (s, Me(31)). ^{13}C NMR (75 MHz, $CDCl_3$): 218.30 (s, C-4), 82.47 (s, C-5), 81.75 (s, C-19), 81.45 (d, C-7), 72.05 (s, C-10), 61.86 (d, C-16), 60.63 (s, C-15), 56.23 (d, C-14), 55.87 (d, C-11), 53.23 (d, C-18), 47.36 (d, C-22), 42.24 (s, C-1), 39.84 (t), 39.21 (t, C-9), 37.48 (t), 37.18 (s, C-23), 35.14 (q), 33.58 (t), 33.34 (q), 30.68 (q), 30.47 (q), 29.99 (q), 28.43 (t), 27.86 (t), 26.72 (t), 26.69 (t), 25.73 (q), 24.03 (t), 20.52 (q) and 12.19 (q, C-24). [61]
Sipholenone C (**78**)<br>$C_{30}H_{48}O_5$<br>Triterpene	*Callyspongia siphonella*<br>Gulf of Eilat	^{1}H NMR (270 MHz, $CDCl_3$, δ/ppm, *J*/Hz): 3.23 (ddd, 13.0, 11.0 and 2.5, H-3), 2.10 (ddd, 11.0, 6.3 and 1.8, H-3), 2.92 (dd, 11.2 and 4.0, H-7), 1.00 (s, Me(24)), 1.31 (s, Me(25)), 1.27 (s, Me(26)), 1.34 (s, Me(27)), 6.15 (d, 2.1, Me(27)), 5.22 (d, 1.8, Me(28)), 1.14 (s, Me(29)), 0.97 (s, Me(30)) and 1.12 (s, Me(31)). ^{13}C NMR (75 MHz, $CDCl_3$): 218.10 (s, C-4), 202.57 (s, C-16), 147.56 (s, C-15), 125.83 (t, C-28), 82.50 (s, C-5), 82.38 (s, C-19), 81.19 (d, C-7), 72.04 (s, C-10), 57.02 (d, C-14), 55.13 (d, C-11), 51.29 (d, C-18), 48.91 (d, C-22), 42.29 (t), 42.16 (s, C-1), 39.94 (t), 39.25 (t, C-9), 37.11 (t), 37.08 (s, C-23), 35.06 (q), 33.87 (t), 33.01 (q), 30.31 (q), 28.48 (q), 26.69 (q), 26.54 (t), 26.41 (t), 25.86 (t), 24.85 (t), 20.50 (q) and 12.23 (q, C-24). [61]
Sipholenone D (**79**)<br>$C_{30}H_{50}O_4$<br>Triterpene	*Callyspongia siphonella*<br>Eritrea and Gulf of Eilat	^{1}H NMR (500 MHz, $CDCl_3$, δ/ppm, *J*/Hz): 3.17 (ddd, 13.0, 11.0 and 2.5, H-3), 2.09 (ddd, 11.0, 6.4 and 1.8, H-3′), 2.98 (dd, 11.4 and 3.8, H-7), 2.27 (2H, m, H-12), 5.28 (t, 7.0, H-13), 2.88 (m, H-15), 1.02 (s, Me-24), 1.31 (s, Me-25), 1.25 (s, Me-26), 1.21 (s, Me-27), 1.12 (d, 7.6, Me-28), 1.24 (s, Me-29), 0.88 (s, Me-30) and 0.96 (s, Me-31). ^{13}C NMR (125 MHz, $CDCl_3$): 42.1 (C-1), 40.6 (C-2), 35.3 (C-3), 217.4 (C-4), 82.6 (C-5), 81.3 (C-7), 26.6 (C-8), 40.8 (C-9), 72.4 (C-10), 58.0 (C-11), 33.5 (C-12), 132.7 (C-13), 140.7 (C-14), 33.8 (C-15), 32.1 (C-16), 21.3 (C-17), 50.1 (C-18), 73.1 (C-19), 37.2 (C-20), 24.0 (C-21), 50.9 (C-22), 33.3 (C-23), 12.5 (C-24), 20.6 (C-25), 26.3 (C-26), 28.8 (C-27), 26.6 (C-28), 23.6 (C-29), 22.3 (C-30) and 30.0 (C-31). [53]
Sipholenone E (**80**)<br>$C_{30}H_{50}O_4$<br>Triterpene	*Callyspongia siphonella*<br>Egypt	^{1}H NMR (400 MHz, $CDCl_3$, δ/ppm, *J*/Hz): 1.25 (H-2), 1.86 (ddd, 13.9, 6.4 and 2.9, H-2), 2.10 (ddd, 10.3, 5.9 and 2.2, H-3), 3.13 (ddd, 13.6, 11.0 and 2.6, H-3), 2.95 (ddd, 10.2 and 5.5, H-7), 1.57 (m, H-8), 1.44 (m, H-9),

		1.76 (m, H-9), 1.29 (m, H-11), 2.26 (m, H-12), 5.25 (ddd, 7.0 and 7.0, H-13), 2.85 (m, H-15), 1.49 (m, H-16), 1.58 (m, H-16), 1.42 (m, H-17), 1.36 (m, H-18), 1.39 (m, H-20), 1.61 (m, H-20), 1.17 (m, H-21), 1.73 (m, H-21), 2.44 (dd, 5.5, H-23), 1.01 (s, H-24), 1.23 (s, H-25), 1.30 (s, H-26), 1.23 (s, H-27), 1.11 (dd, 7.7, H-28), 1.20 (s, H-29), 0.87 (s, H-30) and 0.94 (s, H-31). ^{13}C NMR (100 MHz, CDCl$_3$): 42.1 (C-1), 40.8 (C-2), 35.3 (C-3), 217.6 (C-4), 82.6 (C-5), 81.3 (C-7), 28.6 (C-8), 40.5 (C-9), 73.2 (C-10), 57.9 (C-11), 24.0 (C-12), 132.6 (C-13), 140.8 (C-14), 28.9 (C-15), 33.5 (C-16), 21.2 (C-17), 50.9 (C-18), 72.4 (C-19), 32.0 (C-20), 37.2 (C-21), 33.3 (C-22), 50.0 (C-23), 12.5 (C-24), 26.6 (C-25), 20.6 (C-26), 23.6 (C-27), 22.4 (C-28), 30.1 (C-29), 33.9 (C-30) and 26.4 (C-31). [52]
Sipholenoside A (**81**)<br>$C_{36}H_{60}O_8$<br>Triterpene	*Callyspongia siphonella*<br>Gulf of Eilat	^{1}H NMR (600 MHz, CDCl$_3$, δ/ppm, *J*/Hz): 3.17 (ddd, 13.3, 11.1 and 2.2, H-3), 2.10 (ddd, 11.0, 6.3 and 1.9, H-3′), 2.95 (dd, 11.9 and 4.0, H-7), 5.18 (t, 6.7, H-13), 2.81 (m, H-15), 1.03 (s, Me-24), 1.29 (s, Me-25), 1.23 (s, Me-26), 1.21 (s, Me-27), 1.11 (d, 7.6, Me-28), 1.21 (s, Me-29), 0.84 (s, Me-30), 0.97 (s, Me-31), 4.97 (br d, 1.3, H-1′), 3.70 (br s, H-2′), 3.80 (dd, 9.4 and 3.1, H-3′), 3.47 (t, 9.4, H-4′), 3.87 (dq, 9.4 and 3.6, H-5′) and 1.28 (d, 6.3, Me-6′). ^{13}C NMR (150 MHz, CDCl$_3$): 42.3 (C-1), 40.8 (C-2), 35.2 (C-3), 216.3 (C-4), 82.4 (C-5), 81.3 (C-7), 26.4 (C-8), 41.4 (C-9), 72.8 (C-10), 58.1 (C-11), 32.8 (C-12), 134.1 (C-13), 139.7 (C-14), 33.8 (C-15), 31.7 (C-16), 20.8 (C-17), 46.2 (C-18), 78.9 (C-19), 37.0 (C-20), 23.8 (C-21), 51.3 (C-22), 32.7 (C-23), 12.7 (C-24), 20.4 (C-25), 24.9 (C-26), 28.6 (C-27), 25.9 (C-28), 22.6 (C-29), 23.3 (C-30), 28.8 (C-31), 93.7 (d, C-1′), 72.5 (d, C-2′), 72.2 (d, C-3′), 74.1 (d, C-4′), 67.8 (d, C-5′) and 17.6 (q, C-6′). [53]
Sipholenoside B (**82**)<br>$C_{36}H_{62}O_8$<br>Triterpene	*Callyspongia siphonella*<br>Eritrea	^{1}H NMR (500 MHz, CDCl$_3$, δ/ppm, *J*/Hz): 1.45 (m, H-2), 1.60 (m, H-2), 1.75 (m, H-3), 1.85 (m, H-3), 3.85 (d, 6.7, H-4), 3.55 (dd, 11.9 and 6.9, H-7), 1.30 (m, H-8), 1.73 (m, H-8), 1.50 (m, H-9), 1.62 (m, H-9), 0.86 (br s, H-11), 1.22 (m, H-12), 1.50 (m, H-12), 1.79 (m, H-13), 1.95 (m, H-13), 5.49 (br d, 4.8, H-16), 2.01 (m, H-17), 1.75 (m, H-17), 1.82 (m, H-18), 1.60 (m, H-20), 1.67 (m, H-20), 1.75 (m, H-21), 1.98 (m, H-21), 2.20 (m, H-22), 0.98 (s, Me-24), 1.25 (s, Me-25), 1.14 (s, Me-26), 1.07 (s, Me-27), 1.77 (s, Me-28), 1.24 (s, Me-29), 1.04 (s,

		Me-30), 1.10 (s, Me-31), 4.95 (br s, H-1′), 3.70 (br d, 3.4, H-2′), 3.84 (dd, 9.4 and 3.4, H-3′), 3.40 (t, 9.4, H-4′), 3.85 (dq, 9.4 and 6.3, H-5′) and 1.26 (d, 6.3, Me-6′). ^{13}C NMR (125 MHz, $CDCl_3$): 42.6 (C-1), 33.6 (C-2), 25.1 (C-3), 76.5 (C-4), 77.2 (C-5), 74.1 (C-7), 26.6 (C-8), 39.0 (C-9), 72.6 (C-10), 55.7 (C-11), 26.7 (C-12), 33.6 (C-13), 57.5 (C-14), 143.3 (C-15), 121.1 (C-16), 24.2 (C-17), 45.1 (C-18), 89.3 (C-19), 33.8 (C-20), 21.4 (C-21), 52.9 (C-22), 35.4 (C-23), 12.9 (C-24), 21.3 (C-25), 29.1 (C-26), 29.5 (C-27), 30.1 (C-28), 24.9 (C-29), 31.8 (C-30), 29.8 (C-31), 94.2 (d, C-1′), 71.9 (d, C-2′), 70.5 (d, C-3′), 72.3 (d, C-4′), 67.6 (d, C-5′) and 17.4 (q, C-6′). [53]
Siphonellinol (**83**)<br>$C_{30}H_{52}O_4$<br>Triterpene	*Callyspongia siphonella*<br>Gulf of Eilat	^{1}H NMR (270 MHz, $CDCl_3$, δ/ppm, *J*/Hz): 1.05 (s, Me-31), 1.58 (s, H-24), 1.66 (s, H-25), 1.68 (s, H-30), 3.71 (1H, dd, 9.4 and 3.8, H-16) and 5.06 (1H, t, 6.2, H-22). ^{13}C NMR (75 MHz, $CDCl_3$): 135.80 (s), 128.58 (s), 124.92 (d), 77.11 (d), 76.66 (s), 76.48 (d), 72.32 (s), 71.66 (d), 56.02 (d), 43.38 (s), 42.96 (s) and 131.30 (s). [62]
Siphonellinol B (**84**)<br>$C_{30}H_{52}O_5$<br>Triterpene	*Callyspongia siphonella*<br>Gulf of Eilat	^{1}H NMR (500 MHz, $CDCl_3$, δ/ppm, *J*/Hz): 3.82 (d, 6.7, H-4), 3.53 (dd, 4.2 and 11.7, H-7), 3.66 (dd, 3.7 and 9.7, H-16), 4.01 (br t, 8.2, H-22), 4.85 (br s, H-24), 4.93 (br s, H-24), 1.71 (br s, Me-25), 1.00 (s, Me-26), 1.13 (s, Me-27), 1.27 (s, Me-28), 1.24 (s, Me-29), 1.67 (s, Me-30) and 1.06 (s, Me-31). ^{13}C NMR (125 MHz, $CDCl_3$): 41.9 (s, C-1), 33.5 (t, C-2), 24.3 (t, C-3), 75.7 (d, C-4), 77.0 (s, C-5), 75.5 (d, C-7), 25.7 (t, C-8), 38.3 (t, C-9), 71.2 (s, C-10), 54.9 (d, C-11), 25.6 (t, C-12), 29.6 (t, C-13), 134.6 (s, C-14), 127.7 (s, C-15), 70.4 (d, C-16), 25.7 (t, C-17), 31.3 (t, C-18), 42.0 (s, C-19), 31.4 (t, C-20), 27.7 (t, C-21), 74.9 (d, C-22), 146.1 (s, C-23), 110.2 (t, C-24), 16.7 (q, Me-25), 12.1 (q, Me-26), 28.0 (q, Me-27), 20.3 (q, Me-28), 29.8 (q, Me-29), 19.5 (q, Me-30), 20.6 (q, Me-31). [53]
Siphonellinol C (**85**)<br>$C_{30}H_{52}O_5$<br>Triterpene	*Callyspongia siphonella*<br>Egypt	^{1}H NMR (400 MHz, $CDCl_3$, δ/ppm, *J*/Hz): 1.38 (H-2), 1.52 (m, H-2), 1.74 (H-3), 1.98 (m, H-3), 3.81 (d, 6.6, H-4), 3.52 (dd, 12.1 and 4.4, H-7), 1.38 (H-8), 1.71 (m, H-8), 1.60 (m, H-9), 0.88 (m, H-11), 1.46 (m, H-12), 2.04 (m, H-13), 2.02 (m, H-16), 1.70 (m, H-17), 3.60 (dd, 9.6 and 2.9, H-18), 2.13 (dd, 14.6 and 6.2, H-20), 2.27 (dd, 14.6 and 8.1, H-20), 5.46 (ddd, 15.8, 8.1 and 6.2, H-21), 5.63 (d, 15.8, H-22), 1.28 (s, H-24, H-25),

		0.98 (s, H-26), 1.12 (s, H-27), 1.26 (s, H-28), 1.23 (s, H-29), 1.67 (s, H-30) and 1.06 (s, H-31). ^{13}C NMR (100 MHz, $CDCl_3$): 42.9 (C-1), 34.6 (C-2), 25.3 (C-3), 77.1 (C-4), 77.8 (C-5), 76.4 (C-7), 26.8 (C-8), 39.4 (C-9), 72.3 (C-10), 56.0 (C-11), 26.5 (C-12), 30.1 (C-13), 135.2 (C-14), 128.9 (C-15), 32.6 (C-16), 26.4 (C-17), 72.2 (C-18), 43.6 (C-19), 40.9 (C-20), 123.6 (C-21), 140.7 (C-22), 70.8 (C-23), 29.9 (C-24), 30.0 (C-25), 13.2 (C-26), 29.1 (C-27), 21.4 (C-28), 31.0 (C-29), 20.7 (C-30) and 21.1 (C-31). [59]
Siphonellinol C-23-hydroperoxide (**86**) $C_{30}H_{52}O_6$ Triterpene	*Callyspongia siphonella* Egypt	^{1}H NMR (400 MHz, $CDCl_3$, δ/ppm, *J*/Hz): 1.52 (m, H-2), 1.72 (m, H-3), 2.01 (m, H-3), 3.81 (d, 6.6, H-4), 3.52 (dd, 11.9 and 4.4, H-7), 1.38 (m, H-8), 1.71 (m, H-8), 1.60 (m, H-9), 0.88 (m, H-11), 1.46 (m, H-12), 2.07 (m, H-13), 2.01 (m, H-16), 1.68 (m, H-17), 3.61 (dd, 10.3 and 3.3, H-18), 2.13 (dd, 14.3 and 6.2, H-20), 2.35 (dd, 14.3 and 7.3, H-20), 5.61 (m, H-21), 5.53 (m, H-22), 1.29 (s, H-24), 1.30 (s, H-25), 0.98 (s, H-26), 1.12 (s, H-27), 1.26 (s, H-28), 1.23 (s, H-29), 1.67 (s, H-30) and 1.07 (s, H-31). ^{13}C NMR (100 MHz, $CDCl_3$): 43.0 (C-1), 34.6 (C-2), 25.3 (C-3), 77.1 (C-4), 77.9 (C-5), 76.4 (C-7), 26.7 (C-8), 39.5 (C-9), 72.4 (C-10), 56.0 (C-11), 26.5 (C-12), 32.6 (C-13), 135.6 (C-14), 129.0 (C-15), 30.6 (C-16), 26.9 (C-17), 72.8 (C-18), 44.0 (C-19), 41.5 (C-20), 128.9 (C-21), 135.7 (C-22), 82.1 (C-23), 24.6 (C-24, C-25), 13.2 (C-26), 29.2 (C-27), 21.4 (C-28), 31.1 (C-29), 20.7 (C-30) and 20.4 (C-31). [52]
Siphonellinol D (**87**) $C_{30}H_{52}O_4$ Triterpene	*Callyspongia siphonella* Egypt	^{1}H NMR (400 MHz, $CDCl_3$, δ/ppm, *J*/Hz): 1.56 (m, H-2), 1.71 (m, H-3), 2.01 (m, H-3), 3.81 (d, 7.0, H-4), 3.51 (dd, 12.1 and 4.4, H-7), 1.38 (m, H-8), 1.71 (m, H-8), 1.56 (m, H-9), 0.86 (m, H-11), 1.27 (m, H-12), 1.45 (m, H-12), 1.99 (H-13), 2.09 (m, H-13), 2.03 (m, H-16), 1.68 (m, H-17), 3.70 (dd, 9.5 and 3.3, H-18), 1.36 (m, H-20), 1.53 (m, H-20), 1.67 (m, H-21), 1.87 (m, H-21), 5.04 (m, H-22), 1.56 (s, H-24), 1.65 (s, H-25), 0.97 (s, H-26), 1.12 (s, H-27), 1.26 (s, H-28), 1.22 (s, H-29), 1.66 (s, H-30) and 1.04 (s, H-31). ^{13}C NMR (100 MHz, $CDCl_3$): 42.9 (C-1), 34.5 (C-2), 25.3 (C-3), 77.1 (C-4), 77.8 (C-5), 76.5 (C-7), 26.7 (C-8), 39.4 (C-9), 72.3 (C-10), 56.0 (C-11), 26.6 (C-12), 32.5 (C-13), 135.7 (C-14), 128.6 (C-15), 30.5 (C-16), 26.7 (C-17), 71.6 (C-18), 43.4 (C-19), 37.9 (C-20), 22.9 (C-21), 124.9 (C-22), 131.4 (C-23), 17.8 (C-

		24), 25.8 (C-25), 13.1 (C-26), 29.2 (C-27), 21.4 (C-28), 31.1 (C-29), 20.7 (C-30) and 21.5 (C-31). [52]
	Callyspongia siphonella Egypt	Spectroscopic data not provided (Reference 52 is cited). [57]
Siphonellinol E (**88**) $C_{30}H_{52}O_6$ Triterpene	*Callyspongia siphonella* Egypt	^{1}H NMR (400 MHz, $CDCl_3$, δ/ppm, *J*/Hz): 1.54 (m, H-2), 1.73 (m, H-3), 2.01 (m, H-3), 3.81 (d, 6.6, H-4), 3.52 (dd, 11.7 and 4.4, H-7), 1.38 (m, H-8), 1.71 (m, H-8), 1.56 (m, H-9), 1.64 (m, H-9), 0.87 (m, H-11), 1.26 (m, H-12), 1.46 (m, H-12), 1.95 (H-13), 2.12 (m, H-13), 2.02 (m, H-16), 1.69 (m, H-17), 3.60 (dd, 9.7 and 2.6, H-18), 1.48 (m, H-20), 1.26 (m, H-21), 1.39 (m, H-21), 4.22 (m, H-22), 4.99 (H-24), 5.01 (m, H-24), 1.71 (s, H-25), 0.98 (s, H-26), 1.12 (s, H-27), 1.26 (s, H-28), 1.24 (s, H-29), 1.65 (s, H-30) and 1.04 (s, H-31). ^{13}C NMR (100 MHz, $CDCl_3$): 43.0 (C-1), 34.5 (C-2), 25.3 (C-3), 77.1 (C-4), 77.9 (C-5), 76.5 (C-7), 26.9 (C-8), 39.4 (C-9), 72.3 (C-10), 55.9 (C-11), 26.7 (C-12), 32.2 (C-13), 135.4 (C-14), 129.0 (C-15), 30.5 (C-16), 26.8 (C-17), 71.6 (C-18), 43.1 (C-19), 33.2 (C-20), 25.2 (C-21), 90.2 (C-22), 143.5 (C-23), 114.6 (C-24), 17.3 (C-25), 13.2 (C-26), 29.2 (C-27), 21.4 (C-28), 31.0 (C-29), 20.7 (C-30) and 21.6 (C-31). [52]
24*S*-24-methyl-cholestane-3*β*,5*α*,6*β*,25-tetraol-25-mono acetate (**89**) $C_{30}H_{52}O_5$ Sterol	*Callyspongia fibrosa* India	Supplementary data not found [23]
24*S*-24-methyl chelestane-3*β*,5*α*,6*β*,12*β*,25-pentaol-25-*O*-acetate (**90**) $C_{30}H_{52}O_6$ Sterol	*Callyspongia fibrosa* India	Supplementary data not found [23]
24*S*-24-methyl cholest-25-ene-3*β*,5*α*,6*β*,12*β*-tetrol (**91**) $C_{28}H_{48}O_4$ Sterol	*Callyspongia fibrosa* India	Supplementary data not found [23]
24*S*-24-methyl cholestane-3*β*,6*β*,25-triol-25-*O*-acetate (**92**) $C_{30}H_{52}O_4$ Sterol	*Callyspongia fibrosa* India	Supplementary data not found [23]
24*S*-24-methyl cholestane-3*β*,6*β*,8*β*,25-tetraol-25-*O*-acetate (**93**) $C_{30}H_{52}O_5$ Sterol	*Callyspongia fibrosa* India	Supplementary data not found [23]

24*S*-24-methylcholesterol (**94**)<br>$C_{28}H_{48}O$<br>Sterol	*Callyspongia fibrosa*<br>India	^{1}H NMR* ($CDCl_3$, δ/ppm, *J*/Hz): 3.50 (br s, m, 3b-H), 0.90 (d, 6, 28-H), 5.34 (d), 0.97 (s), 0.80 (d, 6, 26-H), 0.83 (d, 6, 27-H), 0.94 (d, 6, 21-H) and 0.66 (s). [23]
5α-cholestanone (**95**)<br>$C_{27}H_{46}O$<br>Sterol	*Callyspongia siphonella*<br>Egypt	^{1}H NMR (400 MHz, $CDCl_3$, δ/ppm): 0.68 (s, H-18), 0.855 (d, H-27), 0.87 (H-26), 0.905 (H-21) and 1.01 (s, H-19). ^{13}C NMR (100 MHz, $CDCl_3$): 212.19 (C-3). [7]
Callysterol (**96**)<br>$C_{28}H_{48}O$<br>Sterol	*Callyspongia siphonella*<br>Egypt	^{1}H NMR (400 MHz, $CDCl_3$, δ/ppm): 0.61 (d, H-21), 0.94 (s, H-19), 1.76 (m, H-25), 1.93 (m, H-14), 3.46 (1H, m, H-3), 5.10 (1H, m, H-6) and 5.28 (1H, s, H-11, H-12). ^{13}C NMR (100 MHz, $CDCl_3$): 11.86 (C-21), 22.57 (C-26, C-27), 24.31 (C-23), 28.84 (C-25), 31.62 (C-1), 31.94 (C-24), 33.92 (C-13), 34.69 (C-8), 36.28 (C-2), 36.55 (C-10), 37.26 (C-16), 39.69 (C-7), 39.78 (C-22), 42.26 (C-4), 43.07 (C-14), 46.06 (C-15), 50.14 (C-9), 56.88 (C-17), 71.84 (C-3), 121.73 (C-6), 131.82 (C-12), 136.08 (C-11) and 140.78 (C-5). [7]
Callysterol (ergosta-5,11-dien-3β-ol) (**97**)<br>$C_{28}H_{46}O$<br>Sterol	*Callyspongia siphonella*<br>Egypt	^{1}H NMR (300 MHz, $CDCl_3$, δ/ppm, *J*/Hz): 1.71 (m, H-1), 1.51 (m, H-1), 1.37 (m, H-2), 1.36 (m, H-2), 3.51 (m, H-3), 2.26 (m, H-4), 2.23 (m, H-4), 5.31 (m, H-6) 2.0 (m, H-7), 1.69 (m, H-7), 1.47 (m, H-8), 0.93 (m, H-9), 5.16 (m, H-11), 5.15 (d, 7.0, H-12), 1.82 (m, H-14), 0.89 (m, H-15), 1.12 (m, H-16), 1.07 (m, H-16), 1.87 (m, H-17), 0.99 (m, H-18), 0.91 (m, H-19), 1.15 (m, H-20), 0.67 (d, 2.4, H-21), 1.17 (m, H-22), 1.14 (m, H-22), 0.97 (m, H-23), 1.13 (m, H-24), 1.67 (m, H-25), 0.80 (d, 6.1, H-26, H-27) and 0.85 (d, 6.5, H-28). ^{13}C NMR (75 MHz, $CDCl_3$): 31.6 (C-1), 36.2 (C-2), 71.8 (C-3), 42.3 (C-4), 140.7 (C-5), 121.7 (C-6), 39.6 (C-7), 34.7 (C-8), 50.1 (C-9), 36.5 (C-10), 136.0 (C-11), 131.8 (C-12), 33.9 (C-13), 43.0 (C-14), 46.0 (C-15), 37.2 (C-16), 56.8 (C-17), 11.8 (C-18), 18.8 (C-19), 55.9 (C-20), 11.8 (C-21), 39.7 (C-22), 24.3 (C-23), 31.9 (C-24), 28.8 (C-25), 22.5 (C-26, C-27) and 12.3 (C-28). [19]
Cholestenone<br>(4-cholesten-3-one) (**98**)<br>$C_{27}H_{44}O$<br>Sterol	*Callyspongia siphonella*<br>Egypt	^{1}H NMR (400 MHz, $CDCl_3$, δ/ppm): 0.71 (s, H-18), 0.86 (d, H-27), 0.86 (d, H-26), 0.91 (H-21), 1.18 (H-19), 1.84 (m, H-16), 2.03 (m, H-12) and 5.72 (s, H-4). ^{13}C NMR (400 MHz, $CDCl_3$): 123.75 (C-4), 171.63 (C-5) and 199.58 (C-3). [7]
Stigmasta-4,22-dien-3,6-dione (**99**)<br>$C_{29}H_{44}O_2$<br>Sterol	*Callyspongia siphonella*<br>Egypt	^{13}C NMR (100 MHz, $CDCl_3$, δ/ppm): 137.80 (C-22), 129.80 (C-23), 125.48 (C-4), 56.67 (C-17), 55.76 (C-14), 51.25 (C-24), 51.01 (C-9), 46.81 (C-7), 42.42 (C-13), 40.39 (C-20), 39.83 (C-10), 39.04 (C-12), 35.57 (C-1), 34.21 (C-8), 33.98 (C-2), 31.86 (C-25), 28.73 (C-16),

		25.39 (C-28), 24.03 (C-15), 21.17 (C-21), 21.09 (C-27), 20.88 (C-11), 18.99 (C-26), 17.52 (C-19), 12.24 (C-29) and 12.09 (C-18). [7]
Stigmasterone (**100**)<br>$C_{29}H_{46}O$<br>Sterol	*Callyspongia siphonella*<br>Egypt	^{1}H NMR (400 MHz, $CDCl_3$, δ/ppm): 3.22 (H-4), 3.19 (H-4), 2.76 (H-4) and 2.73 (H-4). ^{13}C NMR (100 MHz, $CDCl_3$): 210.39 (C-3), 138.47 (C-5), 138.00 (C-22), 129.39 (C-23), 122.59 (C-6), 56.72 (C-14), 55.94 (C-17), 51.25 (C-24), 49.19 (C-9), 48.37 (C-4), 42.28 (C-13), 40.49 (C-20), 39.58 (C-12), 37.66 (C-2), 36.94 (C-10), 36.87 (C-1), 31.93 (C-7), 31.88 (C-25), 31.81 (C-8), 28.91 (C-16), 25.41 (C-28), 24.35 (C-15), 21.35 (C-21), 21.23 (C-11), 21.09 (C-27), 19.20 (C-19), 18.99 (C-26), 12.25 (C-29) and 12.10 (C-18). [7]
Gelliusterol E (**101**)<br>$C_{25}H_{36}O_2$<br>Sterol	*Callyspongia implexa*<br>Egypt	^{1}H NMR (400 MHz, $CDCl_3$, δ/ppm): 1.11 (m, H-1), 1.87 (m, H-1), 1.49 (m, H-2), 1.84 (m, H-2), 3.58 (m, H-3), 2.34 (m, H-4), 5.61 (d, H-6), 3.85 (br s, H-7), 1.49 (m, H-8), 0.91 (t, H-9), 1.49 (m, H-11), 1.11 (m, H-12), 2.00 (m, H-12), 1.08 (m, H-14), 2.05 (m, H-15), 5.15 (d, H-16), 0.68 (d, H-18), 1.00 (s, H-19), 1.5 (m, H-20), 1.01 (s, H-21), 2.33 (d, H-22) and 1.69 (m, H-25). ^{13}C NMR (100 MHz, $CDCl_3$): 37.0 (C-1), 31.5 (C-2), 72.0 (C-3), 41.4 (C-4), 147.7 (C-5), 124.5 (C-6), 65.9 (C-7), 39.1 (C-8), 44.0 (C-9), 38.0 (C-10), 21.6 (C-11), 40.9 (C-12), 45.6 (C-13), 47.2 (C-14), 25.0 (C-15), 114.2 (C-16), 147.0 (C-17), 12.0 (C-18), 18.0 (C-19), 35.8 (C-20), 19.0 (C-21), 26.2 (C-22), 78.9 (C-23), 77.7 (C-24) and 3.3 (C-25). [28]
β-sitosterol (**102**)<br>$C_{29}H_{50}O$<br>Sterol	*Callyspongia implexa*<br>Egypt	Spectroscopic data not provided. [28]
Siphonocholin (**103**)<br>$C_{28}H_{48}O$<br>Sterol	*Callyspongia siphonella*<br>Saudi Arabia	Spectroscopic data not provided. [63]
Ergosta-5,24(28)-dien-3*β*-ol (**104**)<br>$C_{29}H_{48}O$<br>Sterol	*Callyspongia siphonella*<br>Saudi Arabia	Spectroscopic data not provided. [8]

* Magnetic field strength did not report in the reference.
1 Metabolite NMR data.
2 Classification based on the electronic database The Human Metabolome Database (HMDB) Version 4.0.

Table S3. Alkaloids isolated from *Callyspongia* species.

Metabolite name<br>Chemical formula<br>Type of metabolite	Specie<br>Geographic Location	^{1}H and ^{13}C NMR data [Reference]
2-bromoaldisine (**105**)<br>$C_8H_7BrN_2O_2$<br>Bromopyrrole alkaloid	*Callyspongia* sp.<br>Australia	^{1}H NMR (600 MHz, CD_3OD, δ/ppm): 6.68 (s, H-3), 3.51 (m, H-8) and 2.82 (m, H-9). ^{13}C NMR (CD_3OD): 107.2 (C-2), 113.3 (C-3), 126.9 (C-4), 130.6 (C-5), 164.1 (C-6), 38.2 (C-8), 44.7 (C-9) and 196.2 (C-10). [65]
Callyspongisine A (**106**)<br>$C_{12}H_{14}BrN_5O_5S$<br>Bromopyrrole alkaloid	*Callyspongia* sp.<br>Australia	^{1}H NMR (600 MHz, DMSO-d_6, δ/ppm, *J*/Hz): 12.71 (s, 1-NH), 6.13 (s, H-3), 8.27 (t, 5.4, 7-NH), 3.30 (H-8), 2.47 (m, H-9a), 2.39 (m, H-9b), 10.16 (s, 14-NH_2a), 9.74 (s, 14-NH_2b), 10.34 (t, 5.4, 15-NH), 3.66 (ddd, 7.0, 7.0 and 5.4, H-16), 2.74 (dt, 13.0 and 7.0, H-17a) and 2.67 (dt, 13.0 and 7.0, H-17b). ^{13}C NMR (150 MHz, DMSO-d_6): 105.1 (C-2), 110.5 (C-3), 118.8 (C-4), 127.3 (C-5), 161.1 (C-6), 36.1 (C-8), 36.6 (C-9), 91.1 (C-10), 181.0 (C-11), 172.3 (C-13), 41.1 (C-16) and 48.8 (C-17). [65]
Callyspongisine B (**107**)<br>$C_{12}H_{12}BrN_4O_6S$<br>Bromopyrrole alkaloid	*Callyspongia* sp.<br>Australia	^{1}H NMR (600 MHz, DMSO-d_6, δ/ppm, *J*/Hz): 12.43 (s, 1-NH), 5.86 (s, H-3), 8.10 (dd, 6.9 and 2.8, 7-NH), 3.28 (H-8), 2.26 (dd, 14.8 and 10.0, H-9a), 2.12 (dd, 14.8 and 6.8, H-9b), 8.79 (t, 5.5, 15-NH), 3.50 (m, H-16), 2.68 (m, H-17a) and 2.59 (m, H-17b). ^{13}C NMR (150 MHz, DMSO-d_6): 104.5 (C-2), 110.1 (C-3), 122.1 (C-4), 126.5 (C-5), 161.4 (C-6), 36.3 (C-8), 37.3 (C-9), 82.5 (C-10), 181.8 (C-11), 165.2 (C-13), 39.6 (C-16) and 49.31 (C-17). [65]
Callyspongisine C (**108**)<br>$C_{13}H_{17}BrN_2O_4$<br>Bromopyrrole alkaloid	*Callyspongia* sp.<br>Australia	^{1}H NMR (600 MHz, DMSO-d_6, δ/ppm, *J*/Hz): 12.25 (s, 1-NH), 6.13 (s, H-3), 7.99 (t, 1.0, 7-NH), 3.28 (m, H-8a), 3.15 (ddd, 7.7, 7.7 and 1.0, H-8b), 2.31 (dd, 15.0 and 7.7, H-9a), 2.16 (dd, 15.0 and 7.7, H-9b), 4.15 (dq, 14.3 and 7.0, $CO_2CH_2CH_3$a), 4.11 (dq, 14.3 and 7.0, $CO_2CH_2CH_3$b), 3.45 (dq, 14.2 and 7.0, OCH_2CH_3a), 3.30 (OCH_2CH_3b), 1.17 (t, 7.0, $CO_2CH_2CH_3$) and 1.06 (t, 7.0, OCH_2CH_3). ^{13}C NMR (150 MHz, DMSO-d_6): 103.6 (C-2), 112.6 (C-3), 124.6 (C-4), 125.4 (C-5), 161.7 (C-6), 35.5 (C-8), 34.7 (C-9), 79.3 (C-10), 172.4 (C-11), 61.2 ($CO_2CH_2CH_3$), 60.0 (OCH_2CH_3), 14.2 ($CO_2CH_2CH_3$) and 15.8 (OCH_2CH_3). [65]
Callyspongisine D (**109**)<br>$C_{11}H_{13}BrN_2O_4$<br>Bromopyrrole alkaloid	*Callyspongia* sp.<br>Australia	^{1}H NMR (600 MHz, DMSO-d_6, δ/ppm, *J*/Hz): 12.29 (s, 1-NH), 6.13 (s, H-3), 8.00 (s, 7-NH), 3.26 (m, H-8a), 3.16 (m, H-8b), 2.32 (dd, 15.0 and 7.7, H-9a),

		2.16 (dd, 15.0 and 7.7, H-9b), 3.67 (s, CO_2CH_3) and 3.16 (s, OCH_3). ^{13}C NMR (150 MHz, DMSO-d_6): 103.6 (C-2), 112.4 (C-3), 124.1 (C-4), 125.7 (C-5), 161.6 (C-6), 35.5 (C-8), 34.4 (C-9), 79.7 (C-10), 172.7 (C-11), 52.6 (CO_2CH_3) and 52.2 (OCH_3). [65]
Hymenialdisine (**110**)<br>$C_{11}H_{10}BrN_5O_2$<br>Bromopyrrole alkaloid	*Callyspongia* sp.<br>Australia	^{1}H NMR (600 MHz, DMSO-d_6, δ/ppm, *J*/Hz): 12.83 (s, 1-NH), 6.63 (s, H-3), 8.09 (s, 7-NH), 3.26 (s, H-8) and 3.26 (s, H-9). ^{13}C NMR (150 MHz, DMSO-d_6): 104.9 (C-2), 111.2 (C-3), 121.6 (C-4), 128.3 (C-5), 162.3 (C-6), 38.9 (C-8), 31.8 (C-9), 128.3 (C-10), 121.6 (C-11), 163.7 (C-12) and 154.6 (C-14).[1] [65]<br>^{1}H NMR (600 MHz, DMSO-d_6, δ/ppm, *J*/Hz): 12.63 (s, 1-NH), 6.74 (s, H-3), 8.03 (s, 7-NH), 3.26 (s, H-8) and 2.83 (s, H-9).[2] [65]
Callyimine A (**111**)<br>$C_{14}H_{16}N_3^+$<br>Indolizinium derived alkaloid	*Callyspongia* sp.<br>China	^{1}H NMR (500 MHz, CD_3OD, δ/ppm, *J*/Hz): 4.63 (2H, t, 6.1, H-1), 2.20 (2H, m, H-2), 2.04 (2H, q, 6.7, H-3), 7.78 (1H, d, 8.2, H-5), 8.31 (1H, d, 8.2, H-6), 7.49 (2H, d, 8.4, H-2′, H-6′) and 6.89 (2H, d, 8.4, H-3′, H-5′); ^{13}C NMR (125 MHz, CD_3OD): 53.8 (C-1), 18.2 (C-2), 22.5 (C-3), 150.5 (C-4), 126.9 (C-5), 146.4 (C-6), 136.6 (C-7), 166.0 (C-8), 127.4 (C-1′), 131.3 (C-2′, C-6′), 116.8 (C-3′, C-5′) and 160.0 (C-4′). [18]
Callylactam A (**112**)<br>$C_{13}H_{13}NO_2$<br>Pyridinone derived alkaloid	*Callyspongia* sp.<br>China	^{1}H NMR (500 MHz, CD_3OD, δ/ppm, *J*/Hz): 3.44 (2H, t, 7.3, H-3), 2.43 (2H, q, 7.1, H-4), 6.62 (1H, t, 4.1, H-5), 3.98 (2H, s, H-7), 8.02 (2H, d, 7.3, H-2′, H-6′), 7.51 (2H, t, 7.8, H-3′, H-5′) and 7.62 (1H, t, 7.5, H-4′). ^{13}C NMR (125 MHz, CD_3OD): 168.4 (C-1), 40.4 (C-3), 25.2 (C-4), 141.3 (C-5), 130.8 (C-6), 40.7 (C-7), 199.9 (C-8), 138.2 (C-1′), 129.4 (C-2′, C-6′), 129.7 (C-3′, C-5′) and 134.4 (C-4′). [18]
Clathryimine B (**113**)<br>$C_{15}H_{16}N^+$<br>Quinolizinium derived alkaloid	*Callyspongia* sp.<br>China	^{1}H NMR (500 MHz, $CDCl_3$, δ/ppm, *J*/Hz): 5.19 (bs, H-1), 2.26 (p, 6.5, H-2), 2.09 (p, 6.5, H-3), 3.31 (p, 6.5, H-4), 7.79 (d, 8.5, H-6), 8.42 (dd, 8.5 and 0.8, H-7), 9.92 (bs, H-9), 7.92 (d, 7.5, H-11, H-15), 7.54 (d, 7.5, H-12, H-14) and 7.48 (d, 7.5, H-13). ^{13}C NMR (125 MHz, $CDCl_3$): 56.4 (C-1), 21.3 (C-2), 17.8 (C-3), 28.4 (C-4), 153.4 (C-5), 128.2 (C-6), 141.5 (C-7), 132.7 (C-8), 144.1 (C-9), 138.6 (C-10), 127.6 (C-11, C-15), 129.8 (C-12, C-14) and 130.3 (C-13). [18, 73[3]]
3-(2-(1*H*-indol-3-yl)-2-oxoethyl)-5,6-dihydropyridin-2(1*H*)-one (**114**)<br>$C_{15}H_{14}N_2O_2$<br>Pyridinone derived alkaloid	*Callyspongia* sp.<br>China	^{1}H NMR (100 MHz, $CD_3OD/CDCl_3$, δ/ppm, *J*/Hz): 2.44 (2H, br dq), 3.46 (2H, t, 7), 3.90 (2H, bs), 6.64 (1H, t, 4), 7.29-7.35 (2H, m), 7.40-7.54 (1H, m), 8.16 (1H, s) and 8.20-9.35 (1H, m). [18,74[3]]

3-(2-(4-hydroxyphenyl)-2-oxoethyl)-5,6-dihydropyridin-2(1*H*)-one (**115**)<br>$C_{13}H_{13}NO_3$<br>Pyridinone derived alkaloid	*Callyspongia* sp.<br>China	^{1}H NMR (100 MHz, $CD_3OD/CDCl_3$, δ/ppm, *J*/Hz): 7.92 (d, 9), 6.86 (d), 6.56 (t, 4), 3.92 (s), 3.46 (t, ~7.5) and 2.44 (m, ~4 and ~7.5). [18,74[3]]
(1*R*,3*R*)-1-methyl-2,3,4,9-tetrahydro-1*H*-pyrido[3,4-b]indole-3-carboxylic acid (**116a**)<br>$C_{13}H_{14}N_2O_2$<br>Tryptophan-derived alkaloids	*Callyspongia* sp.<br>China	Spectroscopic data not provided. [66]
(1*R*,3*S*)-1-methyl-2,3,4,9-tetrahydro-1*H*-pyrido[3,4-b]indole-3-carboxylic acid (**116b**)<br>$C_{13}H_{14}N_2O_2$<br>Tryptophan-derived alkaloids	*Callyspongia* sp.<br>China	Spectroscopic data not provided. [66]
C^2-*α*-*D*-mannosylpyranosyl-tryptophan (**117**)<br>$C_{17}H_{22}N_2O_7$<br>Tryptophan-derived alkaloids	*Callyspongia* sp.<br>China	Spectroscopic data not provided. [66]
Ethyl 2-(1*H*-indol-3-yl) acetate (**118**)<br>$C_{12}H_{13}NO_2$<br>Indole alkaloid	*Callyspongia* sp.<br>China	Spectroscopic data not provided. [67]
1*H*-indole-3-carbaldehyde (**119**)<br>C_9H_7NO<br>Indole alkaloid	*Callyspongia* sp.<br>China	Spectroscopic data not provided. [67]
5-bromo trisindoline (**120**)<br>$C_{24}H_{16}BrN_3O$<br>Brominated oxindole alkaloid	*Callyspongia siphonella*<br>Egypt	^{1}H NMR (400 MHz, DMSO-d_6, δ/ppm, *J*/Hz): 10.77 (1H, br s, 1-NH), 7.30 (1H, s, H-4), 7.42 (1H, d, 8, H-6), 6.97 (1H, d, 8, H-7), 11.03 (2H, br s, 1-NH′, 1-NH′′), 6.89 (2H, s, H-2′, H-2′′), 7.22 (2H, d, 8, H-4′, H-4′′), 6.83 (2H, t, 8, H-5′, H-5′′), 7.04 (2H, t, 8, H-6′, H-6′′) and 7.39 (2H, d, 8, H-7′, H-7′′). ^{13}C NMR (100 MHz, DMSO-d_6): 178.9 (C-2), 53.2 (C-3), 137.4 (C-3a), 127.8 (C-4), 114 (C-5), 131.2 (C-6), 112.2 (C-7), 141.1 (C-7a), 124.9 (C-2′, C-2′′), 113.6 (C-3′, C-3′′), 126 (C-3′a, C-3′′a), 121 (C-4′, C-4′′), 119 (C-5′, C-5′′), 121.5 (C-6′, C-6′′), 112.2 (C-7′, C-7′′) and 137.4 (C-7′a, C-7′′a). [7]
6-bromo trisindoline (**121**)<br>$C_{24}H_{16}BrN_3O$<br>Brominated oxindole alkaloid	*Callyspongia siphonella*<br>Egypt	^{1}H NMR (400 MHz, DMSO-d_6, δ/ppm, *J*/Hz): 10.75 (1H, br s, 1-NH), 7.16 (1H, d, 8, H-4), 7.12 (1H, dd, 2 and 8, H-5), 7.14 (1H, s, H-7), 11.0 (1H, br s, 1-NH′, 1-NH′′), 6.85 (2H, d, 2.5, H-2′, H-2′′), 7.2 (2H, d, 8, H-4′, H-4′′), 6.81 (2H, t, 8, H-5′, H-5′′), 7.03 (2H, t, 8, H-6′, H-6′′) and 7.35 (2H, d, 8, H-7′, H-7′′). ^{13}C NMR (100 MHz, DMSO-d_6): 179 (C-2), 52.7 (C-3), 134.3 (C-3a), 127.1 (C-4), 124.6 (C-5), 120.7 (C-6), 112.8 (C-7), 143.5 (C-7a), 124.8 (C-2′, C-2′′), 114.0 (C-3′, C-3′′), 126 (C-3′a, C-3′′a), 121 (C-4′, C-4′′), 118.8 (C-5′, C-5′′), 121.5 (C-6′, C-6′′), 112.1 (C-7′, C-7′′) and 137.4 (C-7′a, C-7′′a). [7]

Untenine A (**122**)<br>$C_{19}H_{30}N_2O_2$<br>Nitroalkyl pyridine alkaloid	*Callyspongia* sp.<br>Japan	^{1}H NMR (400 MHz, CD_3OD, δ/ppm, *J*/Hz): 8.35 (1H, bs, H-2), 7.68 (1H, d, 8.0, H-4), 7.33 (1H, dd, 8.0 and 4.8, H-5), 8.33 (1H, d, 4.8, H-6), 2.71 (2H, t, 7.3, H-7), 2.38 (2H, m, H-8), 5.37 (2H, m, H-9, H-10), 1.90 (2H, m, H-11), 1.15-1.38 (14H, m, H-12-18), 1.96 (2H, m, H-19), 4.42 (2H, t, 7.1, H-20). ^{13}C NMR (CD_3OD): 151.0 (d, C-2), 140.2 (s, C-3), 139.2 (d, C-4), 125.8 (d, C-5), 148.3 (d, C-6), 34.6 (t, C-7), 30.5 (t, C-8), 129.6 (d, C-9), 133.2 (d, C- 10), 28.9 (t, C-11), 31.4 (t, C-12), 31.3, 31.2, 30.8, 30.7 and 28.1 (t for each, C-13-18), 29.2 (t, C-19) and 77.4 (t, C-20). [68]
Untenine B (**123**)<br>$C_{17}H_{28}N_2O_2$<br>Nitroalkyl pyridine alkaloid	*Callyspongia* sp.<br>Japan	^{1}H NMR (400 MHz, CD_3OD, δ/ppm, *J*/Hz): 8.35 (1H, bs, H-2), 7.68 (1H, d, 8.0, H-4), 7.33 (1H, dd, 4.8 and 8.0, H-5), 8.32 (1H, d, 4.8, H-6), 2.65 (2H, t, 7.2, H-7), 1.63 (2H, m, H-8), 1.20-1.40 (16H, m, H-9-16), 1.96 (2H, m, H-17), 4.42 (2H, t, 7.1, H-18). ^{13}C NMR (CD_3OD): 151.1 (d, C-2), 140.5 (s, C-3), 139.1 (d, C-4), 125.6 (d, C-5), 148.4 (d, C-6), 34.1 (t, C-7), 30.0-31.5 and 28.1 (t, C-8-16), 29.1 (t, C-17) and 77.4 (t, C-18). [68]
Untenine C (**124**)<br>$C_{19}H_{28}N_2O_2$<br>Nitroalkyl pyridine alkaloid	*Callyspongia* sp.<br>Japan	^{1}H NMR (400 MHz, CD_3OD, δ/ppm, *J*/Hz): 8.42 (1H, bs, H-2), 7.74 (1H, d, 8.0, H-4), 7.34 (1H, dd, 8.0 and 4.8, H-5), 8.37 (1H, d, 4.8, H-6), 2.80 (2H, t, 7.3, H-7), 2.46 (2H, m, H-8), 2.07 (2H, m, H-11), 1.20-1.45 (14H, m, H-12-18), 1.96 (2H, m, H-19), 4.44 (2H, t, 7.1, H-20). ^{13}C NMR (CD_3OD): 151.2 (d, C-2), 140.6 (s, C-3), 139.1 (d, C-4), 125.6 (d, C-5), 148.6 (d, C-6), 34.2 (t, C-7), 22.0 (t, C-8), 80.4 (s, C-9), 83.5 (s, C-10), 20.0 (t, C-11), 30.5-31.5 and 28.1 (t, C-13-18), 29.1 (t, C-19) and 77.4 (t, C-20). [68]
Niphatoxin C (**125**)<br>$C_{36}H_{50}N_3^+$<br>Tripyridine Alkaloid	*Callyspongia* sp.<br>Australia	^{1}H NMR (600 MHz, DMSO-d_6, δ/ppm, *J*/Hz): 8.97 (s, H-2), 8.41 (d, 8.4, H-4), 8.08 (dd, 8.4 and 6.0, H-5), 8.94 (d, 6.0, H-6), 3.59 (2H, d, 7.2, H-7), 5.56 (br dt, 10.8 and 7.2, H-8), 5.61 (br dt, 10.8 and 7.2, H-9), 2.12 (2H, q, 7.0, H-10), 1.34 (2H, m, H-11), 1.28 (2H, m, H-12, H-13), 1.58 (2H, m, H-14), 2.67 (2H, q, 7.0, H-15), 8.60 (br s, H-17), 8.57 (br d, 6.0, H-19), 7.62 (br dd, 8.4 and 6.0, H-20), 8.00 (br d, 8.4, H-21), 4.57 (2H, t, 7.5, H-1′), 1.90 (2H, quint., 7.5, H-2′), 1.29 (2H, m, H-3′), 2.01 (2H, q, 7.5, H-4′), 5.30 (br dt, 10.8 and 7.2, H-5′), 5.35 (br dt, 10.8 and 7.2, H-6′), 1.96 (2H, q, 7.5, H-7′), 1.28 (2H, m, H-8′, H-9′, H-10′), 1.58 (2H, m, H-11′), 2.67 (2H, q, 7.0, H-12′), 8.60 (br s, H-14′), 8.57 (br d, 6.0, H-16′), 7.62 (dd, 8.4 and 6.0,

		H-17′) and 8.00 (br d, 8.4, H-18′). ^{13}C NMR (125 MHz, DMSO-d_6): 143.7 (C-2), 141.8 (C-3), 144.9 (C-4), 127.7 (C-5), 142.4 (C-6), 29.5 (C-7), 125.0 (C-8), 133.0 (C-9), 26.7 (C-10), 28.7 (C-11), 28.3 (C-12, C-13), 30.2 (C-14), 31.8 (C-15), 139.5 (C-16), 145.8 (C-17), 143.7 (C-19), 124.8 (C-20), 140.1 (C-21), 60.6 (C-1′), 30.3 (C-2′), 25.5 (C-3′), 26.0 (C-4′), 128.7 (C-5′), 130.2 (C-6′), 26.5 (C-7′), 28.9 (C-8′), 28.3 (C-9′, C-10′), 30.2 (C-11′), 31.8 (C-12′), 139.5 (C-13′), 145.8 (C-14′), 143.7 (C-16′), 124.8 (C-17′) and 140.1 (C-18′). [69]
Callysponine (**126**)<br>$C_9H_{14}N_2O_2S$<br>Diazepine	*Callyspongia* sp.<br>China	^{1}H NMR (500 MHz, $CDCl_3$, δ/ppm, *J*/Hz): 3.51 (m, H-3), 3.61 (m, H-3), 2.00 (m, H-4), 2.06 (m, H-4), 2.34 (m, H-5), 4.11 (t, 7.5, H-6), 6.73 (s, H-8), 4.36 (dt, 6.5 and 3.0, H-9), 3.97 (d, 3.0, H-10), 1.34 (d, 6.5, H-11) and 1.89 (m, H-12). ^{13}C NMR (125 MHz, $CDCl_3$): 165.4 (C-1), 45.3 (C-3), 22.6 (C-4), 28.1 (C-5), 59.0 (C-6), 170.2 (C-7), 65.6 (C-9), 59.4 (C-10) and 18.9 (C-11). [70]
Cyclo-(*S*-Pro-*R*-Tyr) (**127**)<br>$C_{14}H_{16}N_2O_3$<br>Diketopiperazine	*Callyspongia* sp.<br>China	^{1}H NMR (500 MHz, $CDCl_3$, δ/ppm, *J*/Hz): 7.03 (2H, d, 8.3, H-2′, H-6′), 6.77 (2H, d, 8.3, H-3′, H-5′), 6.11 (1H, s, NH), 4.22 (1H, dd, 2.9 and 9.6, H-9), 4.07 (1H, t, 7.6, H-6), 3.57 (2H, m, H-3), 3.42 (1H, dd, 11.0 and 14.0, H-10), 2.78 (1H, dd, 9.6 and 14.4, H-10), 2.32 (1H, m, H-5a), 1.96 (1H, m, H-5b) and 1.85 (2H, m, H-4). ^{13}C NMR (125 MHz, $CDCl_3$): 169.95 (s, C-7), 165.48 (s, C-1), 156.05 (s, C-4′), 130.58 (d, C-2′, C-6′), 126.93 (s, C-1′), 116.32 (d, C-3′, C-5′), 59.36 (d, C-6), 56.51 (d, C-9), 45.65 (t, C-3), 36.17 (t, C-10), 28.54 (t, C-5) and 22.64 (t, C-4). [70,75[3]]
Cyclo-(*S*-Pro-*R*-Val) (**128**)<br>$C_{10}H_{16}N_2O_2$<br>Diketopiperazine	*Callyspongia* sp.<br>China	^{1}H NMR (300 MHz, $CDCl_3$, δ/ppm, *J*/Hz): 3.55 (1H, dt, 9.1 and 2.8, H-3), 3.63 (1H, m, H-3), 2.02-1.99 (1H, m, H-4), 1.93-1.88 (1H, m, H-4), 2.4-2.3 (1H, m, H-5), 2.1-2.06 (1H, m, H-5), 4.08 (1H, dt, 7.8 and 1.8, H-6), 5.72 (1H, dd, 1.5 and 1.2, N-H), 3.94 (1H, br s, H-9), 2.64 (1H, m, H-10), 0.91 (3H, d, 7.2, H-11) and 1.06 (3H, d, 7.2, Me-10). ^{13}C NMR (75 MHz, $CDCl_3$): 45.2 (C-3), 22.5 (C-4), 28.6 (C-5), 58.9 (C-6), 60.4 (C-9), 28.4 (C-10), 19.4 (C-11) and 16.1 (Me-10). [70,76[3]]
Cyclo-(*S*-Pro-*R*-Ala) (**129**)<br>$C_8H_{12}N_2O_2$<br>Diketopiperazine	*Callyspongia* sp.<br>China	^{1}H NMR (400 MHz, δ/ppm, *J*/Hz): 1.48 (d, 6.8, CH_3), 1.89-1.96 (1H, m, CH_2), 1.98-2.07 (1H, m, CH_2), 2.09-2.15 (1H, m, CH_2), 2.32-2.37 (1H, m, CH_2), 3.52-3.65 (2H, m, NCH_2), 4.09-4.16 (2H, m, CH) and 6.94 (br, NH). ^{13}C NMR (100 MHz): 15.83 (CH_3), 22.70 (CH_2),

		28.10 (CH_2), 45.38 (CH_2N), 51.12 (CH), 59.21 (CH), 166.36 (CO) and 170.53 (CO). [70,77[3]]
	Callyspongia sp. China	Spectroscopic data not provided. [66]
Cyclo-(*S*-Pro-*R*-Leu) (Cyclo-((*S*)-Pro-(*R*)-Leu)) (**130**) $C_{11}H_{18}N_2O_2$ Diketopiperazine	*Callyspongia* sp. China	^{1}H NMR (300 MHz, $CDCl_3$, δ/ppm, *J*/Hz): 3.6-3.5 (2H, m, H-3), 1.94-1.86 (1H, m, H-4), 2.02-1.99 (1H, m, H-4), 2.13 (1H, m, H-5), 2.33 (1H, m, H-5), 4.12 (1H, t, 8.1, H-6), 5.91 (1H, br s, N-H), 4.01 (1H, dd, 9.4 and 3.4, H-9), 2.01 (1H, m, H-10), 1.52 (1H, ddd, 14.5, 9.6 and 4.9, H-10), 1.76-1.69 (1H, m, H-11), 0.94 (3H, d, 6.3, H-12) and 1.00 (3H, d, 6.3, Me-11). ^{13}C NMR (75 MHz, $CDCl_3$): 171.4 (C-1), 45.6 (C-3), 22.8 (C-4), 28.2 (C-5), 59.1 (C-6), 167.1 (C-7), 53.4 (C-9), 38.7 (C-10), 24.8 (C-11), 22.8 (C-12) and 21.2 (Me-11). [66,70,76[3]]
	Callyspongia sp. China	^{1}H NMR (500 MHz, CD_3OD, δ/ppm, *J*/Hz): 3.54-3.51 (m, H-3), 1.94-1.87 (m, H-4), 2.06-1.99 (m, H-4), 2.35-2.31 (m, H-5), 1.96-1.87 (m, H-5), 4.15 (br s, H-6), 4.29 (t, 7.1, H-9), 1.99-1.94 (m, H-10), 1.57-1.53 (m, H-10), 2.06-2.01 (m, H-11), 1.01 (d, 6.5, Me-12) and 0.97 (d, 6.5, Me-13). ^{13}C NMR (125 MHz, CD_3OD): 172.9 (C-1), 46.5 (C-3), 23.6 (C-4), 29.1 (C-5), 60.3 (C-6), 168.9 (C-7), 54.7 (C-9), 39.4 (C-10), 25.7 (C-11), 23.4 (Me-12) and 22.2 (Me-13). [71]
Callysponine A (**131**) $C_{14}H_{14}N_2O_2S$ Diketopiperazine	*Callyspongia* sp. China	^{1}H NMR (500 MHz, $CDCl_3$, δ/ppm, *J*/Hz) 7.31 (2H, d, 7.5, H-2′, H-6′), 7.26 (2H, dd, 7.5 and 7.1, H-3′, H-5′), 7.22 (1H, d, 7.1, H-4′), 4.73 (1H, dd, 9.5 and 4.5, H-9), 4.15 (1H, dd, 9.0 and 4.0, H-6), 3.29 (1H, dd, 11.0 and 9.5, H_β-10), 3.02 (1H, dd, 11.0 and 4.5, H_α-10), 2.40 (1H, m, H_β-4), 2.24 (2H, m, H-5) and 1.95 (1H, m, H_α-4). ^{13}C NMR (125 MHz, $CDCl_3$) 181.6 (C-3), 174.9 (C-1), 174.6 (C-7), 138.5 (C-1′), 130.4 (C-3′, C-5′), 129.6 (C-2′, C-6′), 124.4 (C-4′), 58.1 (C-6), 55.1 (C-9), 38.2 (C-10), 30.1 (C-4) and 26.7 (C-5). [72]
Cyclo-(Gly-Pro) (**132**) $C_7H_{10}N_2O_2$ Diketopiperazine	*Callyspongia* sp. China	Spectroscopic data not provided. [72]
Cyclo-(Ile-Pro) (**133**) $C_{11}H_{18}N_2O_2$ Diketopiperazine	*Callyspongia* sp. China	^{1}H NMR (300 MHz, $CDCl_3$, δ/ppm, *J*/Hz): 3.6-3.5 (2H, m, H-3), 2.0-1.9 (1H, m, H-4), 1.9-1.8 (1H, m, H-4), 2.3-2.2 (1H, m, H-5), 2.1-2.0 (1H, m, H-5), 4.07 (1H, t, 7.5, H-6), 5.99 (1H, br s, N-H), 3.96 (1H, br s, H-9), 2.4-2.3 (1H, m, H-10), 1.5-1.4 (1H, m, H-11), 1.3-1.1 (1H, m, H-11), 0.92 (3H, t, 7.4, H-12) and 1.05 (3H, d, 7.2, Me-10). ^{13}C NMR (75 MHz, $CDCl_3$):

		169.9 (C-1), 45.2 (C-3), 22.4 (C-4), 28.6 (C-5), 58.9 (C-6), 165.1 (C-7), 60.6 (C-9), 35.3 (C-10), 24.1 (C-11), 12.2 (C-12) and 16.0 (Me-10). [72,76[3]]
Cyclo-(Pro-Pro) (**134**)<br>$C_{10}H_{14}N_2O_2$<br>Diketopiperazine	*Callyspongia* sp.<br>China	^{1}H NMR (400 MHz, DMSO-d_6, δ/ppm, *J*/Hz): 1.70-1.90 (6H, m, H-7a, H-10a, H-8, H-11), 2.13 (2H, m, H-7b, H-10b), 3.36 (4H, m, H-9, H-12) and 4.27 (2H, t, 8.0, H-3, H-6). ^{13}C NMR (100 MHz, DMSO-d_6): 23.0 (C-8, C-11), 27.3 (C-7, C-10), 44.7 (C-9, C-12), 59.9 (C-3, C-6) and 166.1 (C-2, C-5). [72,78[3]]
Cyclo-(Thr-Pro) (**135**)<br>$C_9H_{14}N_2O_3$<br>Diketopiperazine	*Callyspongia* sp.<br>China	Spectroscopic data not provided. [72]
Cyclo-(*R*-Pro-6-hydroxyl-*R*-Ile) (**136**)<br>$C_{11}H_{18}N_2O_3$<br>Diketopiperazine	*Callyspongia* sp.<br>China	^{1}H NMR (500 MHz, CD_3OD, δ/ppm, *J*/Hz): 3.64-3.61 (2H, m, H-3), 3.60 (1H, d, 7.5, H-9), 2.29- 2.26 (1H, m, H-5a), 2.18-2.16 (1H, m, H-4a), 2.13-2.09 (1H, m, H-5b), 2.09-2.05 (1H, m, H-10), 2.01- 1.97 (1H, m, H-4b), 1.78-1.67 (1H, m, H-11a), 1.28-1.19 (1H, m, H-11b), 1.03 (3H, d, 7.0, H- 13) and 0.98 (3H, t, 7.5, H-12). ^{13}C NMR (125 MHz, CD_3OD): 170.5 (C-1), 169.5 (C-7), 88.1 (C-6), 63.6 (C-9), 46.6 (C-3), 41.2 (C-10), 38.1 (C-5), 26.6 (C-11), 20.3 (C-4), 15.9 (C-13) and 11.4 (C-12). [66]
Cyclo-(*R*-Pro-*R*-Phe) (**137**)<br>$C_{14}H_{16}N_2O_2$<br>Diketopiperazine	*Callyspongia* sp.<br>China	^{1}H NMR (300 MHz, $CDCl_3$, δ/ppm, *J*/Hz): 3.7-3.6 (1H, m, H-3), 3.6-3.5 (1H, m, H-3), 1.9-1.8 (2H, m, H-4), 2.4-2.3 (1H, m, H-5), 2.1-2.0 (1H, m, H-5), 4.08 (1H, t, 7.1, H-6), 5.60 (1H, br s, N-H), 4.27 (1H, dd, 10.6 and 2.6, H-9), 3.6-3.5 (1H, m, H-10), 2.77 (1H, dd, 14.4 and 10.8, H-10), and 7.4-7.2 (5H, Ar-10). ^{13}C NMR (75 MHz, $CDCl_3$): 45.6 (C-3), 22.6 (C-4), 28.4 (C-5), 59.2 (C-6), 56.2 (C-9), 36.9 (C-10), 129.4 (C-1′), 129.2 (C-2′), 127.6 (C-3′) and 129.2 (C-4′). [66,76[3]]
Cyclo-(*R*-Tyr-*R*-Phe) (**138**)<br>$C_{15}H_{20}N_2O_3$<br>Diketopiperazine	*Callyspongia* sp.<br>China	Spectroscopic data not provided. [66]
Cyclo-(*S*-Pro-*S*-Phe) (**139**)<br>$C_{14}H_{16}N_2O_2$<br>Diketopiperazine	*Callyspongia* sp.<br>China	^{1}H NMR (300 MHz, $CDCl_3$, δ/ppm): 3.69-3.61 (1H, m, H-3), 3.61-3.50 (1H, m, H-3), 1.92-1.81 (2H, m, H-4), 2.38-2.28 (1H, m, H-5), 2.10-2.00 (1H, m, H-5), 4.08 (1H, t, H-6), 5.62 (1H, br s, N-H), 4.27 (1H, dd, H-9), 3.59-3.45 (1H, m, H-10), 2.77 (1H, dd, H-10), and 7.41-7.18 (5H, Ar). ^{13}C NMR (75 MHz, $CDCl_3$): 169.4 (C-1), 45.2 (C-3), 22.2 (C-4), 28.3 (C-5), 58.9 (C-6), 164.9 (C-7), 56.1 (C-9), 36.6 (C-10), 135.8 (C-1′), 128.9 (C-2′), 127.3 (C-3′) and 129.1 (C-4′). [66,79[3]]
Staphyloamide A (**140**)<br>$C_{11}H_{18}N_2O_3$	*Callyspongia* sp.<br>China	Spectroscopic data not provided. [66]

Diketopiperazine		
Dysamide A (**141**) $C_{14}H_{20}Cl_6N_2O_2$ Diketopiperazine	*Callyspongia* sp. Indonesia	^{1}H NMR (400 MHz, $CDCl_3$, δ/ppm, *J*/Hz): 3.97 (2H, t, 7.1), 3.03 (6H, s), 2.98 (2H, m), 2.50 (2H, ddd, 14.6, 6.3 and 2.4), 1.80 (2H, ddd, 14.6, 7.3 and 7.3) and 1.39 (6H, d, 6.8). ^{13}C NMR (100 MHz, $CDCl_3$) 166.7, 105.4, 61.7, 52.1, 39.0, 33.6 and 17.9. [6]
Callyspongidipeptide A (Cyclo-((*S*)-Pro-8-hydroxy-(*R*)-Ile) (**142**) $C_{11}H_{18}N_2O_3$ Diketopiperazine	*Callyspongia* sp. China	^{1}H NMR (500 MHz, CD_3OD, δ/ppm, *J*/Hz): 3.60-3.53 (m, H-3), 3.52-3.49 (m, H-3), 2.06-2.02 (m, H-4), 1.98-1.91 (m, H-4), 2.35-2.32 (m, H-5), 2.00-1.92 (m, H-5), 4.10 (br s, H-6), 4.22 (t, 7.0, H-9), 2.20-2.16 (m, H-10), 1.49-1.43 (m, H-11), 1.37-1.31 (m, H-11), 1.07 (t, 6.9, Me-12) and 0.95 (d, 6.9, Me-13). ^{13}C NMR (125 MHz, CD_3OD): 172.6 (C-1), 46.2 (C-3), 23.3 (C-4), 29.6 (C-5), 61.3 (C-6), 167.6 (C-7), 60.0 (C-9), 25.5 (C-10), 37.1 (C-11), 15.6 (Me-12) and 12.6 (Me-13). [71]
Cyclo-((*S*)-Pro-(*R*)-Ile) (**143**) $C_{11}H_{18}N_2O_2$ Diketopiperazine	*Callyspongia* sp. China	^{1}H NMR (300 MHz, $CDCl_3$, δ/ppm, *J*/Hz): 3.6-3.5 (2H, m, H-3), 2.0-1.9 (1H, m, H-4), 1.9-1.8 (1H, m, H-4), 2.3-2.2 (1H, m, H-5), 2.1-2.0 (1H, m, H-5), 4.07 (1H, t, 7.5, H-6), 5.99 (1H, br s, N-H), 3.96 (1H, br s, H-9), 2.4-2.3 (1H, m, H-10), 1.5-1.4 (1H, m, H-11), 1.3-1.1 (1H, m, H-11), 0.92 (3H, t, 7.4, H-12) and 1.05 (3H, d, 7.2, Me-10). ^{13}C NMR (75 MHz $CDCl_3$): 169.9 (C-1), 45.2 (C-3), 22.4 (C-4), 28.6 (C-5), 58.9 (C-6), 165.1 (C-7), 60.6 (C-9), 35.3 (C-10), 24.1 (C-11), 12.2 (C-12) and 16.0 (Me-10). [71,76[3]]
Seco-((*S*)-Pro-(*R*)-Val) (**144**) $C_{10}H_{19}N_3O_2$ Pyrrolidine derived	*Callyspongia* sp. China	^{1}H NMR (500 MHz, CD_3OD, δ/ppm, *J*/Hz): 3.59-3.55 (m, H-3), 3.54-3.50 (m, H-3), 2.05-2.01 (m, H-4), 1.98-1.94 (m, H-4), 2.35-2.33 (m, H-5), 1.98-1.94 (m, H-5), 4.05 (br s, H-6), 4.23 (t, 7.0, H-9), 2.50 (dq, 7.0 and 2.5, H-10), 1.11 (d, 7.3, H-11) and 0.95 (d, 6.9, Me-12). ^{13}C NMR (125 MHz, CD_3OD): 172.6 (C-1), 46.2 (C-3), 23.3 (C-4), 29.6 (C-5), 61.6 (C-6), 167.6 (C-7), 60.1 (C-9), 30.0 (C-10), 18.9 (C-11) and 16.7 (Me-12). [71]
(3*R*)-methylazacyclodecane (**145**) $C_{10}H_{21}N$ Nitrogenous macrocycle	*Callyspongia* sp. Federal States of Micronesia	^{1}H NMR (400 MHz, CD_3OD, δ/ppm, *J*/Hz): 3.25 (dt, 11.8 and 6.7, 1H, H-10), 3.19 (m, 1H, H-10), 3.11 (dd, 13.9 and 3.8, 1H, H-2), 3.05 (dd, 13.9 and 7.8, 1H, H-2), 2.13 (m, 1H, H-3), 1.89 (m, 2H, H_2-9), 1.68 (m, 2H, H_2-8), 1.63 (m, 2H, H_2-5), 1.60 (m, 2H, H_2-6), 1.59 (m, 2H, H_2-7), 1.58 (m, 2H, H_2-4) and 1.04 (d, 6.7, 3H, H_3-11). ^{13}C NMR (100 MHz, CD_3OD): 50.6 (C-2), 46.4 (C-10), 31.2 (C-4), 30.4 (C-3), 25.6 (C-7), 25.5 (C-6), 24.2 (C-8), 23.9 (C-5), 23.6 (C-9) and 19.9 (C-11). [5]

Callyazepin (**146**)<br>$C_{22}H_{40}ClNO_3$<br>Nitrogenous macrocycle	*Callyspongia* sp.<br>Federal States of Micronesia	^{1}H NMR (600 MHz, CD_3OD, δ/ppm, *J*/Hz): 3.55 (dd, 13.7 and 12.8, H-2α), 2.88 (dd, 13.7 and 4.8, H-2β), 2.19 (m, H-3), 1.16 (m, H-4), 1.44 (m, H-5), 1.91 (ddt, 13.7, 9.9 and 5.0, H-6α), 1.65 (ddt, 13.7, 9.0 and 3.7, H-6β), 3.43 (ddd, 13.7, 9.0 and 4.2, H-7α), 3.41 (dt, 13.7 and 4.8, H-7β), 1.55 (m, H-8), 1.46 (m, H-9), 1.60-1.35 (m, H-10-16), 1.67 (m, H-17), 1.18 (m, H-17), 0.92 (d, 6.9, H-18), 8.08 (s, H-19), 4.09 (q, 7.3, H-20), 3.83 (s, H-22) and 1.52 (d, 7.3, H-23). ^{13}C NMR (150 MHz, CD_3OD): 52.3 (C-2), 30.4 (C-3), 29.1 (C-4), 38.4 (C-5), 25.7 (C-6), 51.2 (C-7), 32.0 (C-8), 25.9 (C-9), 28.5-23.7 (C-10-16), 31.4 (C-17), 19.6 (C-18), 166.9 (C-19), 49.8 (C-20), 171.5 (C-21), 53.7 (C-22) and 16.2 (C-23). [5]<br>^{1}H NMR (600 MHz, CD_3OD, δ/ppm, *J*/Hz): 3.14 (dd, 14.2 and 11.8, H-2α), 3.19 (dd, 14.2 and 4.8, H-2β), 2.15 (m, H-3), 1.16 (m, H-4), 1.44 (m, H-5), 1.79 (m, H-6α), 1.72 (m, H-6β), 3.85 (ddd, 14.3, 9.6 and 4.8, H-7α), 3.06 (dt, 14.3 and 4.8, H-7β), 1.55 (m, H-8), 1.46 (m, H-9), 1.60-1.35 (m, H-10-16), 1.67 (m, H-17), 1.18 (m, H-17), 0.91 (d, 6.9, H-18), 8.08 (s, H-19), 4.09 (q, 7.3, H-20), 3.83 (s, H-22) and 1.52 (d, 7.3, H-23). ^{13}C NMR (150 MHz, CD_3OD): 57.9 (C-2), 31.0 (C-3), 29.1 (C-4), 38.4 (C-5), 25.4 (C-6), 45.6 (C-7), 32.0 (C-8), 25.9 (C-9), 28.5-23.7 (C-10-16), 31.4 (C-17), 19.9 (C-18), 166.7 (C-19), 49.8 (C-20), 171.5 (C-21), 53.7 (C-22) and 16.2 (C-23). [5]

[1] 10*Z*-hymenialdisine (Cited only in supplementary material of the article).
[2] 10*E*-hymenialdisine (Cited only in supplementary material of the article).
[3] Metabolite NMR data.

Table S4. Simple phenols and phenylpropanoids isolated from *Callyspongia* species.

**Metabolite name**<br>**Chemical formula**<br>**Type of metabolite**	**Specie**<br>**Geographic Location**	**^{1}H and ^{13}C NMR data [Reference]**
2-phenylacetamide (**147**)<br>C_8H_9NO<br>Phenylacetamide[1]	*Callyspongia* sp.<br>China	Spectroscopic data not provided. [67]
ρ-methoxyphenylacetic acid (**148**)<br>$C_9H_{10}O_3$<br>Phenol ether[1]	*Callyspongia* sp.<br>China	^{1}H NMR (500 MHz, $CDCl_3$, δ/ppm, *J*/Hz): 7.13 (2H, d, 8.5, H-4, H-8), 6.77 (2H, d, 8.5, H-5, H-7), 3.69 (3H, s, H-9) and 3.54 (2H, s, H-2). [67]
4-hydroxybenzoic acid (**149**)<br>$C_7H_6O_3$<br>Benzoic Acid Derivative[1]	*Callyspongia fibrosa*<br>China	^{1}H NMR (500 MHz, CD_3OD, δ/ppm, *J*/Hz): 7.86 (2H, 7.0, H-3, H-7) and 6.80 (2H, 7.0, H-4, H-6). [45]
4-hydroxyphenylacetic acid (**150**)<br>$C_8H_8O_3$<br>Phenol[1]	*Callyspongia* sp.<br>China	Spectroscopic data not provided. [67]
(*E*)-4-(4-hydroxyphenyl)-3-buten-2-one (**151**)<br>$C_{10}H_{10}O_2$<br>Cinnamic acid derivative[1]	*Callyspongia* sp.<br>China	^{1}H NMR (500 MHz, $CDCl_3$, δ/ppm, *J*/Hz): 7.44 (2H, d, 8.5), 6.88 (2H, d, 8.5), 7.48 (1H, d, 16), 6.60 (2H, d, 16) and 2.38 (3H, s). ^{13}C NMR (125 MHz, $CDCl_3$): 27.3 (C-1), 199.5 (C-2), 124.6 (C-3), 144.2 (C-4), 126.8 (C-5), 130.4 (C-6, C-10), 116.2 (C-7, C-9) and 158.7 (C-8). [67]
Phenylalanine (**152**)<br>$C_9H_{11}NO_2$<br>Amino Acid[1]	*Callyspongia* sp.<br>China	Spectroscopic data not provided. [67]
3,5-dibromo-4-methoxyphenylacetic acid (**153**)<br>$C_9H_8Br_2O_3$<br>Bromotyrosine derivative	*Callyspongia* sp.<br>Australia	^{1}H NMR (60 MHz, acetone-d_6, δ/ppm): 8.85 (COOH, br s), 7.47 (2H, s), 3.77 (3H, s) and 3.58 (2H, s). ^{13}C NMR (acetone-d_6): 171, 152, 133, 116, 59 and 38. [80,81[2]]
3,5-dibromo-4-methoxyphenylpyruvic acid (**154**)<br>$C_{10}H_8Br_2O_4$<br>Bromotyrosine derivative	*Callyspongia* sp.<br>Australia	^{1}H NMR (600 MHz, DMSO-d_6, δ/ppm, *J*/Hz): 8.03 (s, H-2 and H-6), 6.33 (s, H-7) and 3.77 (s, OCH_3). ^{13}C NMR (150 MHz, $CDCl_3$): 133.4 (C-1), 133.6 (C-2 and C-6), 117.7 (C-3 and C-5), 152.4 (C-4), 106.5 (C-7), 143.8 (C-8), 166.3 (C-9) and 61.0 (OCH_3). [80]
Callyspongic acid (**155**)<br>$C_{19}H_{17}Br_3N_2O_6$<br>Bromotyrosine derivative	*Callyspongia* sp.<br>Australia	^{1}H NMR (600 MHz, DMSO-d_6, δ/ppm, *J*/Hz): 7.23 (d, 2.1, H-2′), 6.79 (d, 8.3, H-5′), 6.92 (dd, 8.3 and 2.1, H-6′), 3.00 (dd, 14.0 and 5.0, H-7′), 2.95 (dd, 14.0 and 8.2, H-7′), 4.44 (m, H-8′), 7.40 (s, H-2 and H-6), 3.73 (d, 14.2, H-7), 3.70 (d, 14.2, H-7), 3.74 (s, OCH_3), 9.98 (s, OH), 7.87 (d, 7.9, NH) and 12.11 (s, N-OH). ^{13}C NMR (150 MHz, $CDCl_3$): 130.1 (C-1′), 133.7 (C-2′), 109.4 (C-3′), 153.3 (C-4′), 116.6 (C-5′), 129.8 (C-6′), 35.2 (C-7′), 54.1 (C-8′), 173.2 (C-9′), 136.7 (C-1), 133.2 (C-2 and C-6), 117.6 (C-3 and C-5), 152.6 (C-4), 28.3 (C-7), 151.1 (C-8), 163.3 (C-9) and 60.9 (OCH_3). [80]
N-acetyl-3,5-dibromo-4-hydroxyl phenylethamine (**156**)<br>$C_{10}H_{11}Br_2NO_2$<br>Bromotyrosine derivative	*Callyspongia* sp.<br>Australia	Spectroscopic data not provided. [80]

N-acetyl-3-bromo-4-hydroxy-phenylethamine (**157**) $C_{10}H_{12}BrNO_2$ Bromotyrosine derivative	*Callyspongia* sp. Australia	^{1}H NMR (600 MHz, DMSO-d_6, δ/ppm, *J*/Hz): 7.28 (d, 2.2, H-2), 6.84 (d, 8.2, H-5), 6.98 (dd, 2.2 and 8.2, H-6), 2.56 (t, 7.3, H-7), 3.18 (t, 7.3, H-8), 1.74 (s, H-2′), 9.94 (s, OH) and 7.82 (t, 5.6, NH). ^{13}C NMR (150 MHz, $CDCl_3$): 129.4 (C-1 and C-6), 133.2 (C-2), 109.6 (C-3), 152.9 (C-4), 116.7 (C-5), 34.4 (C-7), 40.8 (C-8), 169.8 (C-1′) and 23.4 (C-2′). [80]

[1] Classification based on the electronic database The Human Metabolome Database (HMDB) Version 4.0.
[2] Metabolite NMR data.

Table S5. Nucleosides isolated from *Callyspongia* species.

Metabolite name<br>Chemical formula<br>Type of metabolite	Specie<br>Geographic Location	^{1}H and ^{13}C NMR data [Reference]
1*H*-pyrimidine-2,4-dione (**158**)<br>$C_4H_4N_2O_2$<br>Diazine[1]	*Callyspongia fibrosa*<br>China	^{1}H NMR (500 MHz, Pyr-d$_5$, δ/ppm): 13.10 (1H, s), 12.45 (1H, s), 7.53 (1H, s) and 5.81 (1H, s). ^{13}C NMR (125 MHz, DMSO): 164.1 (s), 151.25 (s), 141.78 (d) and 100.04 (d). [45]
5-methylpyrimidine-2,4 (^{1}*H*, ^{3}*H*)-dione (**159**)<br>$C_5H_6N_2O_2$<br>Diazine[1]	*Callyspongia fibrosa*<br>China	^{1}H NMR (500 MHz, DMSO-d$_6$, δ/ppm): 10.92 (1H, s), 10.52 (1H, s), 7.20 (1H, s) and 1.70 (3H, s). ^{13}C NMR (125 MHz, CDCl$_3$): 165.04 (s), 151.62 (s), 137.86 (s), 107.81 (d) and 11.96 (q). [45]
1-(4-hydroxy-5-hydroxymethyl-tetrahydro-furan-2-yl)-5-methyl-^{1}*H*-pyrimidine-2,4-dione (**160**)<br>$C_{10}H_{14}N_2O_5$<br>Pyrimidine nucleoside[1]	*Callyspongia fibrosa*<br>China	^{1}H NMR (500 MHz, DMSO-d$_6$, δ/ppm): 11.25 (1H, s), 7.68 (1H, s), 6.15 (1H, t), 5.21 (1H, d), 5.00 (1H, t), 4.22 (1H, m), 3.74 (1H, q), 3.56 (2H, m), 2.05 (2H, m) and 1.76 (3H, s). ^{13}C NMR (125 MHz, DMSO-d$_6$): 163.5 (s), 150.3 (s), 135.9 (d), 109.2 (s), 87.1 (d), 83.7 (d), 70.7 (d), 61.3 (t), 39.3 (t) and 12.0 (q). [45]
1-(2′-Deoxy-*α*-*D*-ribofuranosyl)thymine (**161**)<br>$C_{10}H_{14}N_2O_5$<br>Nucleoside	*Callyspongia* sp.<br>Not reported	^{1}H NMR (500 MHz, DMSO-d$_6$, δ/ppm, *J*/Hz): 11.27 (1H, br s, 1-NH), 7.69 (1H, s, H-4), 6.16 (1H, t, 7.3, H-1′), 5.22 (1H, d, 4.2, 3′-OH), 5.01 (1H, t, 5.2, 5′-OH), 4.22 (1H, m, H-4′), 3.92 (1H, m, H-3′), 3.56 (2H, m, H-5′), 2.07 (2H, m, H-2′) and 1.76 (3H, s, 5-CH$_3$). ^{13}C NMR (125 MHz, DMSO-d$_6$): 163.5 (C-6), 150.6 (C-2), 139.4 (C-4), 110.7 (C-5), 94.3 (C-1′), 87.1 (C-4′), 70.2 (C-3′), 61.1 (C-5′), 40.3 (C-2′) and 13.2 (5-CH$_3$). [20]
2′-Deoxyuridine (**162**)<br>$C_9H_{12}N_2O_5$<br>Nucleoside	*Callyspongia* sp.<br>Not reported	^{1}H NMR (500 MHz, CD$_3$OD, δ/ppm, *J*/Hz): 8.00 (1H, d, 6.5, H-4), 6.82 (1H, t, 7.0, H-1′), 5.72 (1H, d, 6.5, H-5), 4.41 (1H, m, H-4′), 3.95 (1H, m, H-3′), 3.79 (1H, dd, 12.0 and 3.5, H-5′a), 3.73 (1H, dd, 12.0 and 4.0, H-5′b), 2.31 (1H, m, H-2′a) and 2.21 (1H, m, H-2′b). ^{13}C NMR (125 MHz, CD$_3$OD): 163.3 (C-6), 151.1 (C-2), 141.0 (C-4), 103.4 (C-5), 92.4 (C-1′), 87.2 (C-4′), 71.2 (C-3′), 61.3 (C-5′) and 40.3 (C-2′). [20]
Spongothymidine (**163**)<br>$C_{10}H_{14}N_2O_6$<br>Nucleoside	*Callyspongia* sp.<br>Not reported	^{1}H NMR (500 MHz, CD$_3$OD, δ/ppm, *J*/Hz): 7.83 (1H, s, H-4), 6.30 (1H, t, 7.0, H-1′), 4.42 (1H, m, H-3′), 4.24 (1H, m, H-4′), 3.93 (1H, m, H-2′), 3.83 (1H, dd, 12.0 and 3.0, H-5′a), 3.80 (1H, dd, 12.0 and 3.5, H-5′b) and 1.88 (3H, s, 5-CH$_3$). ^{13}C NMR (125 MHz, CD$_3$OD): 163.7 (C-6), 150.4 (C-2), 140.4 (C-4), 109.7 (C-5), 95.3 (C-1′), 87.0 (C-4′), 73.3 (C-2′), 70.2 (C-3′), 60.1 (C-5′) and 13.1 (5-CH$_3$). [20]
Spongouridine (**164**)<br>$C_9H_{12}N_2O_6$<br>Nucleoside	*Callyspongia* sp.<br>Not reported	^{1}H NMR (500 MHz, Py-d$_5$, δ/ppm, *J*/Hz): 13.01 (1H, br s, 1-NH), 8.53 (1H, d, 8.5, H-4), 7.73 (1H, br s, 5′-OH), 7.06 (2H, br s, 2′ and 3′-OH), 6.82 (1H, d, 3.5, H-1′), 5.80 (1H, d, 8.5, H-5), 4.91 (1H, m, H-3′), 4.90 (1H, m, H-4′), 4.65 (1H, m, H-2′), 4.31 (1H, d, 12.0, H-5′a) and 4.20 (1H, d, 12.0, H-5′b).

		^{13}C NMR (125 MHz, Pyr-d_5): 164.3 (C-6), 152.1 (C-2), 141.0 (C-4), 102.4 (C-5), 90.4 (C-1′), 86.2 (C-4′), 75.9 (C-2′), 71.1 (C-3′) and 61.7 (C-5′). [20]
2′-Deoxyadenosine (**165**)<br>$C_{10}H_{13}N_5O_3$<br>Nucleoside	*Callyspongia* sp.<br>Not reported	^{1}H NMR (500 MHz, CD_3OD, δ/ppm, *J*/Hz): 8.33 (1H, s, H-2), 8.19 (1H, s, H-8), 6.45 (1H, t, 6.2, H-1′), 4.59 (1H, m, H-4′), 4.09 (1H, m, H-3′), 3.86 (1H, dd, 12.0 and 3.0, H-5′a), 3.76 (1H, dd, 12.0 and 3.3, H-5′b), 2.83 (1H, m, H-2′a) and 2.43 (1H, m, H-2′b). ^{13}C NMR (125 MHz, CD_3OD): 157.6 (C-6), 153.5 (C-2), 150.1 (C-4), 141.6 (C-8), 120.1 (C-5), 90.1 (C-1′), 87.2 (C-4′), 73.1 (C-3′), 63.7 (C-5′) and 41.6 (C-2′). [20]
2′-Deoxyinosine (**166**)<br>$C_{10}H_{12}N_4O_4$<br>Nucleoside	*Callyspongia* sp.<br>Not reported	^{1}H NMR (500 MHz, DMSO-d_6, δ/ppm, *J*/Hz): 12.11 (1H, br s, 1-NH), 8.13 (1H, s, H-2), 8.07 (1H, s, H-8), 6.30 (1H, t, 6.9, H-1′), 5.32 (1H, d, 4.0, 3′-OH), 4.96 (1H, t, 5.5, 5′-OH), 4.38 (1H, m, H-4′), 3.94 (1H, m, H-3′), 3.61 (1H, m, H-5′a), 3.48 (1H, m, H-5′b) and 2.61 (2H, m, H-2′). ^{13}C NMR (125 MHz, DMSO-d_6): 166.2 (C-6), 157.2 (C-4), 155.2 (C-2), 148.0 (C-8), 133.9 (C-5), 97.1 (C-1′), 93.1 (C-4′), 80.1 (C-3′), 71.1 (C-5′) and 45.6 (C-2′). [20]
1-(2′-Deoxy-*β*-*D*-erythro-pentofuranosyl)-1*H*-1,2,4-triazole (**167**)<br>$C_7H_{11}N_3O_3$<br>Nucleoside	*Callyspongia* sp.<br>Not reported	^{1}H NMR (500 MHz, CD_3OD, δ/ppm, *J*/Hz): 8.35 (1H, s, H-4), 8.20 (1H, s, H-2), 6.45 (1H, t, 6.1, H-1′), 4.60 (1H, m, H-4′), 4.09 (1H, m, H-3′), 3.86 (1H, dd, 12.0 and 3.0, H-5′a), 3.76 (1H, dd, 12.0 and 3.3, H-5′b), 2.83 (1H, m, H-2′a) and 2.42 (1H, m, H-2′b). ^{13}C NMR (125 MHz, CD_3OD): 153.5 (C-2), 141.6 (C-4), 90.0 (C-1′), 87.2 (C-4′), 73.1 (C-3′), 63.7 (C-5′) and 41.6 (C-2′). [20]
1-(*β*-*D*-Ribofuranosyl)-1*H*-1,2,4-triazole (**168**)<br>$C_7H_{11}N_3O_4$<br>Nucleoside	*Callyspongia* sp.<br>Not reported	^{1}H NMR (500 MHz, CD_3OD, δ/ppm, *J*/Hz): 8.34 (1H, s, H-4), 8.20 (1H, s, H-2), 6.99 (1H, t, 6.4, H-1′), 4.76 (1H, m, H-3′), 4.34 (1H, m, H-4′), 4.19 (1H, m, H-2′), 3.86 (1H, dd, 12.0 and 3.0, H-5′a) and 3.76 (1H, dd, 12.0 and 3.3, H-5′b). ^{13}C NMR (125 MHz, CD_3OD): 153.6 (C-2), 142.1 (C-4), 91.3 (C-1′), 88.2 (C-4′), 75.5 (C-3′), 72.7 (C-2′) and 63.5 (C-5′). [20]

[1] Classification based on the electronic database The Human Metabolome Database (HMDB) Version 4.0.

Table S6. Cyclic peptides and cyclic depsipeptides isolated from *Callyspongia* species.

Metabolite name<br>Chemical formula<br>Type of metabolite	Specie<br>Geographic Location	^{1}H and ^{13}C NMR data [Reference]
	Callyspongia aerizusa<br>Indonesia	Spectroscopic data not provided (Reference 4 is cited). [22]
Callyaerin A (**169**)<br>$C_{69}H_{108}N_{14}O_{14}$<br>Cyclic peptide	*Callyspongia aerizusa*<br>Indonesia	^{1}H NMR (600 MHz, DMSO-d_6, δ/ppm, *J*/Hz): DAA: 8.29 (s, NH), 7.35 (d, 13.2, β); R1 Ile: 5.87 (dd, 13.1 and 10.2, NH), 4.08 (m, α), 1.41 (m, β), 1.41 (m, γ), 0.79 (m, γ), 0.79 (m, γ′δ), 0.42 (t, 7.3, γ′δ); R2 Hyp: 4.26 (m, α), 2.08 (m, β), 1.89 (m, β), 4.41 (m, γ), 3.77 (br d, δ), 3.70 (dd, 11.4 and 3.7, δ), 5.36 (d, 3.1, OH); R3 Val: 9.03 (br s, NH), 2.98 (dd, 10.7 and 7.1, α), 2.65 (m, β), 0.83 (d, 6.6, γ), 0.82 (d, 7.0, γ); R4 Ile: 8.81 (br s, NH), 3.79 (m, α), 1.49 (m, β), 1.40 (m, γ), 1.13 (m, γ), 0.81 (d, 6.9, γ′δ), 0.78 (t, 7.4, γ′δ); R5 Leu: 7.44 (d, 7.0, NH), 4.61 (m, α), 1.77 (m, β), 1.25 (m, β), 1.62 (m, γ), 0.87 (d, 6.5, δ, δ′), 0.86 (d, 6.6, δ, δ′); R6 Pro: 4.05 (m, α), 2.30 (m, β), 1.91 (m, β), 1.99 (m, γ), 1.91 (m, γ), 3.61 (m, δ), 3.47 (m, δ); R7 Pro: 4.27 (m, α), 2.25 (m, β), 1.61 (m, β), 1.94 (m, γ), 1.86 (m, γ), 3.59 (m, δ), 3.31 (m, δ); R8 Leu: 6.72 (d, 10.0, NH), 4.61 (m, α), 1.89 (m, β), 1.57 (m, β), 1.65 (m, γ), 0.96 (d, 6.6, δ, δ′), 0.85 (d, 6.6, δ, δ′); C1 Pro: 4.25 (m, α), 2.26 (m, β), 1.50 (m, β), 1.83 (m, γ), 1.72 (m, γ), 3.51 (m, δ), 3.21 (m, δ); C2 Ile: 7.47 (m, NH), 3.79 (m, α), 1.67 (m, β), 1.17 (m, γ), 1.05 (m, γ), 0.39 (d, 6.8, γ′δ), 0.70 (t, 7.3, γ′δ); C3 Phe: 7.27 (d, 8.6, NH), 4.27 (m, α), 3.09 (dd, 13.9 and 2.9, β), 2.63 (dd, 14.0 and 12.1, β), 7.19-7.11 (m); C4 Gly: 8.15 (dd, 6.8 and 5.5, NH), 3.91 (dd, 16.5 and 7.0, α), 3.48 (dd, 16.5 and 5.3, α), 7.24 (br s, NH_2) and 6.97 (br s, NH_2). ^{13}C NMR (DMSO-d_6): DAA: 167.7 (s, CO), 98.2 (s, α), 143.4 (d, β); R1 Ile: 172.0 (s, CO), 64.4 (d, α), 37.8 (d, β), 24.0 (t, γ), 14.2 (q, γ′δ), 10.5 (q, γ′δ); R2 Hyp: 173.3 (s, CO), 55.6 (d, α), 37.7 (t, β), 68.7 (d, γ), 56.7 (t, δ); R3 Val: 172.0 (s, CO), 66.1 (d, α), 27.1 (t, β), 19.2 (q, γ), 19.5 (q, γ); R4 Ile: 171.2 (s, CO), 59.3 (d, α), 36.1 (d, β), 15.2 (q, γ′δ), 10.2 (q, γ′δ); R5 Leu: 172.3 (s, CO), 49.1 (d, α), 41.1 (t, β), 21.0 (q, δ, δ′), 21.0 (q, δ, δ′); R6 Pro: 171.4 (s, CO), 64.0 (d, α), 26.2 (t, β), 46.1 (t, δ); R7 Pro: 171.4 (s, CO), 62.5 (d, α), 28.5 (t, β), 47.0 (t, δ); R8 Leu: 172.4 (s, CO), 50.0 (d, α), 40.5 (t, β), 22.8 (q, δ, δ′), 23.2 (q, δ, δ′); C1 Pro: 172.7 (s, CO), 61.9 (d, α), 29.4 (t, β), 48.7 (t, δ); C2 Ile: 171.1 (s, CO), 58.6 (d, α), 34.9 (d, β), 14.9 (q, γ′δ), 11.0 (q, γ′δ); C3 Phe: 171.5 (s, CO), 59.3 (d, α), 39.9 (t, β), 138.0 (s, C-1), 129.0 (d, C-2, C-6), 127.7 (d, C-3, C-5), 126.1 (d, C-4); C4 Gly: 170.8 (s, CO) and 42.0 (t, α). [4]
Callyaerin B (**170**)<br>$C_{65}H_{109}N_{13}O_{13}$<br>Cyclic peptide	*Callyspongia aerizusa*<br>Indonesia	^{1}H NMR (600 MHz, DMSO-d_6, δ/ppm, *J*/Hz): DAA: 8.28 (br s, NH), 7.04 (d, 13.6, β); R1 Ile: 5.70 (dd, 13.6 and 10.8, NH), 3.94 (t, 9.8, α), 1.45 (m, β), 1.40 (m, γ), 1.00 (m, γ), 0.85 (γ′δ), 0.76 (t, 7.5, γ′δ); R2 Hyp: 4.24 (m, α), 2.10 (m, β), 1.89 (m, β), 4.40 (br s, γ), 3.67

		(m, δ), 3.80 (m, δ), 5.40 (d, 2.4, OH); R3 Ile: 9.00 (br s, NH), 3.07 (m, α), 2.55 (m, β), 1.40 (m, γ), 1.10 (m, γ), 0.82 (t, γ′δ), 0.78 (γ′δ); R4 Ile: 8.86 (br s, NH), 3.81 (8.8, α), 1.55 (m, β), 1.45 (m, γ), 1.15 (m, γ), 0.84 (γ′δ), 0.81 (t, γ′δ); R5 Leu: 7.37 (d, 6.6, NH), 4.62 (q, ~6.7, α), 1.76 (m, β), 1.30 (m, β), 1.60 (m, γ), 0.88 (δ, δ′); R6 Pro: 4.03 (t, 8.2, α), 2.30 (m, β), 1.90 (m, β), 2.00 (m, γ), 1.90 (2.00 (m, γ), 3.60 (m, δ), 3.48 (m, δ); R7 Pro: 4.25 (m, α), 2.20 (m, β), 1.82 (m, β), 1.90 (m, γ), 1.50 (m, γ), 3.55 (m, δ), 3.35 (m, δ); R8 Leu: 6.63 (10.0, NH), 4.55 (ddd, 10.7, 10.4 and 3.7, α), 1.70 (m, β), 1.60 (m, β), 1.47 (m, γ), 0.88 (δ, δ′), 0.81 (δ, δ′); C1 Pro: 4.25 (m, α), 2.55 (m, β), 1.80 (m, β), 1.70 (m, γ), 1.45 (m, γ), 3.52 (m, δ), 3.15 (m, δ); C2 Ile: 7.58 (d, 10.4, NH), 4.10 (dd, 9.4 and 9.2, α), 1.82 (m, β), 1.45 (m, γ), 1.15 (m, γ), 0.86 (γ′δ), 0.82 (t, γ′δ); C3 Ile: 7.23 (d, 9.5, NH), 4.15 (dd, 9.5 and 5.0, α), 1.90 (m, β), 1.25 (m, γ), 1.00 (m, γ), 0.78 (γ′δ), 0.71 (t, 7.5, γ′δ), 7.17 (s, NH_2) and 6.97 (s, NH_2). ^{13}C NMR (150 MHz, DMSO-d_6): DAA: 167.2 (s, CO), 97.9 (s, α), 142.6 (d, β); R1 Ile: 172.1 (s, CO), 64.6 (d, α), 37.9 (d, β), 29.1 (t, γ); R2 Hyp: 173.4 (s, CO), 59.2 (d, α), 37.5 (t, β), 68.7 (d, γ), 56.7 (t, δ); R3 Ile: 172.1 (s, CO), 64.2 (d, α), 32.4 (d, β), 24.5 (t, γ); R4 Ile: 171.3 (s, CO), 57.8 (d, α), 36.2 (d, β), 25.5 (t, γ); R5 Leu: 171.4 (s, CO), 49.1 (d, α), 41.1 (t, β), 24.7 (d, γ); R6 Pro: 172.3 (s, CO), 63.9 (d, α), 24.9 (t, β), 29.1 (t, γ), 46.1 (t, δ); R7 Pro: 171.4 (s, CO), 61.3 (d, α), 25.7 (t, β), 29.7 (t, γ), 47.0 (t, δ); R8 Leu: 172.7 (s, CO), 50.0 (d, α), 40.5 (t, β), 24.6 (d, γ); C1 Pro: 171.2 (s, CO), 62.5 (d, α), 26.2 (t, β), 29.7 (t, γ), 48.6 (t, δ); C2 Ile: 172.1 (s, CO), 57.8 (d, α), 36.4 (d, β), 25.0 (t, γ); C3 Ile: 172.7 (s, CO), 57.4 (d, α), 36.2 (d, β) and 23.5 (t, γ). [4]
	Callyspongia aerizusa Indonesia	Spectroscopic data not provided (Reference 4 is cited). [22]
	Callyspongia aerizusa Indonesia	Spectroscopic data not provided (Reference 4 is cited). [22]
Callyaerin C (**171**) $C_{63}H_{93}N_{15}O_{13}$ Cyclic peptide	*Callyspongia aerizusa* Indonesia	^{1}H NMR (600 MHz, DMSO-d_6, δ/ppm, *J*/Hz): DAA: 8.57 (s, NH), 7.65 (d, 13.7, β); R1 His: 5.64 (dd, 13.7 and 9.9, NH), 4.48 (ddd, 10.5, 9.9 and 4.0, α), 2.33 (m, β), 2.20 (m, β), 12.03 (s, NH1/3), 7.39 (s, 2H), 6.01 (s, 4H); R2 Hyp: 4.14 (dd, 9.2 and 4.3, α), 2.07 (m, β), 1.64 (m, β), 4.06 (m, γ), 3.54 (m, δ), 1.81 (m, δ), 5.09 (d, 3.8, OH); R3 Leu: 9.84 (d, 7.7, NH), 4.16 (ddd, 11.4, 7.6 and 3.9, α), 1.59 (m, β), 1.51 (m, β), 1.73 (m, γ), 0.86 (d, 6.6, δ), 0.82 (d, 6.5, δ); R4 Leu: 7.57 (d, 6.3, NH), 4.42 (ddd, 9.1, 6.1 and 5.1, α), 1.63 (m, β), 1.22 (m, β), 1.47 (m, γ), 0.89 (d, 6.6, δ), 0.89 (d, 6.5, δ); R5 Pro: 4.30 (dd, 9.0 and 8.0, α), 2.34 (m, β), 1.58 (m, β), 1.92 (m, γ), 3.67 (m, δ), 3.19 (m, δ); R6 Pro: 4.39 (dd, 11.4 and 7.2, α), 2.20 (m, β), 1.89 (m, β), 2.00 (m, γ), 3.56 (m, δ); R7 Val: 7.32 (d, 10.3, NH), 4.67 (dd, 10.2 and 4.4, α), 2.54 (m, β), 1.11 (d, 7.0, γ), 0.97 (d, 6.9, γ); C1 Pro: 4.32 (dd, 10.8 and 7.0, α), 2.29 (m, β), 1.53 (m, β), 1.88 (m, γ), 1.77 (m, γ), 3.48 (m,

		δ), 3.28 (m, δ); C2 Leu: 7.90 (d, 6.2, NH), 3.90 (ddd, 11.1, 7.2 and 3.8, α), 1.64 (m, β), 1.16 (m, β), 1.64 (m, γ), 0.87 (d, 6.5, δ), 0.75 (d, 6.5, δ); C3 Phe: 7.80 (d, 9.3, NH), 4.24 (ddd, 12.1, 9.3 and 3.1, α), 3.30 (m, β), 2.98 (dd, 13.3 and 12.0, β), 7.35 (m, H-2, H-6), 7.15 (m, H-3, H-4, H-5); G4 Gly: 7.74 (dd, 7.1 and 5.1, NH), 3.88 (dd, 17.0 and 7.2, α), 3.54 (dd, 16.8 and 5.0, α), 7.20 (br s, NH_2) and 7.08 (br s, NH_2). ^{13}C NMR (150 MHz, DMSO-d_6): DAA: 167.8 (s, CO), 99.1 (s, α), 143.3 (d, β); R1 His: 172.3 (s, CO), 59.7 (d, α), 34.3 (t, β), 134.9 (d, C-2), 114.6 (d, C-4); R2 Hyp: 171.7 (s, CO), 61.6 (d, α), 37.6 (t, β), 68.5 (d, γ), 54.3 (t, δ); R3 Leu: 172.5 (s, CO), 52.2 (d, α), 39.4 (t, β), 20.7 (q, δ); R4 Leu: 169.9 (s, CO), 49.2 (d, α), 39.7 (t, β); R5 Pro: 172.0 (s, CO), 62.9 (d, α), 29.2 (t, β), 47.2 (t, δ); R6 Pro: 172.0 (s, CO), 63.9 (d, α), 26.8 (t, β), 46.1 (t, δ); R7 Val: 171.8 (s, CO), 56.6 (d, α), 29.3 (d, β), 17.4 (q, γ), 18.9 (q, γ); C1 Pro: 173.7 (s, CO), 62.3 (d, α), 29.1 (t, β), 48.6 (t, δ); C2 Leu: 171.9 (s, CO), 52.6 (d, α), 38.4 (t, β), 20.7 (q, δ); C3 Phe: 171.0 (s, CO), 55.1 (d, α), 37.5 (t, β), 129.6 (d, C-2, C-6), 127.9 (d, C-3, C-5), 126.4 (d, C-4); C4 Gly: 170.7 (s, CO) and 42.1 (t, α). [4]
Callyaerin D (**172**)<br>$C_{69}H_{107}N_{15}O_{15}$<br>Cyclic peptide	*Callyspongia aerizusa*<br>Indonesia	^{1}H NMR (600 MHz, DMSO-d_6, δ/ppm, *J*/Hz): DAA: 8.42 (br s, NH), 7.38 (d, 8.8, β); R1 Ile: 5.30 (t, 10.7, NH), 4.41 (m, α), 1.98 (m, β), 0.94 (m, γ), 1.20 (m, γ), 0.83 (γ′δ), 0.84 (γ′δ); R2 Ile: 7.64 (d, 4.9, NH), 4.00 (dd, 4.9 and 3.5, α), 1.81 (m, β), 1.25 (m, γ), 1.30 (m, γ), 0.80 (m, γ′δ); R3 Phe: 7.50 (d, 6.0, NH), 4.61 (m, α), 3.06 (dd, 13.1 and 9.7, β), 2.77 (dd, 13.1 and 3.5, β), 7.25 (d, 8.8), 7.23 (d, 6.9), 7.21 (d, 7.6); R4 Pro: 4.17 (t, 7.1, α), 1.40 (m, β), 1.70 (m, β), 1.42 (m, γ), 1.66 (m, γ), 2.23 (m, δ), 3.27 (m, δ); R5 Hyp: 4.40 (m, α), 2.00 (m, β), 2.21 (m, β), 1.91 (m, γ), 3.65 (m, δ), 3.93 (t, 6.3, δ); R6 Pro: 4.30 (t, 8.6, α), 1.75 (m, β), 2.17 (m, β), 1.60 (m, γ), 1.70 (m, γ), 3.30 (m, δ), 3.53 (t, 8.8, δ); R7 Leu: 7.54 (d, 9.5, NH), 4.47 (td, 9.4 and 5.5, α), 1.85 (m, β), 1.71 (m, β), 1.70 (m, γ), 0.80 (m, δ), 0.82 (m, δ); C1 Pro: 4.32 (m, α), 1.70 (m, β), 2.16 (m, β), 1.80 (m, γ), 1.90 (m, γ), 3.61 (m, δ), 3.38 (m, δ); C2 Ile: 7.76 (d, 6.3, NH), 3.93 (t, 6.3, α), 1.90 (m, β), 1.40 (m, γ), 1.28 (m, γ), 0.80 (γ′δ), 0.85 (γ′δ); C3 Asn: 7.88 (d, 7.6, NH), 4.55 (q, ~7, α), 2.69 (dd, 15.1 and 6.9, β), 2.35 (dt, 15.1 and 6.9, β), 6.93 (s, NH_2), 7.31 (s, NH_2); C4 Ala: 7.57 (d, 6.9, NH), 4.16 (m, α), 1.25 (d, 6.9, β); C5 Ile: 7.34 (d, 6.7, NH), 4.05 (dd, 8.9 and 6.7, α), 1.77 (m, β), 1.12 (m, γ), 1.45 (m, γ), 0.85 (γ′δ) and 0.87 (γ′δ). [4]
Callyaerin D (**173**)<br>$C_{69}H_{107}N_{15}O_{15}$<br>Cyclic peptide	*Callyspongia aerizusa*<br>Indonesia	^{1}H NMR (700 MHz, DMSO-d_6, δ/ppm, *J*/Hz): DAA: 8.42 (br s, NH), 7.38 (d, 13.9, β); R1 Ile: 5.30 (dd, 13.9 and 10.0, NH), 4.41 (α), 1.98 (m, β), 1.25 (m, γ), 0.94 (m, γ), 0.84 (γ′), 0.83 (δ); R2 Hyp: 4.13 (α), 2.06 (m, β), 1.97 (m, β), 4.40 (γ), 3.93 (m, δ), 3.65 (br d, 10.9, δ), 5.19 (d, 3.8, OH); R3 Ile: 7.64 (d, 4.9, NH), 4.00 (dd, 4.9 and 3.5, α), 1.81 (m, β), 1.30 (m, γ), 1.25 (m, γ), 0.82 (γ′), 0.82 (δ); R4 Phe: 7.50 (d, 6.0, NH), 4.61 (ddd, 9.7, 6.0 and 3.5, α), 3.06 (dd, 13.1 and 9.7,

		β), 2.77 (dd, 13.1 and 3.5, β), 7.24 (br d, 7.2, H-2, H-6), 7.30 (br t, 7.2, H-3, H-5), 7.22 (br t, 7.2, H-4); R5 Pro: 4.32 (α), 2.16 (m, β), 1.80 (m, β), 1.92 (m, γ), 1.90 (m, γ), 3.61 (t, 8.8, δ), 3.38 (m, δ); R6 Pro: 4.16 (α), 2.23 (m, β), 1.40 (m, β), 1.69 (m, γ), 1.47 (m, γ), 3.27 (m, δ), 2.23 (m, δ); R7 Leu: 7.54 (d, 9.5, NH), 4.47 (td, 9.5 and 5.5, α), 1.85 (m, β), 1.71 (m, β), 1.76 (m, γ), 0.85 (δ), 0.87 (δ′); C1 Pro: 4.30 (α), 2.17 (m, β), 1.60 (m, β), 1.79 (m, γ), 1.71 (m, γ), 3.53 (br t, 9.5, δ), 3.30 (m, δ); C2 Ile: 7.76 (d, 6.3, NH), 3.93 (t, 6.3, α), 1.90 (m, β), 1.40 (m, γ), 1.28 (m, γ), 0.87 (γ′), 0.82 (δ); C3 Asn: 7.88 (d, 7.6, NH), 4.55 (q, 7.0, α), 2.69 (dd, 15.1 and 7.0, β), 2.35 (dd, 15.1 and 7.0, β), 6.93 (br s, NH$_2$), 7.31 (br s, NH$_2$); C4 Ala: 7.57 (d, 6.9, NH), 4.17 (α), 1.25 (d, 6.9, β); C5 Ile: 7.34 (d, 6.7), 4.05 (dd, 8.9 and 6.7, α), 1.77 (m, β), 1.12 (m, γ), 1.45 (m, γ), 0.83 (γ′), 0.83 (δ), 7.11 (br s, NH$_2$) and 7.03 (br s, NH$_2$). ^{13}C NMR (176 MHz, DMSO-d_6): DAA: 167.2 (CO), 98.7 (α), 143.6 (β); R1 Ile: 172.7 (CO), 63.8 (α), 35.9 (β), 22.3 (γ), 15.1 (γ′), 11.3 (δ); R2 Hyp: 172.6 (CO), 62.1 (α), 37.5 (β), 68.9 (γ), 55.2 (δ); R3 Ile: 170.5 (CO), 58.7 (α), 35.7 (β), 24.7 (γ), 15.1 (γ′), 11.1 (δ); R4 Phe: 169.0 (CO), 53.4 (α), 36.3 (β), 138.0 (C-1), 129.0 (C-2, C-6), 128.2 (C-3, C-5), 126.5 (C-4); R5 Pro: 171.5 (CO), 63.7 (α), 26.9 (β), 24.3 (γ), 46.3 (δ); R6 Pro: 171.6 (CO), 62.8 (α), 28.6 (β), 25.5 (γ), 46.5 (δ); R7 Leu: 172.5 (CO), 50.6 (α), 41.2 (β), 24.3 (γ), 22.3 (δ), 21.1 (δ′); C1 Pro: 173.6 (CO), 61.6 (α), 28.7 (β), 25.7 (γ), 48.5 (δ); C2 Ile: 171.5 (CO), 58.7 (α), 35.0 (β), 24.6 (γ), 15.1 (γ′), 11.0 (δ); C3 Asn: 171.1 (CO), 50.0 (α), 37.2 (β), 171.3 (CO(γ)); C4 Ala: 171.9 (CO), 48.8 (α), 17.1 (β); C5 Ile: 172.9 (CO), 57.1 (α), 36.2 (β), 24.1 (γ), 15.1 (γ′) and 11.1 (δ). [22]
	Callyspongia aerizusa Indonesia	Spectroscopic data not provided (Reference 4 is cited). [22]
Callyaerin E (**174**) $C_{66}H_{95}N_{13}O_{12}$ Cyclic peptide	*Callyspongia aerizusa* Indonesia	^{1}H NMR (600 MHz, DMSO-d_6, δ/ppm, *J*/Hz): DAA: 8.36 (s, NH), 7.28 (d, 13.6, β); R1 Leu: 5.27 (dd, 13.3 and 10.0, NH), 4.36 (m, α), 1.77 (m, β), 1.23 (m, β), 1.60 (m, γ), 0.96 (d, 6.0, δ, δ′), 0.94 (d, 6.6, δ, δ′); R2 Pro: 4.01 (dd, 9.9 and 5.5, α), 2.16 (m, β), 1.67 (m, β), 2.06 (m, γ), 1.88 (m, γ), 3.93 (br t, 8.8, δ), 3.59 (m, δ); R3 Phe: 7.73 (d, 6.7, NH), 4.39 (m, α), 3.00 (m, β), 7.17-7.32 (m, Aromatic); R4 Phe: 7.31 (d, 6.9, NH), 4.63 (ddd, 8.8, 6.9 and 4.7, α), 3.00 (m, β), 2.71 (dd, 13.3 and 4.7, β), 7.17-7.30 (m, Aromatic); R5 Pro: 4.41 (m, α), 2.15 (m, β), 1.82 (m, β), 1.93 (m, γ), 1.89 (m, γ), 3.49 (br t, 8.8, δ), 3.42 (m, δ); R6 Pro: 4.16 (dd, 7.9 and 9.8, α), 2.22 (m, β), 1.41 (m, β), 1.74 (m, γ), 1.59 (m, γ), 3.34 (m, δ), 2.48 (m, δ); R7 Val: 7.03 (d, 10.1, NH), 4.56 (dd, 10.1 and 3.9, α), 2.33 (m, β), 1.06 (d, 6.9, γ), 0.97 (d, 6.8, γ); C1 Pro: 4.36 (m, α), 2.22 (m, β), 1.57 (m, β), 1.78 (m, γ), 1.68 (m, γ), 3.57 (m, δ), 3.21 (m, δ); C2 Ile: 7.62 (d, 7.9, NH), 3.99 (dd, 7.9 and 7.3, α), 1.95 (m, β), 1.45 (m, γ), 1.21 (m, γ), 0.88 (d, 6.9, γδ), 0.85 (t, 6.9, γδ); C3 Ile: 7.37 (d, 8.0, NH), 4.08 (t, 7.6, α), 1.82

		(m, β), 1.39 (m, γ), 1.27 (m, γ), 0.87 (d, 6.9, γδ), 0.80 (t, 6.7, γδ); G4 Gly: 7.88 (t, 6.0, NH), 3.72 (dd, 16.7 and 6.2, α), 3.57 (dd, 16.7 and 5.9, α), 7.04 (br s, NH_2) and 6.97 (br s, NH_2). ^{13}C NMR (DMSO-d_6): DAA: 167.5 (s, CO), 98.8 (s, α), 143.3 (d, β); R1 Leu: 170.8 (CO), 57.9 (d, α), 41.8 (t, β), 26.5 (d, γ), 22.5 (q, δ, δ′), 23.2 (q, δ, δ′); R2 Pro: 171.9 (s, CO), 63.9 (d, α), 29.1 (t, β), 24.9 (t, γ), 47.8 (d, δ); R3 Phe: 168.9 (s, CO), 54.1 (d, α), 35.1 (t, β), 126.3 (Aromatic), 128.5 (Aromatic), 138.3 (Aromatic); R4 Phe: 171.2 (s, CO), 53.1 (d, α), 36.1 (t, β), 137.3 (s, C-1, Aromatic), 129.5 (d, C-2, C-6, Aromatic), 129.1 (d, C-3, C-5, Aromatic), 126.5 (d, C4, Aromatic); R5 Pro: 171.3 (s, CO), 62.8 (d, α), 28.4 (t, β), 24.5 (t, γ), 47.2 (d, δ); R6 Pro: 171.5 (s, CO), 64.0 (d, α), 29.6 (t, β), 25.8 (t, γ), 49.5 (d, δ); R7 Val: 171.2 (s, CO), 56.5 (d, α), 28.8 (d, β), 18.4 (q, γ), 18.3 (q, γ); C1 Pro: 172.4 (s, CO), 59.5 (d, α), 26.6 (t, β), 23.8 (t, γ), 45.8 (d, δ); C2 Ile: 171.1 (s, CO), 58.3 (d, α), 35.3 (d, β), 24.6 (t, γ), 15.9 (q, γδ), 11.4 (q, γδ); C3 Ile: 170.8 (s, CO), 57.8 (d, α), 34.2 (d, β), 25.1 (t, γ), 15.0 (q, γδ), 10.8 (q, γδ); C4 Gly: 170.9 (s, CO) and 42.3 (t, α). [4]
Callyaerin F (**175**)<br>$C_{58}H_{83}N_{11}O_{10}$<br>Cyclic peptide	*Callyspongia aerizusa*<br>Indonesia	^1H NMR (600 MHz, DMSO-d_6, δ/ppm, *J*/Hz): DAA: 8.25 (s, NH), 7.25 (d, 14.0, β); R1 Val: 5.14 (dd, 10.1 and 13.7, NH), 4.38 (br d, 9.8, α), 2.31 (m, β), 0.87 (d, 6.9, γ), 0.66 (d, 6.9, γ); R2 Pro: 3.95 (dd, 10.3 and 6.7, α), 2.17 (m, β), 1.81 (m, β), 2.03 (m, γ), 1.84 (m, γ), 3.86 (m, δ), 3.74 (m, δ); R3 Val: 7.54 (d, 5.1, NH), 3.86 (m, α), 2.11 (m, β), 0.93 (d, 6.9, γ), 0.86 (d, 6.9, γ); R4 Phe: 7.50 (d, 6.1, NH), 4.54 (ddd, 9.3, 6.1 and 3.5, α), 3.01 (dd, 12.9 and 9.7, β), 2.71 (dd, 12.8 and 3.1, β), 7.14-7.28 (m); R5 Pro: 4.28 (m, α), 2.09 (m, β), 1.80 (m, β), 1.91 (m, γ), 1.83 (m, γ), 3.58 (m, δ), 3.35 (m, δ); C1 Pro: 4.26 (m, α), 2.19 (m, β), 1.42 (m, β), 1.73 (m, γ), 1.45 (m, γ), 3.26 (m, δ), 2.31 (m, δ); C2 Leu: 7.66 (d, 9.6, NH), 4.62 (td, 10.1 and 3.9, α), 1.93 (m, β), 1.67 (m, β), 1.72 (m, γ), 0.94 (d, 6.9, δ), 0.84 (d, 6.9, δ); C3 Phe: 6.17 (d, 7.8, NH), 4.42 (td, 7.6 and 4.5, α), 2.93 (m, β), 7.14-7.28 (m); C4 Ile: 7.52 (d, 8.5, NH), 3.98 (dd, 8.3 and 6.1, α), 1.75 (m, β), 1.29 (m, γ), 1.10 (m, γ), 0.76 (d, 6.8, γ′δ), 0.77 (t, 7.3, γ′δ), 7.04 (br s, NH_2) and 6.95 (br s, NH_2). [4]
Callyaerin F (**176**)<br>$C_{58}H_{83}N_{11}O_{10}$<br>Cyclic peptide	*Callyspongia aerizusa*<br>Indonesia	^1H NMR (700 MHz, DMSO-d_6, δ/ppm, *J*/Hz): DAA: 8.25 (br s, NH), 7.25 (d, 14.0, β); R1 Val: 5.14 (dd, 14.0 and 10.1, NH), 4.38 (br d, 9.8, α), 2.31 (m, β), 0.87 (d, 6.9, γ), 0.66 (d, 6.9, γ′); R2 Pro: 3.95 (dd, 10.3 and 6.7, α), 2.17 (m, β), 1.81 (m, β), 2.03 (m, γ), 1.84 (m, γ), 3.86 (m, δ), 3.74 (m, δ); R3 Val: 7.54 (d, 5.1, NH), 3.86 (m, α), 2.11 (m, β), 0.93 (d, 6.7, γ), 0.86 (d, 6.7, γ′); R4 Phe: 7.50 (d, 6.1, NH), 4.54 (ddd, 9.3, 6.1 and 3.5, α), 3.01 (dd, 12.9 and 9.3, β), 2.71 (dd, 12.9 and 3.5, β), 7.21 (H-2, H-6, Aromatic), 7.28 (H-3, H-5, Aromatic), 7.23 (H-4, Aromatic); R5 Pro: 4.28 (m, α), 2.09 (m, β), 1.80 (m, β), 1.91 (m, γ), 1.83 (m, γ), 3.58 (br dd, 10.0 and 7.0, δ), 3.35 (m, δ); R6 Pro: 4.26 (m, α), 2.29 (m, β), 1.42 (m, β), 1.73 (m, γ), 1.45 (m,

		γ), 3.23 (br dd, 10.2 and 7.4, δ), 2.17 (m, δ); R7 Leu: 7.66 (d, 9.6, NH), 4.62 (td, 10.1 and 3.9, α), 1.93 (m, β), 1.67 (m, β), 1.72 (m, γ), 0.94 (d, 6.7, δ), 0.84 (d, 6.2, δ′); C1 Phe: 6.17 (d, 7.8, NH), 4.42 (td, 7.6 and 4.5, α), 2.93 (m, β), 2.90 (m, β), 7.18 (H-2, H-6, Aromatic), 7.28 (H-3, H-5, Aromatic), 7.15 (H-4, Aromatic); C2 Ile: 7.52 (d, 8.5, NH), 3.98 (dd, 8.3 and 6.1, α), 1.73 (m, β), 1.29 (m, γ), 1.10 (m, γ), 0.77 (t, 7.3, γ′), 0.76 (d, 6.8, δ), 7.04 (br s, NH_2) and 6.95 (br s, NH_2). ^{13}C NMR (176 MHz, DMSO-d_6): DAA: 165.8 (CO), 97.9 (α), 142.7 (β); R1 Val: 172.5 (CO), 63.7 (α), 29.6 (β), 18.4 (γ), 15.4 (γ′); R2 Pro: 172.5 (CO), 63.8 (α), 29.2 (β), 24.8 (γ), 46.6 (δ); R3 Val: 170.7 (CO), 59.8 (α), 29.0 (β), 18.4 (γ), 18.3 (γ′); R4 Phe: 168.7 (CO), 53.8 (α), 36.4 (β), 138.1 (C-1, Aromatic), 128.9 (C-2, C-6, Aromatic), 128.1 (C-3, C-5, Aromatic), 126.5 (C-4, Aromatic); R5 Pro: 170.9 (CO), 63.4 (α), 27.0 (β), 24.3 (γ), 46.4 (δ); R6 Pro: 171.5 (CO), 63.1 (α), 28.7 (β), 25.7 (γ), 46.5 (δ); R7 Leu: 171.8 (CO), 50.2 (α), 40.1 (β), 24.4 (γ), 21.0 (δ), 22.9 (δ′); C1 Phe: 171.0 (CO), 54.0 (α), 36.8 (β), 136.9 (C-1, Aromatic), 129.4 (C-2, C-6, Aromatic), 128.4 (C-3, C-5, Aromatic), 126.1 (C-4, Aromatic); C2 Ile: 173.1 (CO), 57.6 (α), 36.0 (β), 23.8 (γ), 15.9 (γ′) and 11.0 (δ). [22]
Callyaerin G (**177**)<br>$C_{69}H_{91}N_{13}O_{12}$<br>Cyclic peptide	*Callyspongia aerizusa*<br>Indonesia	^{1}H NMR (700 MHz, DMSO-d_6, δ/ppm, *J*/Hz): DAA: 8.23 (br s, NH), 7.46 (d, 13.9, β); R1 Leu: 5.30 (dd, 13.9 and 10.0, NH), 4.27 (α), 1.62 (m, β), 1.08 (m, β), 1.33 (m, γ), 0.53 (d, 6.7, δ), 0.37 (d, 6.4, δ′); R2 Pro: 3.94 (α), 2.10 (m, β), 1.59 (m, β), 2.01 (m, γ), 1.84 (m, γ), 3.92 (m, δ), 3.49 (m, δ); R3 Phe: 7.71 (d, 6.8, NH), 4.22 (α), 2.86 (m, β), 7.12 (br d, 7.2, H-2, H-6, Aromatic), 7.25 (br t, 7.2, H-3, H-5, Aromatic), 7.19 (br t, 7.2, H-4, Aromatic); R4 Phe: 7.21 (NH), 4.63 (q, 7.1, α), 2.96 (m, β), 2.66 (dd, 13.4 and 4.7, β), 7.18 (br d, 7.2, H-2, H-6, Aromatic), 7.25 (br t, 7.2, H-3, H-5, Aromatic), 7.22 (br t, 7.2, H-4, Aromatic); R5 Pro: 4.41 (dd, 9.8 and 7.9, α), 2.15 (m, β), 1.83 (m, β), 1.92 (m, γ), 1.90 (m, γ), 3.45 (m, δ), 3.38 (m, δ); R6 Pro: 4.24 (dd, 10.1 and 7.4, α), 2.28 (m, β), 1.48 (m, β), 1.78 (m, γ), 1.67 (m, γ), 3.40 (m, δ), 2.74 (td, 10.3 and 6.2, δ); R7 Leu: 7.51 (d, 10.1, NH), 4.46 (dt, 10.1 and 3.8, α), 1.92 (m, β), 1.75 (m, β), 1.75 (m, γ), 0.86 (d, 6.5, δ), 0.83 (d, 6.2, δ′); C1 Pro: 4.34 (dd, 9.8 and 7.9, α), 2.08 (m, β), 1.83 (m, β), 1.95 (m, γ), 1.78 (m, γ), 3.86 (m, δ), 3.36 (m, δ); C2 Pro: 4.11 (dd, 10.0 and 7.5, α), 1.89 (m, β), 1.06 (m, β), 1.69 (m, γ), 3.84 (m, δ), 3.37 (m, δ); C3 Phe: 7.67 (d, 7.0, NH), 4.23 (α), 3.23 (dd, 13.4 and 2.7, β), 2.92 (m, β), 7.35 (br d, 7.2, H-2, H-6, Aromatic), 7.25 (br t, 7.2, H-3, H-5, Aromatic), 7.17 (br t, 7.2, H-4, Aromatic); G4 Gly: 7.96 (dd, 7.9 and 5.0, NH), 3.91 (dd, 17.2 and 7.9, α), 3.41 (dd, 17.2 and 5.0, α), 7.20 (br s, NH_2) and 7.14 (br s, NH_2). ^{13}C NMR (176 MHz, DMSO-d_6): DAA: 168.0 (CO), 98.0 (α), 145.3 (β); R1 Leu: 173.3 (CO), 57.7 (α), 42.0 (β), 23.3 (γ), 23.3 (δ), 20.2 (δ′); R2 Pro: 172.2 (CO), 63.0 (α), 28.7 (β), 24.9 (γ), 46.0 (δ); R3 Phe: 170.7

		(CO), 53.8 (α), 35.2 (β), 138.3 (C-1, Aromatic), 128.5 (C-2, C-6, Aromatic), 128.1 (C-3, C-5, Aromatic), 126.5 (C-4, Aromatic); R4 Phe: 169.6 (CO), 52.3 (α), 36.4 (β), 137.7 (C-1, Aromatic), 129.2 (C-2, C-6, Aromatic), 128.1 (C-3, C-5, Aromatic), 126.6 (C-4, Aromatic); R5 Pro: 171.7 (CO), 63.5 (α), 26.5 (β), 24.0 (γ), 46.1 (δ); R6 Pro: 171.4 (CO), 62.5 (α), 28.5 (β), 25.4 (γ), 46.5 (δ); R7 Leu: 171.9 (CO), 50.0 (α), 40.0 (β), 24.2 (γ), 20.6 (δ), 22.7 (δ′); C1 Pro: 171.9 (CO), 64.0 (α), 26.1 (β), 25.4 (γ), 45.9 (δ); C2 Pro: 171.0 (CO), 61.4 (α), 27.7 (β), 24.8 (γ), 47.4 (δ); C3 Phe: 170.9 (CO), 54.5 (α), 37.0 (β), 138.3 (C-1, Aromatic), 130.0 (C-2, C-6, Aromatic), 128.1 (C-3, C-5, Aromatic), 125.9 (C-4, Aromatic); C4 Gly: 171.1 (CO) and 41.9 (α). [22]
Callyaerin G (**178**)<br>$C_{69}H_{91}N_{13}O_{12}$<br>Cyclic peptide	*Callyspongia aerizusa*<br>Indonesia	^{1}H NMR (600 MHz, DMSO-d_6, δ/ppm, *J*/Hz): Leu^{1}: 4.22 (1H, m, α), 1.62 (2H, m, β), 1.33 (1H, m, γ), 0.53 (3H, d, 6.8, δ), 0.38 (3H, d, 6.8, δ′), 5.32 (1H, d, 7.3, NH); Pro^{2}: 4.41 (1H, dd, 9.8 and 7.9, α), 1.94 (1H, m, β), 1.83 (1H, m, β), 2.15 (2H, m, γ), 3.45 (1H, m, δ), 3.38 (1H, m, δ); Pro^{3}: 4.34 (1H, dd, 11.3 and 7.2, α), 1.80 (1H, m, β), 1.78 (1H, m, β), 2.08 (1H, m, γ), 1.93 (1H, m, γ), 3.86 (1H, m, δ), 3.36 (1H, m, δ); Pro^{4}: 3.92 (1H, m, α), 1.88 (1H, m, β), 1.62 (1H, m, β), 2.12 (1H, m, γ), 2.01 (1H, m, γ), 3.49 (1H, m, δ), 3.40 (1H, m, δ); Pro^{5}: 4.23 (1H, m, α), 1.67 (1H, m, β), 1.46 (1H, m, β), 2.27 (1H, m, γ), 1.78 (1H, m, γ), 3.41 (1H, m, δ), 2.75 (1H, m, δ); Leu^{6}: 4.46 (1H, dt, 10.0 and 3.8, α), 1.92 (2H, m, β), 1.75 (1H, m, γ), 0.86 (3H, d, 6.7, δ), 0.82 (3H, d, 6.7, δ′), 7.51 (1H, d, 9.4, NH); FGly^{7}: 7.46 (1H, d, 13.6, β), 8.23 (1H, s, NH); Pro^{8}: 4.11 (1H, dd, 10.0 and 7.5, α), 1.69 (1H, m, β), 1.06 (1H, m, β), 1.90 (2H, m, γ), 3.83 (1H, m, δ), 3.37 (1H, m, δ); Phe^{9}: 4.25 (1H, m, α), 2.92 (1H, dd, 13.1 and 8.5, β), 2.45 (1H, dd, 13.1 and 8.5, β), 7.67 (1H, d, 8.8, NH), 7.26-7.15 (Aromatic); Gly^{10}: 3.91 (1H, dd, 16.5 and 5.0, α), 3.41 (1H, dd, 16.5 and 5.0, α), 7.96 (1H, t, 5.0, NH); Phe^{11}: 4.63 (1H, dd, 9.5 and 7.2, α), 2.95 (1H, dd, 13.2 and 9.1, β), 2.66 (1H, m, β), 7.21 (1H, d, 7.2, NH), 7.26-7.15 (Aromatic); Phe^{12}: 4.27 (1H, dt, 10.1 and 6.3, α), 2.86 (2H, dd, 12.9 and 10.1, β), 7.71 (1H, d, 6.3, NH), 7.26-7.15 (Aromatic); Terminal-NH_2: 7.11 (1H, br s) and 7.12 (1H, br s). [82]
Callyaerin H (**179**)<br>$C_{54}H_{81}N_{11}O_{10}$<br>Cyclic peptide	*Callyspongia aerizusa*<br>Indonesia	^{1}H NMR (600 MHz, DMSO-d_6, δ/ppm, *J*/Hz): DAA: 8.47 (br s, NH), 7.23 (d, H-3); R1 Val: 5.26 (dd, 13.7 and 10.0, NH), 4.42 (dd, 10.0 and 1.4, α), 2.35 (m, β), 0.68 (d, 6.7, γγ′), 0.89 (d, 6.9, γγ′); R2 Pro: 3.97 (dd, 10.5 and 6.8, α), 2.03 (m, β), 1.82 (m, β), 2.18 (m, γ), 1.82 (m, γ), 3.82 (m, δ), 3.75 (m, δ); R3 Val: 7.59 (d, 5.1, NH), 3.86 (t, 4.7, α), 2.10 (m, β), 0.93 (d, 7.0, γγ′), 0.86 (d, γγ′); R4 Phe: 7.51 (d, 5.8, NH), 4.57 (ddd, 9.6, 5.9 and 3.8, α), 3.03 (dd, 13.1 and 9.7, β), 2.77 (dd, 13.1 and 3.6, β), 7.2-7.3 (m); R5 Pro: 4.34 (dd, 11.4 and 7.5, α), 1.80-2.10 (m, β), 1.80-2.10 (m, γ), 3.63 (m, δ), 3.35 (m, δ); R6 Pro: 4.17 (dd, 9.3 and 7.7, α), 2.21 (m, β), 1.69 (m, β), 1.45 (m, γ), 3.27 (m, δ); R7 Leu: 7.54 (d, 9.6, NH), 4.51 (ddd, 9.8, 9.8 and 4.3, α),

		1.77 (m, β), 1.85 (m, γ), 0.86 (d, δ, δ′), 0.82 (d, δ, δ′); C1 Pro: 4.34 (dd, 9.6 and 7.8, α), 2.16 (m, β), 1.72 (m, β), 1.81 (m, γ), 1.55 (m, γ), 3.48 (br t, 8.0, δ), 3.24 (m, δ); C2 Ile: 7.50 (d, 9.1, NH), 4.01 (dd, 9.2 and 6.1, α), 1.92 (m, β), 1.21 (m, γ), 1.40 (m, γ), 0.85 (d, 6.6, γ′δ), 0.82 (t, 7.3, γ′δ), 6.96 (br s, NH_2) and 6.89 (br s, NH_2). ^{13}C NMR (150 MHz, DMSO-d_6): DAA: 167.2 (s, CO), 98.8 (s, C-2), 143.3 (d, C-3); R1 Val: 172.4 (s, CO), 62.7 (d, α), 29.3 (d, β), 15.6 (q, γγ′), 21.6 (q, γγ′); R2 Pro: 172.4 (s, CO), 59.8 (d, α), 26.8 (t, β), 24.3 (t, γ), 46.2 (d, δ); R3 Val: 170.7 (s, CO), 61.0 (d, α), 28.8 (d, β), 18.4 (q, γγ′), 18.5 (q, γγ′); R4 Phe: 168.9 (s, CO), 53.5 (d, α), 36.2 (t, β), 137.9 (s, C-1), 128.9 (d, C-2, C-6), 128.1 (d, C-3, C-5), 126.5 (d, C-4); R5 Pro: 171.5 (s, CO), 63.2 (d, α), 29.5 (t, β), 24.3 (t, γ), 46.4 (d, δ); R6 Pro: 171.3 (s, CO), 63.6 (d, α), 29.5 (t, β), 25.5 (t, γ), 46.5 (d, δ); R7 Leu: 172.2 (s, CO), 50.4 (d, α), 41.0 (t, β), 24.7 (d, γ), 19.9 (q, δ, δ′), 22.6 (q, δ, δ′); C1 Pro: 171.9 (s, CO), 63.6 (d, α), 29.5 (t, β), 25.6 (t, γ), 48.5 (d, δ); C2 Ile: 173.2 (s, CO), 57.2 (d, α), 35.5 (d, β), 28.6 (t, γ), 15.3 (q, γ′δ) and 11.3 (q, γ′δ). [4]
Callyaerin I (**180**)<br>$C_{69}H_{93}N_{13}O_{12}$<br>Cyclic peptide	*Callyspongia aerizusa*<br>Indonesia	^{1}H NMR (600 MHz, DMSO-d_6, δ/ppm, *J*/Hz): DAA: 8.45 (br s, NH), 7.43 (d, 13.5, β); R1 Leu: 5.36 (dd, 13.5 and 9.8, NH), 4.39 (α), 1.59 (m, β), 1.02 (m, β), 1.21 (m, γ), 0.45 (d, 6.4, δ), 0.32 (d, 6.7, δ′); R2 Pro: 3.98 (dd, 10.0 and 7.3, α), 2.14 (m, β), 1.63 (m, β), 2.02 (m, γ), 1.82 (m, γ), 3.96 (m, δ), 3.48 (m, δ); R3 Phe: 7.61 (d, 6.8, NH), 4.38 (α), 2.96 (m, β), 7.25 (H-2, H-6, Aromatic), 7.15 (H-3, H-5, Aromatic), 7.18 (H-4, Aromatic); R4 Phe: 7.25 (NH), 4.63 (α), 2.96 (m, β), 2.68 (dd, 13.4 and 4.7, β), 7.25 (H-2, H-6, Aromatic), 7.29 (H-3, H-5, Aromatic), 7.23 (H-4, Aromatic); R5 Pro: 4.44 (dd, 11.1 and 7.6, α), 2.16 (m, β), 1.81 (m, β), 1.92 (m, γ), 1.90 (m, γ), 3.49 (m, δ), 3.41 (m, δ); R6 Pro: 4.18 (dd, 10.1 and 7.4, α), 2.23 (m, β), 1.42 (m, β), 1.75 (m, γ), 1.59 (m, γ), 3.34 (m, δ), 2.49 (m, δ); R7 Val: 7.03 (d, 10.1, NH), 4.62 (dd, 10.2 and 3.8, α), 2.48 (m, β), 1.19 (d, 7.0, γ), 1.06 (d, 7.0, γ′); C1 Pro: 4.30 (dd, 10.8 and 7.1, α), 2.30 (dt, 12.1 and 6.4, β), 1.52 (m, β), 1.71 (m, γ), 1.85 (m, γ), 3.64 (dd, 11.0 and 7.7, δ), 3.24 (m, δ); C2 Leu: 7.78 (d, 7.0, NH), 3.82 (ddd, 11.4, 7.0 and 4.2, α), 1.66 (m, β), 1.12 (ddd, 13.6, 9.3 and 4.2, β), 1.56 (m, γ), 0.87 (d, 6.6, δ), 0.74 (d, 6.6, δ′); C3 Phe: 7.66 (d, 9.7, NH), 4.23 (ddd, 12.4, 9.7 and 2.9, α), 3.24 (dd, 12.4 and 2.9, β), 2.75 (t, 12.4, β), 7.25 (H-2, H-6, Aromatic), 7.12 (H-3, H-5, Aromatic), 7.15 (H-4, Aromatic); C4 Gly: 7.53 (dd, 7.3 and 5.0, NH), 3.88 (dd, 17.0 and 7.3, α), 3.52 (dd, 17.0 and 5.0, α), 7.21 (br s, NH_2) and 7.10 (br s, NH_2). ^{13}C NMR (150 MHz, DMSO-d_6): DAA: 168.3 (CO), 98.1 (α), 143.3 (β); R1 Leu: 173.4 (CO), 57.4 (α), 42.1 (β), 23.1 (γ), 19.6 (δ), 22.7 (δ′); R2 Pro: 172.1 (CO), 63.1 (α), 28.3 (β), 24.7 (γ), 45.9 (δ); R3 Phe: 170.9 (CO), 53.7 (α), 35.0 (β), 138.0 (C-1, Aromatic), 128.3 (C-2, C-3, C-5, C-6, Aromatic), 126.3 (C-4, Aromatic); R4 Phe: 169.1 (CO),

		52.7 (α), 35.8 (β), 137.8 (C-1, Aromatic), 128.9 (C-2, C-6, Aromatic), 128.1 (C-3, C-5, Aromatic), 126.4 (C-4, Aromatic); R5 Pro: 171.6 (CO), 63.4 (α), 26.9 (β), 24.0 (γ), 46.1 (δ); R6 Pro: 171.7 (CO), 63.1 (α), 28.7 (β), 25.4 (γ), 46.5 (δ); R7 Val: 171.8 (CO), 56.6 (α), 30.0 (β), 17.4 (γ), 18.0 (γ′); C1 Pro: 174.1 (CO), 61.9 (α), 29.2 (β), 25.7 (γ), 48.5 (δ); C2 Leu: 171.5 (CO), 52.6 (α), 38.0 (β), 24.4 (γ), 22.3 (δ), 20.4 (δ′); C3 Phe: 170.7 (CO), 54.4 (α), 37.5 (β), 138.2 (C-1, Aromatic), 129.7 (C-2, C-6, Aromatic), 127.6 (C-3, C-5, Aromatic), 125.9 (C-4, Aromatic); C4 Gly: 170.6 (CO) and 42.1 (α). [22]
Callyaerin J (**181**) $C_{66}H_{95}N_{13}O_{12}$ Cyclic peptide	*Callyspongia aerizusa* Indonesia	^{1}H NMR (600 MHz, DMSO-d_6, δ/ppm, *J*/Hz): DAA: 8.21 (br s, NH), 7.04 (d, 13.4, β); R1 Phe: 5.44 (dd, 13.4 and 9.7, NH), 4.60 (α), 3.34 (dd, 14.2 and 3.1, β), 2.67 (m, β), 7.21 (H-2, H-6, Aromatic), 7.22 (H-3, H-4, H-5, Aromatic); R2 Pro: 4.02 (dd, 10.5 and 6.9, α), 2.23 (m, β), 1.81 (m, β), 2.10 (m, γ), 1.90 (m, γ), 3.97 (br t, 9.7, δ), 3.85 (td, 9.7 and 6.5, δ); R3 Leu: 7.41 (d, 6.2, NH), 4.08 (α), 1.47 (m, β), 1.63 (m, γ), 0.92 (d, 6.6, δ), 0.82 (d, 6.6, δ′); R4 Phe: 7.13 (d, 6.5, NH), 4.60 (α), 3.01 (dd, 13.5 and 8.9, β), 2.64 (dd, 13.5 and 4.9, β), 7.24 (H-2, H-6, Aromatic), 7.26 (H-3, H-5, Aromatic), 7.20 (H-4, Aromatic); R5 Pro: 4.36 (dd, 11.3 and 7.6, α), 2.11 (m, β), 1.80 (m, β), 1.92 (m, γ), 1.90 (m, γ), 3.52 (m, δ), 3.34 (m, δ); R6 Pro: 4.15 (dd, 9.8 and 7.5, α), 2.22 (m, β), 1.42 (m, β), 1.74 (m, γ), 1.58 (m, γ), 3.34 (m, δ), 2.51 (m, δ); R7 Val: 7.10 (d, 10.2, NH), 4.50 (dd, 10.2 and 5.2, α), 2.33 (m, β), 1.06 (d, 6.9, γ), 1.01 (d, 6.9, γ′); C1 Pro: 4.30 (dd, 9.7 and 7.4, α), 2.18 (m, β), 1.54 (m, β), 1.76 (m, γ), 1.65 (m, γ), 3.53 (m, δ), 3.14 (td, 11.3 and 6.7, δ); C2 Ile: 7.68 (d, 7.5, NH), 3.95 (t, 7.5, α), 1.92 (m, β), 1.46 (m, γ), 1.26 (m, γ), 0.86 (d, 6.8, γ′), 0.86 (t, 7.4, δ); C3 Ile: 7.31 (d, 8.4, NH), 4.08 (α), 1.78 (m, β), 1.23 (m, γ), 1.06 (m, γ), 0.69 (d, 6.9, γ′), 0.65 (t, 7.4, δ); C4 Gly: 7.83 (dd, 6.5 and 5.6, NH), 3.70 (dd, 16.8 and 6.5, α), 3.50 (dd, 16.8 and 5.6, α), 7.04 (br s, NH_2) and 7.01 (br s, NH_2). ^{13}C NMR (150 MHz, DMSO-d_6): DAA: 167.3 (CO), 99.2 (α), 142.0 (β); R1 Phe: 172.4 (CO), 60.4 (α), 38.7 (β), 136.0 (C-1, Aromatic), 129.1 (C-2, C-6, Aromatic), 128.2 (C-3, C-5, Aromatic), 126.5 (C-4, Aromatic); R2 Pro: 172.0 (CO), 63.4 (α), 28.8 (β), 24.8 (γ), 46.5 (δ); R3 Leu: 171.9 (CO), 51.8 (α), 38.9 (β), 24.2 (γ), 22.4 (δ), 21.0 (δ′); R4 Phe: 169.1 (CO), 52.5 (α), 36.0 (β), 138.0 (C-1, Aromatic), 128.9 (C-2, C-6, Aromatic), 127.8 (C-3, C-5, Aromatic), 126.2 (C-4, Aromatic); R5 Pro: 171.4 (CO), 63.2 (α), 26.8 (β), 24.1 (γ), 46.3 (δ); R6 Pro: 171.5 (CO), 62.8 (α), 28.6 (β), 25.2 (γ), 46.4 (δ); R7 Val: 170.9 (CO), 56.7 (α), 29.7 (β), 18.7 (γ), 18.3 (γ′); C1 Pro: 172.9 (CO), 61.1 (α), 29.0 (β), 25.5 (γ), 48.4 (δ); C2 Ile: 171.1 (CO), 58.5 (α), 35.1 (β), 24.9 (γ), 15.4 (γ′), 11.0 (δ); C3 Ile: 170.9 (CO), 57.1 (α), 35.4 (β), 24.1 (γ), 15.3 (γ′), 10.5 (δ); C4 Gly: 170.9 (CO) and 42.1 (α). [22]

Callyaerin K (**182**)<br>$C_{77}H_{97}N_{13}O_{16}$<br>Cyclic peptide	*Callyspongia aerizusa*<br>Indonesia	^{1}H NMR (600 MHz, DMSO-d_6, δ/ppm, *J*/Hz): DAA: 8.93 (br s, NH), 7.41 (d, 13.6, β); R1 Phe: 5.53 (dd, 13.6 and 8.5, NH), 4.54 (m, α), 2.87 (m, β), 2.68 (m, β), 7.05 (H-2, H-6, Aromatic), 7.24 (H-3, H-5, Aromatic), 7.21 (H-4, Aromatic); R2 Pro: 3.89 (br d, 7.8, α), 1.44 (m, β), 1.30 (m, β), 1.63 (m, γ), 1.52 (m, γ), 3.43 (m, δ), 3.08 (ddd, 11.4, 8.9 and 7.4, δ); R3 Phe: 8.61 (br s, NH), 3.97 (dd, 10.9 and 5.4, α), 3.30 (m, β), 2.84 (m, β), 7.38 (H-2, H-6, Aromatic), 7.28 (H-3, H-5, Aromatic), 7.21 (H-4, Aromatic); R4 Gly: 8.83 (br s, NH), 3.50 (m, α), 3.24 (m, α); R5 Leu: 7.05 (NH), 4.04 (m, α), 1.58 (m, β), 1.30 (m, β), 1.93 (m, γ), 0.87 (d, 6.5, δ), 0.85 (d, 6.5, δ′); R6 Pro: 4.03 (m, α), 2.15 (m, β), 1.76 (m, β), 1.90 (m, γ), 1.78 (m, γ), 4.02 (m, δ), 3.45 (m, δ); R7 Pro: 4.18 (α), 2.02 (m, β), 1.10 (m, β), 1.44 (m, γ), 1.32 (m, γ), 3.66 (m, δ), 3.15 (m, δ); R8 Phe: 9.23 (br d, 8.1, NH), 5.11 (ddd, 11.8, 8.1 and 3.2, α), 3.27 (β), 3.15 (br d, 11.8, β), 7.11 (H-2, H-6, Aromatic), 7.25 (H-3, H-5, Aromatic), 7.22 (H-4, Aromatic); C1 Pro: 4.07 (dd, 10.1 and 7.7, α), 1.83 (m, β), 0.57 (m, β), 1.61 (m, γ), 1.35 (m, γ), 2.95 (m, δ); C2 Phe: 7.83 (d, 9.3, NH), 4.32 (ddd, 12.6, 9.3 and 3.6, α), 3.33 (m, β), 2.87 (m, β), 7.32 (H-2, H-6, Aromatic), 7.25 (H-3, H-5, Aromatic), 7.19 (H-4, Aromatic); C3 Ile: 7.21 (NH), 4.19 (α), 1.84 (m, β), 1.43 (m, γ), 1.32 (m, γ), 0.82 (d, 6.8, γ′), 0.71 (t, 7.4, δ); C4 Asp: 8.26 (d, 7.7, NH), 4.52 (ddd, 7.7, 6.8 and 6.3, α), 2.70 (dd, 6.3 and 16.6, β) and 2.55 (dd, 6.8 and 16.6, β). ^{13}C NMR (150 MHz, DMSO-d_6): DAA: 168.1 (CO), 101.1 (α), 139.1 (β); R1 Phe: 169.0 (CO), 58.2 (α), 42.8 (β), 135.6 (C-1, Aromatic), 129.2 (C-2, C-6, Aromatic), 127.9 (C-3, C-5, Aromatic), 126.4 (C-4, Aromatic); R2 Pro: 171.9 (CO), 58.9 (α), 31.2 (β), 21.0 (γ), 45.1 (δ); R3 Phe: 170.4 (CO), 55.3 (α), 34.4 (β), 139.0 (C-1, Aromatic), 129.0 (C-2, C-6, Aromatic), 128.1 (C-3, C-5, Aromatic), 126.1 (C-4, Aromatic); R4 Gly: 169.5 (CO) and 42.6 (α); R5 Leu: 170.8 (CO), 49.9 (α), 38.9 (β), 23.1 (γ), 22.6 (δ), 22.0 (δ′); R6 Pro: 169.6 (CO), 58.1 (α), 27.5 (β), 24.1 (γ), 47.3 (δ); R7 Pro: 173.3 (CO), 60.3 (α), 31.6 (β), 21.3 (γ), 47.1 (δ); R8 Phe: 171.7 (CO), 55.9 (α), 37.3 (β), 138.1 (C-1, Aromatic), 128.6 (C-2, C-6, Aromatic), 128.0 (C-3, C-5, Aromatic), 126.3 (C-4, Aromatic); C1 Pro: 171.6 (CO), 60.3 (α), 28.5 (β), 24.6 (γ), 48.1 (δ); C2 Phe: 170.2 (CO), 53.7 (α), 36.9 (β), 138.4 (C-1, Aromatic), 129.4 (C-2, C-6, Aromatic), 128.2 (C-3, C-5, Aromatic), 126.0 (C-4, Aromatic); C3 Ile: 170.2 (CO), 55.8 (α), 35.9 (β), 24.1 (γ), 14.9 (γ′), 10.1 (δ); C4 Asp: 172.1 (CO), 171.9 (CO(γ)), 48.3 (α) and 35.7 (β). [22]
Callyaerin L (**183**)<br>$C_{66}H_{101}N_{13}O_{15}$<br>Cyclic peptide	*Callyspongia aerizusa*<br>Indonesia	^{1}H NMR (700 MHz, CF_3CD_2OH/H_2O 1:1, δ/ppm, *J*/Hz): DAA: 8.19 (br s, NH), 7.24 (d, 13.4, β); R1 Ile: 5.50 (dd, 13.4 and 10.3, NH), 3.73 (t, 10.3, α), 1.50 (m, β), 1.27 (m, γ), 0.62 (m, γ), 0.81 (d, 6.5, γ′), 0.26 (t, 7.4, δ); R2 Hyp: 4.23 (α), 2.35 (m, β), 2.05 (m, β), 4.62 (br s, γ), 4.01 (m, δ), 3.77 (dd, 11.2 and 1.5, δ); R3 Glu: 8.92 (br s, NH), 3.90 (α), 2.12 (m, β), 2.17 (m, β), 2.35 (m, γ), 2.27 (m, γ); R4 Ile: 7.70

		(br s, NH), 4.24 (α), 1.45 (m, β), 1.43 (m, γ), 1.05 (m, γ), 0.90 (d, 6.6, γ′), 0.78 (t, 7.3, δ); R5 Val: 7.75 (d, 7.8, NH), 4.58 (α), 2.21 (m, β), 1.05 (d, 6.8, γ), 1.01 (d, 6.8, γ′); R6 Pro: 4.05 (α), 2.44 (m, β), 1.99 (m, β), 2.23 (m, γ), 2.20 (m, γ), 3.57 (m, δ), 3.52 (m, δ); R7 Pro: 4.37 (α), 2.37 (m, β), 1.74 (m, β), 2.00 (m, γ), 1.96 (m, γ), 3.60 (m, δ), 3.34 (m, δ); R8 Leu: 7.00 (d, 9.8, NH), 4.75 (ddd, 11.7, 9.8 and 3.8, α), 1.95 (m, β), 1.78 (m, β), 1.62 (m, γ), 0.94 (d, 6.5, δ), 0.87 (d, 6.5, δ′); C1 Pro: 4.36 (α), 2.38 (m, β), 1.60 (m, β), 2.02 (m, γ), 1.85 (dq, 12.2 and 5.7, γ), 3.54 (m, δ), 3.35 (m, δ); C2 Leu: 8.11 (d, 7.1, NH), 4.05 (α), 1.60 (m, β), 1.11 (m, β), 1.56 (m, γ), 0.85 (d, 6.5, δ), 0.73 (d, 6.5, δ′); C3 Phe: 7.81 (d, 9.1, NH), 4.40 (ddd, 12.0, 9.1 and 3.1, α), 3.28 (dd, 13.7 and 3.1, β), 2.73 (dd, 13.7 and 12.0, β), 7.21 (H-2, H-6, Aromatic), 7.13 (H-3, H-4, H-5, Aromatic), 7.27 (br s, NH_2) and 6.94 (br s, NH_2). ^{13}C NMR (176 MHz, CF_3CD_2OH/H_2O 1:1): DAA: 171.4 (CO), 102.3 (α), 145.4 (β); R1 Ile: 175.3 (CO), 68.8 (α), 40.9 (β), 26.8 (γ), 16.9 (γ′), 11.7 (δ); R2 Hyp: 175.8 (CO), 63.5 (α), 39.4 (β), 71.8 (γ), 58.6 (δ); R3 Glu: 175.5 (CO), 182.0 (CO(δ)), 59.3 (α), 28.3 (β), 35.1 (γ); R4 Ile: 174.8 (CO), 63.1 (α), 40.8 (β), 26.9 (γ), 16.3 (γ′), 11.1 (δ); R5 Val: 173.7 (CO), 57.9 (α), 32.5 (β), 21.1 (γ), 19.9 (γ′); R6 Pro: 176.0 (CO), 66.8 (α), 28.4 (β), 26.9 (γ), 49.1 (δ); R7 Pro: 176.1 (CO), 65.4 (α), 30.8 (β), 27.4 (γ), 50.1 (δ); R8 Leu: 175.2 (CO), 53.7 (α), 43.2 (β), 26.9 (γ), 24.1 (δ), 21.6 (δ′); C1 Pro: 178.4 (CO), 65.1 (α), 31.6 (β), 28.0 (γ), 52.0 (δ); C2 Leu: 177.4 (CO), 55.7 (α), 41.1 (β), 26.7 (γ), 24.2 (δ), 21.7 (δ′); C3 Phe: 178.6 (CO), 57.9 (α), 40.0 (β), 139.1 (C-1, Aromatic), 131.7 (C-2, C-6, Aromatic), 130.5 (C-3, C-5, Aromatic) and 128.9 (C-4, Aromatic). [22]
Callyaerin M (**184**)<br>$C_{64}H_{95}N_{15}O_{15}$<br>Cyclic peptide	*Callyspongia aerizusa*<br>Indonesia	^{1}H NMR (600 MHz, DMSO-d_6, δ/ppm, *J*/Hz): DAA: 8.40 (br s, NH), 7.40 (d, 13.7, β); R1 AMOIPA: 5.76 (dd, 13.7 and 8.8, NH), 5.13 (dd, 10.8 and 8.8, α), 3.83 (dd, 10.8 and 1.3, β), 3.59 (br dd, 1.7 and 1.3, H-2), 2.09 (s, OCH_3), 7.60 (br t, 1.7, $NH_{(3)}$), 9.64 (br d, 1.7, $NH_{(5)}$); R2 Hyp: 4.14 (α), 2.04 (m, β), 1.92 (m, β), 4.32 (m, γ), 3.50 (m, δ), 3.40 (dd, 10.8 and 3.9, δ), 5.20 (d, 4.2, OH); R3 Leu: 6.71 (d, 6.0, NH), 3.96 (dt, 9.0 and 6.0, α), 1.48 (m, β), 1.47 (m, β), 1.60 (m, γ), 0.86 (d, 6.6, δ), 0.80 (d, 6.6, δ′); R4 Leu: 7.11 (d, 7.1, NH), 4.53 (q, 7.1, α), 1.63 (m, β), 1.37 (m, β), 1.47 (m, γ), 0.93 (d, 6.6, δ), 0.91 (d, 6.6, δ′); R5 Pro: 4.45 (dd, 11.0 and 7.6, α), 2.20 (m, β), 1.91 (m, β), 2.00 (m, γ), 3.58 (m, δ), 3.51 (m, δ); R6 Pro: 4.29 (t, 8.3, α), 2.30 (m, β), 1.62 (m, β), 1.92 (m, γ), 3.64 (m, δ), 3.26 (m, δ); R7 Val: 7.34 (d, 10.2, NH), 4.53 (dd, 10.2 and 5.3, α), 2.46 (m, β), 1.08 (d, 6.9, γ), 0.98 (d, 6.9, γ′); C1 Pro: 4.30 (α), 2.30 (m, β), 1.53 (m, β), 1.88 (m, γ), 1.76 (m, γ), 3.48 (m, δ), 3.27 (m, δ); C2 Leu: 7.96 (d, 6.8, NH), 3.77 (m, α), 1.51 (m, β), 1.08 (m, β), 1.59 (m, γ), 0.82 (d, 6.6, δ), 0.71 (d, 6.6, δ′); C3 Phe: 7.64 (d, 9.2, NH), 4.14 (ddd, 13.1, 9.2 and 3.0, α), 3.19 (dd, 13.1 and 3.0, β), 2.81 (t, 13.1, β), 7.15 (H-2, H-6,

		Aromatic), 7.04 (H-3, H-5, Aromatic), 7.07 (H-4, Aromatic); C4 Gly: 7.46 (dd, 6.9 and 5.5, NH), 3.81 (dd, 17.0 and 6.9, α), 3.55 (dd, 17.0 and 5.5, α), 7.17 (br s, NH_2) and 7.06 (br s, NH_2). ^{13}C NMR (150 MHz, DMSO-d_6): DAA: 167.8 (CO), 99.6 (α), 141.0 (β); R1 AMOIPA: 171.0 (CO), 56.5 (α), 94.1 (β), 140.1 (C-1), 83.5 (C-2), 157.5 (C-4), 52.0 ($OCH_{(3)}$); R2 Hyp: 171.6 (CO), 61.3 (α), 36.9 (β), 68.7 (γ), 54.8 (δ); R3 Leu: 171.6 (CO), 52.6 (α), 39.2 (β), 24.1 (γ), 22.4 (δ), 21.3 (δ'); R4 Leu: 170.2 (CO), 48.5 (α), 40.2 (β), 23.9 (γ), 22.7 (δ), 21.9 (δ'); R5 Pro: 171.7 (CO), 63.8 (α), 26.4 (β), 24.2 (γ), 45.8 (δ); R6 Pro: 171.6 (CO), 62.5 (α), 28.8 (β), 25.3 (γ), 46.8 (δ); R7 Val: 171.4 (CO), 56.7 (α), 29.2 (β), 17.9 (γ), 18.9 (γ'); C1 Pro: 174.2 (CO), 62.1 (α), 29.0 (β), 25.6 (γ), 48.3 (δ); C2 Leu: 172.0 (CO), 52.7 (α), 37.8 (β), 24.1 (γ), 22.5 (δ), 20.5 (δ'); C3 Phe: 170.9 (CO), 54.8 (α), 36.4 (β), 138.2 (C-1, Aromatic), 129.6 (C-2, C-6, Aromatic), 127.2 (C-3, C-5, Aromatic), 125.3 (C-4, Aromatic); C4 Gly: 170.5 (CO) and 42.0 (α). [22]
Callynormine A (**185**) $C_{61}H_{93}N_{11}O_{13}$ Cyclic peptide	*Callyspongia abnormis* Kenyan	^{1}H NMR (500 MHz, CD_3OD, δ/ppm, *J*/Hz): Ile^{1}: 4.37 (m, H-2), 1.98 (m, H-3), 1.08 (d, 6.8, H-3-Me), 1.11 (m, H-4), 1.31 (m, H-4), 0.92 (t, 7.9, H-4-Me), 5.58 (m, NH); Hyp^{2}: 4.40 (m, H-2), 2.08 (m, H-3), 2.31 (m, H-3), 4.57 (m, H-4), 3.72 (d, 11.1, H-5), 4.01 (dd, 3.6 and 11.3, H-5); Val^{3}: 4.17 (d, 4.2, H-2), 2.28 (m, H-3), 1.05 (d, 7.3, H-3-Me), 7.06 (d, 5.5, NH); Leu^{4}: 4.76 (m, H-2), 1.56 (m, H-3), 1.69 (m, H-3), 1.69 (m, H-4), 1.05 (d, 6.2, H-4-Me), 0.99 (d, 5.9, H-4-Me), 7.53 (d, 7.3, NH); Pro^{5}: 4.46 (m, H-2), 1.87 (m, H-3), 2.50 (m, H-3), 2.08 (m, H-4), 3.39 (m, H-5), 3.77 (m, H-5); Pro^{6}: 4.26 (dd, 10.0 and 7.5, H-2), 2.08 (m, H-3), 1.69 (m, H-4), 1.83 (m, H-4), 3.41 (m, H-5), 3.65 (m, H-5); Leu^{7}: 4.96 (dt, 3.7 and 9.8, H-2), 1.90 (m, H-3), 2.00 (m, H-3), 1.83 (m, H-4), 0.98 (d, 6.7, H-4-Me), 1.05 (d, 6.6, H-4-Me), 7.84 (d, 9.5, NH); FGly^{8}: 7.17 (d, 5.0, H-3), 8.64 (s, NH); Pro^{9}: 4.57 (m, H-2), 2.08 (m, H-3), 2.28 (m, H-3), 2.18 (m, H-4), 2.24 (m, H-4), 3.61 (m, H-5), 3.86 (t, 8.9, H-5); Phe^{10}: 4.61 (dt, 4.3 and 12.2, H-2), 3.02 (t, 12.9, H-3), 3.33 (m, H-3), 7.33 (d, 7.4, H-5, H-9), 7.40 (t, 7.6, H-6, H-8), 7.27 (t, 7.27, H-7), 7.73 (m, NH); Leu^{11}: 4.46 (m, H-2), 1.63 (m, H-3), 1.83 (m, H-4), 0.97 (d, 6.7, H-4-Me), 0.96 (d, 6.8, H-4-Me) and 7.73 (m, NH). ^{13}C NMR (125 MHz, CD_3OD): Ile^{1}: 175.4 (CO), 66.4 (C-2), 39.2 (C-3), 17.6 (C-3-Me), 24.3 (C-4), 12.3 (C-4-Me); Hyp^{2}: 174.9 (CO), 64.2 (C-2), 28.5 (C-3), 71.3 (C-4), 57.1 (C-5); Val^{3}: 173.6 (CO), 62.1 (C-2), 30.9 (C-3), 19.1 (C-3-Me), 19.6 (C-3-Me); Leu^{4}: 172.6 (CO), 51.2 (C-2), 43.2 (C-3), 26.2 (C-4), 23.5 (C-4-Me); Pro^{5}: 175.0 (CO), 64.7 (C-2), 30.4 (C-3), 27.3 (C-4), 49.2 (C-5); Pro^{6}: 175.0 (CO), 63.5 (C-2), 38.9 (C-3), 26.5 (C-4), 51.1 (C-5); Leu^{7}: 176.3 (CO), 53.1 (C-2), 42.6 (C-3), 26.8 (C-4), 21.9 (C-4-Me), 24.0 (C-4-Me); FGly^{8}: 170.5 (CO), 100.5 (C-2), 143.8 (C-3); Pro^{9}: 174.7 (CO), 66.3 (C-2), 30.8 (C-3), 26.5 (C-4), 48.2 (C-5); Phe^{10}: 174.6 (CO), 56.7 (C-2),

		38.9 (C-3), 138.8 (C-4), 130.6 (C-5, C-9), 130.0 (C-6, C-8), 128.3 (C-7); Leu^{11}: 178.2 (CO), 53.6 (C-2), 43.2 (C-3), 26.3 (C-4), 21.4 (C-4-Me), 24.8 (C-4-Me). [83]
Callyptide A (**186**)<br>$C_{52}H_{72}N_8O_{12}$<br>Cyclic peptide	*Callyspongia* sp.<br>Saudi Arabia	^{1}H NMR (500 MHz, DMSO-*d*$_6$, δ/ppm, *J*/Hz): Thr^1: 4.32 (1H, dd, 10.1 and 6.9, H-2), 3.71 (1H, dd, 10.1 and 6.8, H-3), 0.88 (3H, d, 6.8, $H_{3\text{-}4}$), 8.33 (1H, d, 6.9, NH); Ala^2: 4.29 (1H, dq, 7.9 and 7.1, H-6), 1.16 (3H, d, 7.1, $H_{3\text{-}7}$), 7.23 (1H, d, 7.9, NH); Leu^3: 3.85 (1H, dd, 7.9 and 6.9, H-9), 1.51 (2H, m, H-10), 1.47 (1H, m, H-11), 0.87 (3H, d, 6.7 Hz, $H_{3\text{-}12}$), 0.87 (3H, d, 6.7, $H_{3\text{-}13}$), 7.38 (1H, d, 7.9, NH); Tyr^4: 4.58 (1H, dt, 8.6 and 6.5, H-15), 2.86 (1H, dd, 13.5 and 6.5, H-16A), 2.81 (1H, m, H-16-B), 7.07 (2H, d, 7.9, H-18, H-22), 6.61 (2H, d, 7.9, H-19, H-21), 7.33 (1H, d, 8.6, NH); Phe^5: 4.52 (1H, dd, 7.6 and 6.8, H-24), 2.94 (1H, dd, 13.7 and 6.8, H-25A), 2.92 (1H, m, H-25B), 7.19 (5H, m, H-27, H-31), 7.10 (2H, m, H-28, H-30), 7.14 (1H, m, H-29), 8.43 (1H, d, 7.6, NH); Ile^6: 4.32 (1H, dd, 7.1 and 6.9, H-33), 1.83 (1H, m, H-34), 1.65 (1H, m, H-35A), 1.35 (1H, m, H-35B), 0.79 (3H, t, 7.1, $H_{3\text{-}36}$), 0.85 (3H, d, 6.9, $H_{3\text{-}37}$), 8.35 (1H, d, 6.9, NH); 3-OHLeu^7: 4.50 (1H, dd, 10.4 and 7.8, H-39), 4.75 (1H, dd, 10.4 and 7.1, H-40), 1.72 (1H, m, H-41), 0.84 (3H, d, 6.9, $H_{3\text{-}42}$), 0.84 (3H, d, 6.9, $H_{3\text{-}43}$), 8.41 (1H, d, 7.8, NH); Tyr^9: 4.58 (1H, dt, 8.6 and 6.5, H-45), 2.86 (1H, dd, 13.5 and 6.5, H-46A), 2.81 (1H, m, H-46B), 7.04 (2H, d, 7.9, H-48, H-52), 6.60 (2H, d, 7.9, H-49, H-51) and 7.33 (1H, d, 8.6, NH). ^{13}C NMR (125 MHz, DMSO-*d*$_6$): Thr^1: 174.2 (C-1), 50.7 (C-2), 71.8 (C-3), 21.4 (C-4); Ala^2: 172.5 (C-5), 47.8 (C-6), 18.0 (C-7); Leu^3: 171.1 (C-8), 53.1 (C-9), 42.7 (C-10), 27.2 (C-11), 22.6 (C-12, C-13); Tyr^4: 170.4 (C-14), 55.2 (C-15), 36.3 (C-16), 128.3 (C-17), 130.1 (C-18, C-22), 114.8 (C-19, C-21), 157.4 (C-20); Phe^5: 166.4 (C-23), 55.5 (C-24), 36.5 (C-25), 135.4 (C-26), 129.1 (C-27, C-31), 128.9 (C-28, C-30), 127.9 (C-29); Ile^6: 171.6 (C-32), 50.7 (C-33), 39.3 (C-34), 24.1 (C-35), 9.2 (C-36), 21.4 (C-37); 3-OHLeu^7: 176.3 (C-38), 55.6 (C-39), 74.5 (C-40), 28.8 (C-41), 23.2 (C-42), 23.2 (C-43); Tyr^9: 170.7 (C-44), 55.2 (C-45), 36.3 (C-46), 128.0 (C-47), 130.1 (C-48, C-52), 114.8 (C-49, C-51) and 157.6 (C-50). [84]
Phoriospongin A (**187**)<br>$C_{52}H_{82}ClN_{11}O_{15}$<br>Depsipeptide	*Callyspongia bilamellata*<br>Australia	^{1}H NMR (400 MHz, CD_3OD, δ/ppm, *J*/Hz): 4.3 (ddd, 11.6, 8.2 and 3.7, H-2), 1.83 (m, H-3a), 1.64 (m, H-3b), 1.48 (m, H-4a), 1.31 (m, H-4b), 0.93 (t, 7.3, H-5), 7.94 (d, 8.2, 2-NH), 3.87 (dd, 16.5 and 4.0, H-7a), 3.67 (dd, 16.5 and 4.0, H-7b), 8.86 (t, 4.0, 7-NH), 5.12 (ddd, 13.1, 10.1 and 7.0, H-9), 3.07 (m, H-10a), 3.03 (dd, 13.1 and 10.1, H-10b), 7.27 (m, H-12, H-16), 7.31 (m, H-13, H-15), 7.26 (m, H-14), 7.98 (d, 7.0, 9-NH), 4.93 (dd, 9.5 and 6.7, H-18), 3.99 (dd, 6.7 and 6.4, H-19), 1.18 (d, 6.4, H-20), 7.2 (d, 9.5, 18-NH), 4.95 (m, H-22), 2.74 (dd, 14.3 and 9.2, H-23a), 2.65 (dd, 14.3 and 4.9, H-23b), 8.58 (d, 10.1, 22-NH), 4.45 (dd, 11 and 8.5, H-26), 2.58 (m, H-27), 4.53 (dq, 6.7 and 1.2, H-28), 1.51 (d, 6.7, H-29), 0.98 (d, 6.4, H-30), 7.85

		(d, 8.5, 26-NH), 3.54 (dd, 9.5 and 4.0, H-32), 1.92 (m, H-33a), 1.58 (m, H-33b), 1.04 (m, H-34a), 0.77 (m, H-34b), 0.82 (t, 6.4, H-35), 3.06 (s, H-52), 4.07 (dd, 7.6 and 6.1, H-37), 1.11 (d, 7.6, H-38), 7.36 (d, 6.1, 37-NH), 2.56 (m, H-40a), 2.21 (m, H-40b), 4.47 (m, H-41), 4.01 (d, 9.8, H-42), 5.32 (dq, 6.4 and 3.7, H-43), 1.28 (d, 3.7, H-44), 8.54 (d, 9.8, 42-NH), 4.615 (m, H-46), 1.75 (m, H-47a), 1.65 (m, H-47b), 1.82 (m, H-48), 1.07 (d, 6.4, H-49), 1.02 (d, 6.4, H-50), 8.41 (d, 6.7, 46-NH) and 8.15 (s, H-51). ^{13}C NMR (100 MHz, CD_3OD): 173.4 (C-1), 54.74 (C-2), 34.09 (C-3a), 20.52 (C-4a), 13.51 (C-5), 172.36 (C-6), 45.19 (C-7a), 175.67 (C-8), 51.99 (C-9), 39.23 (C-10a), 136.94 (C-11), 130.49 (C-12, C-16), 129.61 (C-13, C-15), 128.15 (C-14), 172.36 (C-17), 58.75 (C-18), 69.33 (C-19), 19.56 (C-20), 174.24 (C-21), 50.71 (C-22), 31.68 (C-23a), 173.49 (C-25), 56.66 (C-26), 42.11 (C-27), 59.04 (C-28), 23.19 (C-29), 10.02 (C-30), 174.6 (C-31), 66.83 (C-32), 31.68 (C-33a), 20.69 (C-34a), 14.3 (C-35), 39.88 (C-52), 174.24 (C-36), 51.32 (C-37), 17.66 (C-38), 172.36 (C-39), 40.89 (C-40a), 72.27 (C-41), 56.54 (C-42), 75.49 (C-43), 19.17 (C-44), 175.23 (C-45), 52.63 (C-46), 42.75 (C-47a), 26.00 (C-48), 23.19 (C-49), 22.53 (C-50) and 163.71 (C-51). [85]
Phoriospongin B (**188**) $C_{53}H_{84}ClN_{11}O_{15}$ Depsipeptide	*Callyspongia bilamellata* Australia	^{1}H NMR (400 MHz, CD_3OD, δ/ppm, *J*/Hz): 4.29 (ddd, 11.6, 7.9 and 3.6, H-2), 1.84 (m, H-3a), 1.7 (m, H-3b), 1.46 (m, H-4a), 1.32 (m, H-4b), 0.93 (t, 7.3, H-5), 7.92 (d, 7.9, 2-NH), 3.86 (dd, 16.8 and 3.7, H-7a), 3.66 (dd, 16.8 and 3.7, H-7b), 8.82 (t, 3.7, 7-NH), 5.12 (ddd, 12.8, 10.1 and 6.7, H-9), 3.08 (m, H-10a), 3.02 (m, H-10b), 7.24 (m, H-12, H-16), 7.27 (m, H-13, H-15), 7.25 (m, H-14), 8 (d, 6.7, 9-NH), 4.93 (m, H-18), 4.01 (dd, 6.7 and 6.4, H-19), 1.19 (d, 6.4, H-20), 7.2 (d, 9.2, 18-NH), 4.99 (m, H-22), 2.75 (dd, 14.3 and 9.2, H-23a), 2.65 (dd, 14.3 and 5.2, H-23b), 8.55 (d, 10.1, 22-NH), 4.45 (dd, 11.0 and 8.9, H-26), 2.56 (m, H-27), 4.52 (dq, 6.7 and 1.2, H-28), 1.51 (d, 6.7, H-29), 0.98 (d, 6.4, H-30), 7.81 (d, 8.9, 26-NH), 3.63 (dd, 10.4 and 7.9, H-32), 1.82 (m, H-33a), 1.53 (m, H-33b), 1.09 (m, H-34), 0.81 (d, 6.4, H-35, H-34-Me), 3.05 (s, H-52), 4.06 (dd, 7.0 and 6.1, H-37), 1.12 (d, 7.0, H-38), 7.36 (d, 6.1, 37-NH), 2.57 (m, H-40a), 2.22 (m, H-40b), 4.46 (m, H-41), 3.99 (bs, H-42), 5.32 (dq, 6.4 and 3.0, H-43), 1.26 (d, 6.4, H-44), 8.53 (d, 9.8, 42-NH), 4.63 (dd, 8.5 and 5.8, H-46), 1.73 (m, H-47a), 1.68 (m, H-47b), 1.78 (m, H-48), 1.07 (d, 6.4, H-49), 1.02 (d, 6.4, H-50), 8.41 (d, 6.7, 46-NH) and 8.15 (s, H-51). ^{13}C NMR (100 MHz, CD_3OD): 173.62 (C-1), 54.78 (C-2), 34.22 (C-3a), 20.71 (C-4a), 13.69 (C-5), 172.65 (C-6), 45.21 (C-7a), 175.7 (C-8), 52.69 (C-9), 39.31 (C-10a), 137.16 (C-11), 130.63 (C-12, C-16), 129.8 (C-13, C-15), 128.29 (C-14), 172.65 (C-17), 58.74 (C-18), 69.33 (C-19), 19.65 (C-20), 174.59 (C-21), 50.8 (C-22), 30.46 (C-23a), 173.43 (C-25), 56.73 (C-26), 42.22 (C-27), 59.18 (C-28), 23.39 (C-29), 10.19 (C-30),

	174.66 (C-31), 64.84 (C-32), 39.01 (C-33a), 25.74 (C-34), 24.03 (C-35), 21.94 (C-34-Me), 39.56 (C-52), 174.59 (C-36), 51.43 (C-37), 17.74 (C-38), 172.46 (C-39), 40.94 (C-40a), 72.31 (C-41), 56.73 (C-42), 75.6 (C-43), 19.33 (C-44), 175.28 (C-45), 52.69 (C-46), 42.9 (C-47a), 26.16 (C-48), 23.35 (C-49), 22.7 (C-50) and 163.71 (C-51). [85]

Table S7. Polyketides isolated from *Callyspongia* species.

Metabolite name<br>Chemical formula<br>Type of metabolite	Specie<br>Geographic Location	^{1}H and ^{13}C NMR data [Reference]
Callystatin A (**189**)<br>$C_{29}H_{44}O_4$<br>Polyketide	*Callyspongia truncata*<br>Japan	^{1}H NMR (500 MHz, $CDCl_3$, δ/ppm, *J*/Hz): 6.06 (dd, 9.6 and 1.7, H-2), 6.90 (dt-like, 9.6 and 4.2, H-3), 2.47 (m, H-4), 4.98 (dt-like, 14.2 and 6.8, H-5), 5.76 (dd, 16.0 and 6.8, H-6), 6.63 (d, 16.0, H-7), 5.24 (d, 9.6, H-9), 2.67 (m, H-10), 2.08 (t-like, 7.4, H-11), 5.57 (dt, 15.4 and 7.4, H-12), 6.01 (d, 15.4, H-13), 5.13 (dq, 9.8 and 1.2, H-15), 3.66 (dq, 9.8 and 6.6, H-16), 2.85 (qd, 7.1 and 4.3, H-18), 3.57 (dd, 6.6 and 4.3, H-19), 1.32 (m, H-20), 1.05 (m, H-21), 0.84 (t, 7.3, H-22), 2.18 (q, 6.9, H-23), 1.04 (t, 6.9, H-24), 0.96 (d, 7.6, H-25), 1.82 (d, 1.2, H-26), 1.13 (d, 6.6, H-27), 1.11 (d, 7.1, H-28) and 0.88 (d, 6.4, H-29). ^{13}C NMR (125 MHz, $CDCl_3$): 164.1 (s, C-1), 121.7 (d, C-2), 144.7 (d, C-3), 30.1 (t, C-4), 78.9 (d, C-5), 124.7 (d, C-6), 129.9 (d, C-7), 135.3 (s, C-8), 137.1 (d, C-9), 32.1 (d, C-10), 40.8 (t, C-11), 127.7 (d, C-12), 135.4 (d, C-13), 136.2 (s, C-14), 128.3 (d, C-15), 45.6 (d, C-16), 216.4 (s, C-17), 45.7 (d, C-18), 74.4 (d, C-19), 36.7 (d, C-20), 25.8 (t, C-21), 10.9 (q, C-22), 26.4 (t, C-23), 13.4 (q, C-24), 20.7 (q, C-25), 13.0 (q, C-26), 16.1 (q, C-27), 11.2 (q, C-28) and 14.2 (q, C-29). [86]
	Callyspongia truncata<br>Not reported	^{1}H NMR (500 MHz, C_6D_6, δ/ppm, *J*/Hz): 6.61 (d, 16, H-7), 6.06 (d, 16, H-13), 5.90 (ddd, 10, 2 and 5.5, H-3), 5.81 (dd, 10 and 2, H-2), 5.62 (dt, 16 and 8, H-12), 5.55 (dd, 16 and 6.5, H-6), 5.25 (d, 10, H-15), 5.23 (d, 10, H-9), 4.43 (m, H-5), 3.76 (t-like, 6, H-19), 3.55 (dq, 10 and 7, H-16), 2.84 (dd, 7 and 5.5, H-18), 2.67 (m, H-10), 2.20 (m, $H_{2\text{-}4}$), 2.13 (m, 1'-H_2), 2.07 (dd, 6 and 8, H-l1), 1.77 (s, 14-CH_3), 1.50 (m, $H_{2\text{-}21}$), 1.30 (m, H-20), 1.14 (d, 7, 16-CH_3), 1.07 (t, 7.5, 2'-H_3), 1.04 (d, 7, 18-CH_3), 0.98 (d, 7, 10-CH_3), 0.97 (d, 6.5, 20-CH_3) and 0.87 (t, 7.5, $H_{3\text{-}22}$). [87]
Comantherin (**190**)<br>$C_{16}H_{14}O_5$<br>Linear naphthopyrone	*Callyspongia* sp.<br>Australia	Spectroscopic data not provided. [80]
Callyspongiolide (**191**)<br>$C_{33}H_{42}BrNO_6$<br>Macrolide	*Callyspongia* sp.<br>Indonesia	^{1}H NMR (600 MHz, DMSO-d_6, δ/ppm, *J*/Hz): 5.93 (dd, 12.0 and 2.6, H-2), 6.13 (td, 12.0 and 3.4, H-3), 3.41 (ddd, 14.8, 12.6 and 4.8, H-4), 1.86 (dq, 14.8 and 3.0, H-4), 1.75 (m, H-5), 1.37 (ddd, 14.2, 11.4 and 3.0,

		H-6), 1.01 (overlapped, H-6), 4.47 (br dd, 11.4 and 10.1, H-7), 1.41 (ddd, 14.4, 10.1 and 1.6, H-8), 1.03 (overlapped, H-8), 2.00 (m, H-9), 5.06 (dd, 15.0 and 9.1, H-10), 5.22 (dd, 15.0 and 9.3, H-11), 2.24 (m, H-12), 5.09 (dd, 10.3 and 7.7, H-13), 6.06 (dd, 15.8 and 7.7, H-14), 5.94 (dd, 15.8 and 2.2, H-15), 5.46 (dd, 16.4 and 2.2, H-18), 6.36 (d, 16.4, H-19), 4.89 (d, 4.4, H-21), 6.83 (dd, 7.9 and 1.6, H-25), 7.13 (t, 7.9, H-26), 6.84 (dd, 7.9 and 1.6, H-27), 1.04 (s, H-28), 0.96 (s, H-29), 0.97 (d, 7.1, H-30), 0.87 (d, 6.8, H-31), 0.89 (d, 6.8, H-32), 5.49 (d, 4.4, OH-21) and 10.04 (s, OH-24). ^{13}C NMR (150 MHz, DMSO-d_6): 164.2 (C-1), 122.3 (C-2), 142.5 (C-3), 31.3 (C-4), 26.9 (C-5), 41.1 (C-6), 68.3 (C-7), 44.1 (C-8), 33.2 (C-9), 136.4 (C-10), 132.0 (C-11), 41.8 (C-12), 75.7 (C-13), 139.6 (C-14), 113.4 (C-15), 86.3 (C-16), 90.4 (C-17), 106.8 (C-18), 151.6 (C-19), 43.0 (C-20), 76.5 (C-21), 143.2 (C-22), 111.7 (C-23), 153.3 (C-24), 114.3 (C-25), 126.9 (C-26), 120.1 (C-27), 24.1 (C-28), 22.4 (C-29), 19.9 (C-30), 22.0 (C-31), 17.4 (C-32) and 156.7 (C-33). [88]
5-hydroxy-3-methyl-5-pentyl-2,5-dihydrofuran-2-one (**192**) $C_{10}H_{16}O_3$ Butenolide	*Callyspongia vaginalis* Barbados	^{1}H NMR* ($CDCl_3$, δ/ppm, *J*/Hz): 6.83 (1H, br s, H-4), 1.90 (2H, t, 7.2, H-6), 1.40 (2H, t, 7.2, H-7), 1.30 (2H, m, H-8), 1.31 (2H, m, H-9), 0.89 (3H, t, 5.2, H-10) and 1.94 (3H, s, H-11). ^{13}C NMR ($CDCl_3$): 171.55 (C-2), 132.39 (C-3), 146.80 (C-4), 121.70 (C-5), 37.68 (C-6), 22.99 (C-7), 31.54 (C-8), 22.42 (C-9), 13.82 (C-10) and 10.40 (C-11). [9]
Hydroxydihydrobovolide (**193**) $C_{11}H_{18}O_3$ Dihydrofuran	*Callyspongia* sp. China	^{1}H NMR (270 MHz, $CDCl_3$, δ/ppm, *J*/Hz): 3.04 (1H, br s, OH), 2.00 (1H, ddd, 11.4, 4.4 and 2.2), 1.94 (3H, q, 1.1), 1.82 (3H, q, 1.1), 1.75 (1H, ddd, 11.4, 9.2 and 4.8), 1.28 (6H, m) and 0.88 (3H, t, 6.4). [67,89[1]]
(−)-loliolide (**194**) $C_{10}H_{16}O_3$ Benzofuran[2]	*Callyspongia* sp. China	^{1}H NMR (300 MHz, $CDCl_3$, δ/ppm, *J*/Hz): 1.27 (s, 3H, Me), 1.47 (s, 3H, Me), 1.52 (dd, 1H, 14.5 and 3.8), 1.78 (dd, 1H, 14.5 and 3.8), 1.79 (s, 3H, Me), 2.00 (ddd, 1H, 14.5, 2.8 and 2.8), 2.19 (d, 1H, 2, OH), 2.47 (ddd, 1H, 13.9, 2.7 and 2.7), 4.32 (m, 1H, CHOH) and 5.68 (s, 1H, =CH). ^{13}C NMR (75.4 MHz, $CDCl_3$): 26.5, 27.0, 30.7 (Me), 36.0 (C-4), 45.6, 47.3 (CH_2), 66.6 (C-6), 87.0 (C-7a), 112.8 (C-3), 172.2 (C-3a) and 182.9 (C-2). [67,90[1]]

* Magnetic field strength did not report in the reference.
[1] Metabolite NMR data.
[2] Classification based on the electronic database The Human Metabolome Database (HMDB) Version 4.0.

Table S8. Miscellaneous compounds isolated from *Callyspongia* species.

Metabolite name<br>Chemical formula<br>Type of metabolite	Specie<br>Geographic Location	^{1}H and ^{13}C NMR data [Reference]
Callyspongidic acid C12:0 (**195**)<br>$C_{21}H_{32}O_6$<br>Amphiphilic Diacid	*Callyspongia californica*<br>Ecuador	^{1}H NMR (500 MHz, CD_3OD, δ/ppm, *J*/Hz): 2.82 (m, H-2), 1.89 (m, H-3a), 1.41 (m, H-3b), 1.37 (m, H-4a), 1.26 (m, H-4b), 1.30 (m, H-5), 1.29 (m, H-6), 1.30 (m, H-7), 1.29 (m, H-8), 1.29 (m, H-9), 1.28 (m, H-10), 1.32 (m, H-11), 0.90 (t, 6.8, H-12), 3.05 (d, 13.7, H-3′a), 2.81 (d, 13.7, H-3′b), 7.05 (d, 8.2, H-5′, H-9′) and 6.64 (d, 8.2, H-6′, H-8′). ^{13}C NMR (125 MHz, CD_3OD): 177.5 (C-1), 54.7 (C-2), 29.1 (C-3), 28.8 (C-4), 30.4 (C-5), 30.7 (C-6), 30.5 (C-7), 30.5 (C-8), 30.7 (C-9), 33.1 (C-10), 23.7 (C-11), 14.4 (C-12), 176.8 (C-1′), 80.4 (C-2′), 44.0 (C-3′), 128.1 (C-4′), 132.5 (C-5′, C-9′), 115.7 (C-6′, C-8′) and 157.2 (C-7′). [12]
Callyspongidic acid C13:0 (**196**)<br>$C_{22}H_{34}O_6$<br>Amphiphilic Diacid	*Callyspongia californica*<br>Ecuador	^{1}H NMR (500 MHz, CD_3OD, δ/ppm, *J*/Hz): 2.82 (m, H-2), 1.86 (m, H-3a), 1.39 (m, H-3b), 1.36 (m, H-4a), 1.26 (m, H-4b), 1.33 (m, H-5), 1.30 (m, H-6), 1.30 (m, H-7), 1.32 (m, H-8), 1.32 (m, H-9), 1.29 (m, H-10), 1.26 (m, H-11), 1.32 (m, H-12), 0.90 (t, 6.9, H-13), 3.05 (d, 13.7, H-3′a), 2.81 (d, 13.7, H-3′b), 7.05 (d, 8.4, H-5′, H-9′) and 6.64 (d, 8.4, H-6′, H-8′). ^{13}C NMR (125 MHz, CD_3OD): 177.5 (C-1), 54.8 (C-2), 29.2 (C-3), 28.9 (C-4), 30.5 (C-5), 30.7 (C-6), 30.8 (C-7), 30.5 (C-8), 30.5 (C-9), 30.8 (C-10), 33.1 (C-11), 23.7 (C-12), 14.4 (C-13), 176.9 (C-1′), 80.4 (C-2′), 44.0 (C-3′), 128.2 (C-4′), 132.5 (C-5′, C-9′), 115.7 (C-6′, C-8′) and 157.2 (C-7′). [12]
Callyspongidic acid C14:0 (**197**)<br>$C_{23}H_{36}O_6$<br>Amphiphilic Diacid	*Callyspongia californica*<br>Ecuador	^{1}H NMR (500 MHz, CD_3OD, δ/ppm, *J*/Hz): 2.82 (m, H-2), 1.88 (m, H-3a), 1.38 (m, H-3b), 1.36 (m, H-4a), 1.24 (m, H-4b), 1.32 (m, H-5), 1.32 (m, H-6), 1.30 (m, H-7), 1.29 (m, H-8), 1.30 (m, H-9), 1.33 (m, H-10), 1.29 (m, H-11), 1.26 (m, H-12), 1.31 (m, H-13), 0.90 (t, 6.8, H-14), 3.05 (d, 13.7, H-3′a), 2.81 (d, 13.7, H-3′b), 7.06 (d,

		8.4, H-5′, H-9′) and 6.64 (d, 8.5, H-6′, H-8′). ^{13}C NMR (125 MHz, CD_3OD): 177.6 (C-1), 54.7 (C-2), 29.1 (C-3), 28.8 (C-4), 30.5 (C-5), 30.5 (C-6), 30.7 (C-7), 30.8 (C-8), 30.7 (C-9), 30.5 (C-10), 30.8 (C-11), 33.1 (C-12), 23.7 (C-13), 14.4 (C-14), 176.8 (C-1′), 80.3 (C-2′), 44.0 (C-3′), 128.2 (C-4′), 132.5 (C-5′, C-9′), 115.7 (C-6′, C-8′) and 157.2 (C-7′). [12]
Callyspongidic acid C14:1 (**198**)<br>$C_{23}H_{34}O_6$<br>Amphiphilic Diacid	*Callyspongia californica*<br>Ecuador	^1H NMR (500 MHz, CD_3OD, δ/ppm, *J*/Hz): 2.82 (m, H-2), 1.89 (m, H-3a), 1.41 (m, H-3b), 1.37 (m, H-4a), 1.26 (m, H-4b), 1.32 (m, H-5), 2.03 (m, H-6), 5.34 (m, H-7), 5.35 (m, H-8), 2.02 (m, H-9), 1.35 (m, H-10), 1.30 (m, H-11), 1.28 (m, H-12), 1.32 (m, H-13), 0.91 (t, 6.8, H-14), 3.06 (d, 13.7, H-3′a), 2.80 (d, 13.6, H-3′b), 7.06 (d, 8.3, H-5′, H-9′) and 6.64 (d, 8.4, H-6′, H-8′). ^{13}C NMR (125 MHz, CD_3OD): 178.0 (C-1), 54.7 (C-2), 29.1 (C-3), 28.8 (C-4), 30.0 (C-5), 27.9 (C-6), 130.5 (C-7), 131.1 (C-8), 28.1 (C-9), 30.9 (C-10), 30.4 (C-11), 32.9 (C-12), 23.7 (C-13), 14.4 (C-14), 177.2 (C-1′), 80.6 (C-2′), 44.0 (C-3′), 128.4 (C-4′), 132.5 (C-5′, C-9′), 115.6 (C-6′, C-8′) and 157.1 (C-7′). [12]
2-(3-Methyl-dec-3-enamido)ethanesulfonic acid (**199**)<br>$C_{13}H_{25}NO_4S$<br>*N*-acyl taurine	*Callyspongia* sp.<br>China	^1H NMR (500 MHz, $CDCl_3$, δ/ppm, *J*/Hz): 5.36 (1H, t, 6.5, H-4), 3.63 (2H, t, 7.0, H-1′), 3.00 (2H, t, 7.0, H-2′), 2.91 (2H, s, H-2), 2.07 (2H, m, H-5), 1.77 (3H, s, H-11), 1.18–1.27 (8H, overlap, H-6-9) and 0.93 (3H, t, 6.5, H-10). ^{13}C NMR (125 MHz, $CDCl_3$): 174.6 (C-1), 131.2 (C-4), 130.2 (C-3), 51.5 (C-2′), 48.8 (C-2), 36.6 (C-1′), 29.2 (C-5), 23.7-30.8 (C-6-9), 16.3 (C-11) and 14.6 (C-10). [91]
Callyspongiamide A (**200**)<br>$C_{16}H_{26}Cl_6N_2O_2$<br>Polychlorine-containing modified dipeptides	*Callyspongia* sp.<br>Indonesia	^1H NMR (400 MHz, $CDCl_3$, δ/ppm, *J*/Hz): 3.22 (t, 6.8, H-2), 1.76 (m, H-3), 2.36 (m, H-3), 2.73 (m, H-4), 1.38 (d, 6.3, H-5), 2.37 (s, H-7 and H-8), 4.70 (ddd, 11.7, 8.8 and 2.7, H-2′), 7.44 (d, 8.8, 2-NH), 1.82 (m, H-3′), 2.11 (dd, 12.9 and 11.7, H-3′), 2.54 (m, H-4′), 1.46 (d, 6.3, H-5′), 2.64 (m, H-7′) and 1.09 (t, 7.1, H-8′).

		^{13}C NMR (125 MHz, $CDCl_3$): 172.7 (C-1), 66.9 (C-2), 30.3 (C-3), 53.0 (C-4), 17.2 (C-5), 106.4 (C-6), 41.9 (C-7 and C-8), 208.6 (C-1′), 55.2 (C-2′), 35.8 (C-3′), 52.1 (C-4′), 16.4 (C-5′), 105.4 (C-6′), 33.4 (C-7′) and 7.6 (C-8′). [6]
Callyspongiamide B (**201**)<br>$C_{16}H_{19}Cl_6NO_2$<br>Polychlorine-containing modified dipeptides	*Callyspongia* sp.<br>Indonesia	^{1}H NMR (400 MHz, $CDCl_3$, δ/ppm, *J*/Hz): 6.03 (d, 15.5, H-2), 6.94 (d, 15.5 and 8.0, H-3), 3.37 (br q, 7.1, H-4), 1.47 (d, 6.3, H-5), 4.85 (m, H-2′), 6.18 (d, 7.8, 2-NH), 1.85 (ddd, 13.8, 11.7 and 3.0, H-3′), 2.12 (dd, 12.7 and 11.7, H-3′), 2.57 (m, H-4′), 1.47 (d, 6.3, H-5′), 2.66 (m, H-7′) and 1.11 (t, 7.1, H-8′). ^{13}C NMR (125 MHz, $CDCl_3$): 164.2 (C-1), 126.5 (C-2), 142.2 (C-3), 57.3 (C-4), 17.3 (C-5), 104.2 (C-6), 208.7 (C-1′), 55.4 (C-2′), 36.0 (C-3′), 51.7 (C-4′), 16.5 (C-5′), 105.4 (C-6′), 33.5 (C-7′) and 7.6 (C-8′). [6]
Bastadin 6 (**202**)<br>$C_{34}H_{26}Br_6N_4O_8$<br>Bastadin derivative	*Callyspongia* sp.<br>Australia	^{1}H NMR (270 MHz, $(CD_3)_2SO$, δ/ppm, *J*/Hz): 7.08 (d, 1.7, H-19), 10.00 (s, 15(OH)), 6.23 (d, 1.7, H-17), 2.72 (m, H-20), 3.30 (m, H-21), 8.13 (t, 6, H-22), 11.92 (s, 24(NOH)), 3.66 (s, H-25), 7.64 (s, H-27, H-31), 7.65 (s, H-8, H-12), 2.72 (m, H-6), 3.30 (m, H-5), 8.00 (t, 6, H-4), 11.71 (s, 2(NOH)), 3.57 (s, H-1), 7.04 (d, 1.9, H-36), 10.00 (s, 34(OH)) and 6.16 (d, 1.9, H-38). [80,93[1]]
Bastadin 7 (**203**)<br>$C_{34}H_{26}Br_6N_4O_9$<br>Bastadin derivative	*Callyspongia* sp.<br>Australia	^{1}H NMR (500 MHz, CD_3CN, δ/ppm, *J*/Hz): 3.57 (d, 12.8, H-1), 3.63 (d, 12.8, H-1), 6.89 (t, 6.1, 4-NH), 3.34 (m, H-5), 3.51 (m, H-5), 2.75 (m, H-6), 7.46 (br s, H-8, H-12), 7.10 (d, 1.8, H-17), 6.25 (d, 1.8, H-19), 2.61 (m, H-20), 2.68 (m, H-20), 3.23 (m, H-21), 3.30 (m, H-21), 7.38 (t, 5.8, 22-NH), 6.17 (br s, H-25), 7.63 (br s, H-27, H-31), 7.14 (d, 1.8, H-36), 6.30 (d, 1.8, H-38), 4.47 (br s, OH-25), 7.41 (br s, OH-34), 7.46 (br s, OH-15), 9.87 (s, N-OH) and 10.15 (br s, N-OH). ^{13}C NMR (75 MHz, CD_3CN): 28.1 (C-1), 152.3 (C-2), 163.0 (C-3), 40.0 (C-5), 34.0 (C-6), 141.1 (C-7), 134.3 (C-8, C-12), 117.8 (C-9), 146.8

		(C-10), 117.8 (C-11), 145.1 (C-14), 142.2 (C-15), 109.7 (C-16), 127.2 (C-17), 131.7 (C-18), 112.8 (C-19), 34.0 (C-20), 40.5 (C-21), 164.0 (C-23), 153.9 (C-24), 64.2 (C-25), 142.4 (C-26), 130.1 (C-27, C-31), 118.2 (C-28), 147.7 (C-29), 118.2 (C-30), 145.2 (C-33), 142.0 (C-34), 109.3 (C-35), 127.5 (C-36), 130.4 (C-37) and 113.9 (C-38). [80,94[1]]
Bastadin 8 (**204**)<br>$C_{34}H_{26}Br_6N_4O_9$<br>Bastadin derivative	*Callyspongia* sp.<br>Australia	Spectroscopic data not provided. [80]
Bastadin 9 (**205**)<br>$C_{34}H_{27}Br_5N_4O_8$<br>Bastadin derivative	*Callyspongia* sp.<br>Australia	Spectroscopic data not provided. [80]
Bastadin 16 (**206**)<br>$C_{34}H_{28}Br_4N_4O_8$<br>Bastadin derivative	*Callyspongia* sp.<br>Australia	Spectroscopic data not provided. [80]
Bastadin 18 (**207**)<br>$C_{34}H_{28}Br_4N_4O_8$<br>Bastadin derivative	*Callyspongia* sp.<br>Australia	^1H NMR (500 MHz, CD_3OD, δ/ppm, *J*/Hz): 3.67 (2H, s, H-1), 3.30 (2H, m, 7, H-5), 2.55 (2H, t, 7, H-6), 7.39 (1H, d, 2.0, H-8), 6.82 (1H, d, 8.3, H-11), 7.06 (1H, dd, 8.3 and 2.1, H-12), 7.04 (1H, d, 2.0, H-17), 6.35 (1H, d, 2.0, H-19), 2.66 (2H, t, 6.5, H-20), 3.34 (2H, m, 7, H-21), 3.72 (2H, s, H-25), 7.42 (1H, d, 2.0, H-27), 6.67 (1H, d, 8.4, H-30), 6.94 (1H, dd, 8.5 and 2.1, H-31), 7.13 (1H, d, 1.9, H-36) and 6.46 (1H, d, 1.9, H-38). ^{13}C NMR (125 MHz, CD_3OD): 28.7 (C-1), 153.3 (C-2), 166.0 (C-3), 41.5 (C-5), 35.4 (C-6), 138.6 (C-7), 135.1 (C-8), 115.6 (C-9), 152.4 (C-10), 122.2 (C-11), 130.9 (C-12), 145.3 (C-14), 146.6 (C-15), 111.6 (C-16), 128.5 (C-17), 132.7 (C-18), 118.0 (C-19), 35.5 (C-20), 41.7 (C-21), 166.6 (C-23), 152.6 (C-24), 29.3 (C-25), 135.8 (C-26), 135.1 (C-27), 115.2 (C-28), 153.0 (C-29), 121.2 (C-30), 130.3 (C-31), 145.1 (C-33), 147.0 (C-34), 111.7 (C-35), 129.5 (C-36), 129.7 (C-37) and 118.6 (C-38). [80,95[1]]
Bastadin 24 (**208**)<br>$C_{34}H_{26}Br_4N_4O_8$<br>Bastadin derivative	*Callyspongia* sp.<br>Australia	^1H NMR (270 MHz, $(CD_3)_2SO$, δ/ppm, *J*/Hz): 7.20 (d, 2.0, H-19), 9.89 (s, 15(OH)), 6.43 (d, 2.0, H-17), 2.64 (t, 6, H-20), 3.24 (q, 6, H-21), 7.85 (6, H-22), 12.09

		(s, 24(NOH)), 3.71 (s, H-25), 7.44 (d, 1.9, H-27), 6.66 (d, 8.3, H-30), 7.10 (dd, 1.9 and 8.3, H-31), 7.67 (d, 1.9, H-8), 6.99 (d, 8.8, H-11), 7.45 (dd, 1.9 and 8.8, H-12), 6.42 (d, 15, H-6), 7.34 (dd, 10 and 15, H-5), 10.29 (d, 10, H-4), 11.99 (s, 2(NOH)), 3.50 (s, H-1), 7.09 (d, 1.5, H-36), 9.89 (s, 34(OH)) and 6.40 (d, 1.5, H-38). [80,93[1]]
[(3*S*,4*Z*,6*S*)-6-butyl-6-ethyl-4-ethylidene-1,2-dioxan-3-yl]acetic acid[2] (**209**)<br>$C_{14}H_{24}O_4$<br>Peroxide-containing acid	*Callyspongia* sp.<br>New Guinea	^{1}H NMR* ($CDCl_3$/1 drop D_2O, δ/ppm, *J*/Hz): 5.35 (dq, 2.2 and 7.0, H-11), 5.20 (dd, 8.8 and 5.0, H-3β), 3.05 (dd, 15.5 and 8.8, H-2α), 2.60 (dd, 15.5 and 5.0, H-2β), 2.40 (ddd, 14, 2.5 and 2.2, H-5*α*), 2.00 (d, 14, H-5β), 1.72 (br m, H-7), 1.65 (dd, 7 and 2.5, H-12), 1.56 (dq, 14.4 and 7.2, H-13), 1.42 (dq, 14.4 and 7.2, H-13′), 1.40 (m, H-7′), 1.30 (m, H-8, H-9 and H-9′), 1.17 (m, H-8′), 0.86 (t, 7.2, H-10) and 0.83 (t, 7.2, H-14). ^{13}C NMR ($CDCl_3$/1 drop D_2O): 174.2 (s, C-1), 131.1 (s, C-4), 122.2 (d, C-11), 84.6 (s, C-6), 75.8 (d, C-3), 38.1 (t, C-5), 36.0 (t, C-2), 31.5 (t), 29.0 (t), 25.2 (t), 23.2 (t), 14.2 (C-10), 12.7 (C-12) and 7.4 (C-14). [92]
[(3*S*,4*R*)-6-butyl-4,6-diethyl-1,2dioxan-3-yl]acetic acid[2] (**210**)<br>$C_{14}H_{26}O_4$<br>Peroxide-containing acid	*Callyspongia* sp.<br>New Guinea	^{1}H NMR* ($CDCl_3$/C_6D_6, δ/ppm, *J*/Hz): 4.02 (ddd, 10.5, 9.7 and 2.4, H-3β), 2.35 (dd, 16.5 and 2.4, H-2), 2.18 (bs, H-2′), 1.71 (bt, 13.8, H-7), 1.55 (dd, 13.4 and 4.5, H-5eq), 1.39 (m, H-4*α*, H-7′, H-13), 1.22 (m, H-8, 9, 9′, 11, 13), 1.03 (m, H-8′), 0.98 (dd, 13.4 and 12.0, H-5ax), 0.82 (t, 7.2, H-10), 0.81 (m, H-11′), 0.70 (t, 7.4, H-14) and 0.68 (t, 7.4, H-12). ^{13}C NMR ($CDCl_3$/C_6D_6): 177.5 (s, C-1), 83.7 (s, C-6), 82.5 (d, C-3), 37.0 (d, C-4), 37.0 (t, C-2), 36.8 (t, C-5), 32.1 (t, C-7), 30.2 (t, C-13), 26.1 (t, C-8), 24.9 (t, C-11), 23.9 (t, C-9), 14.7 (q, C-10), 11.1 (q, C-12) and 7.90 (q, C-14). [92]
Callypyrone A (**211**)<br>$C_{30}H_{44}O_8$<br>Callypyrone	*Callyspongia diffusa*<br>India	^{1}H NMR (500 MHz, $CDCl_3$, δ/ppm, *J*/Hz): 0.87 (3H, t, 6.8, H-1), 2.07 (2H, m, H-2), 2.69 (1Hβ, m, H-8), 3.67 (1H*α*, dd, 10.1 and 2.3, H-9), 2.32 (1H*α*, m, H-10), 4.07 (1H*α*, dd, 3.3 and 3.4, H-11), 2.55

		(1Hα, m, H-12), 3.04 (1Hβ, q, 6.5, H-14), 2.37 (1Hα, m, H-18), 3.42 (1Hα, m, H-19), 1.18 (3H, d, 6.0, H-20), 2.43 (3H, s, H-21, H-22 and H-27), 1.12 (3H, d, 7.1, H-23 and H-28), 0.94 (3H, d, 7.0, H-24 and H-25), 1.18 (3H, d, 6.0, H-26) and 2.23 (3H, s, H-2′). ^{13}C NMR (CD_3OD): 10.7 (C-1), 24.1 (C-2), 161.8 (C-3), 120.4 (C-4), 178.9 (C-5), 118.4 (C-6), 164.4 (C-7), 37.9 (C-8), 72.4 (C-9), 33.9 (C-10), 69.4 (C-11), 32.9 (C-12), 109.1 (C-13), 51.5 (C-14), 198.2 (C-15), 106.8 (C-16), 166.4 (C-17), 43.6 (C-18), 68.7 (C-19), 22.7 (C-20), 11.0 (C-21), 11.4 (C-22), 14.0 (C-23), 10.1 (C-24), 10.5 (C-25), 9.7 (C-26), 12.1 (C-27), 13.1 (C-28), 169.4 (C-1′) and 21.0 (C-2′). [26]
Callypyrone B (**212**)<br>$C_{28}H_{42}O_7$<br>Callypyrone	*Callyspongia diffusa*<br>India	^{1}H NMR (500 MHz, $CDCl_3$, δ/ppm, *J*/Hz): 0.88 (3H, t, 6.6, H-1), 2.00 (2H, m, H-2), 2.69 (1Hβ, m, H-8), 3.66 (1Hα, dd, 10.2 and 2.2, H-9), 1.83 (1Hα, m, H-10), 3.36 (1Hα, dd, 3.2 and 3.3, H-11), 2.11 (1Hα, m, H-12), 3.04 (1Hβ, q, 6.4, H-14), 2.33 (1Hα, m, H-18), 3.46 (1Hα, m, H-19), 1.18 (3H, d, 6.1, H-20), 2.44 (3H, s, H-21, H-22 and H-27), 1.10 (3H, d, 7.1, H-23 and H-28), 0.93 (3H, d, 7.2, H-24 and H-25) and 1.18 (3H, d, 7.2, H-26). ^{13}C NMR (CD_3OD): 10.7 (C-1), 24.0 (C-2), 161.4 (C-3), 121.7 (C-4), 178.6 (C-5), 118.7 (C-6), 164.1 (C-7), 37.6 (C-8), 73.0 (C-9), 38.6 (C-10), 74.7 (C-11), 36.3 (C-12), 109.4 (C-13), 51.2 (C-14), 198.9 (C-15), 105.8 (C-16), 166.4 (C-17), 43.9 (C-18), 64.4 (C-19), 21.7 (C-20), 11.1 (C-21), 11.7 (C-22), 14.0 (C-23), 10.1 (C-24), 9.8 (C-25), 9.4 (C-26), 12.1 (C-27) and 13.7 (C-28). [26]

[*] Magnetic field strength did not report in the reference.
[1] Metabolite NMR data.
[2] IUPAC nomenclature (Name not specified by the authors).
